# Bibliometric analysis of the effects of mental fatigue on athletic performance from 2001 to 2021

**DOI:** 10.3389/fpsyg.2022.1019417

**Published:** 2023-01-09

**Authors:** Xiao-Xin Chen, Zhi-Guang Ji, Yi Wang, Jing Xu, Li-Yan Wang, Hong-Biao Wang

**Affiliations:** ^1^School of Psychology, Shanghai University of Sport, Shanghai, China; ^2^Department of Physical Education, Shanghai University of Medicine and Health Sciences, Shanghai, China; ^3^College of Rehabilitation Science, Shanghai University of Medicine and Health Sciences, Shanghai, China

**Keywords:** mental fatigue, athletic performance, technical skill, decision-making, bibliometric analysis

## Abstract

**Aims:**

To explore the research hot topics and main contents in the field of the influence of mental fatigue on athletic performance, and to provide new ideas and directions for future research in this field.

**Methods:**

Using CiteSpace and VOSviewer visualization tool software core collection of Web of Science database to TS = (“mental fatigue” OR “mental exertion” OR “cognitive fatigue” OR “Cognitive exertion” OR “mental exhaustion” OR “mental tiredness”) AND (“athletic performance” OR “technical skill*” OR “Skill*” OR “technique” OR “decision making” OR “performance”) AND (“Humans”) searched for the influence of mental fatigue on athletic performance from 2001 to 2021 to conduct visual analysis. Research hot topics were analyzed from the aspects of high-impact countries/regions, institutions, authors, high-frequency keywords, and mutation terms.

**Results:**

A total of 658 publications were identified finally, and there has been an increasing trend in the annual number of publications, with the United States ranking first in the number of publications and influence. Future research will focus on promoting the application of EEG technology as an objective indicator for assessing mental fatigue, exploring effective methods and measures for pharmacological or non-pharmacological interventions against fatigue, and focusing on the effects of mental fatigue on endurance performance, technical skills, and sports-related decision-making.

**Conclusion:**

The results of the present study help us understand the status of the mental fatigue and athletic performance field and its recent developments.

## Introduction

Athletic performance is reflected in the process of the human body in motion a series of behaviors and psychological activities (Quan, 2016). From the perspective of sports training, Athletic performance is the comprehensive display of physical qualities required for a particular physical exercise (Liang-Yi, 2019). And it is one of the important factors that directly affect the final result and the result of the match. With the rapid development of competitive sports and the increasing scientificization of sports training, the sports world has put forward higher requirements for athletes’ abilities in all aspects, and the development of psychological skills has become a key part of athletes’ competitive level. In the field of sports, mental fatigue is a frequent problem faced by athletes and has a negative impact on the quality of their training and the success of their competitions.

Mental fatigue is a psychobiological state caused by prolonged periods of demanding cognitive (Boksem and Tops, 2008), manifested in subjective fatigue increasing conscious effort, cognitive task performance (accuracy and/or reaction time), and physical assessment mainly in terms of human electrophysiological parameters and biochemical indicators. In some special professionals, such as motor vehicle drivers, pilots, and athletes, mental fatigue increases the probability of making errors in their work or activities (McCormick et al., 2012). In addition, mental fatigue is also one of the main symptoms of patients with neurological disorders (Chaudhuri and Behan, 2004). Studies have shown that mental fatigue will impair endurance performance in humans (Hidenori et al., 2017; Schiphof-Godart et al., 2018; Slimani et al., 2018). It affects technical skills in some sports, such as football skills (Badin et al., 2016; Smith et al., 2017; Soylu et al., 2022), basketball skills (Moreira et al., 2018; Filipas et al., 2021; Fortes et al., 2022), table tennis skills (Mansec et al., 2017), and volleyball skills (Fortes et al., 2021), leading to a decrease in their performance; sport-related decision-making (Smith et al., 2016; Fortes et al., 2019, 2020; Gantois et al., 2020; Lei, 2021) is also affected by mental fatigue, and these abilities are related to athletic performance (Pageaux and Lepers, 2018). Therefore, mental fatigue will affect athletic performance in humans, including endurance performance, technical skills, and decision-making.

In recent years, studies have confirmed that the effects of mental fatigue on athletic performance have broad research prospects and application values (Wei and Jie, 2013; Pageaux and Lepers, 2018). Previous literature has systematically reviewed the effects of mental fatigue on athletic performance (Van Cutsem et al., 2017), and discussions on the combination of systematic review and meta-analysis (McMorris et al., 2018; Brown et al., 2020). However, there are only a few articles on this systematic review screening, and the discussion is only conducted unilaterally, lacking of more extensive important information in this field from the perspective of bibliometric analysis. Bibliometrics is a scientific method that uses mathematical statistics to analyze knowledge carrier, which can reveal the development trend of a particular research topic (Jan and Ludo, 2010; Singleton, 2010). CiteSpace and VOSviewer are Java Web-based data processing and visualization applications (Chao-Mei, 2004), which provide good support for research reviews. Web of Science database is a key source of input data for bibliometric analysis. By extracting keywords from title recognition, abstracts, descriptors, and bibliographic documents, CiteSpace and VOSviewer can categorize the frontier areas of current research (Shuo et al., 2019). So, this bibliometric analysis can fill the gaps in the research on the effects of mental fatigue on athletic performance from the perspective of visual analysis.

Considering the research gap and intended contribution, the study based its investigation upon the premises of the following research objectives:

- To identify the annual publications and the most representative disciplines and journals in the field of the effects of mental fatigue on athletic performance, and to discover influential national institutions and authors and their collaborations.
- By citing relevant data from literature and journals, grasp current research hot topics and explore existing problems.
- Briefly discuss the effects of mental fatigue on endurance performance, technical skills, and sport-related decision-making, to understand the current focus of research in this field and provide some reference and information for in-depth scientific research in the field of mental fatigue.

## Materials and methods

### Data collection

The search terms used to identify publications included: topic (“mental fatigue” OR “mental exertion” OR “cognitive fatigue” OR “cognitive exertion” OR “mental exhaustion” OR “mental tiredness”) AND topic (“athletic performance” OR “technical skill*” OR “skill*” OR “technique” OR “decision making” OR “performance”) AND topic (“Humans”) AND language (“English”) AND literature type (“article”). The search was conducted for publications between 2001 and 2021, and the date of the retrieval was 3 May 2022. The data for bibliometric analysis from the Web of Science Core Collection search index included SCI-EXPANDED, SSCI, A&HCI, CPCI-S, CPCI-SSH, BKCI-S, BKCI-SSH, ESCI, CCR-EXPANDED, and IC. We show the search strategy in Supplementary Appendix 1.

### Data selection

We deleted the duplicate publications, and three people read the abstracts. Finally, 658 publications were selected. We show all information included in the analysis in Supplementary Appendix 2.

### Inclusion criteria

Inclusion criteria were as follows: (1) original peer-reviewed published research on the effects of mental fatigue on athletic performance, including basic and clinical studies and (2) Articles published in Web of Science between 2001 and 2021.

### Exclusion criteria

Exclusion criteria were as follows: (1) publications involving plagiarism; (2) unpublished articles; (3) Meeting summaries, meeting minutes, and errata documents; (4) Irrelevant articles; and (5) Articles not retrieved or not retrieved from Web of Science during this period.

### Analysis methods and tools

CiteSpace is a software developed by Professor Chen Chao-Mei’s team. It has strong technical and functional advantages in mapping scientific knowledge maps in various scientific fields, analyzing citation networks with different characteristics and types, and identifying and presenting new trends and dynamics of scientific development In CiteSpace cooperation network visualization map, the outermost purple circles on behalf of each circle centricity purple ring is thicker, the higher its center position The outbreak of the red ring represents 1 year Line between the two circles on behalf of the partnership between the two countries. The color of the line on behalf of the cooperation of the close degree Color is yellow, the closer cooperation (Chen et al., 2015).

VOSviewer is a Java-based metrology tool developed by Van Eck and Waltman for co-occurrence network clustering and density analysis In VOSviewer’s cooperative network visualizations and co-sponsorship network visualizations, different colors represent different clusters, and lines between circles represent cooperative relationships between different points. In the average publication year graph, different colors correspond to different years. In the density visualizations, the redder the color, the higher the density (Kai-Gao, 2015).

## Results

### Annual trends in publications

**Figure 1:**
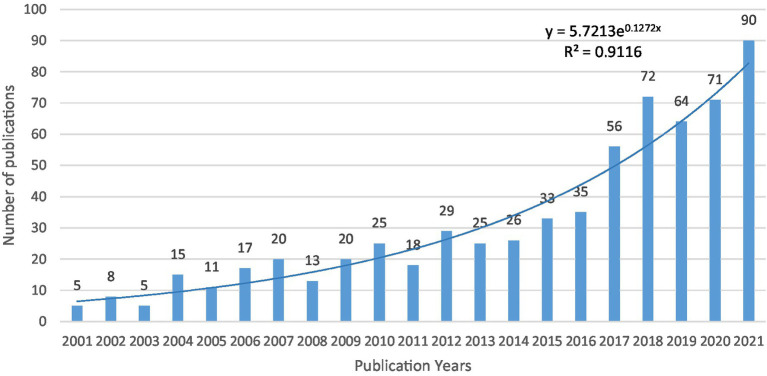
The combination chart of the number of annual publications.

Overall, 658 records published between 2001 and 2021 were analyzed, and as shown in Figure 1, the overall number of publications in studies of mental fatigue on athletic performance increased slowly and steadily. 2003–2015 showed a steady trend in the number of publications. 2016–2021 showed a rapid increase in the number of publications. The significant increase in the number of publications in the last 5 years indicates that the effect of mental fatigue on athletic performance is receiving increasing attention from researchers. To better understand the changing trend of the effect of mental fatigue on athletic performance, a curve is fitted between time and publications published annually using an exponential function. The equation presented in Figure 1 predicts the publications published yearly having an R-square of 94%.

### Analysis of category

**Figure 2:**
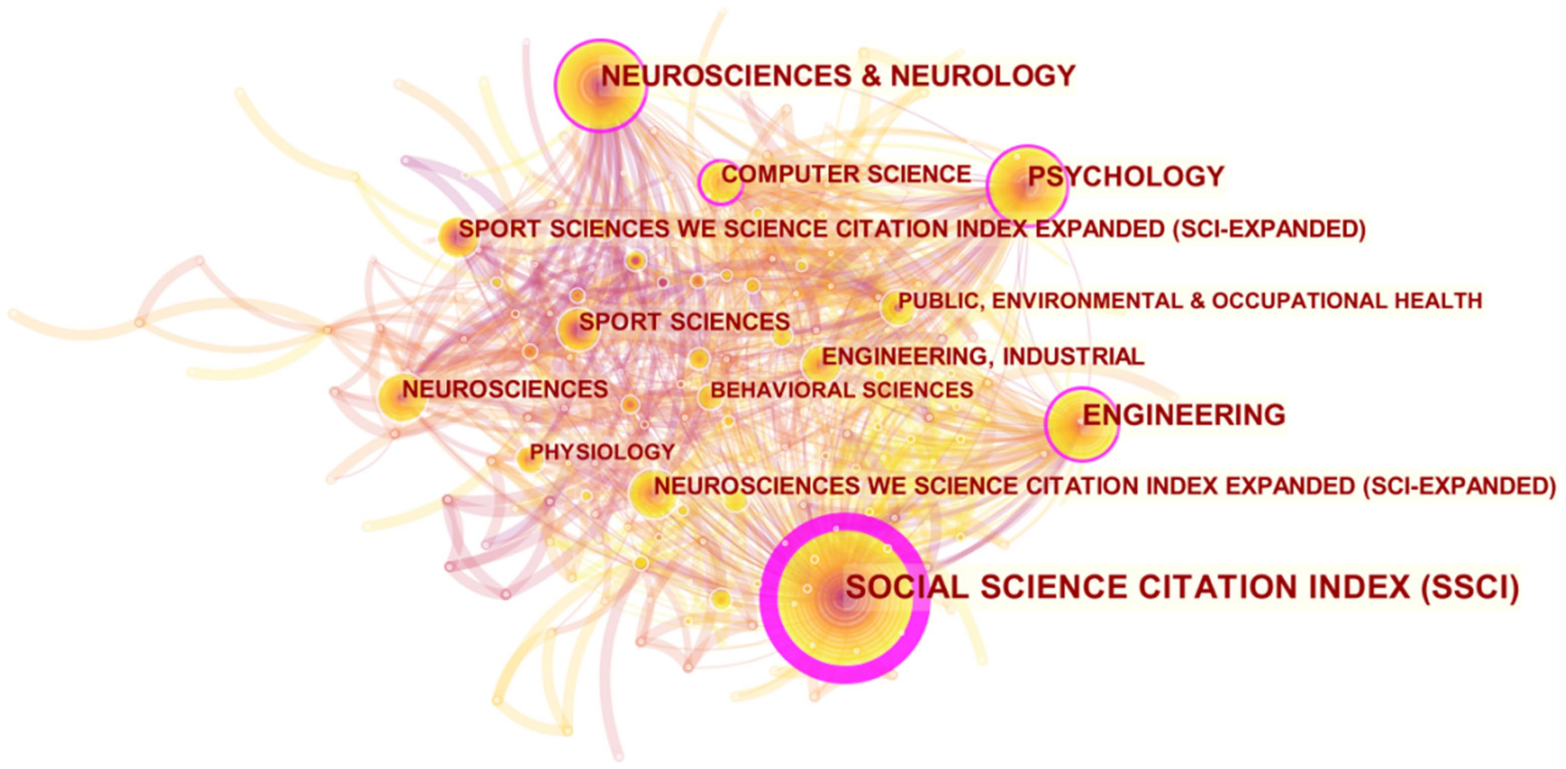
Main category regarding effects of mental fatigue on athletic performance.

Frequency and centrality of the top 10 topic categories related to mental fatigue and motor performance indexed in the Web of Science core collection illustrated by CiteSpace visualization calculations (see Figure 2; Table 1). Among them, the current effects of mental fatigue on athletic performance are mainly applied to four categories: social science (286), neuroscience (139), engineering (129), and psychology (124), as well as industrial management sports science and computer science In addition, the table shows that the effect of mental fatigue on sports performance is mainly applied in the social science (0.85).

**Table 1:** Top 10 main category regarding ding effects of mental fatigue on athletic performance.

Rank	Count	Centrality	Category
1	286	0.85	SOCIAL SCIENCE CITATION INDEX (SSCI)
2	129	0.2	ENGINEERING
2	124	0.2	PSYCHOLOGY
4	50	0.12	COMPUTER SCIENCE
5	139	0.1	NEUROSCIENCES & NEUROLOGY
5	27	0.1	PHARMACOLOGY & PHARMACY
7	17	0.08	CONFERENCE PROCEEDINGS CITATION INDEX – SCIENCE (CPCI-S)
8	5	0.06	FOOD SCIENCE & TECHNOLOGY
9	33	0.05	GENERAL & INTERNAL MEDICINE
9	19	0.05	ENVIRONMENTAL SCIENCES & ECOLOGY
9	15	0.05	HEALTH CARE SCIENCES & SERVICES

### Analysis of countries and regions

**Figure 3:**
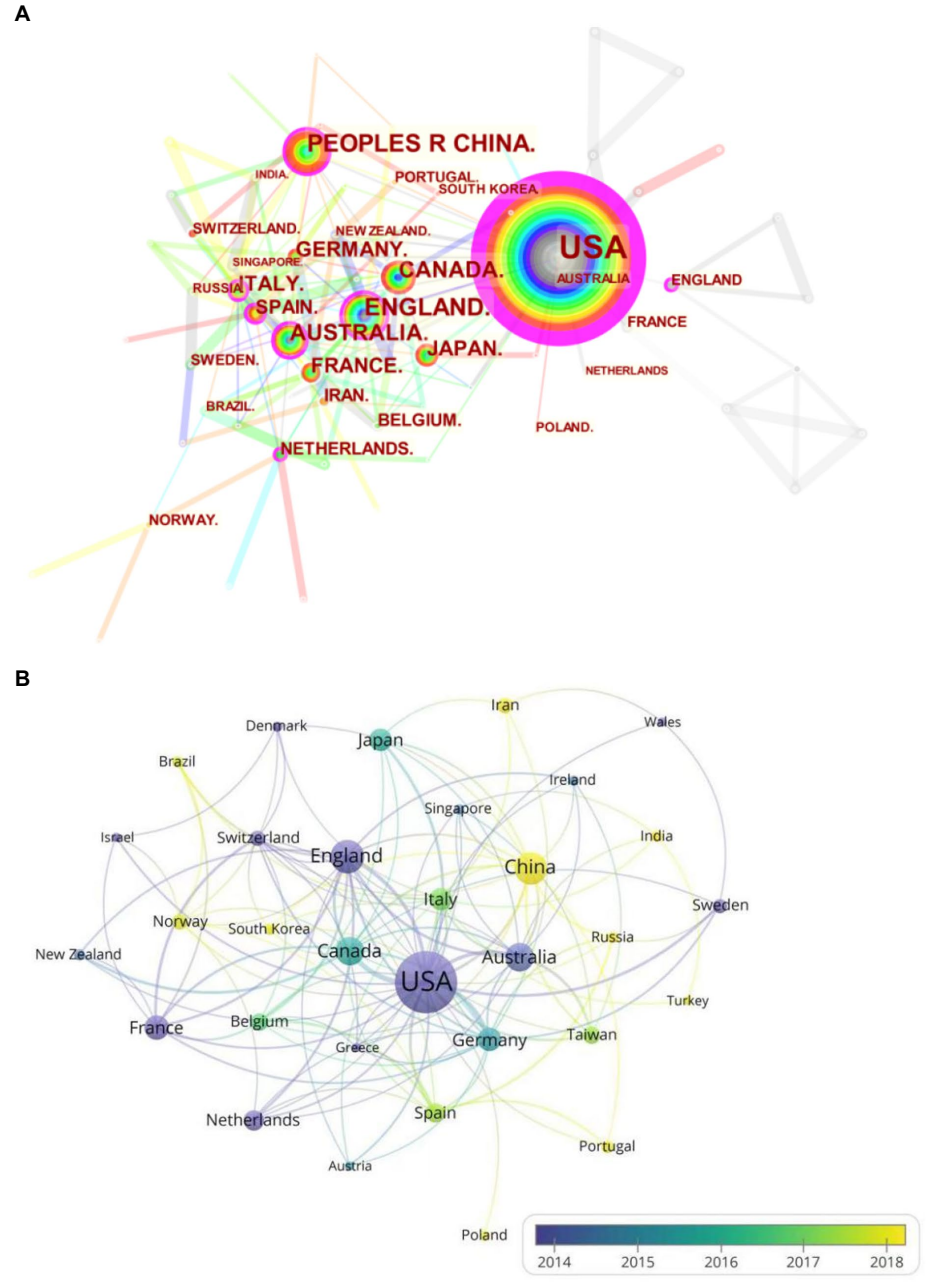
**(A)** The cooperation network visualizations map of countries and regions based on CiteSpace; **(B)** The average annual publication year map of countries and regions based on VOSviewer.

A total of 79 countries participated in the publication of the effects of mental fatigue on athletic performance. The density of national and regional cooperative networks and publications is shown on a map created by CiteSpace and VOSviewer (Figures 3A,B). Table 2 lists the top 10 most productive countries that published most of the publications. As shown in Table 2, the number of publications in this field was 224 in the United States, and 60 in China, followed by Britain (53), Canada (42), Australia (42), Japan (28), Germany (27), Italy (27), France (25), and Spain (21). There are more collaborations in the United States, China, the United Kingdom, Canada, and Germany. It can be seen that domestic and international research pays great attention to the “effect of mental fatigue on athletic performance,” and the research in this area has a large impact in the international arena.

**Table 2:** Top 10 countries/regions by number of publications regarding effects of mental fatigue on athletic performance.

Rank	Country	Counts	Centrality	First publication year
1	USA	224	0.78	2002
2	PEOPLES R CHINA	60	0.13	2008
3	ENGLAND	53	0.15	2008
4	CANADA	42	0.09	2008
5	AUSTRALIA	42	0.17	2007
6	JAPAN	28	0.02	2007
7	GERMANY	27	0.09	2009
8	ITALY	27	0.17	2011
9	FRANCE	25	0.06	2008
10	SPAIN	21	0.12	2010

### Analysis of institution

**Figure 4:**
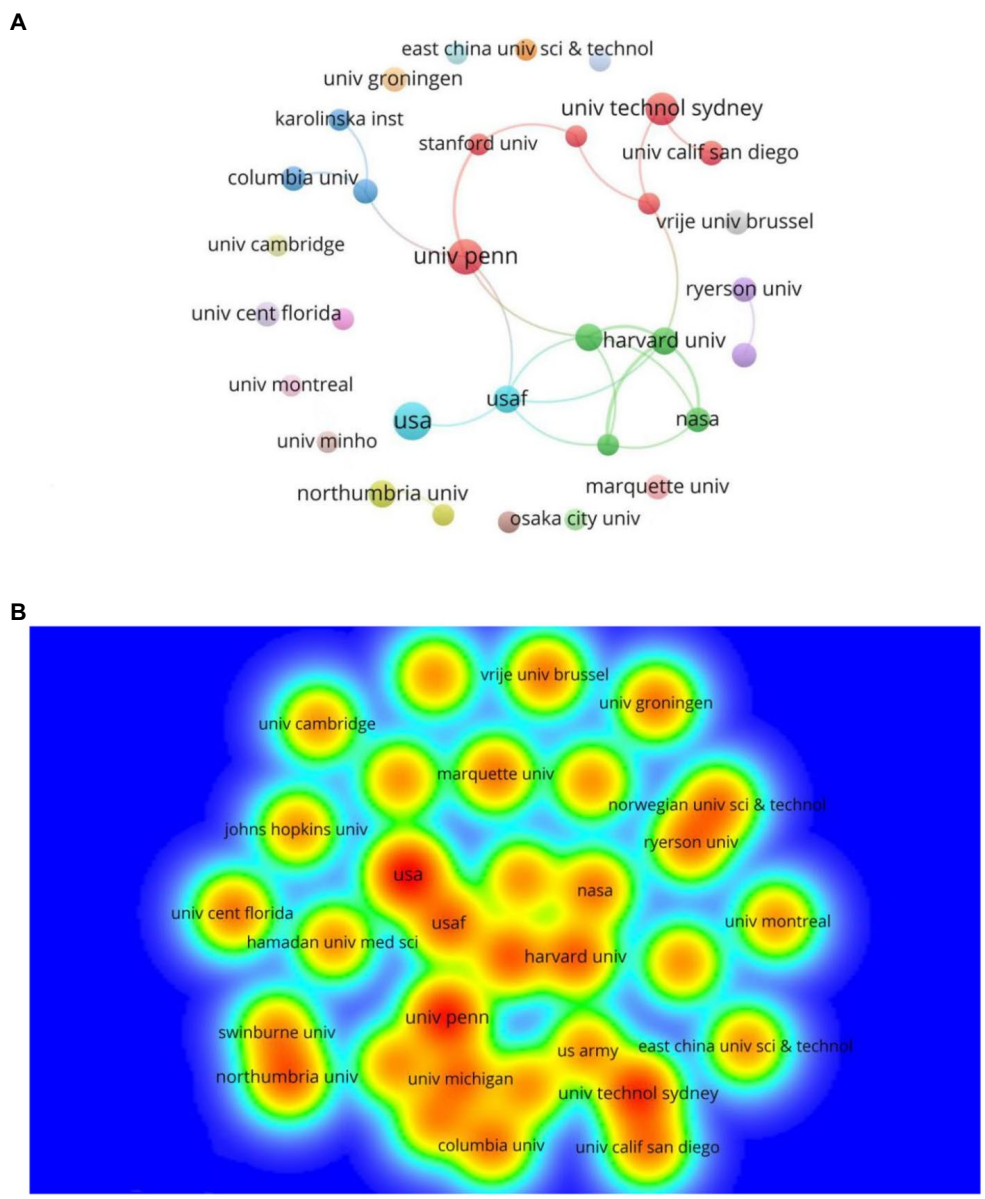
**(A)** The cooperation network visualization map of institutions based on VOSviewer; **(B)** The density visualization map of institutions based on VOSviewer.

A total of 473 institutions participated in the publication of the effects of mental fatigue on athletic performance. The most productive institutions can be found through the visualization maps (Figures 4A,B). Table 3 lists the Top 5 most productive institutions. As shown in Table 3, the United States has close ties with research institutions in Colombia, with more collaboration between the University of Pennsylvania and Stanford University, Harvard University, and the University of California, San Diego, forming a research team. As can be seen from the institutional collaborations, the main research is currently focused on collaborations mainly at the University of Pennsylvania, with fewer collaborations at other institutions, and a close institutional collaboration in this area has not yet been formed. As shown in Table 2, the average number of citations for the USA (11) was 90.82, the United States-based research institutions are in the lead and influential.

**Table 3:** Top 5 institutions by number of publications and total citations.

Rank	Institutions	Publication	Total citations	Average citation
1	USA	11	999	90.82
2	The University of Pennsylvania	10	601	60.1
3	University of Technology Sydney	9	339	37.67
4	Harvard University	7	85	12.14
4	Northumbria University	7	354	50.57
4	United States Air Force	7	129	18.43
4	Washington State University	7	115	16.43

### Analysis of journals

**Figure 5:**
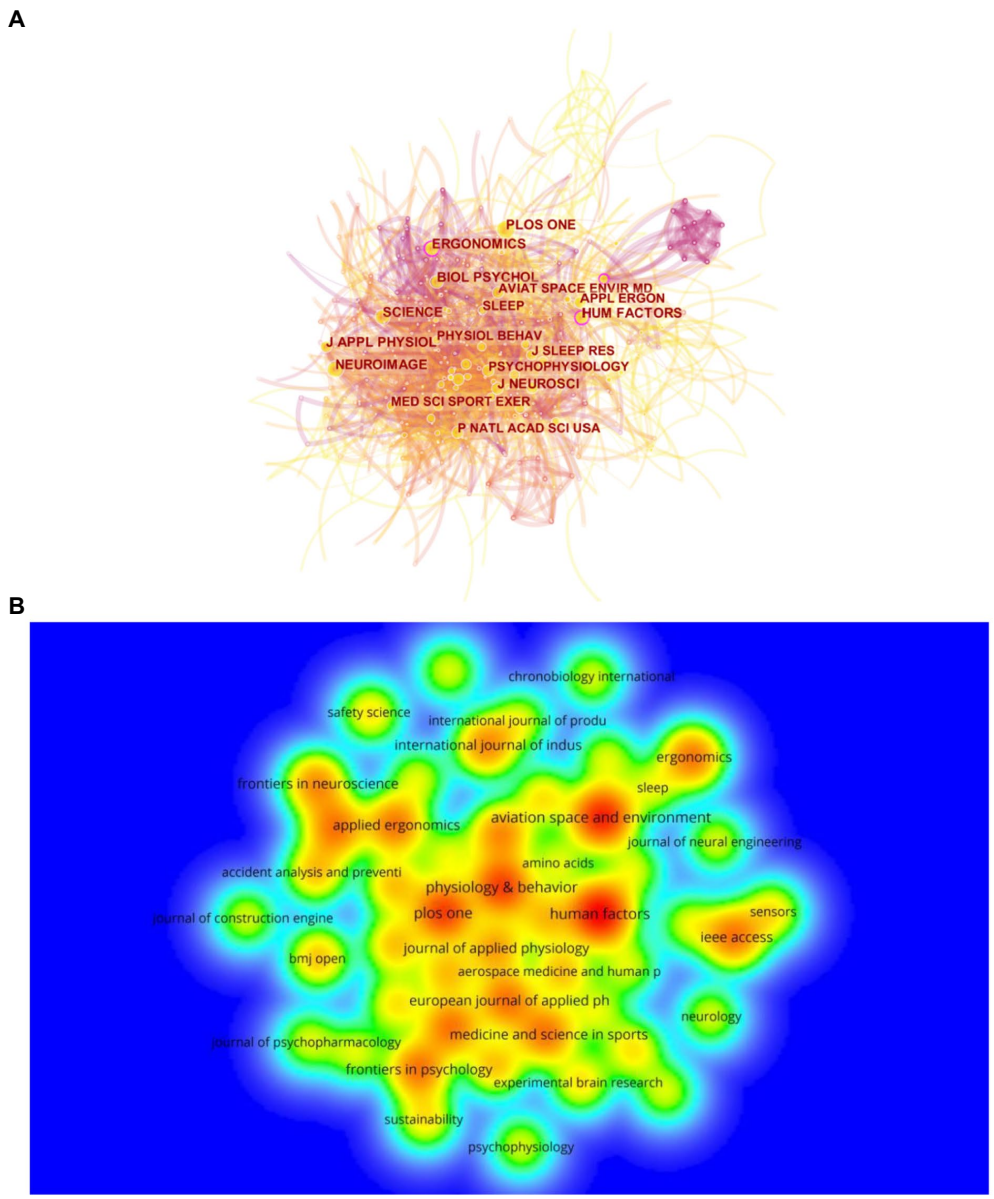
**(A)** The co-citation network visualization map of journals based on CiteSpace; **(B)** The density visualization map of journals based on VOSviewer.

A total of 257 journals were involved in the publishing the effects of mental fatigue on athletic performance. Table 4 lists the top 10 centrality journals regarding effects of mental fatigue on athletic performance. The journals are《ACCIDENT ANAL PREV》, 《PSYCHOPHARMACOLOGY》, 《ERGONOMICS》, 《BIOL PSYCHOL》, 《AVIAT SPACE ENVIR MD, and so on.《ERGONOMICS》and《HUM FACTORS》are the most important journals in this field in terms of number of articles, density, and citations (Figure 5). This means that the quality of publications in these journals is much higher than the average.

**Table 4:** The top 10 most centrality journals regarding effects of mental fatigue on athletic performance.

Rank	Centrality	Citations	Journal
1	0.13	94	ACCIDENT ANAL PREV
2	0.11	93	PSYCHOPHARMACOLOGY
3	0.08	163	ERGONOMICS
3	0.08	133	BIOL PSYCHOL
3	0.08	123	AVIAT SPACE ENVIR MD
6	0.07	128	HUM FACTORS
7	0.06	122	SLEEP
7	0.06	85	BRAIN RES
7	0.06	70	BRAIN RES REV
7	0.06	60	AM J PHYSIOL-REG I

### Analysis of authors

**Figure 6:**
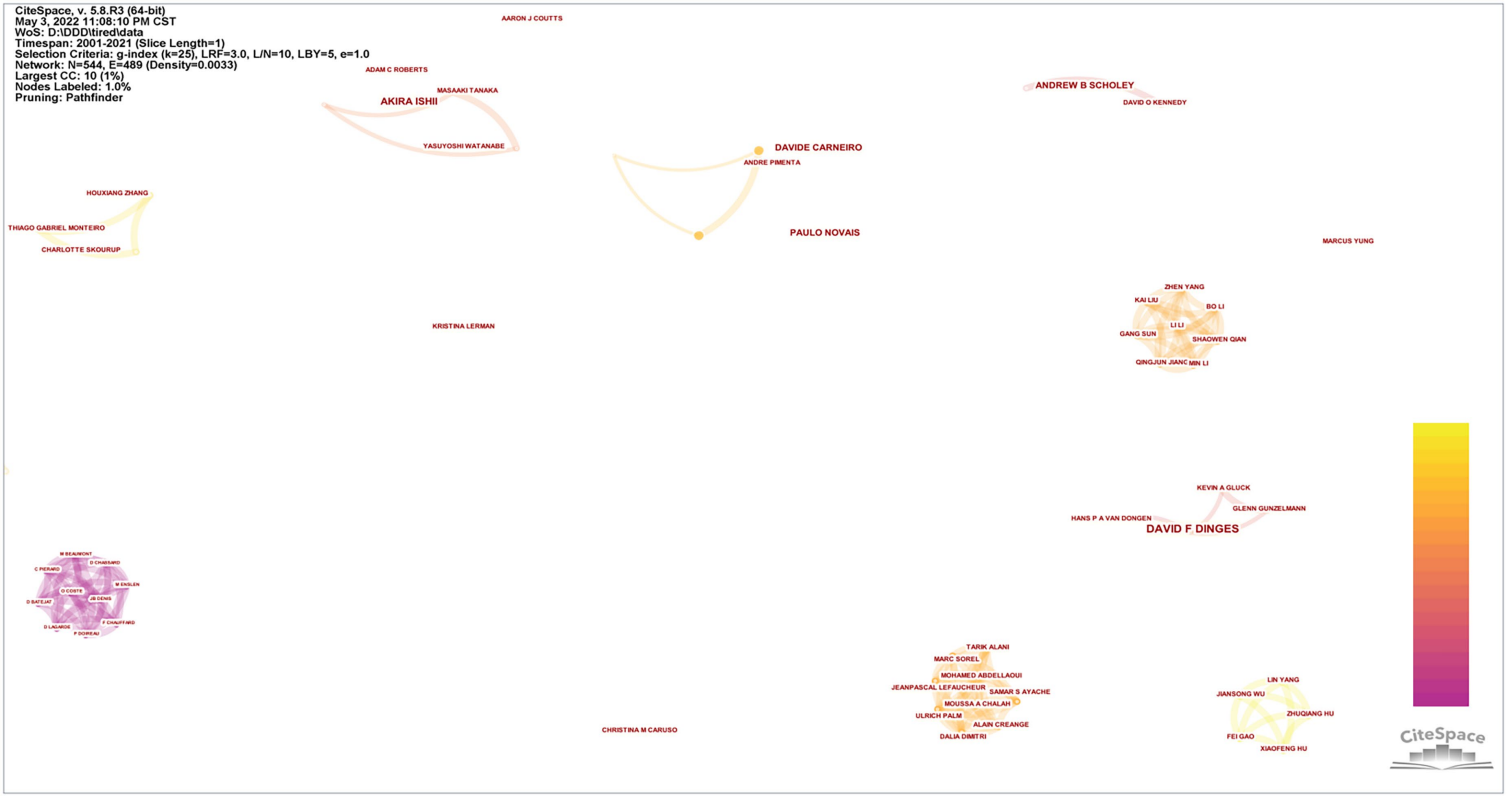
The cooperation network visualization map of authors based on CiteSpace.

Since 2000, a total of 544 researchers have participated in the publication of research in this field. Visualization maps can provide information on potential collaborators and can help researchers to establish collaborations (Figure 6). As shown in Figure 6, the research in this field has not yet taken the form of a relatively tight network, with independent authors and small co-authors predominating. Among them, the small-scale cooperation includes a 10-member team led by M BEAUMONT author, a 9-member team led by MARC SOREL author, and a 7-member team led by LILI author. In addition, the more influential teams are mainly those of DAVID F DINGES, AKIRA ISHI, and ANDREW B SCHOLEY authors. According to the number of publications that can be used as a measure of the depth of the author’s research in this field, the number of publications can be used as a basis for evaluating scientific talent. Table 5 lists the top 5 authors in the number of publications. These authors were Lieberman, Harris R. (8) Lin, Chin-Teng. (6), Dinges, David F. (5), and so on. As can be seen, none of the top 5 authors has more than 10 publications, indicating that research in this area is not widespread and is difficult to publish. The top five co-cited authors were Marcora, Samuele M., Lieberman, Harris R., Dinges, David F., Scholey, Andrew B., and Lieberman, Harris R. (Table 5).

**Table 5:** The top 5 authors in the number of publications and co-cited authors.

Rank	Authors	Publication	Rank	Authors	Co-citations
1	Lieberman, Harris R.	8	1	Marcora, Samuele M.	871
2	Lin, Chin-Teng	6	2	Lieberman, Harris R.	628
3	Dinges, David F.	5	3	Dinges, David F.	445
3	Guastello, Stephen J.	5	4	Scholey, Andrew B.	389
3	Gunzelmann, Glenn	5	5	Lieberman, Harris R.	371
3	Tanaka, Masaaki	5			
3	Watanabe, Yasuyoshi	5			

### Analysis of references

**Figure 7:**
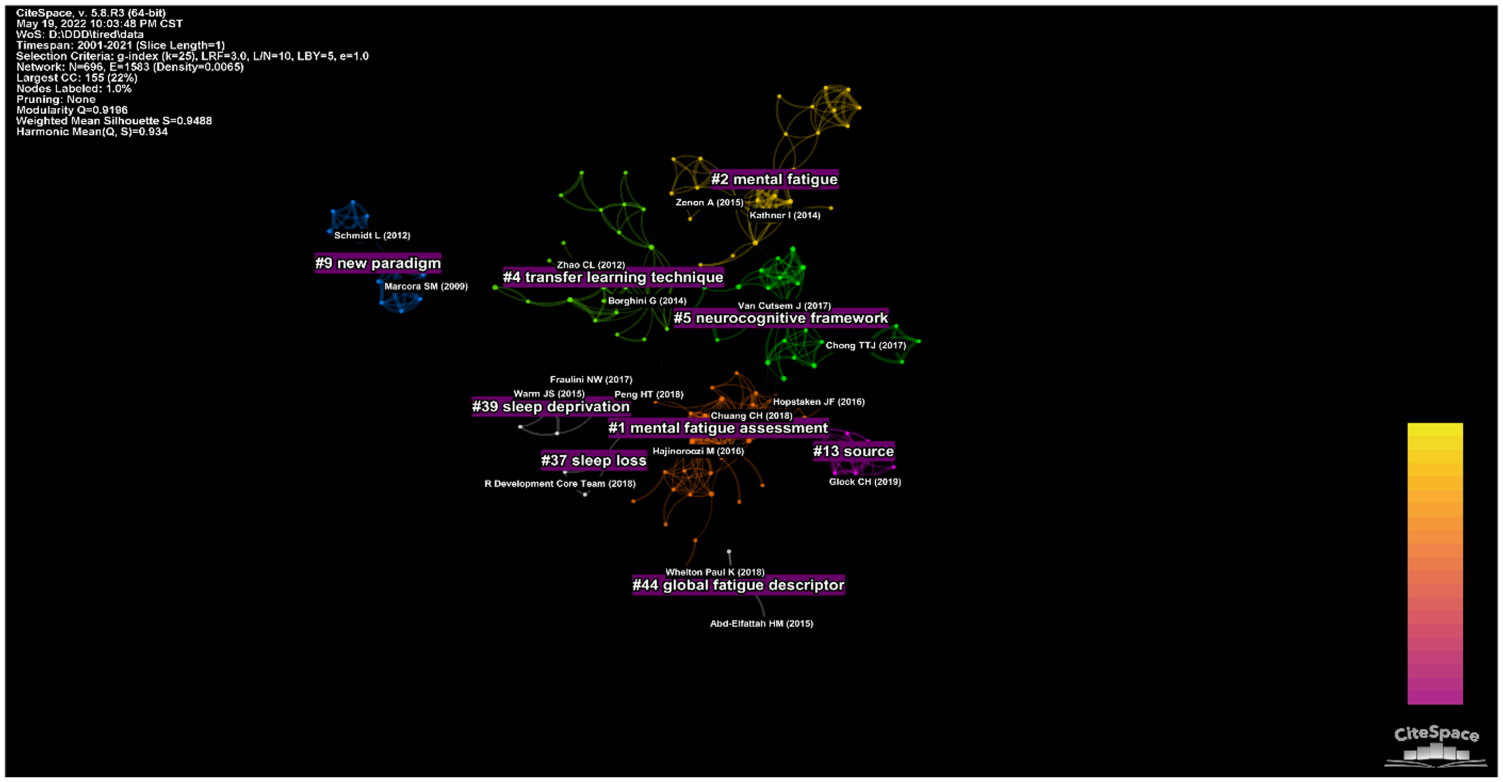
The co-citation cluster network visualization map of references based on CiteSpace.

In the past 20 years, publications in this field have been cited 29,373 times, with an average of 45 citations per publication. The top 5 cited references are presented in Table 6 with the highest number of references being《Development of NASA-TLX (task load index): results of empirical and theoretical research 1998》by Harts G. Moreover, the clustering of the reference based on co-citation of reference can excavate the contents of these documents. Figure 7 shows the co-citation clustering network of co-cited references. These keyword clusters include #1 (“mental fatigue assessment”), #2 (“mental fatigue”), #5 (“neurocognitive framework”), and #9 (“new paradigm”). Therefore, these keyword clusters are a research hot topic in this field.

**Table 6:** The top 5 co-citation of reference.

Rank	Reference	The first author (year)	Co-citation
1	Development of NASA-TLX (task load index): results of empirical and theoretical research	Harts G (1998)	42
2	Effects of mental fatigue on attention: an ERP study	Maarten A S Boksem (2005)	38
3	Mental fatigue: costs and benefits	Maarten A S Boksem (2008)	33
4	Mental fatigue impairs physical performance in humans	Samuele M Marcora (2008)	32
5	Compensatory control in the regulation of human performance under stress and high workload; a cognitive-energetical framework	G R Hockey (1997)	27

### Analysis of keywords

**Figure 8:**
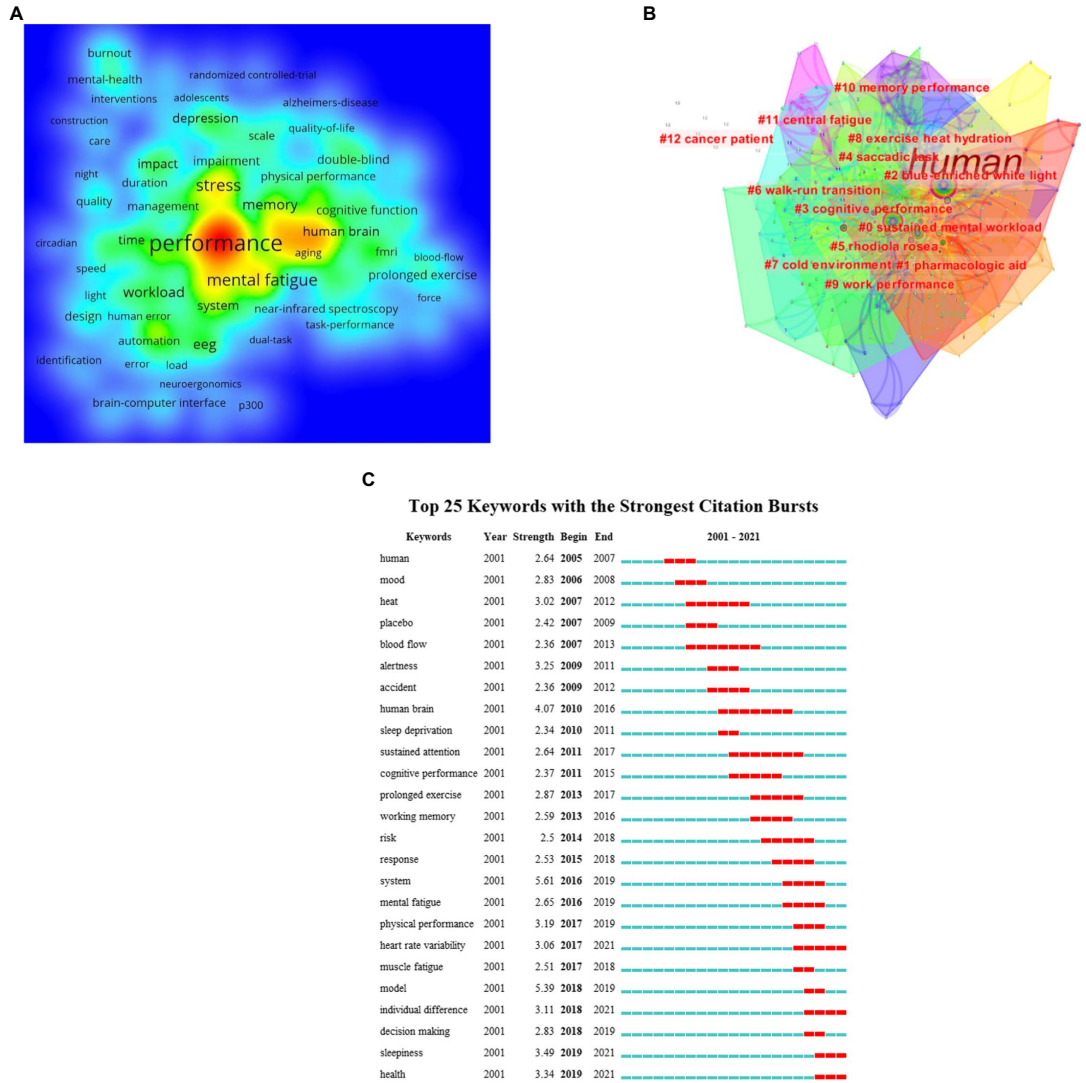
**(A)** The density visualization map of references based on VOSviewer; **(B)** The keywords clustering based on LIR method; **(C)** Top 25 keywords with the strongest citation bursts based on CiteSpace. The red horizontal stripes represent the years with the most frequent publications. The green horizontal stripes represent the years with the most infrequent publications.

Keywords co-occurrence clustering can reflect the hot topics in the research field and understand the discipline development patterns and new directions. It can identify the internal structure of an academic field and reveal the research frontier of the discipline. The top 10 co-occurrence times of keywords are listed in Table 7. Their keywords are performance, fatigue, human, stress, mental fatigue, and so on. Figures 8A,B show the density and clustering of keywords, which are sustained mental workload, sustained mental workload, saccadic task, *Rhodiola rosea*, and cognitive performance. The top 25 keywords with the strongest citation bursts are presented in Figure 8C. There are 25 burst words in all; muscle fatigue, model, individual difference, sleepiness, and health are the keywords with high burst intensity in recent 5 years. It can be seen that keywords with high burst intensity are mainly concentrated in the last 5 years.

**Table 7:** The top 10 co-occurrence times of keyword.

Rank	Keyword	Counts	Centrality	First publication year
1	Performance	177	0.23	2001
2	Fatigue	119	0.15	2004
3	Human	66	0.23	2001
4	Stress	54	0.09	2004
5	Mental fatigue	45	0.02	2010
6	Attention	40	0.07	2004
7	Mood	37	0.12	2001
7	EEG	37	0.05	2001
9	Brain	36	0.12	2004
10	Cognitive performance	33	0.18	2004
10	System	33	0.03	2014

## Discussion

### Analysis of category, country, institution, journals, and authors

From the bibliometric analysis on the effects of mental fatigue on athletic performance over the last 20 years, it was found that the number of publications has gradually increased in recent years, suggesting that more and more researchers are becoming involved in the study of the effects of mental fatigue.

Analyzed by category, the research is mainly applied in the social sciences and involves three disciplines: neuroscience, engineering, and psychology. In recent years, there has been a gradual increase in the number of publication and the application of disciplines in the field has become more widespread, such as industrial management, sports science, and computer science. With the development of these disciplines, the effects of mental fatigue on athletic performance in different occupational groups have received increasing attention.

Analyzed by country and institution, research activities in this field are unevenly distributed worldwide, with 9 of the top 10 countries being developed countries. The United States and its institutions are undoubtedly the leaders in this field. Other countries should be encouraged to improve the quality of their publications while maintaining the quantity of publications.

Analyzed by journals, Ergonomics and American Public Library of Science were cited more frequently, indicating the quality and influence of these two journals. In order to gain insight into the research related to the effects of mental fatigue on athletic performance, we can select publications in these two journals to provide references for research directions.

Analyzed by authors, the top 5 most prolific and most cited authors are from developed countries. Among them, Marcora, Samuele M. from the Endurance Research Centre, UK, was the first cited, and his “Mental fatigue impairs physical performance in humans (Marcora et al., 2009),” published in 2009, was also among the top five references. Marcora, Samuele M., whose research in this area focuses on the effects of mental fatigue on athletic performance, has pioneered research that confirms that the effects of mental fatigue on endurance performance are due to higher perceptions of effort rather than physiological factors such as cardiorespiratory and muscular energy. This finding provides strong evidence that “brain activity influences short-term endurance performance,” and the experimental procedures in the study provide a new paradigm for studying the relationship between fatigue and athletic performance, providing a direction for future research.

### The research hot topics of effects of mental fatigue on athletic performance

Keywords include “brain,” “EEG,” and “eye movement test.” The main characteristics of mental fatigue are “tiredness” and “lack of energy.” The subjective fatigue report is a widely used subjective report indicator to detect the occurrence of mental fatigue. In recent years, with the advancement of fatigue research, some objective physiological indicators have received increasing attention from scholars in this field. Electroencephalography (EEG), which can effectively monitor human brain states and behaviors, is an important objective indicator of mental fatigue, and can provide deeper insights into the fatigue phenomenon (Chen-Teng Lin and Wang, 2020). In a randomized cross over design, Krigolsen et al. (2021) found that β activity in prefrontal cortex in experimental condition compared to the control condition in a motor test of autonomic rhythm. Peng et al. (2019) showed that EEG spectra θ, α, and β were significantly correlated with subjective fatigue index, which can be used as physiological indicators to distinguish “alarm” from “fatigue.” In addition, Zenon et al. (2014) pointed out that pupil diameter increases with the difficulty of the task at hand during mental activities and that pupil size indicates the level of effort devoted to the task. Wachowicz et al. (2015) found that eye movement is a novel and effective method for studying the sensitivity of the attentional system to temporal factors, and eye-movement-based fatigue detection also provides an important objective physiological indicator of mental fatigue. Therefore, a reliable and objective assessment of mental fatigue can provide an understanding of the underlying neurological mechanisms and provide a valid assessment tool.

Keywords include “Rhodiola extract” “cancer patients” and “drug treatment.” How to improve mental fatigue is also an important topic in the field of fatigue-related research. Rhodiola (rose root) is an adaptogenic and potent herb that has received increasing attention in recent years for its ability to reduce the effects of fatigue on athletic performance (Josefin et al., 2009). However, a study (Thomas and Robert, 2006) conducted in Western Europe and North America found that Rhodiola has substantial antioxidant properties and that its effects on improving cognitive function and reducing mental fatigue remain to be further explored. In addition, animal studies (Ditte et al., 2012) have shown that neural activity increases extracellular adenosine concentrations and that cerebral adenosine impairs endurance performance (Mark et al., 2003). Recent studies have shown that caffeine (an adenosine antagonist) can counteract the negative effects of mental fatigue on endurance performance (Rafael et al., 2016). It has been demonstrated that sage monoterpene extracts (Kennedy et al., 2011), functional drinks (Jan et al., 2004), citric balsamic (Scholey et al., 2015), and resveratrol (Wightman et al., 2015) are effective in improving mood and cognitive performance and reducing self-reported “mental fatigue.” These pharmacological anti-fatigue measures are effective while other non-pharmacological anti-fatigue measures such as non-invasive neuroelectrical modulation can also be effective in improving fatigue. McIntire et al. (2021) assessed the effects of non-traumatic cervical transcutaneous vagus nerve stimulation (ctVNS) on cognitive performance mood and attention in sleep-deprived participants and their results suggest that ctVN may be an alternative therapy for the treatment of fatigue in humans. Therefore, pharmacological or non-pharmacological interventions for fatigued individuals to explore effective methods and measures to combat fatigue are also a hot research topic in this field.

### Effects of mental fatigue on athletic performance

Endurance performance, technical skills, and decision-making related to athletic performance are all research topics of interest in this field. In studies of endurance performance, Marcora et al. (2009) found no significant differences in subjects’ physiological responses to fatigue tests, either in the mental fatigue or control conditions, where mental fatigue impaired endurance performance, which was not caused by changes in the heart or in the response of muscle energy during mental fatigue, but by higher perception of effort. The effect of mental fatigue on endurance performance can be explained by the theory proposed by Pageaux et al. (2014) that prolonged exertion of mental activity may lead to the accumulation of adenosine in the anterior cingulate cortex, which, in turn, impairs endurance performance when higher effort is perceived during subsequent endurance exercise. To date, most studies have shown that mental fatigue has a negative impact on endurance performance, but have failed to identify the physiological changes that lead to impaired endurance performance. Therefore, we need to combine the multidisciplinary fields of psychology, physiology, and modeling to explain the negative effect of reasons, and future research could explore biological models of the effects of mental fatigue on endurance performance, as well as the physiological changes that contribute to impaired endurance performance, to better understand how physiological changes affect effort perception and thus endurance performance.

In studies of technical skills, Badin et al. (2016) observed a decrease in shot speed and accuracy in soccer players. Mansec et al. (2017) demonstrated a reduction in ball speed and accuracy during a table tennis performance test involving 45 forehand strokes. Veness et al. (2017) also found a reduction in cricket-technical skills performance in the presence of mental fatigue. Filipas et al. (2021) conducted a study on the skill performance of basketball players and found that mental fatigue would affect the performance of athletes in basketball tests, leading to the decline of their offensive skills. Therefore, it is clear that mental fatigue impairs the execution of sport-related specific technical skills associated with the upper and lower extremities. The negative effects of mental fatigue may extend to other sports technical skills such as rugby drop-goal or darts throwing. In future studies, there is a need to expand the research in other sports to enrich the research on the effects of mental fatigue on athletic performance and provide some suggestions to reduce the negative effects of mental fatigue on competition performance.

In studies of decision-making, decision-making includes the cognitive processes involved in the numerous behaviors observed on the court. It may be related to an athlete’s ability to accurately assess the trajectory of the ball in order to catch it, or to a team sport athlete’s ability to select the best passing option and place teammates in the best position to score (Memmert, 2010; Huijgen et al., 2015), and so is important to an athlete’s performance. Smith et al. (2016) showed that in a state of mental fatigue, soccer players had decreased response accuracy and reaction time and decreased decision-making ability in soccer simulation tests. Fortes et al. (2019) also found that mental fatigue would affect the decision-making performance of amateur volleyball players, leading to a decline in their offensive decision-making. Head et al. (2017) also showed that mental fatigue effects athletes’ decision to shoot accurate targets. These studies (soccer, volleyball, etc.) involved team sports in which decision-making plays an important role. Therefore, mental fatigue affects athletes’ decision-making in sporting competitions, and this effect applies to studies of team sports as well.

### Managerial implications of the review

The review has tried to enrich the research content of the effects of mental fatigue on athletic performance, so as to facilitate relevant researchers to have a more systematic understanding of the research trends in this field, and to provide more broad research ideas and directions in sports psychology, social science, cognitive neuroscience, and engineering. By analysis of category, country, institution, journals, and authors, stakeholders can quickly find the important information from the visual network provided above to understand the general overview of current research. By analysis of the research hot topics, it is suggested that medical personnel should adopt objective methods, such as electroencephalogram (Lin et al., 2021), eye movement (Zenon et al., 2014; Wachowicz et al., 2015), and other technologies to better identify fatigue, and make more accurate and convenient fatigue diagnosis for patients. In addition, some non-pharmacological interventions, such as non-invasive neuroelectrical modulation (McIntire et al., 2021), may be better than pharmacological interventions for human fatigue, helping healthcare professionals to take effective approaches to patients’ fatigue.

Furthermore, the effects of mental fatigue on athletic performance can be studied and discussed from the three aspects of endurance performance, technical skills, and sports-related decision-making, so as to help athletes better cope with mental fatigue in normal training and competition, and remind coaches to pay attention to the training of athletes’ mental fatigue, improve their tolerance to mental fatigue and help athletes achieve better results. Thus, researchers, athletes, or coaches can benefit from the visualized network provided. Furthermore, it shows that considerable research has been carried out in this area. The present bibliometric review and analysis have brought a new idea and aspect that earlier reviews might have ignored unknowingly.

## Conclusion remarks

This paper aims to shed light on the research of the effects of mental fatigue on athletic performance. Furthermore, it offers a groundwork for imminent research. The exploration tactic directed the assortment of 658 documents from 2001 to 2021 in the Web of Science repository. A systematic bibliometric analysis and visualization review is carried out to bridge the gap and identify the hot spots in research.

Analyzed by category, the research is mainly applied in the social sciences and involves three disciplines: neuroscience, engineering, and psychology. Analyzed by country and institution, research activities in this field are unevenly distributed worldwide, the United States and its institutions are undoubtedly the leaders in this field. Analyzed by journals, Ergonomics and American Public Library of Science were cited more frequently, indicating the quality and influence of these two journals. Analyzed by authors, the author Marcora, Samuele M. has the maximum number of citations in the field.

The “mental fatigue assessment,” “mental fatigue,” and “neurocognitive framework” emerge as keyword clusters with a contribution of a research hot topic in this field. The “performance” appears 177 times as an co-occurrence keyword followed by “fatigue” and “human.” The “muscle fatigue,” “model,” “individual difference,” sleepiness,” and “health” as burst words mainly concentrated in the last 5 years. Promoting the use of objective indicators to evaluate mental fatigue in EEG technology research, focusing on the effects of individual pharmacological or non-pharmacological interventions on fatigue, exploring effective methods and measures for fatigue are the main contents. Furthermore, focusing on the effects of mental fatigue on endurance performance, technical skills and sport-related decision-making are also the main research directions of this study.

In conclusion, the research on the effects of mental fatigue on athletic performance is still in a rapid development stage, with the United States being still in the lead. Promoting the use of objective indicators to evaluate mental fatigue in EEG technology research, focusing on the effects of individual pharmacological or non-pharmacological interventions on fatigue, exploring effective methods and measures for fatigue, and focusing on the effects of mental fatigue on endurance performance, technical skills, and sport-related decision-making are the main contents and research directions of this study. This research paper is a boon for a researcher willing to work in the field of mental fatigue. The network visualizations help to identify the research gaps termed Hotspots. Future research can be conducted in these areas, which allows the researcher to minimize the time spent determining the exact topic and field of the study.

## Limitations

First of all, we only collected data from the Web of Science® Core Collection when retrieving literature. So other representative literature may be overlooked and there are limitations in the analysis of research in this field. Secondly, the research on “ego depletion” and “self-control” is also relevant to the fields of mental fatigue. These two search terms were not searched for in this study, which may affect the interpretation of the main content. Third, bibliometrics is a quantitative analysis of publications, and high-cited articles do not necessarily equal high-quality articles or articles with high relevance to the target field. And due to the nature of CiteSpace and VOSviewer software, it is difficult to determine the details of complex partnerships.

## Author contributions

X-XC and Z-GJ designed the study and conceived the article. X-XC interpreted the data and wrote the article. X-XC, YW, and JX collected and analyzed the data. Z-GJ, L-YW, and H-BW revised the article. All authors contributed toward data analysis and drafting and critically revised and approved the final version of the paper.

## Funding

This study was partially supported by Shanghai University Students’ Physical Literacy Testing and Assessment Research (approval number: C2-2020016).

## Conflict of interest

The authors declare that the research was conducted in the absence of any commercial or financial relationships that could be construed as a potential conflict of interest.

## Publisher’s note

All claims expressed in this article are solely those of the authors and do not necessarily represent those of their affiliated organizations, or those of the publisher, the editors and the reviewers. Any product that may be evaluated in this article, or claim that may be made by its manufacturer, is not guaranteed or endorsed by the publisher.

## Author disclaimer

The views expressed in this review come from the authors alone.
