# Supplementary material for: Bibliometric analysis of the effects of mental fatigue on athletic performance from 2001 to 2021
Source: Front Psychol. 2023 Jan 9;13:1019417. doi: 10.3389/fpsyg.2022.1019417 (PMC9869051; doi:10.3389/fpsyg.2022.1019417)
Supplement: Supplementary file 1 [file Table_1.DOCX]

***Supplementary Material***

Contents of supplementary appendix

[Appendix 1 2](#_Toc5642)

[Topic search queries used for data collection 2](#_Toc14404)

[Appendix 2 4](#_Toc5780)

[Information on 658 articles included in the analysis 4](#_Toc22747)

##

## Appendix 1

## Topic search queries used for data collection

| **Web of Science Core Collection^®^ data base** | | |
| --- | --- | --- |
| # 1 | 23,154 | TS= ("mental fatigue") OR TS = ("mental exertion") OR TS = ("mental exertion") OR TS = ("cognitive exertion") OR TS = ("mental exhaustion") OR TS = ("mental tiredness")  Index =SCI-EXPANDED, SSCI, A&HCI, CPCI-S, CPCI-SSH, BKCI-S, BKCI-SSH, ESCI, CCR-EXPANDED, and IC |
| # 2 | 9,522,956 | TS= ("athletic performance") ORTS=( "technical skill*") OR TS=( "skill*" ) OR TS=("technique" ) OR TS=("decision making") OR TS=("performance")  Index =SCI-EXPANDED, SSCI, A&HCI, CPCI-S, CPCI-SSH, BKCI-S, BKCI-SSH, ESCI, CCR-EXPANDED, and IC |
| #3 | 4,475,549 | TS= ("humans")  Index =SCI-EXPANDED, SSCI, A&HCI, CPCI-S, CPCI-SSH, BKCI-S, BKCI-SSH, ESCI, CCR-EXPANDED, and IC |
| # 4 | 1,004 | #1 AND #2 AND #3  Index = SCI-EXPANDED, SSCI, A&HCI, CPCI-S, CPCI-SSH, BKCI-S, BKCI-SSH, ESCI, CCR-EXPANDED, and IC |
| # 5 | 775 | # 4 AND YS=(2001-2021) AND LANGUAGES: (English)  Index =SCI-EXPANDED, SSCI, A&HCI, CPCI-S, CPCI-SSH, BKCI-S, BKCI-SSH, ESCI, CCR-EXPANDED, and IC |
| # 6 | 658 | # 5 AND DOCUMENT TYPES: (Article）  Index =SCI-EXPANDED, SSCI, A&HCI, CPCI-S, CPCI-SSH, BKCI-S, BKCI-SSH, ESCI, CCR-EXPANDED, and IC |

##

## Appendix 2

## Information on 658 articles included in the analysis

FN Clarivate Analytics Web of Science

VR 1.0

PT J

AU Pageaux, B

Marcora, SM

Lepers, R

AF Pageaux, Benjamin

Marcora, Samuele M.

Lepers, Romuald

TI Prolonged Mental Exertion Does Not Alter Neuromuscular Function of the

Knee Extensors

SO MEDICINE AND SCIENCE IN SPORTS AND EXERCISE

LA English

DT Article

DE PERCEPTION OF EFFORT; MUSCLE ACTIVATION; MENTAL FATIGUE; PERIPHERAL

FATIGUE; CENTRAL FATIGUE; ENDURANCE PERFORMANCE

ID MAXIMAL VOLUNTARY CONTRACTIONS; ANTERIOR CINGULATE CORTEX; PLASMA

AMINO-ACIDS; MUSCLE FATIGUE; PERIPHERAL FATIGUE; BRAIN ACTIVATION;

CYCLING EXERCISE; PERFORMANCE; HUMANS; FMRI

AB Purpose: The aim of this study was to test the hypotheses that prolonged mental exertion (i) reduces maximal muscle activation and (ii) increases the extent of central fatigue induced by subsequent endurance exercise. Methods: The neuromuscular function of the knee extensor muscles was assessed in 10 male subjects in two different conditions: (i) before and after prolonged mental exertion leading to mental fatigue and (ii) before and after an easy cognitive task (control). Both cognitive tasks lasted 90 min and were followed by submaximal isometric knee extensor exercise until exhaustion (endurance task), and a third assessment of neuromuscular function. Results: Time to exhaustion was 13% +/- 4% shorter in the mental fatigue condition (230 +/- 22 s) compared with the control condition (266 +/- 26 s) (P < 0.01). Prolonged mental exertion did not have any significant effect on maximal voluntary contraction torque, voluntary activation level, and peripheral parameters of neuromuscular function. A similar significant decrease in maximal voluntary contraction torque (mental fatigue condition: -26.7% +/- 5.7%; control condition: -27.6% +/- 3.3%, P < 0.001), voluntary activation level (mental fatigue: - 10.6% +/- 4.3%; control condition: - 11.2% +/- 5.2%, P < 0.05), and peripheral parameters of neuromuscular function occurred in both conditions after the endurance task. However, mentally fatigued subjects rated perceived exertion significantly higher during the endurance task compared with the control condition (P < 0.05). Conclusions: These findings provide the first experimental evidence that prolonged mental exertion (i) does not reduce maximal muscle activation and (ii) does not increase the extent of central fatigue induced by subsequent endurance exercise. The negative effect of mental fatigue on endurance performance seems to be mediated by the higher perception of effort rather than impaired neuromuscular function.

C1 [Pageaux, Benjamin; Marcora, Samuele M.] Univ Kent Medway, Sch Sport & Exercise Sci, Endurance Res Grp, Chatham, Kent, England.

[Pageaux, Benjamin; Lepers, Romuald] Univ Bourgogne, Lab INSERM U1093, Fac Sci Sports, UFR STAPS, F-21078 Dijon, France.

RP Lepers, R (corresponding author), Univ Bourgogne, Lab INSERM U1093, Fac Sci Sports, BP 27877, F-21078 Dijon, France.

EM romuald.lepers@u-bourgogne.fr

RI Lepers, Romuald/T-8782-2019; Marcora, Samuele/W-4284-2019; Pageaux,

Benjamin/G-9253-2016

OI Marcora, Samuele/0000-0002-1570-7936; Pageaux,

Benjamin/0000-0001-9302-5183; Lepers, Romuald/0000-0002-3870-4017

CR Amann M, 2011, MED SCI SPORT EXER, V43, P2039, DOI 10.1249/MSS.0b013e31821f59ab

Baumeister RF, 2007, SOC PERSONAL PSYCHOL, V1, P115, DOI 10.1111/j.1751-9004.2007.00001.x

Boksem MAS, 2008, BRAIN RES REV, V59, P125, DOI 10.1016/j.brainresrev.2008.07.001

Borg G., 1998, BORGS PERCEIVED EXER, P104

Bray SR, 2008, PSYCHOPHYSIOLOGY, V45, P337, DOI 10.1111/j.1469-8986.2007.00625.x

Bray SR, 2012, BIOL PSYCHOL, V89, P195, DOI 10.1016/j.biopsycho.2011.10.008

BREHM JW, 1989, ANNU REV PSYCHOL, V40, P109, DOI 10.1146/annurev.ps.40.020189.000545

Campbell S., 2001, P HUMAN FACTORS ERGO, V45, P906, DOI DOI 10.1177/154193120104501302

Carter CS, 1998, SCIENCE, V280, P747, DOI 10.1126/science.280.5364.747

Davis JM, 2003, AM J PHYSIOL-REG I, V284, pR399, DOI 10.1152/ajpregu.00386.2002

de Morree HM, 2012, PSYCHOPHYSIOLOGY, V49, P1242, DOI 10.1111/j.1469-8986.2012.01399.x

Di Giulio C, 2006, ADV PHYSIOL EDUC, V30, P51, DOI 10.1152/advan.00041.2005

ENOKA RM, 1995, J ELECTROMYOGR KINES, V5, P141, DOI 10.1016/1050-6411(95)00010-W

Froyd C, 2013, J PHYSIOL-LONDON, V591, P1339, DOI 10.1113/jphysiol.2012.245316

Gailliot MT, 2008, PERSPECT PSYCHOL SCI, V3, P245, DOI 10.1111/j.1745-6924.2008.00077.x

Gandevia SC, 2001, PHYSIOL REV, V81, P1725, DOI 10.1152/physrev.2001.81.4.1725

Liu JZ, 2003, J NEUROPHYSIOL, V90, P300, DOI 10.1152/jn.00821.2002

Lorist MM, 2003, BRAIN COGNITION, V53, P82, DOI 10.1016/S0278-2626(03)00206-9

Marcora SM, 2008, AM J PHYSIOL-REG I, V294, pR874, DOI 10.1152/ajpregu.00678.2007

Marcora SM, 2009, J APPL PHYSIOL, V106, P857, DOI 10.1152/japplphysiol.91324.2008

Marcora SM, 2010, EUR J APPL PHYSIOL, V109, P763, DOI 10.1007/s00421-010-1418-6

Marcora SM, 2009, ENCY PERCEPTION

Matsui T, 2011, J PHYSIOL-LONDON, V589, P3383, DOI [10.1113/jphysiol.2010.203570, 10.1113/jphysiol.2011.203570]

Mizuno K, 2007, J NEURAL TRANSM, V114, P555, DOI 10.1007/s00702-006-0608-1

Mosso A., 1906, FATIGUE, P334

NEWSHOLME EA, 1992, BRIT MED BULL, V48, P477, DOI 10.1093/oxfordjournals.bmb.a072558

Nozaki S, 2009, NUTRITION, V25, P51, DOI 10.1016/j.nut.2008.07.010

Paus T, 2001, NAT REV NEUROSCI, V2, P417, DOI 10.1038/35077500

Place N, 2005, J APPL PHYSIOL, V98, P429, DOI 10.1152/japplphysiol.00664.2004

Place N, 2004, MED SCI SPORT EXER, V36, P1347, DOI 10.1249/01.MSS.0000135786.22996.77

Post M, 2009, HUM BRAIN MAPP, V30, P1014, DOI 10.1002/hbm.20562

Richter M, 2008, PSYCHOPHYSIOLOGY, V45, P869, DOI 10.1111/j.1469-8986.2008.00688.x

Shield A, 2004, SPORTS MED, V34, P253, DOI 10.2165/00007256-200434040-00005

Taylor JL, 2008, J APPL PHYSIOL, V104, P542, DOI 10.1152/japplphysiol.01053.2007

Taylor JL, 2000, J APPL PHYSIOL, V89, P305, DOI 10.1152/jappl.2000.89.1.305

Terry PC, 2003, PSYCHOL SPORT EXERC, V4, P125, DOI 10.1016/S1469-0292(01)00035-8

van der Linden D, 2003, ACTA PSYCHOL, V113, P45, DOI 10.1016/S0001-6918(02)00150-6

van der Linden D, 2006, INT J PSYCHOPHYSIOL, V62, P168, DOI 10.1016/j.ijpsycho.2006.04.001

van Duinen H, 2008, HUM BRAIN MAPP, V29, P281, DOI 10.1002/hbm.20388

van Duinen H, 2007, NEUROIMAGE, V35, P1438, DOI 10.1016/j.neuroimage.2007.02.008

Williamson JW, 2001, J APPL PHYSIOL, V90, P1392, DOI 10.1152/jappl.2001.90.4.1392

NR 41

TC 124

Z9 125

U1 0

U2 19

PU LIPPINCOTT WILLIAMS & WILKINS

PI PHILADELPHIA

PA 530 WALNUT ST, PHILADELPHIA, PA 19106-3621 USA

SN 0195-9131

EI 1530-0315

J9 MED SCI SPORT EXER

JI Med. Sci. Sports Exerc.

PD DEC

PY 2013

VL 45

IS 12

BP 2254

EP 2264

DI 10.1249/MSS.0b013e31829b504a

PG 11

WC Sport Sciences

WE Science Citation Index Expanded (SCI-EXPANDED); Social Science Citation Index (SSCI)

SC Sport Sciences

GA 300LN

UT WOS:000330465500006

PM 23698244

OA Green Submitted

DA 2022-05-04

ER

PT J

AU Marcora, SM

Staiano, W

Manning, V

AF Marcora, Samuele M.

Staiano, Walter

Manning, Victoria

TI Mental fatigue impairs physical performance in humans

SO JOURNAL OF APPLIED PHYSIOLOGY

LA English

DT Article

DE exercise performance; endurance; perceived exertion; motivation

ID ANTERIOR CINGULATE CORTEX; EXERCISE PERFORMANCE; MAXIMAL EXERCISE;

TASK-DIFFICULTY; MUSCLE FATIGUE; CARDIOVASCULAR-RESPONSE; COGNITIVE

PERFORMANCE; PROLONGED EXERCISE; NUCLEUS-ACCUMBENS; BRAIN ACTIVATION

AB Marcora SM, Staiano W, Manning V. Mental fatigue impairs physical performance in humans. J Appl Physiol 106: 857-864, 2009. First published January 8, 2009; doi:10.1152/japplphysiol. 91324.2008.-Mental fatigue is a psychobiological state caused by prolonged periods of demanding cognitive activity. Although the impact of mental fatigue on cognitive and skilled performance is well known, its effect on physical performance has not been thoroughly investigated. In this randomized crossover study, 16 subjects cycled to exhaustion at 80% of their peak power output after 90 min of a demanding cognitive task (mental fatigue) or 90 min of watching emotionally neutral documentaries (control). After experimental treatment, a mood questionnaire revealed a state of mental fatigue (P = 0.005) that significantly reduced time to exhaustion (640 +/- 316 s) compared with the control condition (754 +/- 339 s) (P = 0.003). This negative effect was not mediated by cardiorespiratory and musculoenergetic factors as physiological responses to intense exercise remained largely unaffected. Self-reported success and intrinsic motivation related to the physical task were also unaffected by prior cognitive activity. However, mentally fatigued subjects rated perception of effort during exercise to be significantly higher compared with the control condition (P = 0.007). As ratings of perceived exertion increased similarly over time in both conditions (P = 0.001), mentally fatigued subjects reached their maximal level of perceived exertion and disengaged from the physical task earlier than in the control condition. In conclusion, our study provides experimental evidence that mental fatigue limits exercise tolerance in humans through higher perception of effort rather than cardiorespiratory and musculoenergetic mechanisms. Future research in this area should investigate the common neurocognitive resources shared by physical and mental activity.

C1 [Marcora, Samuele M.; Staiano, Walter; Manning, Victoria] Bangor Univ, Sch Sport Hlth & Exercise Sci, Bangor LL57 2PZ, Gwynedd, Wales.

RP Marcora, SM (corresponding author), Bangor Univ, Sch Sport Hlth & Exercise Sci, George Bldg,Holyhead Rd, Bangor LL57 2PZ, Gwynedd, Wales.

EM s.m.marcora@bangor.ac.uk

RI Staiano, Walter/AAB-3246-2019; Marcora, Samuele M/C-5614-2009; Marcora,

Samuele/W-4284-2019

OI Staiano, Walter/0000-0002-1269-0016; Marcora,

Samuele/0000-0002-1570-7936

CR Amann M, 2008, MED SCI SPORT EXER, V40, P574, DOI [10.1249/MSS.0b013e31815e728f, 10.1249/mss.0b013e31815e728f]

Amann M, 2008, J PHYSIOL-LONDON, V586, P161, DOI 10.1113/jphysiol.2007.141838

Andreacci JL, 2002, J SPORT SCI, V20, P345, DOI 10.1080/026404102753576125

Barch DM, 1997, NEUROPSYCHOLOGIA, V35, P1373, DOI 10.1016/S0028-3932(97)00072-9

BARTH JL, 1976, J MOTOR BEHAV, V8, P189, DOI 10.1080/00222895.1976.10735071

Bassett DR, 2000, MED SCI SPORT EXER, V32, P70, DOI 10.1097/00005768-200001000-00012

Boksem MAS, 2008, BRAIN RES REV, V59, P125, DOI 10.1016/j.brainresrev.2008.07.001

Boksem MAS, 2006, BIOL PSYCHOL, V72, P123, DOI 10.1016/j.biopsycho.2005.08.007

Boksem MAS, 2005, COGNITIVE BRAIN RES, V25, P107, DOI 10.1016/j.cogbrainres.2005.04.011

Borg G., 1998, BORGS PERCEIVED EXER

Burnley M, 2007, EUR J SPORT SCI, V7, P63, DOI 10.1080/17461390701456148

Campbell S., 2001, P HUMAN FACTORS ERGO, V45, P906, DOI DOI 10.1177/154193120104501302

Carter CS, 1998, SCIENCE, V280, P747, DOI 10.1126/science.280.5364.747

Charloux A, 2000, EUR J APPL PHYSIOL, V82, P313, DOI 10.1007/s004210000226

Cook DB, 2007, NEUROIMAGE, V36, P108, DOI 10.1016/j.neuroimage.2007.02.033

Coyle E F, 2001, Exerc Sport Sci Rev, V29, P88, DOI 10.1097/00003677-200104000-00009

Critchley HD, 2003, BRAIN, V126, P2139, DOI 10.1093/brain/awg216

Dalsgaard MK, 2006, J CEREBR BLOOD F MET, V26, P731, DOI 10.1038/sj.jcbfm.9600256

Davis JM, 1997, MED SCI SPORT EXER, V29, P45, DOI 10.1097/00005768-199701000-00008

Fairclough SH, 2004, BIOL PSYCHOL, V66, P177, DOI 10.1016/j.biopsycho.2003.10.001

Gailliot MT, 2008, PERSPECT PSYCHOL SCI, V3, P245, DOI 10.1111/j.1745-6924.2008.00077.x

Gandevia SC, 2001, PHYSIOL REV, V81, P1725, DOI 10.1152/physrev.2001.81.4.1725

Gibson AS, 2004, BRIT J SPORT MED, V38, P797, DOI 10.1136/bjsm.2003.009852

GIBSON H, 1993, J NEUROL NEUROSUR PS, V56, P993, DOI 10.1136/jnnp.56.9.993

Gonzalez-Alonso J, 2004, J PHYSIOL-LONDON, V557, P331, DOI 10.1113/jphysiol.2004.060574

Gonzalez-Alonso J, 2003, CIRCULATION, V107, P824, DOI 10.1161/01.CIR.0000049746.29175.3F

Goode JH, 2003, J SAFETY RES, V34, P309, DOI 10.1016/S0022-4375(03)00033-1

Hallett M, 2007, CLIN NEUROPHYSIOL, V118, P1179, DOI 10.1016/j.clinph.2007.03.019

Hargreaves M, 2008, J APPL PHYSIOL, V104, P1541, DOI 10.1152/japplphysiol.00088.2008

HOLM S, 1979, SCAND J STAT, V6, P65

HOOKS MS, 1992, PHARMACOL BIOCHEM BE, V42, P765, DOI 10.1016/0091-3057(92)90027-D

Hsu AR, 2006, J APPL PHYSIOL, V100, P2031, DOI 10.1152/japplphysiol.00806.2005

Joyner MJ, 2008, J PHYSIOL-LONDON, V586, P35, DOI 10.1113/jphysiol.2007.143834

KARLSSON J, 1970, J APPL PHYSIOL, V29, P598, DOI 10.1152/jappl.1970.29.5.598

Kayser B, 2003, EUR J APPL PHYSIOL, V90, P411, DOI 10.1007/s00421-003-0902-7

Kohlisch O, 1996, ERGONOMICS, V39, P213, DOI 10.1080/00140139608964452

KUIPERS H, 1985, INT J SPORTS MED, V6, P197, DOI 10.1055/s-2008-1025839

Lal SKL, 2001, BIOL PSYCHOL, V55, P173, DOI 10.1016/S0301-0511(00)00085-5

Larsen TS, 2008, J PHYSIOL-LONDON, V586, P2807, DOI 10.1113/jphysiol.2008.151449

Lorist MM, 2000, PSYCHOPHYSIOLOGY, V37, P614, DOI 10.1111/1469-8986.3750614

Lorist MM, 2005, COGNITIVE BRAIN RES, V24, P199, DOI 10.1016/j.cogbrainres.2005.01.018

Lorist MM, 2002, J PHYSIOL-LONDON, V545, P313, DOI 10.1113/jphysiol.2002.027938

Lorist MM, 2008, BRAIN RES, V1232, P113, DOI 10.1016/j.brainres.2008.07.053

Marcora S, 2009, J APPL PHYSIOL, V106, P2060, DOI 10.1152/japplphysiol.90378.2008

Marcora SM, 2008, EUR J APPL PHYSIOL, V104, P929, DOI 10.1007/s00421-008-0818-3

Marcora SM, 2008, AM J PHYSIOL-REG I, V294, pR874, DOI 10.1152/ajpregu.00678.2007

MARTIN BJ, 1981, EUR J APPL PHYSIOL O, V47, P345, DOI 10.1007/BF02332962

Meeusen R, 2006, SPORTS MED, V36, P881, DOI 10.2165/00007256-200636100-00006

Mortensen SP, 2005, J PHYSIOL-LONDON, V566, P273, DOI 10.1113/jphysiol.2005.086025

Mortensen SP, 2008, J PHYSIOL-LONDON, V586, P2621, DOI 10.1113/jphysiol.2007.149401

Mosso A., 1891, FATICA

Noble B. J., 1996, PERCEIVED EXERTION

Nybo L, 2004, PROG NEUROBIOL, V72, P223, DOI 10.1016/j.pneurobio.2004.03.005

Nybo L, 2008, J APPL PHYSIOL, V104, P871, DOI 10.1152/japplphysiol.00910.2007

Paus T, 2001, NAT REV NEUROSCI, V2, P417, DOI 10.1038/35077500

Paus T, 1998, NEUROREPORT, V9, pR37, DOI 10.1097/00001756-199806220-00001

Richter M, 2008, PSYCHOPHYSIOLOGY, V45, P869, DOI 10.1111/j.1469-8986.2008.00688.x

Rudebeck PH, 2006, NAT NEUROSCI, V9, P1161, DOI 10.1038/nn1756

Salamone JD, 2007, PSYCHOPHARMACOLOGY, V191, P389, DOI 10.1007/s00213-006-0623-9

SENN S, 2002, CROSS OVER TRAILS CL

Sgherza AL, 2002, J APPL PHYSIOL, V93, P2023, DOI 10.1152/japplphysiol.00521.2002

Silvestrini N, 2007, PSYCHOPHYSIOLOGY, V44, P650, DOI 10.1111/j.1469-8986.2007.00532.x

Smith SA, 2006, EXP PHYSIOL, V91, P89, DOI 10.1113/expphysiol.2005.032367

ten Caat M, 2008, J NEUROSCI METH, V171, P271, DOI 10.1016/j.jneumeth.2008.03.022

Terry PC, 2003, PSYCHOL SPORT EXERC, V4, P125, DOI 10.1016/S1469-0292(01)00035-8

van der Linden D, 2003, ACTA PSYCHOL, V113, P45, DOI 10.1016/S0001-6918(02)00150-6

van der Linden D, 2006, INT J PSYCHOPHYSIOL, V62, P168, DOI 10.1016/j.ijpsycho.2006.04.001

van der Linden D, 2006, PSYCHOL RES-PSYCH FO, V70, P395, DOI 10.1007/s00426-005-0228-7

Walton ME, 2006, NEURAL NETWORKS, V19, P1302, DOI 10.1016/j.neunet.2006.03.005

Walton ME, 2003, J NEUROSCI, V23, P6475

Ward SA, 2007, EXP PHYSIOL, V92, P321, DOI 10.1113/expphysiol.2006.034389

Williamson JW, 2001, J APPL PHYSIOL, V90, P1392, DOI 10.1152/jappl.2001.90.4.1392

Williamson JW, 2002, J APPL PHYSIOL, V92, P1317, DOI 10.1152/japplphysiol.00939.2001

Williamson JW, 2006, EXP PHYSIOL, V91, P51, DOI 10.1113/expphysiol.2005.032037

Wright RA, 2008, INT J PSYCHOPHYSIOL, V69, P127, DOI 10.1016/j.ijpsycho.2008.04.002

Wright RA, 2007, MOTIV EMOTION, V31, P219, DOI 10.1007/s11031-007-9066-9

Wright RA, 2008, SOC PERSONAL PSYCHOL, V2, P682, DOI 10.1111/j.1751-9004.2008.00093.x

Zijdewind I, 2006, CLIN NEUROPHYSIOL, V117, P660, DOI 10.1016/j.clinph.2005.11.016

NR 78

TC 629

Z9 646

U1 15

U2 227

PU AMER PHYSIOLOGICAL SOC

PI BETHESDA

PA 9650 ROCKVILLE PIKE, BETHESDA, MD 20814 USA

SN 8750-7587

EI 1522-1601

J9 J APPL PHYSIOL

JI J. Appl. Physiol.

PD MAR

PY 2009

VL 106

IS 3

BP 857

EP 864

DI 10.1152/japplphysiol.91324.2008

PG 8

WC Physiology; Sport Sciences

WE Science Citation Index Expanded (SCI-EXPANDED); Social Science Citation Index (SSCI)

SC Physiology; Sport Sciences

GA 412IY

UT WOS:000263719700016

PM 19131473

OA Green Published

DA 2022-05-04

ER

PT J

AU Muller, T

Apps, MAJ

AF Muller, Tanja

Apps, Matthew A. J.

TI Motivational fatigue: A neurocognitive framework for the impact of

effortful exertion on subsequent motivation

SO NEUROPSYCHOLOGIA

LA English

DT Article

DE Motivation; Effort; Fatigue; Cognitive; Physical; Anterior cingulate

ID MEDIAL FRONTAL-CORTEX; TIME-ON-TASK; HUMAN CINGULATE CORTEX; OLD-WORLD

MONKEY; ANTERIOR CINGULATE; DECISION-MAKING; MENTAL FATIGUE; PREFRONTAL

CORTEX; INDIVIDUAL-DIFFERENCES; INTEGRATIVE THEORY

AB Fatigue - a feeling of exhaustion arising from exertion - is a significant barrier to successful behaviour and one of the most prominent symptoms in primary care. During extended behaviours, fatigue increases over time, leading to decrements in performance in both cognitively and physically demanding tasks. However, to date, theoretical accounts of fatigue have not fully characterised the neuroanatomical basis of cognitive and physical fatigue nor placed results within broader discussions of the functional properties of the systems implicated. Here, we review recent neurophysiological and neuroimaging research that has begun to identify the neural mechanisms underlying changes in behaviour occurring due to fatigue. Strikingly, this research has implicated systems in the brain, including the dorsal anterior cingulate cortex (dACC), anterior insula, and lateral prefrontal cortex, that in separate lines of research have been linked to motivating the exertion of effort, to persisting towards goals and to processing one's internal states. We put forward a neurocognitive framework for fatigue and its impact on motivation. Levels of fatigue arising from effortful behaviours impact on processing in systems that weigh up the costs and benefits of exerting effort. As a result, as levels of fatigue rise, the value of exerting effort into a task declines, leading to reductions in performance. This account provides a new framework for understanding the effects of fatigue during cognitively and physically demanding tasks as well as for understanding motivational impairments in health and disease.

C1 [Muller, Tanja; Apps, Matthew A. J.] Univ Oxford, Dept Expt Psychol, Oxford, England.

[Muller, Tanja; Apps, Matthew A. J.] Univ Oxford, Nuffield Dept Clin Neurosci, Wellcome Ctr Integrat Neuroimaging WIN, Oxford, England.

RP Muller, T; Apps, MAJ (corresponding author), Radcliffe Observ Quarter, Dept Expt Psychol, New Radcliffe House,Woodstock Rd, Oxford OX2 6GG, England.

EM tanja.mueller@psy.ox.ac.uk; matthew.apps@psy.ox.ac.uk

RI Apps, Matthew/ABA-6387-2021

OI Apps, Matthew/0000-0001-5793-2202; Muller, Tanja/0000-0003-0659-7883

FU BBSRC Anniversary Future Leader FellowshipUK Research & Innovation

(UKRI)Biotechnology and Biological Sciences Research Council (BBSRC)

[BB/M013596/1]; Wellcome Institutional Strategic Support Grant; German

Academic Exchange Service (DAAD)Deutscher Akademischer Austausch Dienst

(DAAD); BBSRCUK Research & Innovation (UKRI)Biotechnology and Biological

Sciences Research Council (BBSRC) [BB/R010668/1] Funding Source: UKRI

FX MAJA was funded by a BBSRC Anniversary Future Leader Fellowship

(BB/M013596/1) and a Wellcome Institutional Strategic Support Grant. TM

was funded by a scholarship from the German Academic Exchange Service

(DAAD).

CR Ainley V, 2016, PHILOS T R SOC B, V371, DOI 10.1098/rstb.2016.0003

Alexander WH, 2018, J COGNITIVE NEUROSCI, V30, P1061, DOI 10.1162/jocn_a_01154

Alexander WH, 2017, J COGNITIVE NEUROSCI, V29, P1674, DOI 10.1162/jocn_a_01138

Amiez C, 2013, J NEUROSCI, V33, P2217, DOI 10.1523/JNEUROSCI.2779-12.2013

Ang YS, 2017, PLOS ONE, V12, DOI 10.1371/journal.pone.0169938

Apps MAJ, 2015, SCI REP-UK, V5, DOI 10.1038/srep16880

Apps MAJ, 2014, J NEUROSCI, V34, P6190, DOI 10.1523/JNEUROSCI.2701-13.2014

Asplund CL, 2013, NEUROIMAGE, V82, P326, DOI 10.1016/j.neuroimage.2013.05.119

Aston-Jones G, 2005, ANNU REV NEUROSCI, V28, P403, DOI 10.1146/annurev.neuro.28.061604.135709

Avanzino L, 2011, NEUROSCIENCE, V174, P84, DOI 10.1016/j.neuroscience.2010.11.008

Balsters JH, 2016, NEUROIMAGE-CLIN, V11, P494, DOI 10.1016/j.nicl.2016.03.016

Blain B, 2016, P NATL ACAD SCI USA, V113, P6967, DOI 10.1073/pnas.1520527113

Boksem MAS, 2008, BRAIN RES REV, V59, P125, DOI 10.1016/j.brainresrev.2008.07.001

Boksem MAS, 2006, BIOL PSYCHOL, V72, P123, DOI 10.1016/j.biopsycho.2005.08.007

Boksem MAS, 2005, COGNITIVE BRAIN RES, V25, P107, DOI 10.1016/j.cogbrainres.2005.04.011

Bonnelle V, 2016, CEREB CORTEX, V26, P807, DOI 10.1093/cercor/bhv247

Bonnelle V, 2015, J PHYSIOL-PARIS, V109, P16, DOI 10.1016/j.jphysparis.2014.04.002

Borghini G, 2014, NEUROSCI BIOBEHAV R, V44, P58, DOI 10.1016/j.neubiorev.2012.10.003

Botvinick M, 2015, ANNU REV PSYCHOL, V66, P83, DOI 10.1146/annurev-psych-010814-015044

Botvinick MM, 2009, COGN AFFECT BEHAV NE, V9, P16, DOI 10.3758/CABN.9.1.16

Carroll TJ, 2017, J APPL PHYSIOL, V122, P1068, DOI 10.1152/japplphysiol.00775.2016

Chaudhuri A, 2004, LANCET, V363, P978, DOI 10.1016/S0140-6736(04)15794-2

Chong TTJ, 2017, PLOS BIOL, V15, DOI 10.1371/journal.pbio.1002598

Chong TTJ, 2015, CORTEX, V69, P40, DOI 10.1016/j.cortex.2015.04.003

Cools R, 2015, CURR OPIN BEHAV SCI, V4, P152, DOI 10.1016/j.cobeha.2015.05.007

Craig AD, 2009, NAT REV NEUROSCI, V10, P59, DOI 10.1038/nrn2555

Craig AD, 2003, CURR OPIN NEUROBIOL, V13, P500, DOI 10.1016/S0959-4388(03)00090-4

Critchley HD, 2004, NAT NEUROSCI, V7, P189, DOI 10.1038/nn1176

Critchley HD, 2000, J PHYSIOL-LONDON, V523, P259, DOI 10.1111/j.1469-7793.2000.t01-1-00259.x

Critchley HD, 2005, NEUROIMAGE, V27, P885, DOI 10.1016/j.neuroimage.2005.05.047

Critchley HD, 2003, BRAIN, V126, P2139, DOI 10.1093/brain/awg216

Croxson PL, 2009, J NEUROSCI, V29, P4531, DOI 10.1523/JNEUROSCI.4515-08.2009

Cullen W, 2002, IRISH J MED SCI, V171, P10, DOI 10.1007/BF03168931

Danielmeier C, 2015, CURR BIOL, V25, P1461, DOI 10.1016/j.cub.2015.04.022

de Morree HM, 2014, J APPL PHYSIOL, V117, P1514, DOI 10.1152/japplphysiol.00898.2013

Demyttenaere K, 2005, INT J NEUROPSYCHOPH, V8, P93, DOI 10.1017/S1461145704004729

Dixon ML, 2014, NEUROSCI BIOBEHAV R, V45, P9, DOI 10.1016/j.neubiorev.2014.04.011

Dobryakova E, 2013, J INT NEUROPSYCH SOC, V19, P849, DOI 10.1017/S1355617713000684

Dowlati Y, 2010, BIOL PSYCHIAT, V67, P446, DOI 10.1016/j.biopsych.2009.09.033

Draper A, 2018, NEUROPSYCHOPHARMACOL, V43, P1107, DOI 10.1038/npp.2017.231

Enoka RM, 2011, J ELECTROMYOGR KINES, V21, P208, DOI 10.1016/j.jelekin.2010.10.006

Felger JC, 2017, NEUROPSYCHOPHARMACOL, V42, P216, DOI 10.1038/npp.2016.143

Fleming SM, 2012, PHILOS T R SOC B, V367, P1338, DOI 10.1098/rstb.2011.0417

Fleming SM, 2010, SCIENCE, V329, P1541, DOI 10.1126/science.1191883

Gendolla, 2011, MOTIVATION AFFECTS C, P61, DOI DOI 10.1037/13090-003

Grill-Spector K, 2006, TRENDS COGN SCI, V10, P14, DOI 10.1016/j.tics.2005.11.006

Grinband J, 2011, NEUROIMAGE, V57, P303, DOI 10.1016/j.neuroimage.2010.12.027

Haber SN, 2010, NEUROPSYCHOPHARMACOL, V35, P4, DOI 10.1038/npp.2009.129

Hartmann MN, 2013, BEHAV PROCESS, V100, P192, DOI 10.1016/j.beproc.2013.09.014

HEEGER DJ, 1992, VISUAL NEUROSCI, V9, P181, DOI 10.1017/S0952523800009640

Helton WS, 2015, COGNITION, V134, P165, DOI 10.1016/j.cognition.2014.10.001

Hockey G.R.J., 2011, COGNITIVE FATIGUE MU, P167, DOI [10.1037/12343-008, DOI 10.1037/12343-008]

Holroyd CB, 2015, PSYCHOL REV, V122, P54, DOI 10.1037/a0038339

Holroyd CB, 2012, TRENDS COGN SCI, V16, P122, DOI 10.1016/j.tics.2011.12.008

Hopstaken JF, 2015, PSYCHOPHYSIOLOGY, V52, P305, DOI 10.1111/psyp.12339

Hou LJ, 2016, FRONT PSYCHOL, V7, DOI 10.3389/fpsyg.2016.01154

Inzlicht M, 2012, PERSPECT PSYCHOL SCI, V7, P450, DOI 10.1177/1745691612454134

Job V, 2010, PSYCHOL SCI, V21, P1686, DOI 10.1177/0956797610384745

Kennerley SW, 2009, EUR J NEUROSCI, V29, P2061, DOI 10.1111/j.1460-9568.2009.06743.x

Kennerley SW, 2009, J COGNITIVE NEUROSCI, V21, P1162, DOI 10.1162/jocn.2009.21100

Klein-Flugge MC, 2015, PLOS COMPUT BIOL, V11, DOI 10.1371/journal.pcbi.1004116

Klein-Flugge MC, 2016, J NEUROSCI, V36, P10002, DOI 10.1523/JNEUROSCI.0292-16.2016

Kolling N, 2016, CURR OPIN NEUROBIOL, V37, P36, DOI 10.1016/j.conb.2015.12.007

Kolling N, 2016, NAT NEUROSCI, V19, P1280, DOI 10.1038/nn.4382

Kool W, 2010, J EXP PSYCHOL GEN, V139, P665, DOI 10.1037/a0020198

KRUPP LB, 1989, ARCH NEUROL-CHICAGO, V46, P1121, DOI 10.1001/archneur.1989.00520460115022

Kuppuswamy A, 2017, BRAIN, V140, P2240, DOI 10.1093/brain/awx153

Kurniawan IT, 2013, J NEUROSCI, V33, P6160, DOI 10.1523/JNEUROSCI.4777-12.2013

Kurzban R, 2013, BEHAV BRAIN SCI, V36, P661, DOI 10.1017/S0140525X12003196

Le Heron C, 2018, NEUROPSYCHOLOGIA, V118, P54, DOI 10.1016/j.neuropsychologia.2017.07.003

Lim J, 2010, NEUROIMAGE, V49, P3426, DOI 10.1016/j.neuroimage.2009.11.020

Liu JZ, 2002, BRAIN RES, V957, P320, DOI 10.1016/S0006-8993(02)03665-X

Lockwood PL, 2017, NAT HUM BEHAV, V1, DOI 10.1038/s41562-017-0131

Lorist MM, 2005, COGNITIVE BRAIN RES, V24, P199, DOI 10.1016/j.cogbrainres.2005.01.018

MACKWORTH JF, 1964, CAN J PSYCHOLOGY, V18, P209, DOI 10.1037/h0083302

Magno E, 2006, J NEUROSCI, V26, P4769, DOI 10.1523/JNEUROSCI.0369-06.2006

Manohar SG, 2015, CURR BIOL, V25, P1707, DOI 10.1016/j.cub.2015.05.038

Marcora S, 2009, J APPL PHYSIOL, V106, P2060, DOI 10.1152/japplphysiol.90378.2008

MESULAM MM, 1982, J COMP NEUROL, V212, P38, DOI 10.1002/cne.902120104

Meyniel F, 2016, ELIFE, V5, DOI 10.7554/eLife.17282

Meyniel F, 2014, PLOS COMPUT BIOL, V10, DOI 10.1371/journal.pcbi.1003584

Meyniel F, 2014, J NEUROSCI, V34, P1, DOI 10.1523/JNEUROSCI.1711-13.2014

Meyniel F, 2013, P NATL ACAD SCI USA, V110, P2641, DOI 10.1073/pnas.1211925110

Micklewright D, 2017, SPORTS MED, V47, P2375, DOI 10.1007/s40279-017-0711-5

Miller EM, 2012, PLOS ONE, V7, DOI 10.1371/journal.pone.0038680

Mockel T, 2015, SCI REP-UK, V5, DOI 10.1038/srep10113

MUFSON EJ, 1982, J COMP NEUROL, V212, P23, DOI 10.1002/cne.902120103

Naccache L, 2005, NEUROPSYCHOLOGIA, V43, P1318, DOI 10.1016/j.neuropsychologia.2004.11.024

Neubert FX, 2015, P NATL ACAD SCI USA, V112, pE2695, DOI 10.1073/pnas.1410767112

NUECHTERLEIN KH, 1983, SCIENCE, V220, P327, DOI 10.1126/science.6836276

Otto T, 2014, SOC COGN AFFECT NEUR, V9, P307, DOI 10.1093/scan/nss136

Palomero-Gallagher N, 2008, J COMP NEUROL, V508, P906, DOI 10.1002/cne.21684

PANDYA DN, 1981, EXP BRAIN RES, V42, P319, DOI 10.1007/BF00237497

Parent M, 2006, J COMP NEUROL, V496, P202, DOI 10.1002/cne.20925

Parvizi J, 2013, NEURON, V80, P1359, DOI 10.1016/j.neuron.2013.10.057

Petrides M, 1999, EUR J NEUROSCI, V11, P1011, DOI 10.1046/j.1460-9568.1999.00518.x

Petrides M, 2006, J COMP NEUROL, V498, P227, DOI 10.1002/cne.21048

Prevost C, 2010, J NEUROSCI, V30, P14080, DOI 10.1523/JNEUROSCI.2752-10.2010

Procyk E, 2016, CEREB CORTEX, V26, P467, DOI 10.1093/cercor/bhu213

Ridderinkhof KR, 2004, SCIENCE, V306, P443, DOI 10.1126/science.1100301

Salamone JD, 2016, BRAIN, V139, P1325, DOI 10.1093/brain/aww050

Sarter M, 2006, BRAIN RES REV, V51, P145, DOI 10.1016/j.brainresrev.2005.11.002

Schmidt L, 2012, PLOS BIOL, V10, DOI 10.1371/journal.pbio.1001266

Shalev L, 2011, NEUROPSYCHOLOGIA, V49, P2584, DOI 10.1016/j.neuropsychologia.2011.05.006

Shenhav A, 2017, ANNU REV NEUROSCI, V40, P99, DOI 10.1146/annurev-neuro-072116-031526

Shenhav A, 2013, NEURON, V79, P217, DOI 10.1016/j.neuron.2013.07.007

SHIGIHARA Y, 2013, NEUROL PSYCHIAT BRAI, V19, DOI [10.1016/j.npbr.2012.07.002, DOI 10.1016/J.NPBR.2012.07.002]

Sidhu SK, 2013, SPORTS MED, V43, P437, DOI 10.1007/s40279-013-0020-6

Skapinakis P, 2003, INT REV PSYCHIATR, V15, P57, DOI 10.1080/0954026021000045958

Stephan KE, 2016, FRONT HUM NEUROSCI, V10, DOI 10.3389/fnhum.2016.00550

Studer B, 2016, PROG BRAIN RES, V229, P25, DOI 10.1016/bs.pbr.2016.06.014

Tanaka M, 2006, BMC NEUROL, V6, DOI 10.1186/1471-2377-6-9

Tanaka M, 2014, BRAIN RES, V1561, P60, DOI 10.1016/j.brainres.2014.03.009

Tanaka M, 2012, NEUROSCI BIOBEHAV R, V36, P727, DOI 10.1016/j.neubiorev.2011.10.004

Ullsperger M, 2014, PHYSIOL REV, V94, P35, DOI 10.1152/physrev.00041.2012

Ullsperger M, 2010, BRAIN STRUCT FUNCT, V214, P629, DOI 10.1007/s00429-010-0261-1

Umemoto A., 2017, BIORXIV, DOI [10.1101/199687, DOI 10.1101/199687]

Van Cutsem J, 2017, SPORTS MED, V47, P1569, DOI 10.1007/s40279-016-0672-0

Vassena E, 2017, J COGNITIVE NEUROSCI, V29, P1633, DOI 10.1162/jocn_a_01160

Vassena E, 2014, PLOS ONE, V9, DOI 10.1371/journal.pone.0091008

Verguts T, 2015, FRONT BEHAV NEUROSCI, V9, DOI [10.3389/fnbeh.2015.0005, 10.3389/fnbeh.2015.00057]

VOGT BA, 1987, J COMP NEUROL, V262, P271, DOI 10.1002/cne.902620208

Vollestad NK, 1997, J NEUROSCI METH, V74, P219, DOI 10.1016/S0165-0270(97)02251-6

Walton ME, 2006, NEURAL NETWORKS, V19, P1302, DOI 10.1016/j.neunet.2006.03.005

Warm JS, 2008, HUM FACTORS, V50, P433, DOI 10.1518/001872008X312152

Westbrook A, 2016, NEURON, V89, P695, DOI [10.1016/j.neuron.2015.12.029, 10.1016/j.neuron.2016.07.020]

Westbrook A, 2013, PLOS ONE, V8, DOI 10.1371/journal.pone.0068210

Williamson JW, 2006, EXP PHYSIOL, V91, P51, DOI 10.1113/expphysiol.2005.032037

Wilson CRE, 2016, PLOS BIOL, V14, DOI 10.1371/journal.pbio.1002576

Winstanley CA, 2016, J NEUROSCI, V36, P12069, DOI 10.1523/JNEUROSCI.1713-16.2016

Wittmann MK, 2016, NAT COMMUN, V7, DOI 10.1038/ncomms12327

Wylie GR, 2017, COGN AFFECT BEHAV NE, V17, P838, DOI 10.3758/s13415-017-0515-y

NR 132

TC 53

Z9 54

U1 9

U2 40

PU PERGAMON-ELSEVIER SCIENCE LTD

PI OXFORD

PA THE BOULEVARD, LANGFORD LANE, KIDLINGTON, OXFORD OX5 1GB, ENGLAND

SN 0028-3932

EI 1873-3514

J9 NEUROPSYCHOLOGIA

JI Neuropsychologia

PD FEB

PY 2019

VL 123

BP 141

EP 151

DI 10.1016/j.neuropsychologia.2018.04.030

PG 11

WC Behavioral Sciences; Neurosciences; Psychology, Experimental

WE Science Citation Index Expanded (SCI-EXPANDED); Social Science Citation Index (SSCI)

SC Behavioral Sciences; Neurosciences & Neurology; Psychology

GA HO3XI

UT WOS:000460855900013

PM 29738794

OA Green Submitted

DA 2022-05-04

ER

PT J

AU Otani, H

Kaya, M

Tamaki, A

Watson, P

AF Otani, Hidenori

Kaya, Mitsuharu

Tamaki, Akira

Watson, Phillip

TI Separate and combined effects of exposure to heat stress and mental

fatigue on endurance exercise capacity in the heat

SO EUROPEAN JOURNAL OF APPLIED PHYSIOLOGY

LA English

DT Article

DE Cognitive function; Core temperature; Fatigue; Heat stress; Skin

temperature; Thermoregulation

ID PROLONGED EXERCISE; PHYSICAL PERFORMANCE; RUNNING PERFORMANCE; PERCEIVED

EXERTION; HOT ENVIRONMENT; TEMPERATURE; BODY; ACCLIMATION; HUMANS;

HYPOHYDRATION

AB This study investigated the effects of exposure to pre-exercise heat stress and mental fatigue on endurance exercise capacity in a hot environment.

Eight volunteers completed four cycle exercise trials at 80% maximum oxygen uptake until exhaustion in an environmental chamber maintained at 30 A degrees C and 50% relative humidity. The four trials required them to complete a 90 min pre-exercise routine of either a seated rest (CON), a prolonged demanding cognitive task to induce mental fatigue (MF), warm water immersion at 40 A degrees C during the last 30 min to induce increasing core temperature (WI), or a prolonged demanding cognitive task and warm water immersion at 40 A degrees C during the last 30 min (MF + WI).

Core temperature when starting exercise was higher following warm water immersion (similar to 38 A degrees C; WI and MF + WI) than with no water immersion (similar to 36.8 A degrees C; CON and MF, P < 0.001). Self-reported mental fatigue when commencing exercise was higher following cognitive task (MF and MF + WI) than with no cognitive task (CON and WI; P < 0.05). Exercise time to exhaustion was reduced by warm water immersion (P < 0.001) and cognitive task (P < 0.05). Compared with CON (18 +/- 7 min), exercise duration reduced 0.8, 26.6 and 46.3% in MF (17 +/- 7 min), WI (12 +/- 5 min) and MF + WI (9 +/- 3 min), respectively.

This study demonstrates that endurance exercise capacity in a hot environment is impaired by either exposure to pre-exercise heat stress or mental fatigue, and this response is synergistically increased during combined exposure to them.

C1 [Otani, Hidenori] Himeji Dokkyo Univ, Fac Hlth Care Sci, 7-2-1 Kamiono, Himeji, Hyogo 6708524, Japan.

[Kaya, Mitsuharu; Tamaki, Akira] Hyogo Univ Hlth Sci, Kobe, Hyogo, Japan.

[Watson, Phillip] Vrije Univ Brussel, Dept Human Physiol, Brussels, Belgium.

RP Otani, H (corresponding author), Himeji Dokkyo Univ, Fac Hlth Care Sci, 7-2-1 Kamiono, Himeji, Hyogo 6708524, Japan.

EM hotani@himeji-du.ac.jp

RI Tamaki, Akira/ABA-5962-2020

OI Otani, Hidenori/0000-0001-7737-392X

CR Barwood MJ, 2008, MED SCI SPORT EXER, V40, P387, DOI 10.1249/mss.0b013e31815adf31

BORG GAV, 1982, MED SCI SPORT EXER, V14, P377, DOI 10.1249/00005768-198205000-00012

Cheung SS, 1998, J APPL PHYSIOL, V84, P1731, DOI 10.1152/jappl.1998.84.5.1731

Ely BR, 2009, J APPL PHYSIOL, V107, P1519, DOI 10.1152/japplphysiol.00577.2009

FRAYN KN, 1983, J APPL PHYSIOL, V55, P628, DOI 10.1152/jappl.1983.55.2.628

Galloway SDR, 1997, MED SCI SPORT EXER, V29, P1240, DOI 10.1097/00005768-199709000-00018

Gonzalez-Alonso J, 1999, J APPL PHYSIOL, V86, P1032, DOI 10.1152/jappl.1999.86.3.1032

Gonzalez-Alonso J, 2008, J PHYSIOL-LONDON, V586, P45, DOI 10.1113/jphysiol.2007.142158

Hardy JD, 1938, J NUTR, V15, P477, DOI 10.1093/jn/15.5.477

ISO, 1995, 10551 ISO

Jay O, 2007, AM J PHYSIOL-REG I, V292, pR167, DOI 10.1152/ajpregu.00338.2006

Lloyd Alex, 2016, Temperature (Austin), V3, P514, DOI 10.1080/23328940.2016.1189991

Lloyd A, 2016, J APPL PHYSIOL, V120, P567, DOI 10.1152/japplphysiol.00876.2015

MacMahon C, 2014, J SPORT EXERCISE PSY, V36, P375, DOI 10.1123/jsep.2013-0249

Marcora SM, 2009, J APPL PHYSIOL, V106, P857, DOI 10.1152/japplphysiol.91324.2008

Maughan RJ, 2007, J AM COLL NUTR, V26, p604S, DOI 10.1080/07315724.2007.10719666

Maughan RJ, 2007, J SPORT SCI, V25, P797, DOI 10.1080/02640410600875143

Maughan RJ, 2012, EUR J APPL PHYSIOL, V112, P2313, DOI 10.1007/s00421-011-2206-7

Meeusen R, 2010, SCAND J MED SCI SPOR, V20, P19, DOI 10.1111/j.1600-0838.2010.01205.x

MINARD D, 1970, PHYSL BEHAVIORAL TEM, P345

Mora-Rodriguez R, 2010, EUR J APPL PHYSIOL, V109, P973, DOI 10.1007/s00421-010-1436-4

NIELSEN B, 1993, J PHYSIOL-LONDON, V460, P467, DOI 10.1113/jphysiol.1993.sp019482

Nybo L, 2001, J PHYSIOL-LONDON, V534, P279, DOI 10.1111/j.1469-7793.2001.t01-1-00279.x

Otani H, 2006, J THERM BIOL, V31, P186, DOI 10.1016/j.jtherbio.2005.11.018

Otani H, 2016, EUR J APPL PHYSIOL, V116, P769, DOI 10.1007/s00421-016-3335-9

Otani H, 2013, J SPORT SCI MED, V12, P197

Pageaux B, 2015, FRONT HUM NEUROSCI, V9, DOI 10.3389/fnhum.2015.00067

Pageaux B, 2014, EUR J APPL PHYSIOL, V114, P1095, DOI 10.1007/s00421-014-2838-5

Pageaux B, 2013, MED SCI SPORT EXER, V45, P2254, DOI 10.1249/MSS.0b013e31829b504a

RAMANATHAN NL, 1964, J APPL PHYSIOL, V19, P531, DOI 10.1152/jappl.1964.19.3.531

ROBERTS MF, 1977, J APPL PHYSIOL, V43, P133, DOI 10.1152/jappl.1977.43.1.133

Rowell L.B., 1986, HUMAN CIRCULATION RE

Sawka MN, 2011, COMPR PHYSIOL, V1, P1883, DOI 10.1002/cphy.c100082

Sawka MN, 2012, EXP PHYSIOL, V97, P327, DOI [10.1113/expphysiol.2011.061026, 10.1113/expphysiol.2011.061002]

Sgherza AL, 2002, J APPL PHYSIOL, V93, P2023, DOI 10.1152/japplphysiol.00521.2002

Smith MR, 2016, MED SCI SPORT EXER, V48, P267, DOI 10.1249/MSS.0000000000000762

Smith MR, 2015, MED SCI SPORT EXER, V47, P1682, DOI 10.1249/MSS.0000000000000592

STERNBERG S, 1969, AM SCI, V57, P421

Stevens CJ, 2016, SCAND J MED SCI SPOR, V26, P1209, DOI 10.1111/sms.12555

Stevens CJ, 2017, SPORTS MED, V47, P829, DOI 10.1007/s40279-016-0625-7

Stohr EJ, 2011, J APPL PHYSIOL, V111, P891, DOI 10.1152/japplphysiol.00528.2011

Stroop JR, 1935, J EXP PSYCHOL, V18, P643, DOI 10.1037/h0054651

Terry PC, 2003, PSYCHOL SPORT EXERC, V4, P125, DOI 10.1016/S1469-0292(01)00035-8

Thelwell RC, 2003, SPORT PSYCHOL, V17, P318, DOI 10.1123/tsp.17.3.318

Trinity JD, 2010, J APPL PHYSIOL, V109, P745, DOI 10.1152/japplphysiol.00377.2010

Watson P, 2005, J PHYSIOL-LONDON, V565, P873, DOI 10.1113/jphysiol.2004.079202

Watson P, 2012, MED SCI SPORT EXER, V44, P336, DOI 10.1249/MSS.0b013e31822dc5ed

WESNES K, 1984, PSYCHOPHARMACOLOGY, V82, P147, DOI 10.1007/BF00427761

NR 48

TC 17

Z9 17

U1 3

U2 39

PU SPRINGER

PI NEW YORK

PA 233 SPRING ST, NEW YORK, NY 10013 USA

SN 1439-6319

EI 1439-6327

J9 EUR J APPL PHYSIOL

JI Eur. J. Appl. Physiol.

PD JAN

PY 2017

VL 117

IS 1

BP 119

EP 129

DI 10.1007/s00421-016-3504-x

PG 11

WC Physiology; Sport Sciences

WE Science Citation Index Expanded (SCI-EXPANDED); Social Science Citation Index (SSCI)

SC Physiology; Sport Sciences

GA EL0LG

UT WOS:000394313300012

PM 27864637

DA 2022-05-04

ER

PT J

AU Schouppe, S

Van Oosterwijck, J

Wiersema, JR

Van Damme, S

Willems, T

Danneels, L

AF Schouppe, Stijn

Van Oosterwijck, Jessica

Wiersema, Jan R.

Van Damme, Stefaan

Willems, Tine

Danneels, Lieven

TI Physical or Cognitive Exertion Does Not Influence Cortical Movement

Preparation for Rapid Arm Movements

SO MOTOR CONTROL

LA English

DT Article

DE central nerve system; contingent negative variation;

electroencephalography

ID ANTICIPATORY POSTURAL ADJUSTMENTS; CONTINGENT NEGATIVE-VARIATION; BACK

MUSCLE FATIGUE; MENTAL FATIGUE; STIMULUS ANTICIPATION; MOTOR

PREPARATION; AEROBIC EXERCISE; PERFORMANCE; PAIN; RELIABILITY

AB The contribution of central factors to movement preparation (e.g., the contingent negative variation [CNV]) and the influence of fatigue on such factors are still unclear, even though executive cognitive functions are regarded as key elements in motor control. Therefore, this study examined CNV amplitude with electroencephalography in 22 healthy humans during a rapid arm movement task prior to and following three experimental conditions: (a) a no exertion/control condition, (b) a physical exertion, and (c) a cognitive exertion. CNV amplitude was affected neither by a single bout of physical/cognitive exertion nor by the control condition. Furthermore, no time-on-task effects of the rapid arm movement task on the CNV were found. Exertion did not affect cortical movement preparation, which is in contrast to previous findings regarding time-on-task effects of exertion on CNV. Based on the current findings, the rapid arm movement task is deemed suitable to measure cortical movement preparation, without being affected by learning effects and physical/cognitive exertion.

C1 [Schouppe, Stijn; Van Oosterwijck, Jessica; Willems, Tine; Danneels, Lieven] Univ Ghent, SPINE Res Unit Ghent, Dept Rehabil Sci & Physiotherapy, Fac Med & Hlth Sci, Ghent, Belgium.

[Schouppe, Stijn; Van Oosterwijck, Jessica] Pain Mot Int Res Grp, Brussels, Belgium.

[Van Oosterwijck, Jessica] Res Fdn Flanders FWO, Brussels, Belgium.

[Wiersema, Jan R.; Van Damme, Stefaan] Univ Ghent, Dept Expt Clin & Hlth Psychol, Ghent, Belgium.

RP Van Oosterwijck, J (corresponding author), Univ Ghent, SPINE Res Unit Ghent, Dept Rehabil Sci & Physiotherapy, Fac Med & Hlth Sci, Ghent, Belgium.

EM Jessica.VanOosterwijck@ugent.be

RI Van Oosterwijck, Jessica/O-5565-2019; Danneels, Lieven/F-9883-2017

OI Van Oosterwijck, Jessica/0000-0002-8946-4383; Danneels,

Lieven/0000-0002-3030-2697

FU Special Research Fund/Bijzonder Onderzoeksfonds (BOF) at Ghent

University [BOF14/IOP/067]; Research Foundation-Flanders (FWO)FWO

[12L5616N]

FX This study was funded by an interdisciplinary grant (BOF14/IOP/067) from

the Special Research Fund/Bijzonder Onderzoeksfonds (BOF) at Ghent

University. Van Oosterwijck is a Postdoctoral Fellow funded by the

Research Foundation-Flanders (FWO; grant number 12L5616N). The funding

sources had no involvement in the development of the study design; in

the collection, analysis, and interpretation of data; in the writing of

the report; and in the decision to submit the article for publication.

CR Abd-Elfattah HM, 2015, J ADV RES, V6, P351, DOI 10.1016/j.jare.2015.01.011

Achttien RJ, 2011, KNGF RICHTLIJN HARTR

Allison GT, 2002, CLIN BIOMECH, V17, P414, DOI 10.1016/S0268-0033(02)00029-3

Allison GT, 2001, MANUAL THER, V6, P221, DOI 10.1054/math.2001.0412

Ansari TL, 2011, BIOL PSYCHOL, V86, P337, DOI 10.1016/j.biopsycho.2010.12.013

Apkarian AV, 2011, PAIN, V152, pS49, DOI 10.1016/j.pain.2010.11.010

Barthel T, 2001, AMINO ACIDS, V20, P63, DOI 10.1007/s007260170066

Bisson EJ, 2011, GAIT POSTURE, V33, P83, DOI 10.1016/j.gaitpost.2010.10.001

Boksem MAS, 2008, BRAIN RES REV, V59, P125, DOI 10.1016/j.brainresrev.2008.07.001

Boksem MAS, 2006, BIOL PSYCHOL, V72, P123, DOI 10.1016/j.biopsycho.2005.08.007

Booth M, 2000, RES Q EXERCISE SPORT, V71, pS114

Borg G., 1998, BORGS PERCEIVED EXER

BORG GAV, 1982, MED SCI SPORT EXER, V14, P377, DOI 10.1249/00005768-198205000-00012

Boucher JA, 2012, J MANIP PHYSIOL THER, V35, P662, DOI 10.1016/j.jmpt.2012.10.003

BRUNIA CHM, 1988, ELECTROEN CLIN NEURO, V69, P234, DOI 10.1016/0013-4694(88)90132-0

Carroll TJ, 2017, J APPL PHYSIOL, V122, P1068, DOI 10.1152/japplphysiol.00775.2016

Chaudhuri A, 2004, LANCET, V363, P978, DOI 10.1016/S0140-6736(04)15794-2

Cohen J., 1988, STAT POWER ANAL BEHA, V2nd ed

CONNOR WH, 1969, J EXP PSYCHOL, V82, P310, DOI 10.1037/h0028181

Coorevits P, 2008, J ELECTROMYOGR KINES, V18, P997, DOI 10.1016/j.jelekin.2007.10.012

Corbeil P, 2003, GAIT POSTURE, V18, P92, DOI 10.1016/S0966-6362(02)00198-4

Craig CL, 2003, MED SCI SPORT EXER, V35, P1381, DOI 10.1249/01.MSS.0000078924.61453.FB

DAMEN EJP, 1987, PSYCHOPHYSIOLOGY, V24, P700, DOI 10.1111/j.1469-8986.1987.tb00353.x

de Morree HM, 2014, J APPL PHYSIOL, V117, P1514, DOI 10.1152/japplphysiol.00898.2013

de Morree HM, 2012, PSYCHOPHYSIOLOGY, V49, P1242, DOI 10.1111/j.1469-8986.2012.01399.x

DEGROOT MH, 1991, GEDRAG GEZONDHEID, V20, P46

Du Rietz E, 2019, BEHAV BRAIN RES, V359, P474, DOI 10.1016/j.bbr.2018.11.024

Enoka RM, 2016, MED SCI SPORT EXER, V48, P2228, DOI 10.1249/MSS.0000000000000929

FREUDE G, 1987, EUR J APPL PHYSIOL, V56, P105, DOI 10.1007/BF00696384

Fujiwara K, 2009, J ELECTROMYOGR KINES, V19, P113, DOI 10.1016/j.jelekin.2007.08.003

Gaillard A. W. K., 1991, J PSYCHOPHYSIOL, V5, P337

Halperin I, 2015, EUR J APPL PHYSIOL, V115, P2031, DOI 10.1007/s00421-015-3249-y

Hedayati R, 2014, J BACK MUSCULOSKELET, V27, P33, DOI 10.3233/BMR-130416

Hodges PW, 1997, EXP BRAIN RES, V114, P362, DOI 10.1007/PL00005644

Hodges PW, 2003, J ELECTROMYOGR KINES, V13, P361, DOI 10.1016/S1050-6411(03)00042-7

Jacobs JV, 2008, CLIN NEUROPHYSIOL, V119, P1431, DOI 10.1016/j.clinph.2008.02.015

Jacobs JV, 2010, CLIN NEUROPHYSIOL, V121, P431, DOI 10.1016/j.clinph.2009.11.076

Jacobs JV, 2009, BEHAV NEUROSCI, V123, P455, DOI 10.1037/a0014479

Johnston J, 2001, CLIN NEUROPHYSIOL, V112, P68, DOI 10.1016/S1388-2457(00)00452-1

Kappenman E. S, 2011, OXFORD HDB EVENT REL

Kato Y, 2009, INT J PSYCHOPHYSIOL, V72, P204, DOI 10.1016/j.ijpsycho.2008.12.008

Knox MF, 2018, SPINE J, V18, P1934, DOI 10.1016/j.spinee.2018.06.008

KOK A, 1978, BIOL PSYCHOL, V6, P219, DOI 10.1016/0301-0511(78)90024-8

Lakens D, 2013, FRONT PSYCHOL, V4, DOI 10.3389/fpsyg.2013.00863

Luck SJ, 2014, INTRODUCTION TO THE EVENT-RELATED POTENTIAL TECHNIQUE, 2ND EDITION, P1

Luck SJ, 2017, PSYCHOPHYSIOLOGY, V54, P146, DOI 10.1111/psyp.12639

MACKWORTH JF, 1964, CAN J PSYCHOLOGY, V18, P209, DOI 10.1037/h0083302

Maeda K, 2007, GAIT POSTURE, V25, P78, DOI 10.1016/j.gaitpost.2006.01.004

Marcora SM, 2009, J APPL PHYSIOL, V106, P857, DOI 10.1152/japplphysiol.91324.2008

Marshall P, 2003, J ELECTROMYOGR KINES, V13, P477, DOI 10.1016/S1050-6411(03)00027-0

Marshall PW, 2008, ARCH PHYS MED REHAB, V89, P1305, DOI 10.1016/j.apmr.2007.11.051

Marshall PWM, 2014, EXP BRAIN RES, V232, P3515, DOI 10.1007/s00221-014-4040-8

Masaki H, 2004, PSYCHOPHYSIOLOGY, V41, P220, DOI 10.1111/j.1469-8986.2004.00150.x

Mochizuki G, 2017, NEUROSCIENCE, V348, P143, DOI 10.1016/j.neuroscience.2017.02.014

Moseley GL, 2012, NEUROREHAB NEURAL RE, V26, P646, DOI 10.1177/1545968311433209

Muller T, 2019, NEUROPSYCHOLOGIA, V123, P141, DOI 10.1016/j.neuropsychologia.2018.04.030

Muller-Gethmann H, 2000, PSYCHOPHYSIOLOGY, V37, P507, DOI 10.1111/1469-8986.3740507

Pageaux B, 2015, FRONT HUM NEUROSCI, V9, DOI 10.3389/fnhum.2015.00067

Paillard T, 2012, NEUROSCI BIOBEHAV R, V36, P162, DOI 10.1016/j.neubiorev.2011.05.009

Park RJ, 2014, NEUROSCIENCE, V261, P161, DOI 10.1016/j.neuroscience.2013.12.037

ROSLER F, 1991, ELECTROEN CLIN NEURO, P116

Schillings ML, 2006, EUR J APPL PHYSIOL, V97, P521, DOI 10.1007/s00421-006-0211-z

Schouppe S, 2019, EXP BRAIN RES, V237, P3011, DOI 10.1007/s00221-019-05585-0

Sparto PJ, 1999, SPINE, V24, P1791, DOI 10.1097/00007632-199909010-00008

Stevens VK, 2006, AM J PHYS MED REHAB, V85, P727, DOI 10.1097/01.phm.0000233180.88299.f6

Strang AJ, 2008, J SPORT MED PHYS FIT, V48, P9

Strang AJ, 2007, EXP BRAIN RES, V178, P49, DOI 10.1007/s00221-006-0710-5

Strang AJ, 2009, EXP BRAIN RES, V197, P245, DOI 10.1007/s00221-009-1908-0

Stroth S, 2009, BRAIN RES, V1269, P114, DOI 10.1016/j.brainres.2009.02.073

Suehiro T, 2018, J ELECTROMYOGR KINES, V39, P128, DOI 10.1016/j.jelekin.2018.02.008

Tanaka M, 2014, BRAIN RES, V1561, P60, DOI 10.1016/j.brainres.2014.03.009

TECCE JJ, 1972, PSYCHOL BULL, V77, P73, DOI 10.1037/h0032177

Peixoto LRT, 2010, IEEE ENG MED BIO, P4922, DOI 10.1109/IEMBS.2010.5627256

The-IPAQ-Group, 1998, INT PHYS ACT QUEST

Tomita H, 2012, HUM MOVEMENT SCI, V31, P578, DOI 10.1016/j.humov.2011.07.013

Tsai CL, 2014, PSYCHONEUROENDOCRINO, V41, P121, DOI 10.1016/j.psyneuen.2013.12.014

Van Damme B, 2014, J ELECTROMYOGR KINES, V24, P636, DOI 10.1016/j.jelekin.2014.05.008

Van Damme S, 2018, PAIN, V159, P7, DOI 10.1097/j.pain.0000000000001054

van der Linden D, 2003, ACTA PSYCHOL, V113, P45, DOI 10.1016/S0001-6918(02)00150-6

van Mechelen, 2004, TIJDSCHRIFT GEZONDHE, V82, P457

VANBOXTEL GJM, 1994, BIOL PSYCHOL, V38, P37, DOI 10.1016/0301-0511(94)90048-5

Vercoulen J.H.M.M., 1999, GEDRAGSTHERAPIE, V32, P31

VERCOULEN JHMM, 1994, J PSYCHOSOM RES, V38, P383, DOI 10.1016/0022-3999(94)90099-X

Wald FD., 1990, NEDERLANDS TIJDSCHRI, V45, P86

WALTER WG, 1964, NATURE, V203, P380, DOI 10.1038/203380a0

Wang-Price Sharon, 2017, Int J Exerc Sci, V10, P213

NR 86

TC 1

Z9 1

U1 0

U2 5

PU HUMAN KINETICS PUBL INC

PI CHAMPAIGN

PA 1607 N MARKET ST, PO BOX 5076, CHAMPAIGN, IL 61820-2200 USA

SN 1087-1640

EI 1543-2696

J9 MOTOR CONTROL

JI Motor Control

PD OCT

PY 2020

VL 24

IS 4

BP 473

EP 498

DI 10.1123/mc.2019-0115

PG 26

WC Neurosciences; Sport Sciences

WE Science Citation Index Expanded (SCI-EXPANDED)

SC Neurosciences & Neurology; Sport Sciences

GA NV6AN

UT WOS:000574402000001

PM 32717721

OA Green Published

DA 2022-05-04

ER

PT J

AU Bray, SR

Graham, JD

Ginis, KAM

Hicks, AL

AF Bray, Steven R.

Graham, Jeffrey D.

Ginis, Kathleen A. Martin

Hicks, Audrey L.

TI Cognitive task performance causes impaired maximum force production in

human hand flexor muscles

SO BIOLOGICAL PSYCHOLOGY

LA English

DT Article

DE Self-regulation; Muscle fatigue; Perceived exertion

ID SELF-CONTROL; STRENGTH MODEL; EGO DEPLETION; FATIGUE; EXERCISE; BRAIN;

GLUCOSE; STRESS; WORK; PHYSIOLOGY

AB The purpose of this study was to investigate effects of demanding cognitive task performance on intermittent maximum voluntary muscle contraction (MVC) force production. Participants performed either a modified Stroop or control task for 22 min. After the first min and at 3-min intervals thereafter, participants rated fatigue, perceived mental exertion and performed a 4-s MVC handgrip squeeze. A mixed ANOVA showed a significant interaction, F(7, 259) = 2.43, p=.02, with a significant linear reduction in MVC force production over time in the cognitively depleting condition (p=.01) and no change for controls. Ratings of perceived mental exertion, F(7, 252)=2.39, p < .05, mirrored the force production results with a greater linear increase over time in the cognitive depletion condition (p < .001) compared to controls. Findings support current views that performance of cognitively demanding tasks diminishes central nervous system resources that govern self-regulation of physical tasks requiring maximal voluntary effort. (C) 2011 Elsevier B.V. All rights reserved.

C1 [Bray, Steven R.] McMaster Univ, Dept Kinesiol, Hamilton, ON L8S 4K1, Canada.

RP Bray, SR (corresponding author), McMaster Univ, Dept Kinesiol, 1280 Main St W, Hamilton, ON L8S 4K1, Canada.

EM sbray@mcmaster.ca

RI Hicks, Audrey/AAF-7199-2021

OI MARTIN GINIS, KATHLEEN/0000-0002-7076-3594; Graham,

Jeffrey/0000-0002-6728-2597; Bray, Steven/0000-0003-0658-3772

CR Ahsberg E, 2000, ERGONOMICS, V43, P252, DOI 10.1080/001401300184594

Baumeister R.F, 1994, LOSING CONTROL WHY P

Baumeister RF, 2007, SOC PERSONAL PSYCHOL, V1, P115, DOI 10.1111/j.1751-9004.2007.00001.x

Baumeister RF, 2007, CURR DIR PSYCHOL SCI, V16, P351, DOI 10.1111/j.1467-8721.2007.00534.x

Bigland-Ritchie B, 1981, Exerc Sport Sci Rev, V9, P75

Blackwood SK, 1998, J NEUROL NEUROSUR PS, V65, P541, DOI 10.1136/jnnp.65.4.541

Borg G., 1998, BORGS PERCEIVED EXER

Bray SR, 2008, PSYCHOPHYSIOLOGY, V45, P337, DOI 10.1111/j.1469-8986.2007.00625.x

Brisswalter J, 2002, SPORTS MED, V32, P555, DOI 10.2165/00007256-200232090-00002

Dalsgaard MK, 2007, J NEUROSCI RES, V85, P3334, DOI 10.1002/jnr.21274

DAVIS JM, 1995, INT J SPORT NUTR, V5, pS29, DOI 10.1123/ijsn.5.s1.s29

ENOKA RM, 1992, J APPL PHYSIOL, V72, P1631, DOI 10.1152/jappl.1992.72.5.1631

Etnier JL, 1997, J SPORT EXERCISE PSY, V19, P249, DOI 10.1123/jsep.19.3.249

Gailliot MT, 2007, PERS SOC PSYCHOL REV, V11, P303, DOI 10.1177/1088868307303030

Gailliot MT, 2007, J PERS SOC PSYCHOL, V92, P325, DOI 10.1037/0022-3514.92.2.325

Gailliot MT, 2008, PERSPECT PSYCHOL SCI, V3, P245, DOI 10.1111/j.1745-6924.2008.00077.x

Gandevia SC, 2001, PHYSIOL REV, V81, P1725, DOI 10.1152/physrev.2001.81.4.1725

Garcin M, 2003, INT J SPORTS MED, V24, P285, DOI 10.1055/s-2003-39502

GIFT AG, 1989, NURS RES, V38, P286

Ginis KAM, 2010, PSYCHOL HEALTH, V25, P1147, DOI 10.1080/08870440903111696

Gruber SA, 2002, NEUROIMAGE, V16, P349, DOI 10.1006/nimg.2002.1089

Hagger MS, 2010, PSYCHOL BULL, V136, P495, DOI 10.1037/a0019486

Hockey GRJ, 1997, BIOL PSYCHOL, V45, P73

Kahneman D., 1973, ATTENTION EFFORT

Kayser B, 2003, EUR J APPL PHYSIOL, V90, P411, DOI 10.1007/s00421-003-0902-7

Larsby B, 2005, INT J AUDIOL, V44, P131, DOI 10.1080/14992020500057244

LARSSON SE, 1995, EUR J APPL PHYSIOL O, V71, P493, DOI 10.1007/BF00238550

Laursen B, 2002, SCAND J WORK ENV HEA, V28, P215, DOI 10.5271/sjweh.668

Lui J.Z, 2003, J NEUROPHYSIOL, V90, P300

Lundberg U, 2002, WORK STRESS, V16, P166, DOI 10.1080/02678370210136699

MacDonell CW, 2005, ERGONOMICS, V48, P1749, DOI 10.1080/00140130500319757

Marcora SM, 2009, J APPL PHYSIOL, V106, P857, DOI 10.1152/japplphysiol.91324.2008

McKenna MJ, 2008, J APPL PHYSIOL, V104, P286, DOI 10.1152/japplphysiol.01139.2007

Milham MP, 2002, BRAIN COGNITION, V49, P277, DOI 10.1006/brcg.2001.1501

Mosso A., 1915, FATIGUE

Muraven M, 2000, PSYCHOL BULL, V126, P247, DOI 10.1037/0033-2909.126.2.247

Muraven M, 1998, J PERS SOC PSYCHOL, V74, P774, DOI 10.1037/0022-3514.74.3.774

Nybo L, 2003, MED SCI SPORT EXER, V35, P589, DOI 10.1249/01.MSS.0000058433.85789.66

Peterson BS, 1999, BIOL PSYCHIAT, V45, P1237, DOI 10.1016/S0006-3223(99)00056-6

SOGAARD K, 2006, J PHYSL, P511

Taylor JL, 2008, J APPL PHYSIOL, V104, P542, DOI 10.1152/japplphysiol.01053.2007

Taylor JL, 2006, CLIN EXP PHARMACOL P, V33, P400, DOI 10.1111/j.1440-1681.2006.04363.x

Tomporowski PD, 2003, ACTA PSYCHOL, V112, P297, DOI 10.1016/S0001-6918(02)00134-8

van Duinen H, 2007, NEUROIMAGE, V35, P1438, DOI 10.1016/j.neuroimage.2007.02.008

Vohs KD, 2011, SOC PSYCHOL PERS SCI, V2, P166, DOI 10.1177/1948550610386123

Waersted M, 2000, EUR J APPL PHYSIOL, V83, P151, DOI 10.1007/s004210000273

Waersted M, 1996, ERGONOMICS, V39, P661, DOI 10.1080/00140139608964488

Wallace HM, 2002, SELF IDENTITY, V1, P35, DOI 10.1080/152988602317232786

NR 48

TC 58

Z9 61

U1 3

U2 26

PU ELSEVIER

PI AMSTERDAM

PA RADARWEG 29, 1043 NX AMSTERDAM, NETHERLANDS

SN 0301-0511

EI 1873-6246

J9 BIOL PSYCHOL

JI Biol. Psychol.

PD JAN

PY 2012

VL 89

IS 1

BP 195

EP 200

DI 10.1016/j.biopsycho.2011.10.008

PG 6

WC Psychology, Biological; Behavioral Sciences; Psychology; Psychology,

Experimental

WE Science Citation Index Expanded (SCI-EXPANDED); Social Science Citation Index (SSCI)

SC Psychology; Behavioral Sciences

GA 884NJ

UT WOS:000299714500026

PM 22020133

DA 2022-05-04

ER

PT J

AU Van Cutsem, J

De Pauw, K

Buyse, L

Marcora, S

Meeusen, R

Roelands, B

AF Van Cutsem, Jeroen

De Pauw, Kevin

Buyse, Luk

Marcora, Samuele

Meeusen, Romain

Roelands, Bart

TI Effects of Mental Fatigue on Endurance Performance in the Heat

SO MEDICINE AND SCIENCE IN SPORTS AND EXERCISE

LA English

DT Article

DE EXERCISE; WHOLE-BODY ENDURANCE PERFORMANCE; HEAT; EFFORT

ID SELF-PACED EXERCISE; PHYSICAL PERFORMANCE; PERCEIVED EXERTION; DISTINCT

ASPECTS; ERP; TEMPERATURE; TASK; HYPERTHERMIA; ATTENTION; HUMANS

AB Purpose: Mental fatigue is a psychobiological state caused by prolonged periods of demanding cognitive activity and has been observed to decrease time trial (TT) endurance performance by similar to 3.5% in normal ambient temperatures. Recently, it has been suggested that heat may augment the negative effect of mental fatigue on cognitive performance, raising the question whether it may also amplify the effect of mental fatigue on TT performance. Methods: In 30 degrees C and 30% relative humidity, 10 endurance-trained male athletes (mean +/- SD; age = 22 +/- 3 yr, W-max = 332 +/- 41 W) completed two experimental conditions: intervention (I; 45-min Stroop task) and control (C; 45-min documentary). Pre-and postintervention/control, cognitive performance was followed up with a 5-min Flanker task. Thereafter, subjects cycled for 45 min at a fixed pace equal to 60% W-max, immediately followed by a self-paced TT in which they had to produce a fixed amount of work (equal to cycling 15 min at 80% W-max) as fast as possible. Results: Self-reported mental fatigue was significantly higher after I compared with C (P < 0.05). Moreover EEG measures also indicated the occurrence of mental fatigue during the Stroop (P < 0.05). TT did not differ between conditions (I = 906 +/- 30 s, C = 916 +/- 29 s). Throughout exercise, physiological (HR, blood lactate, core, and skin temperature) and perceptual measures (perception of effort and thermal sensation) were not affected by mental fatigue. Conclusion: No negative effects of mild mental fatigue were observed on performance and the physiological and perceptual responses to endurance exercise in the heat. Most plausibly, mild mental fatigue does not reduce endurance performance when the brain is already stressed by a hot environment.

C1 [Van Cutsem, Jeroen; De Pauw, Kevin; Buyse, Luk; Meeusen, Romain; Roelands, Bart] Vrije Univ Brussel, Human Physiol Res Grp, Pl Laan 2, B-1050 Brussels, Belgium.

[Van Cutsem, Jeroen; Marcora, Samuele] Univ Kent Medway, Sch Sport & Exercise Sci, Endurance Res Grp, Chatham, Kent, England.

[Meeusen, Romain] James Cook Univ, Sch Publ Hlth Trop Med & Rehabil Sci, Townsville, Qld, Australia.

[Roelands, Bart] Fund Sci Res Flanders FWO, Brussels, Belgium.

RP Meeusen, R (corresponding author), Vrije Univ Brussel, Human Physiol Res Grp, Pl Laan 2, B-1050 Brussels, Belgium.

EM rmeeusen@vub.ac.be

RI Roelands, Bart/E-2337-2011; Marcora, Samuele/W-4284-2019; Van Cutsem,

Jeroen/AAZ-6281-2021; De Pauw, Kevin/E-8937-2018; Van Cutsem,

Jeroen/AAZ-7325-2021

OI Roelands, Bart/0000-0002-2808-044X; Marcora,

Samuele/0000-0002-1570-7936; De Pauw, Kevin/0000-0002-6901-7199; Buyse,

Luk/0000-0002-7163-8752; Van Cutsem, Jeroen/0000-0001-6122-7629

CR Blanchfield A, 2014, FRONT HUM NEUROSCI, V8, DOI 10.3389/fnhum.2014.00967

Boksem MAS, 2005, COGNITIVE BRAIN RES, V25, P107, DOI 10.1016/j.cogbrainres.2005.04.011

BORG GAV, 1982, MED SCI SPORT EXER, V14, P377, DOI 10.1249/00005768-198205000-00012

Brownsberger J, 2013, INT J SPORTS MED, V34, P1029, DOI 10.1055/s-0033-1343402

Coombes SA, 2012, CEREB CORTEX, V22, P616, DOI 10.1093/cercor/bhr141

De Pauw K, 2013, INT J SPORT PHYSIOL, V8, P111, DOI 10.1123/ijspp.8.2.111

Farrell MJ, 2011, J THERM BIOL, V36, P57, DOI 10.1016/j.jtherbio.2010.11.003

Folstein JR, 2008, PSYCHOPHYSIOLOGY, V45, P152, DOI 10.1111/j.1469-8986.2007.00602.x

Gajewski PD, 2008, BRAIN RES, V1189, P127, DOI 10.1016/j.brainres.2007.10.076

Gajewski PD, 2015, BRAIN COGNITION, V98, P87, DOI 10.1016/j.bandc.2015.06.004

Hopstaken JF, 2015, PSYCHOPHYSIOLOGY, V52, P305, DOI 10.1111/psyp.12339

Inzlicht M, 2014, TRENDS COGN SCI, V18, P127, DOI 10.1016/j.tics.2013.12.009

Jeukendrup A, 1996, MED SCI SPORT EXER, V28, P266, DOI 10.1097/00005768-199602000-00017

Kathner I, 2014, BIOL PSYCHOL, V102, P118, DOI 10.1016/j.biopsycho.2014.07.014

Lorist MM, 2009, BRAIN RES, V1270, P95, DOI 10.1016/j.brainres.2009.03.015

MacMahon C, 2014, J SPORT EXERCISE PSY, V36, P375, DOI 10.1123/jsep.2013-0249

Marcora SM, 2009, J APPL PHYSIOL, V106, P857, DOI 10.1152/japplphysiol.91324.2008

Martin K, 2016, PLOS ONE, V11, DOI 10.1371/journal.pone.0159907

Nybo L, 2001, J APPL PHYSIOL, V91, P2017, DOI 10.1152/jappl.2001.91.5.2017

Nybo L, 2001, J APPL PHYSIOL, V91, P1055, DOI 10.1152/jappl.2001.91.3.1055

Pageaux B, 2016, FRONT PHYSIOL, V7, DOI 10.3389/fphys.2016.00587

Pageaux B, 2015, FRONT HUM NEUROSCI, V9, DOI 10.3389/fnhum.2015.00067

Pageaux B, 2014, EUR J APPL PHYSIOL, V114, P1095, DOI 10.1007/s00421-014-2838-5

Pageaux B, 2013, MED SCI SPORT EXER, V45, P2254, DOI 10.1249/MSS.0b013e31829b504a

Parkin JM, 1999, J APPL PHYSIOL, V86, P902, DOI 10.1152/jappl.1999.86.3.902

Periard JD, 2011, EXP PHYSIOL, V96, P134, DOI 10.1113/expphysiol.2010.054213

Polich J, 2007, CLIN NEUROPHYSIOL, V118, P2128, DOI 10.1016/j.clinph.2007.04.019

Polich J, 2006, INT J PSYCHOPHYSIOL, V60, P172, DOI 10.1016/j.ijpsycho.2005.12.012

Qian SW, 2015, BEHAV BRAIN RES, V280, P6, DOI 10.1016/j.bbr.2014.11.036

RAMANATHAN NL, 1964, J APPL PHYSIOL, V19, P531, DOI 10.1152/jappl.1964.19.3.531

Sawka MN, 2012, EXP PHYSIOL, V97, P327, DOI [10.1113/expphysiol.2011.061026, 10.1113/expphysiol.2011.061002]

Smith MR, 2015, MED SCI SPORT EXER, V47, P1682, DOI 10.1249/MSS.0000000000000592

Smith MR, 2014, P 19 ANN C EUR COLL, P676

Van Cutsem J, 2017, SPORTS MED, V47, P1569, DOI 10.1007/s40279-016-0672-0

Van Cutsem J, 2015, EUR J APPL PHYSIOL, V115, P2135, DOI 10.1007/s00421-015-3193-x

Wascher E, 2014, BIOL PSYCHOL, V96, P57, DOI 10.1016/j.biopsycho.2013.11.010

Weiner KS, 2016, NEUROPSYCHOLOGIA, V83, P48, DOI 10.1016/j.neuropsychologia.2015.06.033

Weng TB, 2015, MED SCI SPORT EXER, V47, P1460, DOI 10.1249/MSS.0000000000000542

Williamson JW, 2001, J APPL PHYSIOL, V90, P1392, DOI 10.1152/jappl.2001.90.4.1392

Zenon A, 2015, J NEUROSCI, V35, P8737, DOI 10.1523/JNEUROSCI.3789-14.2015

NR 40

TC 27

Z9 27

U1 1

U2 53

PU LIPPINCOTT WILLIAMS & WILKINS

PI PHILADELPHIA

PA TWO COMMERCE SQ, 2001 MARKET ST, PHILADELPHIA, PA 19103 USA

SN 0195-9131

EI 1530-0315

J9 MED SCI SPORT EXER

JI Med. Sci. Sports Exerc.

PD AUG

PY 2017

VL 49

IS 8

BP 1677

EP 1687

DI 10.1249/MSS.0000000000001263

PG 11

WC Sport Sciences

WE Science Citation Index Expanded (SCI-EXPANDED); Social Science Citation Index (SSCI)

SC Sport Sciences

GA FA6KL

UT WOS:000405552000020

PM 28282326

OA Green Accepted

DA 2022-05-04

ER

PT J

AU Van Cutsem, J

Roelands, B

Pluym, B

Tassignon, B

Verschueren, J

De Pauw, K

Meeusen, R

AF Van Cutsem, Jeroen

Roelands, Bart

Pluym, Bert

Tassignon, Bruno

Verschueren, Jo

De Pauw, Kevin

Meeusen, Romain

TI Can Creatine Combat the Mental Fatigue-associated Decrease in Visuomotor

Skills?

SO MEDICINE AND SCIENCE IN SPORTS AND EXERCISE

LA English

DT Article

DE CREATINE SUPPLEMENTATION; PHOSPHOCREATINE; MENTAL EXERTION; COGNITIVE

FATIGUE; VISUOMOTOR RESPONSE TIME; COGNITIVE PERFORMANCE

ID SLEEP-DEPRIVATION; HUMAN BRAIN; SUPPLEMENTATION; PERFORMANCE;

INHIBITION; STRENGTH; EXERCISE; LACTATE

AB Purpose The importance of the brain in sports was recently confirmed by the negative effect of mental fatigue (MF) on sport-specific psychomotor skills. Creatine supplementation improves strength but can also improve cognitive functioning. To explore the role of creatine in combating MF, we evaluated whether creatine supplementation counteracts the MF-associated impairment in sport-specific psychomotor skills. Methods In 23 degrees C, 14 healthy participants (4 females, 10 males; mean +/- SD, age = 24 +/- 3 yr, mass = 74 +/- 13 kg, height = 179 +/- 9 cm) performed a 90-min mentally fatiguing task (counterbalanced, crossover, and double-blinded; i.e., Stroop task) in two different conditions: after a 7-d creatine supplementation (CR; 20 g center dot d(-1)) and after a 7-d calcium lactate supplementation (placebo [PLAC]), separated by a 5-wk washout. In both conditions, a 7-min sport-specific visuomotor task, a dynamic handgrip strength endurance task, and a 3-min Flanker task was performed before and after the mentally fatiguing task. Physiological and perceptual responses were measured throughout the protocol. Results Handgrip strength endurance was higher in CR compared with PLAC (P = 0.022). MF impaired visuomotor response time (+4.4%; P = 0.022) and Flanker accuracy (-5.0%; P = 0.009) in both conditions. Accuracy on the Stroop task was higher in CR compared with PLAC (+4.9%; P = 0.026). Within the perceptual and physiological parameters, only motivation and vigor (P <= 0.027) were lower in CR compared with PLAC. Conclusion Creatine supplementation improved physical (strength endurance) and prolonged cognitive (Stroop accuracy) performance, yet it did not combat MF-induced impairments in short sport-specific psychomotor or cognitive (Flanker) performance. These results warrant further investigation in the potential role of creatine in combating the MF-associated decrements in prolonged (e.g., 90-min soccer game) sport performance and suggest a role of brain phosphocreatine in MF.

C1 [Van Cutsem, Jeroen; Roelands, Bart; Pluym, Bert; Tassignon, Bruno; Verschueren, Jo; De Pauw, Kevin; Meeusen, Romain] Vrije Univ Brussel, Human Physiol & Sports Physiotherapy Res Grp, Pl Laan 2, B-1050 Brussels, Belgium.

RP Meeusen, R (corresponding author), Vrije Univ Brussel, Human Physiol & Sports Physiotherapy Res Grp, Pl Laan 2, B-1050 Brussels, Belgium.

EM romain.meeusen@vub.be

RI Van Cutsem, Jeroen/AAZ-7325-2021; Van Cutsem, Jeroen/AAZ-6281-2021; De

Pauw, Kevin/E-8937-2018; Roelands, Bart/E-2337-2011; Tassignon,

Bruno/M-8793-2017; Pluym, Bert/AAJ-8940-2020

OI Roelands, Bart/0000-0002-2808-044X; Tassignon,

Bruno/0000-0003-3216-4045; Pluym, Bert/0000-0003-2572-2707; Verschueren,

Jo/0000-0002-4971-3971; Van Cutsem, Jeroen/0000-0001-6122-7629; De Pauw,

Kevin/0000-0002-6901-7199

CR Brzezicka A, 2013, BEHAV BRAIN SCI, V36, P682, DOI 10.1017/S0140525X13000940

Campbell S., 2001, P HUMAN FACTORS ERGO, V45, P906, DOI DOI 10.1177/154193120104501302

Dechent P, 1999, AM J PHYSIOL-REG I, V277, pR698, DOI 10.1152/ajpregu.1999.277.3.R698

Dolan E, 2019, EUR J SPORT SCI, V19, P1, DOI 10.1080/17461391.2018.1500644

Ekelund U, 2006, PUBLIC HEALTH NUTR, V9, P258, DOI 10.1079/PHN2005840

Gerodimos Vassilis, 2017, J Hand Surg Am, V42, pe175, DOI 10.1016/j.jhsa.2016.12.014

HART S G, 1988, P139

Head J, 2012, CONSCIOUS COGN, V21, P1617, DOI 10.1016/j.concog.2012.08.009

Hopstaken JF, 2015, PSYCHOPHYSIOLOGY, V52, P305, DOI 10.1111/psyp.12339

Jones AM, 2009, AM J PHYSIOL-REG I, V296, pR1078, DOI 10.1152/ajpregu.90896.2008

Kurzban R, 2013, BEHAV BRAIN SCI, V36, P661, DOI 10.1017/S0140525X12003196

Lanhers C, 2017, SPORTS MED, V47, P163, DOI 10.1007/s40279-016-0571-4

Lovatt D, 2012, P NATL ACAD SCI USA, V109, P6265, DOI 10.1073/pnas.1120997109

MacLeod CM, 2000, TRENDS COGN SCI, V4, P383, DOI 10.1016/S1364-6613(00)01530-8

Marcora SM, 2009, J APPL PHYSIOL, V106, P857, DOI 10.1152/japplphysiol.91324.2008

Martin K, 2018, SPORTS MED, V48, P2041, DOI 10.1007/s40279-018-0946-9

McMorris T, 2007, PHYSIOL BEHAV, V90, P21, DOI 10.1016/j.physbeh.2006.08.024

McMorris T, 2006, PSYCHOPHARMACOLOGY, V185, P93, DOI 10.1007/s00213-005-0269-z

Mielgo-Ayuso J, 2019, NUTRIENTS, V11, DOI 10.3390/nu11040757

Pageaux B, 2014, EUR J APPL PHYSIOL, V114, P1095, DOI 10.1007/s00421-014-2838-5

Pageaux B, 2013, MED SCI SPORT EXER, V45, P2254, DOI 10.1249/MSS.0b013e31829b504a

Penna EM, 2018, PEDIATR EXERC SCI, V30, P208, DOI 10.1123/pes.2017-0128

Rae C, 2003, P ROY SOC B-BIOL SCI, V270, P2147, DOI 10.1098/rspb.2003.2492

Rae CD, 2015, NEUROCHEM INT, V89, P249, DOI 10.1016/j.neuint.2015.08.010

Rawson ES, 2011, AMINO ACIDS, V40, P1349, DOI 10.1007/s00726-011-0855-9

Russell S, 2019, J SCI MED SPORT, V22, P723, DOI 10.1016/j.jsams.2018.12.008

SAPPEYMARINIER D, 1992, J CEREBR BLOOD F MET, V12, P584, DOI 10.1038/jcbfm.1992.82

Shulman RG, 2001, NMR BIOMED, V14, P389, DOI 10.1002/nbm.741

Silva-Cavalcante MD, 2018, EUR J APPL PHYSIOL, V118, P2477, DOI 10.1007/s00421-018-3974-0

Smith MR, 2016, MED SCI SPORT EXER, V48, P267, DOI 10.1249/MSS.0000000000000762

Taylor IM, 2020, INT REV SPORT EXER P, V13, P1, DOI 10.1080/1750984X.2018.1480050

Turner CE, 2015, MAGN RESON IMAGING, V33, P1163, DOI 10.1016/j.mri.2015.06.018

Turner CE, 2015, J NEUROSCI, V35, P1773, DOI 10.1523/JNEUROSCI.3113-14.2015

Urbanski RL, 1999, INT J SPORT NUTR, V9, P136, DOI 10.1123/ijsn.9.2.136

Van Cutsem J, 2018, PSYCHOPHARMACOLOGY, V235, P947, DOI 10.1007/s00213-017-4809-0

Van Cutsem J, 2017, SPORTS MED, V47, P1569, DOI 10.1007/s40279-016-0672-0

Veness D, 2017, J SPORT SCI, V35, P2461, DOI 10.1080/02640414.2016.1273540

Wald FD., 1990, NEDERLANDS TIJDSCHRI, V45, P86

Watanabe A, 2002, NEUROSCI RES, V42, P279, DOI 10.1016/S0168-0102(02)00007-X

Young WB, 2002, J SPORT MED PHYS FIT, V42, P282

NR 40

TC 20

Z9 20

U1 8

U2 27

PU LIPPINCOTT WILLIAMS & WILKINS

PI PHILADELPHIA

PA TWO COMMERCE SQ, 2001 MARKET ST, PHILADELPHIA, PA 19103 USA

SN 0195-9131

EI 1530-0315

J9 MED SCI SPORT EXER

JI Med. Sci. Sports Exerc.

PD JAN

PY 2020

VL 52

IS 1

BP 120

EP 130

DI 10.1249/MSS.0000000000002122

PG 11

WC Sport Sciences

WE Science Citation Index Expanded (SCI-EXPANDED); Social Science Citation Index (SSCI)

SC Sport Sciences

GA KC0KO

UT WOS:000506876400014

PM 31403610

OA Bronze

DA 2022-05-04

ER

PT J

AU Brownsberger, J

Edwards, A

Crowther, R

Cottrell, D

AF Brownsberger, J.

Edwards, A.

Crowther, R.

Cottrell, D.

TI Impact of Mental Fatigue on Self-paced Exercise

SO INTERNATIONAL JOURNAL OF SPORTS MEDICINE

LA English

DT Article

DE mental fatigue; effort perception; brain regulation; pacing; EEG

ID PERCEIVED EXERTION; MOOD; HUMANS; MUSCLE; EEG; PERFORMANCE; VALIDITY;

RUNNERS; RATINGS

AB The purpose of this study was to examine whether mental fatigue influences the perceived effort required to complete fairly light and hard effort self-paced exercise challenges. 12 participants completed 2 trials in a randomised cross-over design. Each participant was required to complete a time-matched pre-exercise task: 1) a continuous cognitive activity test (EXP condition; n=12), or 2) a time-matched passive neutral observation task (CON condition; n=12). Following the pre-exercise task, participants performed 2 consecutive bouts of self-paced cycling exercise again in randomized order at fairly light (RPE 11) and hard (RPE 15) effort. Physiological, psychological and EEG indices were measured throughout both conditions. EXP participants reported significantly greater sensations of fatigue (p<0.01) and demonstrated greater EEG beta-band activation compared with CON (p<0.01) prior to exercise. Power outputs from the exercise bouts were significantly reduced for EXP in both self-paced: RPE 11 (83 +/- 7 vs. 99 +/- 7W; p=0.005) and RPE 15 (132 +/- 9 vs. 143 +/- 8W; p=0.028) trials. This study demonstrates that individuals with higher self-reported sensations of fatigue and elevations of EEG beta activity in the prefrontal cortex of the brain prior to exercise produce less work during self-paced exercise trials than in a control condition, probably due to an altered perception of effort.

C1 [Brownsberger, J.; Edwards, A.; Crowther, R.] James Cook Univ, Inst Sport & Exercise Sci, Cairns 4870, Australia.

[Cottrell, D.] James Cook Univ, Cairns 4870, Australia.

RP Edwards, A (corresponding author), James Cook Univ, Inst Sport & Exercise Sci, McGregor Rd, Cairns 4870, Australia.

EM andrew.edwards@jcu.edu.au

RI Cottrell, David/I-6811-2013

OI Cottrell, David/0000-0002-2405-6362

FU Institute of Sport & Exercise Science for incidental consumables and

minor costs

FX All authors contributed equally to planning, data collection, analysis

and assembly of this manuscript. The corresponding author is the team

leader and project supervisor. 2. This project was not subject to a

grant and was financially supported by discretionary budget of the

Institute of Sport & Exercise Science for incidental consumables and

minor costs. 3. The authors of this manuscript have no competing

interests.

CR Ahearn EP, 1997, J PSYCHIATR RES, V31, P569, DOI 10.1016/S0022-3956(97)00029-0

Bassett DR, 1997, MED SCI SPORT EXER, V29, P591, DOI 10.1097/00005768-199705000-00002

Berger BG, 2000, J APPL SPORT PSYCHOL, V12, P69, DOI 10.1080/10413200008404214

Boksem MAS, 2005, COGNITIVE BRAIN RES, V25, P107, DOI 10.1016/j.cogbrainres.2005.04.011

BORG GAV, 1982, MED SCI SPORT EXER, V14, P377, DOI 10.1249/00005768-198205000-00012

Chen MJ, 2002, J SPORT SCI, V20, P873, DOI 10.1080/026404102320761787

Crabbe JB, 2004, PSYCHOPHYSIOLOGY, V41, P563, DOI 10.1111/j.1469-8986.2004.00176.x

DUNBAR CC, 1992, MED SCI SPORT EXER, V24, P94

Edwards A M, 2012, PACING SPORT EXERCIS, P49

Edwards AM, 1999, INT J SPORTS MED, V20, P1, DOI 10.1055/s-2007-971082

Edwards AM, 2011, PSYCHOPHYSIOLOGY, V48, P136, DOI 10.1111/j.1469-8986.2010.01034.x

French DN, 2007, J APPL PHYSIOL, V102, P94, DOI 10.1152/japplphysiol.00586.2006

Goode JH, 2003, J SAFETY RES, V34, P309, DOI 10.1016/S0022-4375(03)00033-1

Harriss DJ, 2011, INT J SPORTS MED, V32, P819, DOI 10.1055/s-0031-1287829

kerstedt T, 2004, CHRONOBIOL INT, V21, P1055

Lambert EV, 2005, BRIT J SPORT MED, V39, P52, DOI 10.1136/bjsm.2003.011247

Lander PJ, 2009, BRIT J SPORT MED, V43, P789, DOI 10.1136/bjsm.2008.056085

Lorist MM, 2000, PSYCHOPHYSIOLOGY, V37, P614, DOI 10.1111/1469-8986.3750614

Lorist MM, 2009, BRAIN RES, V1270, P95, DOI 10.1016/j.brainres.2009.03.015

Marcora SM, 2009, J APPL PHYSIOL, V106, P857, DOI 10.1152/japplphysiol.91324.2008

Marcora SM, 2010, EUR J APPL PHYSIOL, V109, P763, DOI 10.1007/s00421-010-1418-6

Miller R, 2007, INT J PSYCHOPHYSIOL, V64, P18, DOI 10.1016/j.ijpsycho.2006.07.009

Moraes H, 2007, ARQ NEURO-PSIQUIAT, V65, P637, DOI 10.1590/S0004-282X2007000400018

Nielsen B, 2001, PFLUG ARCH EUR J PHY, V442, P41, DOI 10.1007/s004240100515

Noakes TD, 2004, J APPL PHYSIOL, V96, P1571, DOI 10.1152/japplphysiol.01124.2003

Noakes TD, 2011, APPL PHYSIOL NUTR ME, V36, P23, DOI 10.1139/H10-082

Schneider S, 2009, PHYSIOL BEHAV, V96, P709, DOI 10.1016/j.physbeh.2009.01.007

Swart J, 2009, BRIT J SPORT MED, V43, P782, DOI 10.1136/bjsm.2008.055889

Watt MJ, 2003, J PHYSIOL-LONDON, V550, P325, DOI 10.1113/jphysiol.2003.043133

Yeung RR, 1996, J PSYCHOSOM RES, V40, P123, DOI 10.1016/0022-3999(95)00554-4

NR 30

TC 66

Z9 66

U1 1

U2 43

PU GEORG THIEME VERLAG KG

PI STUTTGART

PA RUDIGERSTR 14, D-70469 STUTTGART, GERMANY

SN 0172-4622

EI 1439-3964

J9 INT J SPORTS MED

JI Int. J. Sports Med.

PD DEC

PY 2013

VL 34

IS 12

BP 1029

EP 1036

DI 10.1055/s-0033-1343402

PG 8

WC Sport Sciences

WE Science Citation Index Expanded (SCI-EXPANDED)

SC Sport Sciences

GA 259SV

UT WOS:000327547500002

PM 23771830

DA 2022-05-04

ER

PT J

AU Blomstrand, E

AF Blomstrand, E

TI Amino acids and central fatigue

SO AMINO ACIDS

LA English

DT Article

DE amino acids; branched-chain amino acids; central fatigue; performance

ID PLASMA-FREE TRYPTOPHAN; ENDURANCE PERFORMANCE; CARBOHYDRATE FEEDINGS;

PROLONGED EXERCISE; SUSTAINED EXERCISE; PHYSICAL EXERCISE; MUSCLE; 5-HT;

5-HYDROXYTRYPTAMINE; SUPPLEMENTATION

AB There is an increasing interest in the mechanisms behind central fatigue, particularly in relation tb changes in brain monoamine metabolism and the influence of specific amino acids on fatigue. Several studies in experimental animals have shown that physical exercise increases the synthesis and metabolism of brain 5-hydroxytryptamine (5-HT). Support for the involvement of 5-HT in fatigue can be found in studies where the brain concentration of 5-HT has been altered by means of pharmacological agents. When the 5-HT level was elevated in this way the performance was impaired in both rats and human subjects, and in accordance with this a decrease in the 5-HT level caused an improvement in running performance in rats. The precursor of 5-HT is the amino acid tryptophan and the synthesis of 5-HT in the brain is thought to be regulated by the blood supply of free tryptophan in relation to other large neutral amino acids (including the branched-chain amino acids, BCAA) since these compete with tryptophan for transport into the brain. Studies in human subjects have shown that the plasma ratio of free tryptophan/BCAA increases during and, particularly, after sustained exercise. This would favour the transport of tryptophan into the brain and also the synthesis and release of S-HT which may lead to central fatigue. Attempts have been made to influence the 5-HT level by giving BCAA to human subjects during different types of sustained heavy exercise. The results indicate that ingestion of BCAA reduces the perceived exertion and mental fatigue during exercise and improves cognitive performance after the exercise. In addition, in some situations ingestion of BCAA might also improve physical performance; during exercise in the heat or in a competitive race when the central component of fatigue is assumed to be more pronounced than in a laboratory experiment. However, more experiments are needed to further clarify the effect of BCAA and also of tryptophan ingestion on physical performance and mental fatigue.

C1 Stockholm Univ, Coll Phys Educ & Sports, Dept Hlth & Sport Sci, Stockholm, Sweden.

Karolinska Inst, Dept Physiol & Pharmacol, Stockholm, Sweden.

RP Blomstrand, E (corresponding author), Stockholm Univ, Coll Phys Educ & Sports, Dept Hlth & Sport Sci, Stockholm, Sweden.

CR ASTRAND PO, 1986, TXB WORK PHYSL, P118

BAILEY SP, 1992, ACTA PHYSIOL SCAND, V145, P75, DOI 10.1111/j.1748-1716.1992.tb09338.x

BAILEY SP, 1993, J APPL PHYSIOL, V74, P3006, DOI 10.1152/jappl.1993.74.6.3006

BARCHAS JD, 1963, BIOCHEM PHARMACOL, V12, P1232, DOI 10.1016/0006-2952(63)90101-1

Blomstrand E, 1997, ACTA PHYSIOL SCAND, V159, P41, DOI 10.1046/j.1365-201X.1997.547327000.x

BLOMSTRAND E, 1988, ACTA PHYSIOL SCAND, V133, P115, DOI 10.1111/j.1748-1716.1988.tb08388.x

BLOMSTRAND E, 1991, EUR J APPL PHYSIOL, V63, P83, DOI 10.1007/BF00235174

BLOMSTRAND E, 1989, ACTA PHYSIOL SCAND, V136, P473, DOI 10.1111/j.1748-1716.1989.tb08689.x

BLOMSTRAND E, 1995, ACTA PHYSIOL SCAND, V153, P87, DOI 10.1111/j.1748-1716.1995.tb09839.x

BLOMSTRAND E, UNPUB BRANCHED CHAIN

Calders P, 1997, MED SCI SPORT EXER, V29, P1182, DOI 10.1097/00005768-199709000-00010

Calders P, 1999, MED SCI SPORT EXER, V31, P583, DOI 10.1097/00005768-199904000-00015

CHAOULOFF F, 1989, J NEURAL TRANSM-GEN, V78, P121, DOI 10.1007/BF01252498

CHAOULOFF F, 1986, J NEUROCHEM, V46, P1647, DOI 10.1111/j.1471-4159.1986.tb01789.x

CHAOULOFF F, 1989, ACTA PHYSIOL SCAND, V137, P1, DOI 10.1111/j.1748-1716.1989.tb08715.x

CURZON G, 1973, NATURE, V242, P198, DOI 10.1038/242198a0

DAVIS JM, 1992, EUR J APPL PHYSIOL, V65, P513, DOI 10.1007/BF00602357

Davis JM, 1999, INT J SPORTS MED, V20, P309, DOI 10.1055/s-2007-971136

Farris JW, 1998, J APPL PHYSIOL, V85, P807, DOI 10.1152/jappl.1998.85.3.807

FERNSTROM JD, 1990, J NUTR BIOCHEM, V1, P508, DOI 10.1016/0955-2863(90)90033-H

HASSMEN P, 1994, NUTRITION, V10, P405

HENRIKSSON J, 1991, J EXP BIOL, V160, P149

KIRBY LG, 1995, BRAIN RES, V682, P189, DOI 10.1016/0006-8993(95)00349-U

LEHMANN M, 1995, INT J SPORTS MED, V16, P155, DOI 10.1055/s-2007-972984

MACLEAN DA, 1994, AM J PHYSIOL-ENDOC M, V267, pE1010, DOI 10.1152/ajpendo.1994.267.6.E1010

MACLEAN DA, 1993, J APPL PHYSIOL, V74, P2711, DOI 10.1152/jappl.1993.74.6.2711

Madsen K, 1996, J APPL PHYSIOL, V81, P2644, DOI 10.1152/jappl.1996.81.6.2644

McConell G, 1999, J APPL PHYSIOL, V87, P1083, DOI 10.1152/jappl.1999.87.3.1083

MCMENAMY RH, 1958, J BIOL CHEM, V233, P1436

Meeusen R, 1996, BRAIN RES, V740, P245, DOI 10.1016/S0006-8993(96)00872-4

Mittleman KD, 1998, MED SCI SPORT EXER, V30, P83, DOI 10.1097/00005768-199801000-00012

NEWSHOLME EA, 1986, ACTA PHYSIOL SCAND, V128, P93

NEWSHOLME EA, 1983, BIOCH MED SCI, P339

PARDRIDGE WM, 1977, J NEUROCHEM, V28, P103, DOI 10.1111/j.1471-4159.1977.tb07714.x

Struder HK, 1997, CAN J APPL PHYSIOL, V22, P280, DOI 10.1139/h97-019

Struder HK, 1999, EUR J APPL PHYSIOL O, V79, P318, DOI 10.1007/s004210050514

VANHALL G, 1995, J PHYSIOL-LONDON, V486, P789, DOI 10.1113/jphysiol.1995.sp020854

VARNIER M, 1994, EUR J APPL PHYSIOL O, V69, P26, DOI 10.1007/BF00867923

VERGER PH, 1994, PHYSIOL BEHAV, V3, P523

WAGENMAKERS AJM, 1992, MED SPORT SCI, V34, P69

WIDRICK JJ, 1993, J APPL PHYSIOL, V74, P2998, DOI 10.1152/jappl.1993.74.6.2998

WILSON WM, 1992, EXP PHYSIOL, V77, P921, DOI 10.1113/expphysiol.1992.sp003660

WRIGHT DA, 1991, J APPL PHYSIOL, V71, P1082, DOI 10.1152/jappl.1991.71.3.1082

NR 43

TC 96

Z9 105

U1 1

U2 26

PU SPRINGER-VERLAG

PI NEW YORK

PA 175 FIFTH AVE, NEW YORK, NY 10010 USA

SN 0939-4451

J9 AMINO ACIDS

JI Amino Acids

PY 2001

VL 20

IS 1

BP 25

EP 34

DI 10.1007/s007260170063

PG 10

WC Biochemistry & Molecular Biology

WE Science Citation Index Expanded (SCI-EXPANDED)

SC Biochemistry & Molecular Biology

GA 404WE

UT WOS:000167124400004

PM 11310928

DA 2022-05-04

ER

PT J

AU Esposito, F

Otto, T

Zijlstra, FRH

Goebel, R

AF Esposito, Fabrizio

Otto, Tobias

Zijlstra, Fred R. H.

Goebel, Rainer

TI Spatially Distributed Effects of Mental Exhaustion on Resting-State FMRI

Networks

SO PLOS ONE

LA English

DT Article

ID INDEPENDENT COMPONENT ANALYSIS; BOLD SIGNAL FLUCTUATIONS; FUNCTIONAL

CONNECTIVITY; SUSTAINED ATTENTION; ASL PERFUSION; ALPHA-BAND; BASE-LINE;

ACTIVATION; FATIGUE; PERFORMANCE

AB Brain activity during rest is spatially coherent over functional connectivity networks called resting-state networks. In restingstate functional magnetic resonance imaging, independent component analysis yields spatially distributed network representations reflecting distinct mental processes, such as intrinsic (default) or extrinsic (executive) attention, and sensory inhibition or excitation. These aspects can be related to different treatments or subjective experiences. Among these, exhaustion is a common psychological state induced by prolonged mental performance. Using repeated functional magnetic resonance imaging sessions and spatial independent component analysis, we explored the effect of several hours of sustained cognitive performances on the resting human brain. Resting-state functional magnetic resonance imaging was performed on the same healthy volunteers in two days, with and without, and before, during and after, an intensive psychological treatment (skill training and sustained practice with a flight simulator). After each scan, subjects rated their level of exhaustion and performed an N-back task to evaluate eventual decrease in cognitive performance. Spatial maps of selected resting-state network components were statistically evaluated across time points to detect possible changes induced by the sustained mental performance. The intensive treatment had a significant effect on exhaustion and effort ratings, but no effects on N-back performances. Significant changes in the most exhausted state were observed in the early visual processing and the anterior default mode networks (enhancement) and in the fronto-parietal executive networks (suppression), suggesting that mental exhaustion is associated with a more idling brain state and that internal attention processes are facilitated to the detriment of more extrinsic processes. The described application may inspire future indicators of the level of fatigue in the neural attention system.

C1 [Esposito, Fabrizio] Univ Salerno, Dept Med & Surg, I-84100 Salerno, Italy.

[Esposito, Fabrizio; Goebel, Rainer] Maastricht Univ, Dept Cognit Neurosci, Maastricht, Netherlands.

[Otto, Tobias; Zijlstra, Fred R. H.] Maastricht Univ, Dept Work & Social Psychol, Maastricht, Netherlands.

[Goebel, Rainer] Netherlands Inst Neurosci, Dept Vis & Cognit, Amsterdam, Netherlands.

RP Esposito, F (corresponding author), Univ Salerno, Dept Med & Surg, I-84100 Salerno, Italy.

EM faesposito@unisa.it

RI Esposito, Fabrizio/M-8555-2015

OI Esposito, Fabrizio/0000-0002-5099-9786; Goebel,

Rainer/0000-0003-1780-2467; Zijlstra, Fred/0000-0002-3505-9753

CR Ackerman PL, 2009, J EXP PSYCHOL-APPL, V15, P163, DOI 10.1037/a0015719

Albert NB, 2009, CURR BIOL, V19, P1023, DOI 10.1016/j.cub.2009.04.028

Allen EA, 2011, FRONT SYST NEUROSCI, V5, DOI 10.3389/fnsys.2011.00002

Barnes A, 2009, PLOS ONE, V4, DOI 10.1371/journal.pone.0006626

BARTLETT EJ, 1988, J CEREBR BLOOD F MET, V8, P502, DOI 10.1038/jcbfm.1988.91

Birn RM, 2008, HUM BRAIN MAPP, V29, P740, DOI 10.1002/hbm.20577

BISWAL B, 1995, MAGNET RESON MED, V34, P537, DOI 10.1002/mrm.1910340409

Boksem MAS, 2006, BIOL PSYCHOL, V72, P123, DOI 10.1016/j.biopsycho.2005.08.007

Cabeza R, 2000, J COGNITIVE NEUROSCI, V12, P1, DOI 10.1162/08989290051137585

Calhoun VD, 2009, NEUROIMAGE, V45, pS163, DOI 10.1016/j.neuroimage.2008.10.057

Colby CL, 1996, COGNITIVE BRAIN RES, V5, P105, DOI 10.1016/S0926-6410(96)00046-8

Corbetta M, 2002, NAT REV NEUROSCI, V3, P201, DOI 10.1038/nrn755

CORBETTA M, 1993, J NEUROSCI, V13, P1202

Coull JT, 1998, NEUROPSYCHOLOGIA, V36, P1325, DOI 10.1016/S0028-3932(98)00035-9

Cox RW, 1996, COMPUT BIOMED RES, V29, P162, DOI 10.1006/cbmr.1996.0014

Damoiseaux JS, 2006, P NATL ACAD SCI USA, V103, P13848, DOI 10.1073/pnas.0601417103

De Luca M, 2006, NEUROIMAGE, V29, P1359, DOI 10.1016/j.neuroimage.2005.08.035

Deichmann R, 2003, NEUROIMAGE, V19, P430, DOI 10.1016/S1053-8119(03)00073-9

Demeter E, 2011, NEUROIMAGE, V54, P1518, DOI 10.1016/j.neuroimage.2010.09.026

Duff EP, 2008, HUM BRAIN MAPP, V29, P778, DOI 10.1002/hbm.20601

Duncan J, 2010, TRENDS COGN SCI, V14, P172, DOI 10.1016/j.tics.2010.01.004

Esposito F, 2005, NEUROIMAGE, V25, P193, DOI 10.1016/j.neuroimage.2004.10.042

Esposito F, 2013, BRAIN, V136, P710, DOI 10.1093/brain/awt007

Esposito F, 2011, CURR OPIN NEUROL, V24, P378, DOI 10.1097/WCO.0b013e32834897a5

Esposito F, 2010, NEUROIMAGE, V53, P534, DOI 10.1016/j.neuroimage.2010.06.061

Fan J, 2005, NEUROIMAGE, V26, P471, DOI 10.1016/j.neuroimage.2005.02.004

FORMAN SD, 1995, MAGNET RESON MED, V33, P636, DOI 10.1002/mrm.1910330508

FRISTON KJ, 1995, NEUROIMAGE, V2, P45, DOI 10.1006/nimg.1995.1007

GEVINS A, 1993, ELECTROEN CLIN NEURO, V87, P128, DOI 10.1016/0013-4694(93)90119-G

Glover GH, 2000, MAGNET RESON MED, V44, P162, DOI 10.1002/1522-2594(200007)44:1<162::AID-MRM23>3.0.CO;2-E

Goebel R, 2006, HUM BRAIN MAPP, V27, P392, DOI 10.1002/hbm.20249

Gordon EM, 2012, HUMAN BRAIN MAPPING

Greicius MD, 2004, P NATL ACAD SCI USA, V101, P4637, DOI 10.1073/pnas.0308627101

Greicius MD, 2008, HUM BRAIN MAPP, V29, P839, DOI 10.1002/hbm.20537

Grigg O, 2010, PLOS ONE, V5, DOI 10.1371/journal.pone.0013311

Gusnard DA, 2001, NAT REV NEUROSCI, V2, P685, DOI 10.1038/35094500

HACKER W, 1978, ERGONOMICS, V21, P187, DOI 10.1080/00140137808931712

Hockey GRJ, 1997, BIOL PSYCHOL, V45, P73

Hyvarinen A, 1999, IEEE T NEURAL NETWOR, V10, P626, DOI 10.1109/72.761722

Hyvarinen A, 2001, INDEPENDENT COMPONENT ANALYSIS: PRINCIPLES AND PRACTICE, P71

Kamei T, 1998, PERCEPT MOTOR SKILL, V87, P1419, DOI 10.2466/pms.1998.87.3f.1419

Khalili-Mahani N., 2011, HUM BRAIN MAPP

Kim J, 2006, NEUROIMAGE, V31, P376, DOI 10.1016/j.neuroimage.2005.11.035

Koric L, 2011, HUM BRAIN MAPP

Langdon DW, 2011, CURR OPIN NEUROL, V24, P244, DOI 10.1097/WCO.0b013e328346a43b

Lee HL, 2013, NEUROIMAGE, V65, P216, DOI 10.1016/j.neuroimage.2012.10.015

Lewin JS, 1996, J COMPUT ASSIST TOMO, V20, P695, DOI 10.1097/00004728-199609000-00002

Lewis JW, 2011, HUM BRAIN MAPP, V32, P2241, DOI 10.1002/hbm.21185

Licata SC, 2013, NEUROIMAGE, V70, P211, DOI 10.1016/j.neuroimage.2012.12.055

Lim JL, 2013, NEUROIMAGE, V76, P81, DOI 10.1016/j.neuroimage.2013.03.018

Lim J, 2010, NEUROIMAGE, V49, P3426, DOI 10.1016/j.neuroimage.2009.11.020

Mantini D, 2007, P NATL ACAD SCI USA, V104, P13170, DOI 10.1073/pnas.0700668104

McKeown MJ, 1998, HUM BRAIN MAPP, V6, P160, DOI 10.1002/(SICI)1097-0193(1998)6:3<160::AID-HBM5>3.3.CO;2-R

Nobre AC, 1997, BRAIN, V120, P515, DOI 10.1093/brain/120.3.515

O'Hara RB, 2010, METHODS ECOL EVOL, V1, P118, DOI 10.1111/j.2041-210X.2010.00021.x

OGAWA S, 1990, P NATL ACAD SCI USA, V87, P9868, DOI 10.1073/pnas.87.24.9868

Ogg RJ, 2008, MAGN RESON IMAGING, V26, P504, DOI 10.1016/j.mri.2007.09.004

Pfurtscheller G, 1996, INT J PSYCHOPHYSIOL, V24, P39, DOI 10.1016/S0167-8760(96)00066-9

Schrauf M., 2011, P HUMAN FACTORS ERGO, DOI [10.1177/1071181311551045, DOI 10.1177/1071181311551045]

Smith SM, 2009, P NATL ACAD SCI USA, V106, P13040, DOI 10.1073/pnas.0905267106

Soares JM, 2013, PLOS ONE, V8, DOI 10.1371/journal.pone.0066500

Sonnentag S, 2006, J APPL PSYCHOL, V91, P330, DOI 10.1037/0021-9010.91.2.330

Tamaki M, 1999, PSYCHIAT CLIN NEUROS, V53, P273, DOI 10.1046/j.1440-1819.1999.00548.x

Vahdat S, 2011, J NEUROSCI, V31, P16907, DOI 10.1523/JNEUROSCI.2737-11.2011

Valdez P, 2008, MIND BRAIN EDUC, V2, P7, DOI 10.1111/j.1751-228X.2008.00023.x

van de Ven VG, 2004, HUM BRAIN MAPP, V22, P165, DOI 10.1002/hbm.20022

van de Ven V, 2009, NEUROIMAGE, V47, P1982, DOI 10.1016/j.neuroimage.2009.05.057

van den Heuvel MP, 2009, HUM BRAIN MAPP, V30, P3127, DOI 10.1002/hbm.20737

van der Linden D, 2003, ACTA PSYCHOL, V113, P45, DOI 10.1016/S0001-6918(02)00150-6

Waites AB, 2005, HUM BRAIN MAPP, V24, P59, DOI 10.1002/hbm.20069

Worden MS, 2000, J NEUROSCI, V20

Zhou YX, 2012, RADIOLOGY, V265, P882, DOI 10.1148/radiol.12120748

Zijlstra FRH, 1993, EFFICIENCY WORK BEHA

NR 73

TC 27

Z9 27

U1 0

U2 11

PU PUBLIC LIBRARY SCIENCE

PI SAN FRANCISCO

PA 1160 BATTERY STREET, STE 100, SAN FRANCISCO, CA 94111 USA

SN 1932-6203

J9 PLOS ONE

JI PLoS One

PD APR 4

PY 2014

VL 9

IS 4

AR e94222

DI 10.1371/journal.pone.0094222

PG 13

WC Multidisciplinary Sciences

WE Science Citation Index Expanded (SCI-EXPANDED)

SC Science & Technology - Other Topics

GA AE6MJ

UT WOS:000334107500134

PM 24705397

OA Green Published, Green Submitted, gold

DA 2022-05-04

ER

PT J

AU Machek, SB

Bagley, JR

AF Machek, Steven B.

Bagley, James R.

TI Creatine Monohydrate Supplementation: Considerations for Cognitive

Performance in Athletes

SO STRENGTH AND CONDITIONING JOURNAL

LA English

DT Article

DE ergogenic aids; resistance training; cognitive enhancement; mental

fatigue; athletic performance; nootropics

ID HIGH-INTENSITY EXERCISE; MENTAL FATIGUE; SLEEP-DEPRIVATION;

DOUBLE-BLIND; HUMAN BRAIN; MUSCLE; STRENGTH; MALES; BODY; ACCUMULATION

AB Creatine monohydrate supplementation can increase total creatine and phosphocreatine stores for resynthesis of adenosine triphosphate. Although most existing literature has investigated creatine to improve strength and body composition, it has also been shown to promote brain energy homeostasis and improve cognitive parameters. This may be another mechanism for performance enhancement because exercise is both physically and mentally depleting. This article aims to (a) reinforce the efficacy of creatine supplementation in athletes, (b) showcase creatine's role as a cognitive enhancer, and (c) establish the need for future interventions in creatine's effect as a comprehensive ergogenic aid (combining physical and cognitive benefits).

C1 [Machek, Steven B.; Bagley, James R.] San Francisco State Univ, Coll Hlth & Social Sci, Dept Kinesiol, San Francisco, CA 94132 USA.

RP Bagley, JR (corresponding author), San Francisco State Univ, Coll Hlth & Social Sci, Dept Kinesiol, San Francisco, CA 94132 USA.

EM jrbagley@sfsu.edu

OI Machek, Steven/0000-0003-3544-0666

CR Antonio J, 2013, J INT SOC SPORT NUTR, V10, DOI 10.1186/1550-2783-10-36

Azizi M, 2011, PROCD SOC BEHV, V15, P1626, DOI 10.1016/j.sbspro.2011.03.342

Backx EMP, 2017, SPORTS MED, V47, P1661, DOI 10.1007/s40279-016-0670-2

Baddeley A, 1998, Q J EXP PSYCHOL-A, V51, P819, DOI 10.1080/027249898391413

Beal MF, 2011, AMINO ACIDS, V40, P1305, DOI 10.1007/s00726-011-0851-0

Benton D, 2011, BRIT J NUTR, V105, P1100, DOI 10.1017/S0007114510004733

Bonilla DA, 2015, REV COLOMB QUIM, V44, P11, DOI 10.15446/rev.colomb.quim.v44n1.53978

Branch JD, 2003, INT J SPORT NUTR EXE, V13, P198, DOI 10.1123/ijsnem.13.2.198

Buford TW, 2007, J INT SOC SPORT NUTR, V4, DOI 10.1186/1550-2783-4-6

Burke LM, 1996, INT J SPORT NUTR, V6, P222, DOI 10.1123/ijsn.6.3.222

Chwalbinska-Moneta J, 2003, INT J SPORT NUTR EXE, V13, P173, DOI 10.1123/ijsnem.13.2.173

Claudino JG, 2014, J INT SOC SPORT NUTR, V11, DOI 10.1186/1550-2783-11-32

Colcombe S, 2003, PSYCHOL SCI, V14, P125, DOI 10.1111/1467-9280.t01-1-01430

Cook CJ, 2011, J INT SOC SPORT NUTR, V8, DOI 10.1186/1550-2783-8-2

Cooke MB, 2014, EUR J APPL PHYSIOL, V114, P1321, DOI 10.1007/s00421-014-2866-1

Cooper R, 2012, J INT SOC SPORT NUTR, V9, DOI 10.1186/1550-2783-9-33

Dechent P, 1999, AM J PHYSIOL-REG I, V277, pR698, DOI 10.1152/ajpregu.1999.277.3.R698

Ekkekakis P, 2006, INT J OBESITY, V30, P652, DOI 10.1038/sj.ijo.0803052

Ekkekakis P, 2003, COGNITION EMOTION, V17, P213, DOI 10.1080/02699930302292

Engl E, 2015, J NEUROSCI, V35, P9249, DOI 10.1523/JNEUROSCI.1195-15.2015

Gee TI, 2016, SPORTS NUTR THER, V1, P1, DOI [10.4172/2473-6449.1000115, DOI 10.4172/2473-6449.1000115]

GORDON A, 1995, CARDIOVASC RES, V30, P413, DOI 10.1016/0008-6363(95)00062-3

Gouttebarge V, 2012, EUR J SPORT SCI, V2, P33

Green AL, 1996, AM J PHYSIOL-ENDOC M, V271, pE821, DOI 10.1152/ajpendo.1996.271.5.E821

Gualano B, 2008, AMINO ACIDS, V34, P245, DOI 10.1007/s00726-007-0508-1

HARRIS RC, 1992, CLIN SCI, V83, P367, DOI 10.1042/cs0830367

Hultman E, 1996, J APPL PHYSIOL, V81, P232

Jones L, 2014, J SPORT EXERCISE PSY, V36, P528, DOI [10.1123/jsep.2014-0251, 10.1123/jsep.2013-0251]

Juhasz I, 2009, ACTA PHYSIOL HUNG, V96, P325, DOI 10.1556/APhysiol.96.2009.3.6

Kilduff LP, 2004, INT J SPORT NUTR EXE, V14, P443, DOI 10.1123/ijsnem.14.4.443

Klopstock T, 2011, AMINO ACIDS, V40, P1297, DOI 10.1007/s00726-011-0850-1

Kocak S, 2003, J SPORT MED PHYS FIT, V43, P488

Kolpakova M E, 2013, Ross Fiziol Zh Im I M Sechenova, V99, P889

Kondo DG, 2016, AMINO ACIDS, V48, P1941, DOI 10.1007/s00726-016-2194-3

Kreider RB, 2003, MOL CELL BIOCHEM, V244, P89, DOI 10.1023/A:1022465203458

Kreider RB, 2017, J INT SOC SPORT NUTR, V14, DOI 10.1186/s12970-017-0173-z

Lanhers C, 2015, SPORTS MED, V45, P1285, DOI 10.1007/s40279-015-0337-4

Ling J, 2009, BEHAV PHARMACOL, V20, P673, DOI 10.1097/FBP.0b013e3283323c2a

Lyoo IK, 2012, AM J PSYCHIAT, V169, P937, DOI 10.1176/appi.ajp.2012.12010009

Mashiko T, 2004, BRIT J SPORT MED, V38, P617, DOI 10.1136/bjsm.2003.007690

McMorris T, 2007, PHYSIOL BEHAV, V90, P21, DOI 10.1016/j.physbeh.2006.08.024

McMorris T, 2006, PSYCHOPHARMACOLOGY, V185, P93, DOI 10.1007/s00213-005-0269-z

McMorris T, 2007, AGING NEUROPSYCHOL C, V14, P517, DOI 10.1080/13825580600788100

McNaughton LR, 1998, EUR J APPL PHYSIOL O, V78, P236, DOI 10.1007/s004210050413

Pan JW, 2007, AM J PHYSIOL-REG I, V292, pR1745, DOI 10.1152/ajpregu.00717.2006

Persky AM, 2001, PHARMACOL REV, V53, P161

Peyrebrune MC, 2005, MED SCI SPORT EXER, V37, P2140, DOI 10.1249/01.mss.0000179101.38913.73

Powers ME, 2003, J ATHL TRAINING, V38, P44

Rae C, 2003, P ROY SOC B-BIOL SCI, V270, P2147, DOI 10.1098/rspb.2003.2492

Rae CD, 2015, NEUROCHEM INT, V89, P249, DOI 10.1016/j.neuint.2015.08.010

Rawson ES, 2003, J STRENGTH COND RES, V17, P822

Roitman S, 2007, BIPOLAR DISORD, V9, P754, DOI 10.1111/j.1399-5618.2007.00532.x

Rossouw F, 2000, NUTR RES, V317, P25

Santos RVT, 2004, LIFE SCI, V75, P1917, DOI 10.1016/j.lfs.2003.11.036

Schlattner U, 2006, BBA-MOL BASIS DIS, V1762, P164, DOI 10.1016/j.bbadis.2005.09.004

Smith MR, 2016, MED SCI SPORT EXER, V48, P267, DOI 10.1249/MSS.0000000000000762

Stanton R, 2000, J STRENGTH COND RES, V14, P322

Steenge GR, 1998, AM J PHYSIOL-ENDOC M, V275, pE974, DOI 10.1152/ajpendo.1998.275.6.E974

STOCKLER S, 1994, PEDIATR RES, V36, P409

Stout J, 2000, J APPL PHYSIOL, V88, P109, DOI 10.1152/jappl.2000.88.1.109

Tarnopolsky MA, 2000, INT J SPORT NUTR EXE, V10, P452, DOI 10.1123/ijsnem.10.4.452

Terjung RL, 2000, MED SCI SPORT EXER, V32, P706, DOI 10.1097/00005768-200003000-00024

Trexler ET, 2015, INT J SPORT NUTR EXE, V25, P607, DOI 10.1123/ijsnem.2014-0193

Turner CE, 2015, J NEUROSCI, V35, P1773, DOI 10.1523/JNEUROSCI.3113-14.2015

Twycross-Lewis R, 2016, AMINO ACIDS, V48, P1843, DOI 10.1007/s00726-016-2237-9

Urbanski RL, 1999, INT J SPORT NUTR, V9, P136, DOI 10.1123/ijsn.9.2.136

Vagnozzi R, 2013, J HEAD TRAUMA REHAB, V28, P284, DOI 10.1097/HTR.0b013e3182795045

Volek JS, 1997, J AM DIET ASSOC, V97, P765, DOI 10.1016/S0002-8223(97)00189-2

Wallimann T, 2011, AMINO ACIDS, V40, P1271, DOI 10.1007/s00726-011-0877-3

Watanabe A, 2002, NEUROSCI RES, V42, P279, DOI 10.1016/S0168-0102(02)00007-X

Zajac A, 2001, J STRENGTH COND RES, V15, P357, DOI 10.1519/00124278-200108000-00017

NR 71

TC 2

Z9 2

U1 2

U2 19

PU LIPPINCOTT WILLIAMS & WILKINS

PI PHILADELPHIA

PA TWO COMMERCE SQ, 2001 MARKET ST, PHILADELPHIA, PA 19103 USA

SN 1524-1602

EI 1533-4295

J9 STRENGTH COND J

JI Strength Cond. J.

PD APR

PY 2018

VL 40

IS 2

BP 82

EP 93

DI 10.1519/SSC.0000000000000369

PG 12

WC Sport Sciences

WE Science Citation Index Expanded (SCI-EXPANDED); Social Science Citation Index (SSCI)

SC Sport Sciences

GA GO7NT

UT WOS:000440256400009

DA 2022-05-04

ER

PT J

AU Blomstrand, E

AF Blomstrand, E

TI A role for branched-chain amino acids in reducing central fatigue

SO JOURNAL OF NUTRITION

LA English

DT Article; Proceedings Paper

CT Symposium on Branched-Chain Amino Acids in Exercise held at the Annual

Meeting of the International-Society-for-Sports-Nutrition

CY JUN 17, 2005

CL New Orleans, LA

SP Amino Vital Sports Sci Fdn, Int Soc Sports Nutr

DE branched-chain amino acids; tryptophan; exercise; 5-hydroxytryptamine;

central fatigue

ID L-TRYPTOPHAN SUPPLEMENTATION; PLASMA-FREE TRYPTOPHAN; PROLONGED

EXERCISE; SUSTAINED EXERCISE; 5-HYDROXYTRYPTAMINE METABOLISM;

5-HYDROXYINDOLEACETIC ACID; ENDURANCE PERFORMANCE; EXTRACELLULAR 5-HT;

BRAIN TRYPTOPHAN; WARM ENVIRONMENT

AB Several factors have been identified to cause peripheral fatigue during exercise, whereas the mechanisms behind central fatigue are less well known. Changes in the brain 5-hydroxytryptamine (5-HT) level is one factor that has been suggested to cause fatigue. The rate-limiting step in the synthesis of 5-HT is the transport of tryptophan across the blood-brain barrier. This transport is influenced by the fraction of tryptophan available for transport into the brain and the concentration of the other large neutral amino acids, including the BCAAs (leucine, isoleucine, and valine), which are transported via the same carrier system. Studies in human subjects have shown that the plasma ratio of free tryptophan (unbound to albumin)/BCAAs increases and that tryptophan is taken up by the brain during endurance exercise, suggesting that this may increase the synthesis of 5-HT in the brain. Ingestion of BCAAs increases their concentration in plasma. This may reduce the uptake of tryptophan by the brain and also 5-HT synthesis and thereby delay fatigue. Accordingly, when BCAAs were supplied to human subjects during a standardized cycle ergometer exercise their ratings of perceived exertion and mental fatigue were reduced, and, during a competitive 30-km cross-country race, their performance on different cognitive tests was improved after the race. In some situations the intake of BCAAs also improves physical performance. The results also suggest that ingestion of carbohydrates during exercise delays a possible effect of BCAAs on fatigue since the brain's uptake of tryptophan is reduced.

C1 Univ Stockholm, Coll Phys Educ & Sports, Astrand Lab, S-10691 Stockholm, Sweden.

Karolinska Inst, Dept Physiol & Pharmacol, S-10401 Stockholm, Sweden.

RP Blomstrand, E (corresponding author), Univ Stockholm, Coll Phys Educ & Sports, Astrand Lab, S-10691 Stockholm, Sweden.

EM eva.blomstrand@gih.se

CR Astrand P.-O., 2003, TXB WORK PHYSL

BAILEY SP, 1992, ACTA PHYSIOL SCAND, V145, P75, DOI 10.1111/j.1748-1716.1992.tb09338.x

BAILEY SP, 1993, INT J SPORTS MED, V14, P330, DOI 10.1055/s-2007-1021187

BARCHAS JD, 1963, BIOCHEM PHARMACOL, V12, P1232, DOI 10.1016/0006-2952(63)90101-1

Blomstrand E, 2005, ACTA PHYSIOL SCAND, V185, P203, DOI 10.1111/j.1365-201X.2005.01482.x

Blomstrand E, 1997, ACTA PHYSIOL SCAND, V159, P41, DOI 10.1046/j.1365-201X.1997.547327000.x

BLOMSTRAND E, 1988, ACTA PHYSIOL SCAND, V133, P115, DOI 10.1111/j.1748-1716.1988.tb08388.x

BLOMSTRAND E, 1991, EUR J APPL PHYSIOL, V63, P83, DOI 10.1007/BF00235174

Blomstrand E, 2001, AM J PHYSIOL-ENDOC M, V281, pE365, DOI 10.1152/ajpendo.2001.281.2.E365

BLOMSTRAND E, 1989, ACTA PHYSIOL SCAND, V136, P473, DOI 10.1111/j.1748-1716.1989.tb08689.x

BLOMSTRAND E, 1995, ACTA PHYSIOL SCAND, V153, P87, DOI 10.1111/j.1748-1716.1995.tb09839.x

CHAOULOFF F, 1987, NEUROPHARMACOLOGY, V26, P1099, DOI 10.1016/0028-3908(87)90254-1

CHAOULOFF F, 1986, J NEUROCHEM, V46, P1647, DOI 10.1111/j.1471-4159.1986.tb01789.x

Chaouloff F, 1997, MED SCI SPORT EXER, V29, P58, DOI 10.1097/00005768-199701000-00009

CHAOULOFF F, 1986, J NEUROCHEM, V46, P1313, DOI 10.1111/j.1471-4159.1986.tb00656.x

CHAOULOFF F, 1985, BRIT J PHARMACOL, V86, P33, DOI 10.1111/j.1476-5381.1985.tb09432.x

Cheuvront SN, 2004, J APPL PHYSIOL, V97, P1275, DOI 10.1152/japplphysiol.00357.2004

Christensen EH, 1939, SKAND ARCH PHYSIOL, V81, P172, DOI 10.1111/j.1748-1716.1939.tb01321.x

COYLE EF, 1983, J APPL PHYSIOL, V55, P230, DOI 10.1152/jappl.1983.55.1.230

CURZON G, 1973, NATURE, V242, P198, DOI 10.1038/242198a0

DAVIS JM, 1992, EUR J APPL PHYSIOL, V65, P513, DOI 10.1007/BF00602357

Davis JM, 1999, INT J SPORTS MED, V20, P309, DOI 10.1055/s-2007-971136

Dwyer D, 2002, EXP PHYSIOL, V87, P83, DOI 10.1113/eph8702176

Farris JW, 1998, J APPL PHYSIOL, V85, P807, DOI 10.1152/jappl.1998.85.3.807

Fernstrom JD, 2005, J NUTR, V135, p1539S, DOI 10.1093/jn/135.6.1539S

FERNSTROM JD, 1978, J NEUROCHEM, V30, P1531, DOI 10.1111/j.1471-4159.1978.tb10489.x

FERNSTROM JD, 1972, SCIENCE, V178, P414, DOI 10.1126/science.178.4059.414

Gomez-Merino D, 2001, NEUROSCI LETT, V301, P143, DOI 10.1016/S0304-3940(01)01626-3

Gomez-Merino D, 2001, INT J SPORTS MED, V22, P317, DOI 10.1055/s-2001-15645

HASSMEN P, 1994, NUTRITION, V10, P405

JAKEMAN PM, 1994, EXP PHYSIOL, V79, P461, DOI 10.1113/expphysiol.1994.sp003780

KIRBY LG, 1995, BRAIN RES, V682, P189, DOI 10.1016/0006-8993(95)00349-U

MACLEAN DA, 1994, AM J PHYSIOL-ENDOC M, V267, pE1010, DOI 10.1152/ajpendo.1994.267.6.E1010

MACLEAN DA, 1993, J APPL PHYSIOL, V74, P2711, DOI 10.1152/jappl.1993.74.6.2711

Meeusen R, 2003, ADV EXP MED BIOL, V527, P521

Meeusen R, 1996, BRAIN RES, V740, P245, DOI 10.1016/S0006-8993(96)00872-4

Mittleman KD, 1998, MED SCI SPORT EXER, V30, P83, DOI 10.1097/00005768-199801000-00012

Newsholme E.A., 1987, ADV MYOCHEMISTRY, P127

Newsholme EA, 1983, BIOCH MED SCI, P784

Nybo L, 2003, ACTA PHYSIOL SCAND, V179, P67, DOI 10.1046/j.1365-201X.2003.01175.x

Nybo L, 2004, PROG NEUROBIOL, V72, P223, DOI 10.1016/j.pneurobio.2004.03.005

Nybo L, 2003, J APPL PHYSIOL, V95, P1125, DOI 10.1152/japplphysiol.00241.2003

Pardridge WM, 1998, NEUROCHEM RES, V23, P635, DOI 10.1023/A:1022482604276

Pitsiladis YP, 2002, EXP PHYSIOL, V87, P215, DOI 10.1113/eph8702342

SATO Y, 1981, CLIN PHYSIOL, V1, P151, DOI 10.1111/j.1475-097X.1981.tb00883.x

SEGURA R, 1988, INT J SPORTS MED, V9, P301, DOI 10.1055/s-2007-1025027

SOARES DD, 2002, PHARM BIOCH BEHAV, V74, P357

STENSRUD T, 1992, INT J SPORTS MED, V13, P481, DOI 10.1055/s-2007-1021302

Strachan AT, 2004, EXP PHYSIOL, V89, P657, DOI 10.1113/expphysiol.2004.027839

Strachan AT, 1999, MED SCI SPORT EXER, V31, P547, DOI 10.1097/00005768-199904000-00009

Struder HK, 1997, CAN J APPL PHYSIOL, V22, P280, DOI 10.1139/h97-019

Struder HK, 1998, HORM METAB RES, V30, P188, DOI 10.1055/s-2007-978864

VANHALL G, 1995, J PHYSIOL-LONDON, V486, P789, DOI 10.1113/jphysiol.1995.sp020854

VARNIER M, 1994, EUR J APPL PHYSIOL O, V69, P26, DOI 10.1007/BF00867923

WAGENMAKERS AJM, 1992, MED SPORT SCI, V34, P69

Watson P, 2004, EUR J APPL PHYSIOL, V93, P306, DOI 10.1007/s00421-004-1206-2

Wilson WM, 1996, BEHAV PHARMACOL, V7, P101

WILSON WM, 1992, EXP PHYSIOL, V77, P921, DOI 10.1113/expphysiol.1992.sp003660

YOUNG SN, 1986, NUTR BRAIN, P49

NR 59

TC 128

Z9 139

U1 1

U2 33

PU AMER SOCIETY NUTRITIONAL SCIENCE

PI BETHESDA

PA 9650 ROCKVILLE PIKE, RM L-2407A, BETHESDA, MD 20814 USA

SN 0022-3166

J9 J NUTR

JI J. Nutr.

PD FEB

PY 2006

VL 136

IS 2

BP 544S

EP 547S

DI 10.1093/jn/136.2.544S

PG 4

WC Nutrition & Dietetics

WE Conference Proceedings Citation Index - Science (CPCI-S); Science Citation Index Expanded (SCI-EXPANDED)

SC Nutrition & Dietetics

GA 006JU

UT WOS:000234894600036

PM 16424144

OA Bronze

DA 2022-05-04

ER

PT J

AU Ramos, G

Vaz, JR

Mendonca, GV

Pezarat-Correia, P

Rodrigues, J

Alfaras, M

Gamboa, H

AF Ramos, G.

Vaz, J. R.

Mendonca, G. V.

Pezarat-Correia, P.

Rodrigues, J.

Alfaras, M.

Gamboa, H.

TI Fatigue Evaluation through Machine Learning and a Global Fatigue

Descriptor

SO JOURNAL OF HEALTHCARE ENGINEERING

LA English

DT Article

ID HEART-RATE-VARIABILITY; MUSCLE FATIGUE; LACTATE THRESHOLD; MUSCULAR

FATIGUE; EXERCISE; FOREST

AB Research in physiology and sports science has shown that fatigue, a complex psychophysiological phenomenon, has a relevant impact in performance and in the correct functioning of our motricity system, potentially being a cause of damage to the human organism. Fatigue can be seen as a subjective or objective phenomenon. Subjective fatigue corresponds to a mental and cognitive event, while fatigue referred as objective is a physical phenomenon. Despite the fact that subjective fatigue is often undervalued, only a physically and mentally healthy athlete is able to achieve top performance in a discipline. Therefore, we argue that physical training programs should address the preventive assessment of both subjective and objective fatigue mechanisms in order to minimize the risk of injuries. In this context, our paper presents a machine-learning system capable of extracting individual fatigue descriptors (IFDs) from electromyographic (EMG) and heart rate variability (HRV) measurements. Our novel approach, using two types of biosignals so that a global (mental and physical) fatigue assessment is taken into account, reflects the onset of fatigue by implementing a combination of a dimensionless (0-1) global fatigue descriptor (GFD) and a support vector machine (SVM) classifier. The system, based on 9 main combined features, achieves fatigue regime classification performances of 0.82 +/- 0.24, ensuring a successful preventive assessment when dangerous fatigue levels are reached. Training data were acquired in a constant work rate test (executed by 14 subjects using a cycloergometry device), where the variable under study (fatigue) gradually increased until the volunteer reached an objective exhaustion state.

C1 [Ramos, G.; Alfaras, M.] PLUX Wireless Biosignals SA, Ave 5 Outubro, P-105059 Lisbon, Portugal.

[Vaz, J. R.] Univ Nebraska, Dept Biomech, Omaha, NE 68182 USA.

[Vaz, J. R.] Univ Nebraska, Ctr Res Human Movement Variabil, Omaha, NE 68182 USA.

[Vaz, J. R.] Laureate Int Univ, Univ Europeia, Lisbon, Portugal.

[Vaz, J. R.; Mendonca, G. V.; Pezarat-Correia, P.] Univ Lisbon, Fac Human Kinet, CIPER, Neuromuscular Res Lab, Lisbon, Portugal.

[Rodrigues, J.; Gamboa, H.] NOVA Univ Lisbon, Fac Sci & Technol, Lab Instrumentat Biomed Engn & Radiat Phy LIBPhys, Caparica, Portugal.

[Alfaras, M.] Univ Jaume 1, Castellon de La Plana, Spain.

[Gamboa, H.] NOVA Univ Lisbon, Fac Sci & Technol, Dept Phys, Caparica, Portugal.

RP Ramos, G (corresponding author), PLUX Wireless Biosignals SA, Ave 5 Outubro, P-105059 Lisbon, Portugal.

EM gramos@plux.info

RI Vaz, João R/AAH-7314-2019; Gamboa, Hugo/M-8799-2013; Pezarat-Correia,

Pedro/O-1661-2019

OI Vaz, João R/0000-0001-9691-5456; Gamboa, Hugo/0000-0002-4022-7424;

Pezarat-Correia, Pedro/0000-0001-6154-5563; Vilhena de Mendonca,

Goncalo/0000-0001-8161-8598; Ramos, Guilherme/0000-0001-6041-7834;

Rodrigues, Joao Manuel/0000-0001-7320-511X; Alfaras,

Miquel/0000-0002-8942-5843

FU Fundacao para a Ciencia e Tecnologia (FCT)Portuguese Foundation for

Science and TechnologyEuropean Commission [PTDC/DTP-DES/5714/2014];

University of Nebraska at Omaha Office of Research and Creative

Activity; AHAAmerican Heart Association [CMUP-ERI/HCI/0046]; ITN

AffecTech, under the Marie Sklodowska Curie Actions (ERC H2020 Project)

[722022]; FCTPortuguese Foundation for Science and TechnologyEuropean

Commission; [NIH-P20GM109090]

FX The authors thank Ms. Carolina Teodosio for assistance during the data

collection. The acquired data were collected within the projected

PTDC/DTP-DES/5714/2014-Contralateral effects of low intensity resistance

training combined with blood flow restriction, funded by Fundacao para a

Ciencia e Tecnologia (FCT), and J. R. Vaz was supported by

NIH-P20GM109090 and by the University of Nebraska at Omaha Office of

Research and Creative Activity. Joao Rodrigues participates in

"iNOVA4Health-Programme in Translational Medicine" with a FCT grant I&D

2015-2020, while Guilherme Ramos was supported in the first phase by AHA

CMUP-ERI/HCI/0046.The authors acknowledge the support that Miquel

Alfaras received from ITN AffecTech, under the Marie Sklodowska Curie

Actions (ERC H2020 Project ID: 722022).

CR Acharya UR, 2006, MED BIOL ENG COMPUT, V44, P1031, DOI 10.1007/s11517-006-0119-0

Ament W, 2009, SPORTS MED, V39, P389, DOI 10.2165/00007256-200939050-00005

[Anonymous], 2015, TECH REP

Becker BJ, 2007, STAT SCI, V22, P414, DOI 10.1214/07-STS243

Benoit CE, 2019, NEUROPSYCHOLOGIA, V123, P30, DOI 10.1016/j.neuropsychologia.2018.06.017

BIGLANDRITCHIE B, 1984, MUSCLE NERVE, V7, P691, DOI 10.1002/mus.880070902

Bigliassi M., 2014, APPL MATH, V05, P1878, DOI DOI 10.4236/am.2014.513181

Binder RK, 2008, EUR J CARDIOV PREV R, V15, P726, DOI 10.1097/HJR.0b013e328304fed4

Bonato P, 2001, IEEE T BIO-MED ENG, V48, P745, DOI 10.1109/10.930899

Borenstein M, 2010, RES SYNTH METHODS, V1, P97, DOI 10.1002/jrsm.12

Borresen J, 2009, SPORTS MED, V39, P779, DOI 10.2165/11317780-000000000-00000

Camata TV, 2011, J STRENGTH COND RES, V25, P2537, DOI 10.1519/JSC.0b013e318202e6a0

Camm AJ, 1996, EUR HEART J, V17, P354

Cifrek M, 2009, CLIN BIOMECH, V24, P327, DOI 10.1016/j.clinbiomech.2009.01.010

CORTES C, 1995, MACH LEARN, V20, P273, DOI 10.1023/A:1022627411411

Cottin FO, 2004, MED SCI SPORT EXER, V36, P594, DOI 10.1249/01.MSS.0000121982.14718.2A

da Silva R A, 2008, Electromyogr Clin Neurophysiol, V48, P147

Dantas JL, 2010, IEEE ENG MED BIO, P5979, DOI 10.1109/IEMBS.2010.5627579

DE LUCA CJ, 1984, CRIT REV BIOMED ENG, V11, P251

de Mendonca GV, 2017, EUR J APPL PHYSIOL, V117, P1373, DOI 10.1007/s00421-017-3627-8

ENOKA RM, 1995, J ELECTROMYOGR KINES, V5, P141, DOI 10.1016/1050-6411(95)00010-W

Enoka RM, 2016, MED SCI SPORT EXER, V48, P2228, DOI 10.1249/MSS.0000000000000929

Faller L, 2009, REV BRAS FISIOTER, V13, P422, DOI 10.1590/S1413-35552009005000057

Farina D, 2006, EXERC SPORT SCI REV, V34, P121, DOI 10.1249/00003677-200607000-00006

Faude O, 2009, SPORTS MED, V39, P469, DOI 10.2165/00007256-200939060-00003

Graham RB, 2015, PLOS ONE, V10, DOI 10.1371/journal.pone.0135069

Granitto PM, 2006, CHEMOMETR INTELL LAB, V83, P83, DOI 10.1016/j.chemolab.2006.01.007

Gruet M, 2013, NEUROSCIENCE, V231, P384, DOI 10.1016/j.neuroscience.2012.10.058

Hearst MA, 1998, IEEE INTELL SYST APP, V13, P18, DOI 10.1109/5254.708428

Hermens HJ, 2000, J ELECTROMYOGR KINES, V10, P361, DOI 10.1016/S1050-6411(00)00027-4

HOWLEY ET, 1995, MED SCI SPORT EXER, V27, P1292

Ioannis D., 2014, ACTA MED MARTINIANA, V14, P5, DOI [10.1515/acm-2015-0001., DOI 10.1515/ACM-2015-0001]

Kim HG, 2018, PSYCHIAT INVEST, V15, P235, DOI 10.30773/pi.2017.08.17

Kimura T, 2008, EUR J APPL PHYSIOL, V104, P651, DOI 10.1007/s00421-008-0816-5

Knaflitz M, 1999, J ELECTROMYOGR KINES, V9, P337, DOI 10.1016/S1050-6411(99)00009-7

Koumarianou A., 2015, J CLIN CASE REPORTS, V5, P1

Lewis S, 2001, BMJ-BRIT MED J, V322, P1479, DOI 10.1136/bmj.322.7300.1479

MacIsaac D, 2001, J ELECTROMYOGR KINES, V11, P439, DOI 10.1016/S1050-6411(01)00021-9

Makivic B., 2013, J EXERCISE PHYSL ONL, V16, P103

Mourot L, 2014, INT J SPORT PHYSIOL, V9, P695, DOI [10.1123/ijspp.2013-0286, 10.1123/IJSPP.2013-0286]

Larrauri JM, 2013, CARDIOTECHNIX: PROCEEDINGS OF THE INTERNATIONAL CONGRESS ON CARDIOVASCULAR TECHNOLOGIES, P107, DOI 10.5220/0004666501070114

OTSU N, 1979, IEEE T SYST MAN CYB, V9, P62, DOI 10.1109/TSMC.1979.4310076

Pageaux B., 2018, SPORT BRAIN SCI PR C, V240

PAN J, 1985, IEEE T BIO-MED ENG, V32, P230, DOI 10.1109/TBME.1985.325532

Patel M, 2011, EXPERT SYST APPL, V38, P7235, DOI 10.1016/j.eswa.2010.12.028

Phinyomark A, 2011, MEAS SCI REV, V11, P45, DOI 10.2478/v10048-011-0009-y

Pichon AP, 2004, MED SCI SPORT EXER, V36, P1702, DOI 10.1249/01.MSS.0000142403.93205.35

Pimentel A, 2015, INTERACT COMPUT, V27, P492, DOI 10.1093/iwc/iwv008

Rodrigues J, 2017, COMPUT BIOL MED, V87, P322, DOI 10.1016/j.compbiomed.2017.06.009

Rouffet DM, 2008, J ELECTROMYOGR KINES, V18, P866, DOI 10.1016/j.jelekin.2007.03.008

Rozzi SL, 1999, J ATHL TRAINING, V34, P106

Sarmiento S, 2013, J SYST SCI COMPLEX, V26, P104, DOI 10.1007/s11424-013-2287-y

Schmitt L, 2015, FRONT PHYSIOL, V6, DOI 10.3389/fphys.2015.00343

Shi J, 2007, MED ENG PHYS, V29, P472, DOI 10.1016/j.medengphy.2006.07.004

Taelman J, 2011, ADV EXP MED BIOL, V701, P353, DOI 10.1007/978-1-4419-7756-4_48

Thongpanja S, 2013, ELEKTRON ELEKTROTECH, V19, P51, DOI 10.5755/j01.eee.19.3.3697

Tonnessen E, 2014, PLOS ONE, V9, DOI 10.1371/journal.pone.0101796

Tran Y, 2009, J PSYCHOPHYSIOL, V23, P143, DOI 10.1027/0269-8803.23.3.143

Vitor-Costa Marcelo, 2012, Rev. bras. cineantropom. desempenho hum., V14, P660, DOI 10.5007/1980-0037.2012v14n6p660

Wan JJ, 2017, EXP MOL MED, V49, DOI 10.1038/emm.2017.194

WASSERMAN K, 1973, J APPL PHYSIOL, V35, P236, DOI 10.1152/jappl.1973.35.2.236

Whelton Paul K, 2018, J Am Soc Hypertens, V12, DOI 10.1016/j.jash.2018.06.010

YOSHITAKE H, 1978, ERGONOMICS, V21, P231, DOI 10.1080/00140137808931718

Zwarts MJ, 2008, CLIN NEUROPHYSIOL, V119, P2, DOI 10.1016/j.clinph.2007.09.126

NR 64

TC 5

Z9 5

U1 5

U2 31

PU HINDAWI LTD

PI LONDON

PA ADAM HOUSE, 3RD FLR, 1 FITZROY SQ, LONDON, W1T 5HF, ENGLAND

SN 2040-2295

EI 2040-2309

J9 J HEALTHC ENG

JI J. Healthc. Eng.

PD JAN 7

PY 2020

VL 2020

AR 6484129

DI 10.1155/2020/6484129

PG 18

WC Health Care Sciences & Services

WE Science Citation Index Expanded (SCI-EXPANDED); Social Science Citation Index (SSCI)

SC Health Care Sciences & Services

GA KE2JA

UT WOS:000508383300001

PM 31998469

OA Green Published, gold

DA 2022-05-04

ER

PT J

AU O'Keeffe, K

Dean, J

Hodder, S

Lloyd, A

AF O'Keeffe, Kate

Dean, Jacob

Hodder, Simon

Lloyd, Alex

TI Self-Selected Motivational Music Enhances Physical Performance in

Normoxia and Hypoxia in Young Healthy Males

SO FRONTIERS IN PSYCHOLOGY

LA English

DT Article

DE music; hypoxia; physical performance; motivation; combined stressors

ID COGNITIVE PERFORMANCE; EXERCISE PERFORMANCE; TWITCH INTERPOLATION; SPORT

PERFORMANCE; MOUNT EVEREST; FATIGUE; RESPONSES; ANXIETY; IMPACT;

VALIDATION

AB Humans exposed to hypoxia are susceptible to physiological and psychological impairment. Music has ergogenic effects through enhancing psychological factors such as mood, emotion, and cognition. This study aimed to investigate music as a tool for mitigating the performance decrements observed in hypoxia. Thirteen males (mean +/- SD; 24 +/- 4 years) completed one familiarization session and four experimental trials; (1) normoxia (sea level, 0.209 FiO(2)) and no music; (2) normoxia (0.209 FiO(2)) with music; (3) normobaric hypoxia (similar to 3800 m, 0.13 FiO(2)) and no music; and (4) normobaric hypoxia (0.13 FiO(2)) with music. Experimental trials were completed at 21 degrees C with 50% relative humidity. Music was self-selected prior to the familiarization session. Each experimental trial included a 15-min time trial on an arm bike, followed by a 60-s isometric maximal voluntary contraction (MVC) of the biceps brachii. Supramaximal nerve stimulation quantified central and peripheral fatigue with voluntary activation (VA%) calculated using the doublet interpolation method. Average power output (W) was reduced with a main effect of hypoxia (p = 0.02) and significantly increased with a main effect of music (p = 0.001). When combined the interaction was additive (p = 0.87). Average MVC force (N) was reduced in hypoxia (p = 0.03) but VA% of the biceps brachii was increased with music (p = 0.02). Music reduced subjective scores of mental effort, breathing discomfort, and arm discomfort in hypoxia (p < 0.001). Music increased maximal physical exertion through enhancing neural drive and diminishing detrimental mental processes, enhancing performance in normoxia (6.3%) and hypoxia (6.4%).

C1 [O'Keeffe, Kate; Dean, Jacob; Hodder, Simon; Lloyd, Alex] Loughborough Univ, Environm Ergon Res Ctr, Loughborough, Leics, England.

RP Lloyd, A (corresponding author), Loughborough Univ, Environm Ergon Res Ctr, Loughborough, Leics, England.

EM a.lloyd@lboro.ac.uk

CR Abraini JH, 1998, PFLUG ARCH EUR J PHY, V436, P553, DOI 10.1007/s004240050671

Amann M, 2008, J APPL PHYSIOL, V104, P861, DOI 10.1152/japplphysiol.01008.2007

Barwood MJ, 2008, MED SCI SPORT EXER, V40, P387, DOI 10.1249/mss.0b013e31815adf31

Bigliassi M, 2016, PHYSIOL BEHAV, V158, P128, DOI 10.1016/j.physbeh.2016.03.001

Boos CJ, 2018, PLOS ONE, V13, DOI 10.1371/journal.pone.0197147

Borg G., 1998, BORGS PERCEIVED EXER

Bradbury KE, 2019, J APPL PHYSIOL, V127, P513, DOI 10.1152/japplphysiol.00023.2019

Cote IM, 2016, P ROY SOC B-BIOL SCI, V283, DOI 10.1098/rspb.2015.2592

Crust L, 2006, J SPORT SCI, V24, P187, DOI 10.1080/02640410500131514

Downey AE, 2007, RESP PHYSIOL NEUROBI, V156, P137, DOI 10.1016/j.resp.2006.08.006

Edworthy J, 2006, ERGONOMICS, V49, P1597, DOI 10.1080/00140130600899104

Elliott, 2007, EUROPEAN J SPORT SCI, V5, P97, DOI [DOI 10.1080/17461390500171310, 10.1080/17461390500171310]

English T, 2019, PHYSIOL BEHAV, V208, DOI 10.1016/j.physbeh.2019.112567

Fagenholz PJ, 2007, WILD ENVIRON MED, V18, P312, DOI 10.1580/07-WEME-BR-102R1.1

Fan JL, 2016, HIGH ALT MED BIOL, V17, P72, DOI 10.1089/ham.2016.0034

Faul F, 2007, BEHAV RES METHODS, V39, P175, DOI 10.3758/BF03193146

Feldman G, 2013, J RES PERS, V47, P111, DOI 10.1016/j.jrp.2012.10.001

Folland JP, 2007, J ELECTROMYOGR KINES, V17, P317, DOI 10.1016/j.jelekin.2006.04.008

Fulco CS, 1998, AVIAT SPACE ENVIR MD, V69, P793

Gaoua N, 2012, J ENVIRON PSYCHOL, V32, P158, DOI 10.1016/j.jenvp.2012.01.002

Goodall S, 2012, J PHYSIOL-LONDON, V590, P2767, DOI 10.1113/jphysiol.2012.228890

Hatzigeorgiadis A, 2018, J APPL SPORT PSYCHOL, V30, P388, DOI 10.1080/10413200.2017.1395930

Herbert RD, 1999, J NEUROPHYSIOL, V82, P2271, DOI 10.1152/jn.1999.82.5.2271

Humberstone-Gough CE, 2013, J SPORT SCI MED, V12, P394

Hutchinson MJ, 2020, J SCI MED SPORT, V23, P403, DOI 10.1016/j.jsams.2019.10.012

Karageorghis CI, 2018, SCAND J MED SCI SPOR, V28, P1166, DOI 10.1111/sms.12979

Karageorghis CI, 2006, J SPORT SCI, V24, P899, DOI 10.1080/02640410500298107

Karageorghis CI, 2012, INT REV SPORT EXER P, V5, P44, DOI 10.1080/1750984X.2011.631026

Karageorghis CI, 2012, INT REV SPORT EXER P, V5, P67, DOI 10.1080/1750984X.2011.631027

Komiyama T, 2017, SCI REP-UK, V7, DOI 10.1038/s41598-017-10332-y

Lane A., 2009, E J APPL PSYCHOL, V5, DOI [10.7790/ejap.v5i1.123, DOI 10.7790/EJAP.V5I1.123]

Lane AM, 2004, J SPORT SCI, V22, P886, DOI 10.1080/02640410400005875

Lefferts WK, 2016, PHYSIOL BEHAV, V165, P108, DOI 10.1016/j.physbeh.2016.07.003

Lloyd Alex, 2016, Temperature (Austin), V3, P514, DOI 10.1080/23328940.2016.1189991

Lloyd A, 2016, J APPL PHYSIOL, V120, P567, DOI 10.1152/japplphysiol.00876.2015

Lloyd A, 2015, EUR J APPL PHYSIOL, V115, P2007, DOI 10.1007/s00421-015-3181-1

Lowe M, 2007, ARCH CLIN NEUROPSYCH, V22, pS89, DOI 10.1016/j.acn.2006.10.010

Mammarella N, 2007, AGING CLIN EXP RES, V19, P394

Martin K, 2019, HUM FACTORS, V61, P1205, DOI 10.1177/0018720819839817

Matthews G., 2013, PSYCHOL STRESS NEW R, V1, P49, DOI DOI 10.1016/J.FOODRES.2014.12.027

McMorris T, 2017, NEUROSCI BIOBEHAV R, V74, P225, DOI 10.1016/j.neubiorev.2017.01.019

MERTON PA, 1954, J PHYSIOL-LONDON, V123, P553, DOI 10.1113/jphysiol.1954.sp005070

Millet GY, 2012, J APPL PHYSIOL, V112, P1335, DOI 10.1152/japplphysiol.00804.2011

Mitropoulos A, 2017, J SPORT SCI MED, V16, P558

Muggeridge DJ, 2013, INT J SPORT NUTR EXE, V23, P498, DOI 10.1123/ijsnem.23.5.498

O'Keeffe K, 2021, J SPORT SCI, V39, P1687, DOI 10.1080/02640414.2021.1896104

Raglin JS, 2001, SPORTS MED, V31, P875, DOI 10.2165/00007256-200131120-00004

REJESKI WJ, 1985, J SPORT EXERCISE PSY, V7, P371, DOI 10.1123/jsp.7.4.371

Robertson EY, 2010, MED SCI SPORT EXER, V42, P394, DOI 10.1249/MSS.0b013e3181b34b57

Sarkamo T, 2013, WIRES COGN SCI, V4, P441, DOI 10.1002/wcs.1237

Scheeren TWL, 2012, J CLIN MONIT COMPUT, V26, P279, DOI 10.1007/s10877-012-9348-y

Shield A, 2004, SPORTS MED, V34, P253, DOI 10.2165/00007256-200434040-00005

Shukitt-Hale B., 1993, BEHAV COGN FUNCT SPO, V16, P97, DOI [10.2165/00007256-199316020-00003, DOI 10.2165/00007256-199316020-00003]

Stavrou NAM, 2015, PHYSIOL BEHAV, V139, P497, DOI 10.1016/j.physbeh.2014.12.015

Taylor L, 2016, FRONT PHYSIOL, V6, DOI 10.3389/fphys.2015.00372

Tenenbaum G, 2004, PSYCHOL SPORT EXERC, V5, P89, DOI 10.1016/S1469-0292(02)00041-9

Terry PC, 2003, PSYCHOL SPORT EXERC, V4, P125, DOI 10.1016/S1469-0292(01)00035-8

Terry PC, 2020, PSYCHOL BULL, V146, P91, DOI 10.1037/bul0000216

Vanhatalo A, 2011, J PHYSIOL-LONDON, V589, P5517, DOI 10.1113/jphysiol.2011.216341

Virues-Ortega J, 2006, DEVELOPMENTAL SCI, V9, P400, DOI 10.1111/j.1467-7687.2006.00505.x

Virues-Ortega Javier, 2004, Neuropsychology Review, V14, P197

Williams TB, 2019, EXP PHYSIOL, V104, P1384, DOI 10.1113/EP087647

Woodman T, 2003, J SPORT SCI, V21, P443, DOI 10.1080/0264041031000101809

NR 63

TC 0

Z9 0

U1 0

U2 0

PU FRONTIERS MEDIA SA

PI LAUSANNE

PA AVENUE DU TRIBUNAL FEDERAL 34, LAUSANNE, CH-1015, SWITZERLAND

SN 1664-1078

J9 FRONT PSYCHOL

JI Front. Psychol.

PD DEC 10

PY 2021

VL 12

AR 787496

DI 10.3389/fpsyg.2021.787496

PG 12

WC Psychology, Multidisciplinary

WE Social Science Citation Index (SSCI)

SC Psychology

GA YI0JM

UT WOS:000743543700001

PM 34956012

OA gold, Green Published

DA 2022-05-04

ER

PT J

AU Chabal, S

Welles, R

Haran, FJ

Markwald, R

AF Chabal, Sarah

Welles, Rebecca

Haran, F. Jay

Markwald, Rachel

TI Effects of sleep and fatigue on teams in a submarine environment

SO UNDERSEA AND HYPERBARIC MEDICINE

LA English

DT Article

DE submariner; fatigue; circadian rhythms

ID HUMAN CIRCADIAN SYSTEM; INDIVIDUAL-DIFFERENCES; DECISION-MAKING; SHIFT

WORK; SUBSEQUENT RECOVERY; PEER RESPONSES; MENTAL FATIGUE; ONE NIGHT;

PERFORMANCE; DEPRIVATION

AB Successful submarine operations rely on the performance of tactical teams who must work under conditions of physiological and cognitive fatigue. Sleep loss and circadian disruption contribute to fatigue in this setting and, although the effects of this fatigue have been studied extensively in individuals, little is understood about how fatigue impacts team performance - especially in a submarine environment. The present review provides an overview of the fatigue on submarine teams and is divided into four main sections:

1) A discussion of factors that should be considered in team fatigue research.

2) An outline of how sleep and circadian rhythms of submariners are impacted by submarine-specific factors.

3) A discussion of the known effects of fatigue from sleep loss and circadian disruption on individual performance.

4) A consideration of how this fatigue impacts team performance. As the submarine force has recognized the need to protect submariner sleep and improve team dynamics, it is vital that future research accounts for the interplay between these two factors.

C1 [Chabal, Sarah; Welles, Rebecca; Haran, F. Jay] Naval Submarine Med Res Lab, Groton, CT 06340 USA.

[Welles, Rebecca] Leidos Inc, Reston, VA USA.

[Markwald, Rachel] Naval Hlth Res Ctr, San Diego, CA USA.

RP Chabal, S (corresponding author), Naval Submarine Med Res Lab, Groton, CT 06340 USA.

EM sarah.a.chabal.civ@mail.mil

RI markwald, rachel/S-8634-2019

OI Markwald, Rachel/0000-0002-0432-3937

FU Naval Sea Systems Command Deep Submergence Biomedical Development

program [F1503]

FX The views expressed in this report are those of the authors and do not

necessarily reflect the official policy or position of the Department of

the Navy, Department of Defense, nor the U.S. Government. I am an

employee of the U.S. Government. This work was prepared as part of my

official duties. Title 17 U.S.C. 105 provides that 'Copyright protection

under this title is not available for any work of the United States

Government.' Title 17 U.S.C. 101 defines a U.S. Government work as a

work prepared by a military service member or employee of the U.S.

Government as part of that person's official duties. This work was

supported by the Naval Sea Systems Command Deep Submergence Biomedical

Development program under work unit number F1503.

CR Ainsworth LL, 1971, HUMRROTR7116

Akerstedt T, 2003, OCCUP MED-OXFORD, V53, P89, DOI 10.1093/occmed/kqg046

Akerstedt T, 2010, J SLEEP RES, V19, P298, DOI 10.1111/j.1365-2869.2009.00796.x

Alhola Paula, 2007, Neuropsychiatr Dis Treat, V3, P553

Angus KG, 1992, WHY WE NAP

[Anonymous], 2007, OPNAV INSTRUCTION 10

[Anonymous], 2014, ADV CHANGE NOTICE 1

[Anonymous], BS45 01 WATCH TEAM D

Antunes LC, 2010, NUTR RES REV, V23, P155, DOI 10.1017/S0954422410000016

Aschoff Jurgen, 1981, BIOL RHYTHMS, P311, DOI 10.1007/978-1-4615-6552-9_17

Axelsson J, 2008, CHRONOBIOL INT, V25, P297, DOI 10.1080/07420520802107031

Banks S, 2007, J CLIN SLEEP MED, V3, P519

Banks S, 2010, SLEEP, V33, P1013, DOI 10.1093/sleep/33.8.1013

Baranski JV, 2007, HUM FACTORS, V49, P646, DOI 10.1518/001872007X215728

Bard EG, 1996, SPEECH COMMUN, V20, P71, DOI 10.1016/S0167-6393(96)00045-3

Barnes C, 2004, AFRLHEBRTR20040020

Barnes CM, 2009, ACAD MANAGE REV, V34, P56, DOI 10.5465/AMR.2009.35713280

Baron RS., 1986, ADV EXP SOC PSYCHOL, P1, DOI [10.1016/S0065-2601(08)60211-7, DOI 10.1016/S0065-2601(08)60211-7]

Barsade SG, 2002, ADMIN SCI QUART, V47, P644, DOI 10.2307/3094912

Basner M, 2008, SLEEP, V31, P1251

Beauregard M, 2001, J NEUROSCI, V21, DOI 10.1523/JNEUROSCI.21-18-j0001.2001

Belenky G, 2003, J SLEEP RES, V12, P1, DOI 10.1046/j.1365-2869.2003.00337.x

BLAGROVE M, 1995, APPL COGNITIVE PSYCH, V9, P21, DOI 10.1002/acp.2350090103

Blassingame SR, 2001, THESIS

Bocca ML, 2006, CLIN NEUROPHYSIOL, V117, P894, DOI 10.1016/j.clinph.2006.01.003

Boksem MAS, 2005, COGNITIVE BRAIN RES, V25, P107, DOI 10.1016/j.cogbrainres.2005.04.011

Borbely A A, 1982, Hum Neurobiol, V1, P195

Caldwell JA, 2005, AVIAT SPACE ENVIR MD, V76, pC39

Chabal S, 2016, SUBM TECHN S 2016 LA

Chaiken S, 2011, COGNITIVE FATIGUE MU

Chang YK, 2012, BRAIN RES, V1453, P87, DOI 10.1016/j.brainres.2012.02.068

COMSUBFOR, 2014, COMM INT US SUBM FOR

Czeisler CA, 1999, SCIENCE, V284, P2177, DOI 10.1126/science.284.5423.2177

Dasborough MT, 2009, LEADERSHIP QUART, V20, P571, DOI 10.1016/j.leaqua.2009.04.009

Davidson RJ, 1999, TRENDS COGN SCI, V3, P11, DOI 10.1016/S1364-6613(98)01265-0

De Dreu CKW, 2003, J APPL PSYCHOL, V88, P741, DOI 10.1037/0021-9010.88.4.741

Dijk D, 1992, J SLEEP RES, V1

Dijk DJ, 2001, AM J PHYSIOL-REG I, V281, pR1647, DOI 10.1152/ajpregu.2001.281.5.R1647

Dinges DF, 1997, SLEEP, V20, P267

Dinges OF, 1992, J SLEEP RES, V21

Dorrian J, 2003, SLEEP, V26, P871, DOI 10.1093/sleep/26.7.871

DOWD PJ, 1974, J APPL PSYCHOL, V59, P748, DOI 10.1037/h0037512

Duffy JF, 2005, J BIOL RHYTHM, V20, P326, DOI 10.1177/0748730405277983

Duplessis CA, 2003, UNDERSEA HYPERBAR M, V34, P21

Engle-Friedman M, 2003, J SLEEP RES, V12, P113, DOI 10.1046/j.1365-2869.2003.00351.x

Etkin A, 2011, TRENDS COGN SCI, V15, P85, DOI 10.1016/j.tics.2010.11.004

Frings D, 2015, J APPL SOC PSYCHOL, V45, P461, DOI 10.1111/jasp.12312

Frings D, 2011, J EXP PSYCHOL-APPL, V17, P371, DOI 10.1037/a0025131

Gawron V., 2015, HUM PERF EXTREM ENV, V12

Gladstein DL, 1994, ADM SCI Q, V29, P499

GRAYBIEL A, 1976, AVIAT SPACE ENVIR MD, V47, P873

GRAYBIEL A, 1965, AEROSPACE MED, V36, P733

GRIFFITT W, 1971, J PERS SOC PSYCHOL, V17, P92, DOI 10.1037/h0030458

Gunzelmann G, 2010, COGNITIVE FATIGUE MU

Hackman J.R., 1987, HDB ORG BEHAV

HARKINS SG, 1989, J PERS SOC PSYCHOL, V56, P934, DOI 10.1037/0022-3514.56.6.934

Harrington John, 2012, Dent Clin North Am, V56, P319, DOI 10.1016/j.cden.2012.01.005

Harrison Y, 1997, SLEEP, V20, P871, DOI 10.1093/sleep/20.10.871

Harrison Y, 1999, ORGAN BEHAV HUM DEC, V78, P128, DOI 10.1006/obhd.1999.2827

Harrison Y, 2000, J EXP PSYCHOL-APPL, V6, P236, DOI 10.1037//1076-898X.6.3.236

Harville D, 2005, AFRLHEBRRT20050085

Henelius A, 2014, SLEEP, V37, P1257, DOI 10.5665/sleep.3850

Hoeksema-van Orden CYD, 1998, J PERS SOC PSYCHOL, V75, P1179, DOI 10.1037/0022-3514.75.5.1179

Hunt PD, 1995, 951A NAV HLTH RES C

Irwin M, 1996, FASEB J, V10, P643, DOI 10.1096/fasebj.10.5.8621064

Jackson CL, 2003, J APPL PSYCHOL, V88, P459, DOI 10.1037/0021-9010.88.3.459

Kahn-Greene ET, 2006, PERS INDIV DIFFER, V41, P1433, DOI 10.1016/j.paid.2006.06.002

Kamphuis J, 2012, SLEEP MED, V13, P327, DOI 10.1016/j.sleep.2011.12.006

Karatsoreos IN, 2012, CURR NEUROL NEUROSCI, V12, P218, DOI 10.1007/s11910-012-0252-0

Karau S.J., 1998, GROUP DYN-THEOR RES, V2, P185, DOI [10.1037/1089-2699.2.3.185, DOI 10.1037/1089-2699.2.3.185]

Keller MW, 2011, 2011001 NSMRL

Kelly TL, 1999, J BIOL RHYTHM, V14, P190, DOI 10.1177/074873099129000597

Killgore WDS, 2006, J SLEEP RES, V15, P7, DOI 10.1111/j.1365-2869.2006.00487.x

Killgore WDS, 2008, SLEEP MED, V9, P517, DOI 10.1016/j.sleep.2007.07.003

Kiniorski ET, 2004, AVIAT SPACE ENVIR MD, V75, P872

Krueger GP, 1989, 89221 USAARL

Krueger GP, 1991, HDB MILITARY PSYCHOL, P244

Labelle V, 2013, BRAIN COGNITION, V81, P10, DOI 10.1016/j.bandc.2012.10.001

LATANE B, 1979, J PERS SOC PSYCHOL, V37, P822, DOI 10.1037//0022-3514.37.6.822

LAUGHLIN PR, 1983, J PERS SOC PSYCHOL, V45, P94, DOI 10.1037/0022-3514.45.1.94

Lawson BD, 1998, ACTA ASTRONAUT, V43, P181, DOI 10.1016/S0094-5765(98)00153-2

Lawson BD, 2012, 201211 USAARL

Leach LS, 2009, HEALTH CARE MANAGE R, V34, P29, DOI 10.1097/01.HMR.0000342977.84307.64

Lepine JA, 2001, ACAD MANAGE REV, V26, P67, DOI 10.2307/259395

Leproult R, 2003, AM J PHYSIOL-REG I, V284, pR280, DOI 10.1152/ajpregu.00197.2002

LEWY AJ, 1980, SCIENCE, V210, P1267, DOI 10.1126/science.7434030

Lim J, 2010, NEUROIMAGE, V49, P3426, DOI 10.1016/j.neuroimage.2009.11.020

Lorist MM, 2000, PSYCHOPHYSIOLOGY, V37, P614, DOI 10.1111/1469-8986.3750614

MASCORD DJ, 1992, J SAFETY RES, V23, P19, DOI 10.1016/0022-4375(92)90036-9

MHAT 9, 2013, OP END FREED OEF 201, P9

Miller JC, 2003, TR1226 NSMRL

Miller NL, 2012, OXFORD HDB MILITARY

Mockel T, 2015, SCI REP-UK, V5, DOI 10.1038/srep10113

Money KE, 1969, MOTION SICKNESS

Nagai M, 2011, J OCCUP HEALTH, V53, P312, DOI 10.1539/joh.10-0072-OA

NAITOH P, 1983, International Journal of Chronobiology, V8, P149

Odle-Dusseau HN, 2010, CHRONOBIOL INT, V27, P318, DOI 10.3109/07420520903502226

Perelli LP, 1980, SAMTR80491 BROOKS AI

Perrier J, 2016, BIOL PSYCHOL, V121, P1, DOI 10.1016/j.biopsycho.2016.09.010

Philip P, 2005, IND HEALTH, V43, P30, DOI 10.2486/indhealth.43.30

Pilcher JJ, 2002, ERGONOMICS, V45, P682, DOI 10.1080/00140130210158419

Pilcher JJ, 1996, SLEEP, V19, P318, DOI 10.1093/sleep/19.4.318

Pilcher JJ, 1997, J AM COLL HEALTH, V46, P121, DOI 10.1080/07448489709595597

Pilcher JJ, 2011, ERGONOMICS, V54, P587, DOI 10.1080/00140139.2011.592599

Porkka-Heiskanen T, 2000, NEUROSCIENCE, V99, P507, DOI 10.1016/S0306-4522(00)00220-7

Porkka-Heiskanen Tarja, 2003, ScientificWorldJournal, V3, P790

Presidential Commission on the Space Shuttle Challenger Accident, 1986, REP PRES COMM SPAC S

Rabat A, 2016, FRONT BEHAV NEUROSCI, V10, DOI 10.3389/fnbeh.2016.00095

Revell VL, 2005, J BIOL RHYTHM, V20, P270, DOI 10.1177/0748730405275655

Saksvik IB, 2011, SLEEP MED REV, V15, P221, DOI 10.1016/j.smrv.2010.07.002

Salas E, 2005, SMALL GR RES, V36, P555, DOI 10.1177/1046496405277134

Sallinen M, 2013, J SLEEP RES, V22, P273, DOI 10.1111/jsr.12013

Sandal GM, 1970, REV ENVIRON SCI BIO, V5121, P399

Saper CB, 2005, NATURE, V437, P1257, DOI 10.1038/nature04284

SCHAEFER KE, 1979, UNDERSEA BIOMED RES, V6, pS81

Scheer FAJL, 2009, P NATL ACAD SCI USA, V106, P4453, DOI 10.1073/pnas.0808180106

Schoenewolf G., 1990, MODERN PSYCHOANALYSI, V15, P49

Shay J., 1998, PARAMETERS, V28, P93

Smith ME, 2002, SLEEP, V25, P784

Stern JA, 1994, DOTFAAAM94261

Stern JA, 1996, DOTFAAAM9691

Stolgitis WC, 1969, THESIS

TAUB JM, 1973, PSYCHOPHYSIOLOGY, V10, P559, DOI 10.1111/j.1469-8986.1973.tb00805.x

TAUB JM, 1974, PSYCHOSOM MED, V36, P164, DOI 10.1097/00006842-197403000-00008

Tharion WJ, 2003, AVIAT SPACE ENVIR MD, V74, P309

Thomas M, 2000, J SLEEP RES, V9, P335, DOI 10.1046/j.1365-2869.2000.00225.x

Thomas Maria L., 2003, Thalamus & Related Systems, V2, P199, DOI 10.1016/S1472-9288(03)00020-7

TILLEY A, 1984, Q J EXP PSYCHOL-A, V36, P281, DOI 10.1080/14640748408402159

van der Hulst M., 2001, TRANSPORT RES F-TRAF, V4, P103, DOI DOI 10.1016/S1369-8478(01)00017-1

Van Dongen HPA, 2003, SLEEP, V26, P117, DOI 10.1093/sleep/26.2.117

Van Dongen HPA, 2005, SLEEP, V28, P479, DOI 10.1093/sleep/28.4.479

Van Dongen HPA, 2004, SLEEP, V27, P423

Vgontzas AN, 2004, J CLIN ENDOCR METAB, V89, P2119, DOI 10.1210/jc.2003-031562

Watson NF, 2015, J CLIN SLEEP MED, V11, P591, DOI 10.5664/jcsm.4758

Whitmire A.M., 2009, NASA, P85

Whitmore J, 1996, SPEECH COMMUN, V20, P55, DOI 10.1016/S0167-6393(96)00044-1

Whitmore J, 2008, AFRLRHBRTR20080005

WILKINSON R, 1961, J EXP PSYCHOL, V62, P263, DOI 10.1037/h0048787

WILLIAMS K, 1981, J PERS SOC PSYCHOL, V40, P303, DOI 10.1037/0022-3514.40.2.303

Wright HR, 2004, J PINEAL RES, V36, P140, DOI 10.1046/j.1600-079X.2003.00108.x

Wright KP, 2001, P NATL ACAD SCI USA, V98, P14027, DOI 10.1073/pnas.201530198

Wyatt JK, 1999, AM J PHYSIOL-REG I, V277, pR1152

Yoo SS, 2007, CURR BIOL, V17, pR877, DOI 10.1016/j.cub.2007.08.007

Young CR, 2015, J BIOL RHYTHM, V30, P144, DOI 10.1177/0748730415575432

NR 144

TC 3

Z9 6

U1 1

U2 21

PU UNDERSEA & HYPERBARIC MEDICAL SOC INC

PI DURHAM

PA 21 WEST COLONY PLACE, STE 280, DURHAM, NC 27705 USA

SN 1066-2936

J9 UNDERSEA HYPERBAR M

JI Undersea Hyperb. Med.

PD MAY-JUN

PY 2018

VL 45

IS 3

BP 257

EP 272

PG 16

WC Marine & Freshwater Biology; Medicine, Research & Experimental

WE Science Citation Index Expanded (SCI-EXPANDED); Social Science Citation Index (SSCI)

SC Marine & Freshwater Biology; Research & Experimental Medicine

GA HL3QA

UT WOS:000458629700002

PM 30028913

DA 2022-05-04

ER

PT J

AU Smirmaul, BPC

de Moraes, AC

Angius, L

Marcora, SM

AF Smirmaul, Bruno P. C.

de Moraes, Antonio Carlos

Angius, Luca

Marcora, Samuele M.

TI Effects of caffeine on neuromuscular fatigue and performance during

high-intensity cycling exercise in moderate hypoxia

SO EUROPEAN JOURNAL OF APPLIED PHYSIOLOGY

LA English

DT Article

DE Altitude; Perception of effort; Central fatigue; Peripheral fatigue;

Exercise performance

ID LOCOMOTOR MUSCLE FATIGUE; PERIPHERAL FATIGUE; MENTAL EXERTION; POWER

OUTPUT; INGESTION; PERCEPTION; RESPONSES; CAPACITY; FEEDBACK; HUMANS

AB To investigate the effects of caffeine on performance, neuromuscular fatigue and perception of effort during high-intensity cycling exercise in moderate hypoxia.

Seven adult male participants firstly underwent an incremental exercise test on a cycle ergometer in conditions of acute normobaric hypoxia (fraction inspired oxygen = 0.15) to establish peak power output (PPO). In the following two visits, they performed a time to exhaustion test (78 +/- 3% PPO) in the same hypoxic conditions after caffeine ingestion (4 mg kg(-1)) and one after placebo ingestion in a double-blind, randomized, counterbalanced cross-over design.

Caffeine significantly improved time to exhaustion by 12%. A significant decrease in subjective fatigue was found after caffeine consumption. Perception of effort and surface electromyographic signal amplitude of the vastus lateralis were lower and heart rate was higher in the caffeine condition when compared to placebo. However, caffeine did not reduce the peripheral and central fatigue induced by high-intensity cycling exercise in moderate hypoxia.

The caffeine-induced improvement in time to exhaustion during high-intensity cycling exercise in moderate hypoxia seems to be mediated by a reduction in perception of effort, which occurs despite no reduction in neuromuscular fatigue.

C1 [Smirmaul, Bruno P. C.] Sao Paulo State Univ UNESP, Dept Phys Educ, Rio Claro, SP, Brazil.

[de Moraes, Antonio Carlos] Univ Campinas UNICAMP, Fac Phys Educ, Sao Paulo, SP, Brazil.

[Angius, Luca; Marcora, Samuele M.] Univ Kent Medway, Sch Sport & Exercise Sci, Endurance Res Grp, Chatham ME4 4AG, Kent, England.

RP Marcora, SM (corresponding author), Univ Kent Medway, Sch Sport & Exercise Sci, Endurance Res Grp, Chatham ME4 4AG, Kent, England.

EM brunosmirmaul@gmail.com; acmoraes@fef.unicamp.br;

lucaangius83@gmail.com; s.m.marcora@kent.ac.uk

RI Marcora, Samuele/W-4284-2019; MORAES, ANTONIO C/J-1056-2016; Smirmaul,

Bruno P/H-3905-2012

OI Marcora, Samuele/0000-0002-1570-7936; Smirmaul, Bruno

P/0000-0001-7612-3099; Angius, Luca/0000-0003-4606-4272; MORAES, ANTONIO

CARLOS/0000-0002-0489-8204

FU CNPQ (Conselho Nacional de Desenvolvimento Cientifico)Conselho Nacional

de Desenvolvimento Cientifico e Tecnologico (CNPQ) [131724/2011-8];

Santander International Mobility Grant-PRPG UNICAMP

FX BPCS received support by the CNPQ (Conselho Nacional de Desenvolvimento

Cientifico)-scholarship (Grant No. 131724/2011-8)-and by the Santander

International Mobility Grant-PRPG UNICAMP.

CR Amann M, 2008, J APPL PHYSIOL, V104, P861, DOI 10.1152/japplphysiol.01008.2007

Amann M, 2006, J APPL PHYSIOL, V101, P119, DOI 10.1152/japplphysiol.01596.2005

Amann M, 2013, J APPL PHYSIOL, V115, P355, DOI 10.1152/japplphysiol.00049.2013

Astorino TA, 2010, J STRENGTH COND RES, V24, P257, DOI 10.1519/JSC.0b013e3181c1f88a

BERGLUND B, 1982, INT J SPORTS MED, V3, P234, DOI 10.1055/s-2008-1026094

Black CD, 2015, MED SCI SPORT EXER, V47, P1145, DOI 10.1249/MSS.0000000000000513

BORG GAV, 1982, MED SCI SPORT EXER, V14, P377, DOI 10.1249/00005768-198205000-00012

Bradshaw DI, 2005, RES Q EXERCISE SPORT, V76, P426

Budini F, 2014, J ELECTROMYOGR KINES, V24, P412, DOI 10.1016/j.jelekin.2014.02.003

Campbell S., 2001, P HUMAN FACTORS ERGO, V45, P906, DOI DOI 10.1177/154193120104501302

Smirmaul BDC, 2011, APPL PHYSIOL NUTR ME, V36, P773, DOI [10.1139/h11-081, 10.1139/H11-081]

Smirmaul BDC, 2012, BRIT J SPORT MED, V46, P308, DOI 10.1136/bjsm.2010.071407

Chapman Robert F, 2009, Phys Sportsmed, V37, P97, DOI 10.3810/psm.2009.12.1747

Cureton KJ, 2007, INT J SPORT NUTR EXE, V17, P35, DOI 10.1123/ijsnem.17.1.35

Dahlstrom BK, 2013, J EXERC SCI FIT, V11, P78, DOI 10.1016/j.jesf.2013.10.001

Davis JK, 2009, SPORTS MED, V39, P813, DOI 10.2165/11317770-000000000-00000

de Morree HM, 2014, J APPL PHYSIOL, V117, P1514, DOI 10.1152/japplphysiol.00898.2013

de Morree HM, 2012, PSYCHOPHYSIOLOGY, V49, P1242, DOI 10.1111/j.1469-8986.2012.01399.x

Del Coso J, 2011, APPL PHYSIOL NUTR ME, V36, P555, DOI [10.1139/h11-052, 10.1139/H11-052]

Doherty M, 2004, INT J SPORT NUTR EXE, V14, P626, DOI 10.1123/ijsnem.14.6.626

Doherty M, 2004, J SPORT SCI, V22, P637, DOI 10.1080/02640410310001655741

Eaton TR, 2016, INT J SPORT NUTR EXE, V26, P33, DOI 10.1123/ijsnem.2015-0108

Froyd C, 2013, J PHYSIOL-LONDON, V591, P1339, DOI 10.1113/jphysiol.2012.245316

FULCO CS, 1994, AVIAT SPACE ENVIR MD, V65, P539

Gandevia SC, 2006, J APPL PHYSIOL, V100, P1749, DOI 10.1152/japplphysiol.00121.2006

Goldstein ER, 2010, J INT SOC SPORT NUTR, V7, DOI 10.1186/1550-2783-7-5

Goodall S, 2012, J PHYSIOL-LONDON, V590, P2767, DOI 10.1113/jphysiol.2012.228890

Graham TE, 2001, SPORTS MED, V31, P785, DOI 10.2165/00007256-200131110-00002

Hermens HJ, 2000, J ELECTROMYOGR KINES, V10, P361, DOI 10.1016/S1050-6411(00)00027-4

HOLM S, 1979, SCAND J STAT, V6, P65

Johnson MA, 2015, J APPL PHYSIOL, V119, P840, DOI 10.1152/japplphysiol.00072.2015

Kalmar JM, 2004, EXERC SPORT SCI REV, V32, P143

KUIPERS H, 1985, INT J SPORTS MED, V6, P197, DOI 10.1055/s-2008-1025839

Landrum R.E, 1992, COLL STUD J, V26, P151

LOPES JM, 1983, J APPL PHYSIOL, V54, P1303, DOI 10.1152/jappl.1983.54.5.1303

Marcora S, 2009, J APPL PHYSIOL, V106, P2060, DOI 10.1152/japplphysiol.90378.2008

Marcora SM, 2008, AM J PHYSIOL-REG I, V294, pR874, DOI 10.1152/ajpregu.00678.2007

Marcora SM, 2010, EUR J APPL PHYSIOL, V109, P763, DOI 10.1007/s00421-010-1418-6

Mohr T, 1998, J APPL PHYSIOL, V85, P979, DOI 10.1152/jappl.1998.85.3.979

Pageaux B, 2015, FRONT HUM NEUROSCI, V9, DOI 10.3389/fnhum.2015.00067

Pageaux B, 2013, MED SCI SPORT EXER, V45, P2254, DOI 10.1249/MSS.0b013e31829b504a

Romer LM, 2007, AM J PHYSIOL-REG I, V292, pR598, DOI 10.1152/ajpregu.00269.2006

Rozand V, 2014, FRONT HUM NEUROSCI, V8, DOI 10.3389/fnhum.2014.00755

Smirmaul Bruno Paula Caraça, 2013, Rev. bras. educ. fís. esporte, V27, P333

Stadheim HK, 2015, J APPL PHYSIOL, V119, P1501, DOI 10.1152/japplphysiol.00509.2015

Strojnik V, 1998, J APPL PHYSIOL, V84, P344, DOI 10.1152/jappl.1998.84.1.344

Tallis J, 2015, BRIT J PHARMACOL, V172, P3703, DOI 10.1111/bph.13187

Tallis J, 2012, J APPL PHYSIOL, V112, P64, DOI 10.1152/japplphysiol.00801.2011

Tarnopolsky M, 2000, J APPL PHYSIOL, V89, P1719, DOI 10.1152/jappl.2000.89.5.1719

Tarnopolsky MA, 2008, APPL PHYSIOL NUTR ME, V33, P1284, DOI 10.1139/H08-121

Terry PC, 2003, PSYCHOL SPORT EXERC, V4, P125, DOI 10.1016/S1469-0292(01)00035-8

THOMAS S, 1992, CAN J SPORT SCI, V17, P338

Zenon A, 2015, J NEUROSCI, V35, P8737, DOI 10.1523/JNEUROSCI.3789-14.2015

NR 53

TC 13

Z9 16

U1 1

U2 58

PU SPRINGER

PI NEW YORK

PA 233 SPRING ST, NEW YORK, NY 10013 USA

SN 1439-6319

EI 1439-6327

J9 EUR J APPL PHYSIOL

JI Eur. J. Appl. Physiol.

PD JAN

PY 2017

VL 117

IS 1

BP 27

EP 38

DI 10.1007/s00421-016-3496-6

PG 12

WC Physiology; Sport Sciences

WE Science Citation Index Expanded (SCI-EXPANDED)

SC Physiology; Sport Sciences

GA EL0LG

UT WOS:000394313300004

PM 27864638

OA Green Published, Green Accepted, hybrid

DA 2022-05-04

ER

PT J

AU Smith, MR

Marcora, SM

Coutts, AJ

AF Smith, Mitchell R.

Marcora, Samuele M.

Coutts, Aaron J.

TI Mental Fatigue Impairs Intermittent Running Performance

SO MEDICINE AND SCIENCE IN SPORTS AND EXERCISE

LA English

DT Article

ID MATCH-RELATED FATIGUE; EXERCISE PERFORMANCE; RUGBY UNION; ENDURANCE

PERFORMANCE; BRAIN REGULATION; MOTION ANALYSIS; TEAM-SPORT; HUMANS;

INTENSITY; RESPONSES

AB Purpose

The purpose of the study was to investigate the effects of mental fatigue on intermittent running performance.

Methods

Ten male intermittent team sports players performed two identical self-paced, intermittent running protocols. The two trials were separated by 7 d and preceded, in a randomized-counterbalanced order, by 90 min of either emotionally neutral documentaries (control) or the AX-continuous performance test (AX-CPT; mental fatigue). Subjective ratings of fatigue and vigor were measured before and after these treatments, and motivation was recorded before the intermittent running protocol. Velocity, heart rate, oxygen consumption, blood glucose and lactate concentrations, and ratings of perceived exertion (RPE) were measured throughout the 45-min intermittent running protocol. Session RPE was recorded 30 min after the intermittent running protocol.

Results

Subjective ratings of fatigue were higher after the AX-CPT (P = 0.005). This mental fatigue significantly reduced velocity at low intensities (1.28 +/- 0.18 m center dot s(-1) vs 1.31 +/- 0.17 m center dot s(-1); P = 0.037), whereas high-intensity running and peak velocities were not significantly affected. Running velocity at all intensities significantly declined over time in both conditions (P < 0.001). Oxygen consumption was significantly lower in the mental fatigue condition (P = 0.007). Other physiological variables, vigor and motivation, were not significantly affected. Ratings of perceived exertion during the intermittent running protocol were not significantly different between conditions despite lower overall velocity in the mental fatigue condition. Session RPE was significantly higher in the mental fatigue condition (P = 0.013).

Conclusion

Mental fatigue impairs intermittent running performance. This negative effect of mental fatigue seems to be mediated by higher perception of effort.

C1 [Smith, Mitchell R.; Coutts, Aaron J.] Univ Technol Sydney, Fac Hlth, Sport & Exercise Discipline Grp, Sydney, NSW 2007, Australia.

[Marcora, Samuele M.] Univ Kent, Sch Sport & Exercise Sci, Endurance Res Grp, Medway ME4 4AG, Kent, England.

RP Marcora, SM (corresponding author), Univ Kent, Sch Sport & Exercise Sci, Medway ME4 4AG, Kent, England.

EM s.m.marcora@kent.ac.uk

RI Smith, Mitchell Robert/M-9396-2019; Marcora, Samuele/W-4284-2019;

Coutts, Aaron J/E-5261-2011

OI Smith, Mitchell Robert/0000-0002-8168-5405; Marcora,

Samuele/0000-0002-1570-7936; Coutts, Aaron J/0000-0002-1782-7691

FU Union of European Football Associations (UEFA)

FX Professor Samuele Marcora was funded by a grant from the Union of

European Football Associations (UEFA). The other authors declared no

conflict of interest.

CR Ackerman PL, 2011, COGNITIVE FATIGUE MU

Ahlberg K, 2003, LANCET, V362, P640, DOI 10.1016/S0140-6736(03)14186-4

Borg E, 2002, ACTA PSYCHOL, V109, P157, DOI 10.1016/S0001-6918(01)00055-5

BREHM JW, 1989, ANNU REV PSYCHOL, V40, P109, DOI 10.1146/annurev.ps.40.020189.000545

Brownsberger J, 2013, INT J SPORTS MED, V34, P1029, DOI 10.1055/s-0033-1343402

Budgett R, 1998, BRIT J SPORT MED, V32, P107, DOI 10.1136/bjsm.32.2.107

Campbell S., 2001, P HUMAN FACTORS ERGO, V45, P906, DOI DOI 10.1177/154193120104501302

Cook CJ, 2012, PHYSIOL BEHAV, V106, P683, DOI 10.1016/j.physbeh.2012.05.009

Coutts A, 2003, J SPORT SCI, V21, P97, DOI 10.1080/0264041031000070831

Coutts AJ, 2010, J SCI MED SPORT, V13, P543, DOI 10.1016/j.jsams.2009.09.004

Deutsch MU, 2007, J SPORT SCI, V25, P461, DOI 10.1080/02640410600631298

Duffield R, 2009, J STRENGTH COND RES, V23, P1238, DOI 10.1519/JSC.0b013e318194e0b1

Duthie G, 2003, SPORTS MED, V33, P973, DOI 10.2165/00007256-200333130-00003

Edwards AM, 2013, SPORTS MED, V43, P1057, DOI 10.1007/s40279-013-0091-4

Fairclough SH, 2004, BIOL PSYCHOL, V66, P177, DOI 10.1016/j.biopsycho.2003.10.001

Foster C, 2001, J STRENGTH COND RES, V15, P109, DOI 10.1519/00124278-200102000-00019

Gailliot MT, 2007, J PERS SOC PSYCHOL, V92, P325, DOI 10.1037/0022-3514.92.2.325

Kempton T, 2013, J SPORT SCI, V31, P1770, DOI 10.1080/02640414.2013.803583

Kohlisch O, 1996, ERGONOMICS, V39, P213, DOI 10.1080/00140139608964452

Kurzban R, 2013, BEHAV BRAIN SCI, V36, P661, DOI 10.1017/S0140525X12003196

Lorist MM, 2005, COGNITIVE BRAIN RES, V24, P199, DOI 10.1016/j.cogbrainres.2005.01.018

Marcora SM, 2008, EUR J APPL PHYSIOL, V104, P929, DOI 10.1007/s00421-008-0818-3

Marcora SM, 2009, J APPL PHYSIOL, V106, P857, DOI 10.1152/japplphysiol.91324.2008

Marcora SM, 2009, J APPL PHYSIOL, V106, P344, DOI 10.1152/japplphysiol.zdg-8326.pcpcomm.2008

Marcora SM, 2010, EUR J APPL PHYSIOL, V109, P763, DOI 10.1007/s00421-010-1418-6

Mashiko T, 2004, BRIT J SPORT MED, V38, P617, DOI 10.1136/bjsm.2003.007690

MCAULEY E, 1992, J APPL SOC PSYCHOL, V22, P312, DOI 10.1111/j.1559-1816.1992.tb01542.x

Noakes TD, 2005, BRIT J SPORT MED, V39, P120, DOI 10.1136/bjsm.2003.010330

Pageaux B, 2014, EUR J APPL PHYSIOL, V114, P1095, DOI 10.1007/s00421-014-2838-5

Pageaux B, 2013, MED SCI SPORT EXER, V45, P2254, DOI 10.1249/MSS.0b013e31829b504a

Patterson SD, 2007, INT J SPORT NUTR EXE, V17, P445, DOI 10.1123/ijsnem.17.5.445

Rampinini E, 2007, INT J SPORTS MED, V28, P1018, DOI 10.1055/s-2007-965158

Rampinini E, 2008, MED SCI SPORT EXER, V40, P934, DOI 10.1249/MSS.0b013e3181666eb8

Sirotic AC, 2008, J SCI MED SPORT, V11, P500, DOI 10.1016/j.jsams.2007.04.008

Skyes D, 2009, INT J PERF ANAL SPOR, V9, P47

Spencer M, 2004, J SPORT SCI, V22, P843, DOI 10.1080/02640410410001716715

Terry PC, 2003, PSYCHOL SPORT EXERC, V4, P125, DOI 10.1016/S1469-0292(01)00035-8

Tucker R, 2009, BRIT J SPORT MED, V43, P392, DOI 10.1136/bjsm.2008.050799

Ulmer HV, 1996, EXPERIENTIA, V52, P416, DOI 10.1007/BF01919309

Waldron M, 2014, SPORTS MED, V44, P1645, DOI 10.1007/s40279-014-0230-6

NR 40

TC 105

Z9 106

U1 1

U2 63

PU LIPPINCOTT WILLIAMS & WILKINS

PI PHILADELPHIA

PA TWO COMMERCE SQ, 2001 MARKET ST, PHILADELPHIA, PA 19103 USA

SN 0195-9131

EI 1530-0315

J9 MED SCI SPORT EXER

JI Med. Sci. Sports Exerc.

PD AUG

PY 2015

VL 47

IS 8

BP 1682

EP 1690

DI 10.1249/MSS.0000000000000592

PG 9

WC Sport Sciences

WE Science Citation Index Expanded (SCI-EXPANDED); Social Science Citation Index (SSCI)

SC Sport Sciences

GA CM8IF

UT WOS:000357942200016

PM 25494389

DA 2022-05-04

ER

PT J

AU Herlambang, MB

Taatgen, NA

Cnossen, F

AF Herlambang, Mega B.

Taatgen, Niels A.

Cnossen, Fokie

TI Modeling motivation using goal competition in mental fatigue studies

SO JOURNAL OF MATHEMATICAL PSYCHOLOGY

LA English

DT Article

DE Cognitive modeling; Cognitive architecture; Goal competition; Mental

fatigue; Motivation; PRIMs

ID WORK; LOAD

AB Motivation can counteract the effects of mental fatigue. However, the underlying mechanism by which motivation affects performance in mentally fatiguing tasks is obscure.

In this paper, we propose goal competition as a paradigm to understand the role of motivation and built three models of mental fatigue studies to demonstrate the mechanism in a cognitive architecture named PRIMs. Each of these studies explored the impact of reward and mental fatigue on performance. Overall, performance decreased in nonreward conditions but remained stable in reward conditions.

The comparisons between our models and empirical data showed that our models were able to capture human performance. We managed to model changes in performance levels by adjusting the value of the main task goals, which controls the competition with distractions. In all the tasks modeled, the best model fits were obtained by a linear decrease in goal activation, suggesting this is a general pattern. We discuss possible mechanisms for activation decrease, and the potential of goal competition to model motivation. (C) 2021 The Authors. Published by Elsevier Inc.

C1 [Herlambang, Mega B.; Taatgen, Niels A.; Cnossen, Fokie] Univ Groningen, Bernoulli Inst Math Comp Sci & Artificial Intelli, Groningen, Netherlands.

[Herlambang, Mega B.] Inst Teknol Indonesia, Dept Ind Engn, South Tangerang, Indonesia.

RP Herlambang, MB (corresponding author), Univ Groningen, Bernoulli Inst Math Comp Sci & Artificial Intelli, Groningen, Netherlands.

EM m.b.herlambang@rug.nl; n.a.taatgen@rug.nl; f.cnossen@rug.nl

OI Taatgen, Niels/0000-0001-7286-3460; Cnossen, Fokie/0000-0001-7435-4889;

Herlambang, Mega Bagus/0000-0001-8946-2604

FU Lembaga Pengelola Dana Pendidikan (LPDP) from the Indonesia government

FX This study was supported by Lembaga Pengelola Dana Pendidikan (LPDP)

from the Indonesia government awarded to Mega B. Herlambang.

CR AASMAN J, 1987, HUM FACTORS, V29, P161, DOI 10.1177/001872088702900204

Ackerman P.L., 2011, COGNITIVE FATIGUE MU, P149164, DOI [10.1037/12343-007, DOI 10.1037/12343-007]

Akerstedt T, 2004, J PSYCHOSOM RES, V57, P427, DOI 10.1016/j.jpsychores.2003.12.001

Anderson JR, 2004, PSYCHOL REV, V111, P1036, DOI 10.1037/0033-295x.111.4.1036

Boksem MAS, 2008, BRAIN RES REV, V59, P125, DOI 10.1016/j.brainresrev.2008.07.001

Boksem MAS, 2006, BIOL PSYCHOL, V72, P123, DOI 10.1016/j.biopsycho.2005.08.007

Chong TTJ, 2017, PLOS BIOL, V15, DOI 10.1371/journal.pbio.1002598

Craig CM, 2019, HUM FACTORS, V61, P426, DOI 10.1177/0018720818822350

Di Domenico SI, 2017, FRONT HUM NEUROSCI, V11, DOI 10.3389/fnhum.2017.00145

Doran SM, 2001, ARCH ITAL BIOL, V139, P253

Earle F, 2015, MOTIV EMOTION, V39, P467, DOI 10.1007/s11031-015-9481-2

Gunzelmann G., 2011, COGNITIVE FATIGUE MU, P83

Helton WS, 2017, HUM FACTORS, V59, P91, DOI 10.1177/0018720816683509

Herlambang MB, 2021, PLOS ONE, V16, DOI 10.1371/journal.pone.0243754

Herlambang MB, 2019, HUM FACTORS, V61, P1171, DOI 10.1177/0018720819828569

Hockey G.R.J., 2011, COGNITIVE FATIGUE MU, P167, DOI [10.1037/12343-008, DOI 10.1037/12343-008]

Hockey R, 2013, PSYCHOLOGY OF FATIGUE: WORK, EFFORT AND CONTROL, P132

Hopstaken JF, 2015, BIOL PSYCHOL, V110, P100, DOI 10.1016/j.biopsycho.2015.06.013

Huijser S, 2018, CONSCIOUS COGN, V58, P170, DOI 10.1016/j.concog.2017.12.004

Jongman G. M. G., 1998, P 2 EUR C COGN MOD, P52

KRUPP LB, 1989, ARCH NEUROL-CHICAGO, V46, P1121, DOI 10.1001/archneur.1989.00520460115022

Kurzban R, 2013, BEHAV BRAIN SCI, V36, P661, DOI 10.1017/S0140525X12003196

Muller T, 2019, NEUROPSYCHOLOGIA, V123, P141, DOI 10.1016/j.neuropsychologia.2018.04.030

Powers WT., 1973, BEHAV CONTROL PERCEP

Qi P, 2019, ENGINEERING-PRC, V5, P276, DOI 10.1016/j.eng.2018.11.025

Ryan RM, 2000, CONTEMP EDUC PSYCHOL, V25, P54, DOI 10.1006/ceps.1999.1020

Taatgen NA, 2013, PSYCHOL REV, V120, P439, DOI 10.1037/a0033138

van der Linden D, 2003, ACTA PSYCHOL, V113, P45, DOI 10.1016/S0001-6918(02)00150-6

Warm JS, 2008, HUM FACTORS, V50, P433, DOI 10.1518/001872008X312152

Wessely S., 1998, CHRONIC FATIGUE ITS

Wigfield A., 2006, HDB CHILD PSYCHOL, P933

NR 31

TC 1

Z9 1

U1 2

U2 3

PU ACADEMIC PRESS INC ELSEVIER SCIENCE

PI SAN DIEGO

PA 525 B ST, STE 1900, SAN DIEGO, CA 92101-4495 USA

SN 0022-2496

EI 1096-0880

J9 J MATH PSYCHOL

JI J. Math. Psychol.

PD JUN

PY 2021

VL 102

AR 102540

DI 10.1016/j.jmp.2021.102540

EA MAY 2021

PG 11

WC Mathematics, Interdisciplinary Applications; Social Sciences,

Mathematical Methods; Psychology, Mathematical

WE Science Citation Index Expanded (SCI-EXPANDED); Social Science Citation Index (SSCI)

SC Mathematics; Mathematical Methods In Social Sciences; Psychology

GA SJ4KT

UT WOS:000655502200009

OA hybrid, Green Published

DA 2022-05-04

ER

PT J

AU Yung, M

Manji, R

Wells, RP

AF Yung, Marcus

Manji, Rahim

Wells, Richard P.

TI Exploring the Relationship of Task Performance and Physical and

Cognitive Fatigue During a Daylong Light Precision Task

SO HUMAN FACTORS

LA English

DT Article

DE fatigue; errors; performance; low-load work; work measurement

ID HUMAN MUSCLE FATIGUE; MENTAL FATIGUE; NEUROMUSCULAR FATIGUE;

PHYSIOLOGICAL TREMOR; UPPER-EXTREMITY; WORK; PIPETTES; CONTRACTIONS;

ERGONOMICS; ATTENTION

AB Objective: Our aim was to explore the relationship between fatigue and operation system performance during a simulated light precision task over an 8-hr period using a battery of physical (central and peripheral) and cognitive measures.

Background: Fatigue may play an important role in the relationship between poor ergonomics and deficits in quality and productivity. However, well-controlled laboratory studies in this area have several limitations, including the lack of work relevance of fatigue exposures and lack of both physical and cognitive measures. There remains a need to understand the relationship between physical and cognitive fatigue and task performance at exposure levels relevant to realistic production or light precision work.

Method: Errors and fatigue measures were tracked over the course of a micropipetting task. Fatigue responses from 10 measures and errors in pipetting technique, precision, and targeting were submitted to principal component analysis to descriptively analyze features and patterns.

Results: Fatigue responses and error rates contributed to three principal components (PCs), accounting for 50.9% of total variance. Fatigue responses grouped within the three PCs reflected central and peripheral upper extremity fatigue, postural sway, and changes in oculomotor behavior.

Conclusion: In an 8-hr light precision task, error rates shared similar patterns to both physical and cognitive fatigue responses, and/or increases in arousal level.

Application: The findings provide insight toward the relationship between fatigue and operation system performance (e.g., errors). This study contributes to a body of literature documenting task errors and fatigue, reflecting physical (both central and peripheral) and cognitive processes.

C1 [Yung, Marcus] Univ Waterloo, Waterloo, ON, Canada.

[Manji, Rahim] Univ Waterloo, Ergon & Occupat Biomech Lab, Waterloo, ON, Canada.

[Wells, Richard P.] Univ Waterloo, Dept Kinesiol, 200 Univ Ave West, Waterloo, ON N2L 3G1, Canada.

RP Yung, M (corresponding author), Univ Waterloo, Dept Kinesiol, 200 Univ Ave West, Waterloo, ON N2L 3G1, Canada.

EM m4yung@uwaterloo.ca

OI Yung, Marcus/0000-0003-3192-1470

FU AUTO21 Network Centres of Excellence [A506-AWH]; CIHR GrantCanadian

Institutes of Health Research (CIHR) [MAT-9186]

FX The authors would like to acknowledge Joe Church for his contributions

during data collection. This study was funded by a research grant

provided by AUTO21 Network Centres of Excellence (A506-AWH). Development

of the custom Nintendo Wii balance board data acquisition software was

funded by CIHR Grant (#MAT-9186).

CR Adamo DE, 2002, EUR J APPL PHYSIOL, V88, P134, DOI 10.1007/s00421-002-0660-y

Axelsson J., 2000, THESIS

Babault N, 2006, J APPL PHYSIOL, V100, P780, DOI 10.1152/japplphysiol.00737.2005

BASCHERA P, 1979, ERGONOMICS, V22, P377, DOI 10.1080/00140137908924622

Basmajian J.V., 1985, MUSCLES ALIVE

Behm DG, 2004, CAN J APPL PHYSIOL, V29, P274, DOI 10.1139/h04-019

Bilodeau M, 2009, J ELECTROMYOGR KINES, V19, P131, DOI 10.1016/j.jelekin.2007.06.014

Boksem MAS, 2006, BIOL PSYCHOL, V72, P123, DOI 10.1016/j.biopsycho.2005.08.007

Boksem MAS, 2005, COGNITIVE BRAIN RES, V25, P107, DOI 10.1016/j.cogbrainres.2005.04.011

Buckley RJ, 2016, CONSCIOUS COGN, V45, P174, DOI 10.1016/j.concog.2016.09.002

Carignan B, 2012, EUR J APPL PHYSIOL, V112, P1269, DOI 10.1007/s00421-011-2080-3

Carpenter MG, 2010, NEUROSCIENCE, V171, P196, DOI 10.1016/j.neuroscience.2010.08.030

Ciriello V.M, 2002, THEORETICAL ISSUES E, V3, P274, DOI DOI 10.1080/14639220110114672

Cote JN, 2002, EXP BRAIN RES, V146, P394, DOI 10.1007/s00221-002-1186-6

Cote JN, 2008, MOTOR CONTROL, V12, P79, DOI 10.1123/mcj.12.2.79

Cram J.R., 1998, INTRO SURFACE ELECTR

Curran S, 1998, HUM PSYCHOPHARM CLIN, V13, P337, DOI 10.1002/(SICI)1099-1077(199807)13:5<337::AID-HUP7>3.3.CO;2-G

David G, 1997, APPL ERGON, V28, P257, DOI 10.1016/S0003-6870(97)00002-1

Davranche K, 2005, J SPORT EXERCISE PSY, V27, P515, DOI 10.1123/jsep.27.4.515

Dennerlein JT, 2003, AIHA J-J SCI OCCUP E, V64, P799, DOI 10.1080/15428110308984875

Drury CG, 1997, ERGONOMICS, V40, P249, DOI 10.1080/001401397188134

Dudschig C, 2009, BRAIN RES, V1296, P56, DOI 10.1016/j.brainres.2009.08.009

Dutilh G, 2012, ATTEN PERCEPT PSYCHO, V74, P454, DOI 10.3758/s13414-011-0243-2

Eagles J. B., 1955, S FAT, P41

Eklund J A, 1999, Int J Occup Saf Ergon, V5, P143

ELBLE RJ, 1976, J NEUROPHYSIOL, V39, P370, DOI 10.1152/jn.1976.39.2.370

ENOKA RM, 1992, J APPL PHYSIOL, V72, P1631, DOI 10.1152/jappl.1992.72.5.1631

Epling SL, 2016, EXP BRAIN RES, V234, P2979, DOI 10.1007/s00221-016-4700-y

Falck AC, 2010, HUM FACTOR ERGON MAN, V20, P24, DOI 10.1002/hfm.20172

Folkard S, 2004, AVIAT SPACE ENVIR MD, V75, pA161

FREDRIKSSON K, 1995, ERGONOMICS, V38, P1067, DOI 10.1080/00140139508925173

Gandevia SC, 1998, ACTA PHYSIOL SCAND, V162, P275, DOI 10.1046/j.1365-201X.1998.0299f.x

Gibson EL, 2002, NUTR RES REV, V15, P169, DOI 10.1079/NRR200131

Greig M, 2008, ERGONOMICS, V51, P1238, DOI 10.1080/00140130802037289

Hallett M, 1998, MOVEMENT DISORD, V13, P43

HAMMARSKJOLD E, 1992, EUR J APPL PHYSIOL, V64, P402, DOI 10.1007/BF00625058

Hamrol A, 2011, HUM FACTOR ERGON MAN, V21, P156, DOI 10.1002/hfm.20233

Head J, 2014, ACTA PSYCHOL, V153, P87, DOI 10.1016/j.actpsy.2014.09.007

Head JR, 2016, FRONT PHYSIOL, V7, DOI 10.3389/fphys.2016.00373

HESTER SB, 1980, J NERV MENT DIS, V168, P679, DOI 10.1097/00005053-198011000-00007

Holtzer R, 2010, AGING NEUROPSYCHOL C, V18, P108, DOI 10.1080/13825585.2010.517826

Hubley-Kozey CL, 2006, J ELECTROMYOGR KINES, V16, P365, DOI 10.1016/j.jelekin.2005.07.014

Jolliffe I., 2002, PRINCIPAL COMPONENT, P1

Lakie M, 2004, J NEUROL NEUROSUR PS, V75, P1013, DOI 10.1136/jnnp.2003.022749

Leyk D, 2006, EUR J APPL PHYSIOL, V96, P593, DOI 10.1007/s00421-005-0126-0

Lintula M, 2006, INT J IND ERGONOM, V36, P257, DOI 10.1016/j.ergon.2005.06.011

Lu ML, 2008, INT J IND ERGONOM, V38, P18, DOI 10.1016/j.ergon.2007.08.006

Madeleine P, 2001, J ELECTROMYOGR KINES, V11, P113, DOI 10.1016/S1050-6411(00)00044-4

Mehta RK, 2012, EUR J APPL PHYSIOL, V112, P2891, DOI 10.1007/s00421-011-2264-x

Meijman TF, 1997, INT J IND ERGONOM, V20, P31, DOI 10.1016/S0169-8141(96)00029-7

Mizuno K., 2008, FATIGUE SCI HUMAN HL, P47, DOI 10.1007/978-4-431-73464-2_4

Morris TL, 1996, BIOL PSYCHOL, V42, P343, DOI 10.1016/0301-0511(95)05166-X

Neumann W. P., 2016, 8 IFAC C MAN MOD MAN

Neumann WP, 2010, INT J OPER PROD MAN, V30, P923, DOI 10.1108/01443571011075056

Nussbaum M.A., 2013, IIE T OCCUPATIONAL E, V1, P16, DOI [10.1080/21577323.2011.637153, DOI 10.1080/21577323.2011.637153]

Oxendine J.B., 1970, QUEST, V13, P23, DOI [10.1080/00336297.1970.10519673, DOI 10.1080/00336297.1970.10519673]

Paillard T, 2012, NEUROSCI BIOBEHAV R, V36, P162, DOI 10.1016/j.neubiorev.2011.05.009

Park JK, 2013, ERGONOMICS, V56, P1147, DOI 10.1080/00140139.2013.799234

Reason J. T., 1990, HUMAN ERROR

Rose LM, 2013, HUM FACTOR ERGON MAN, V23, P368, DOI 10.1002/hfm.20324

Saxby DJ, 2013, J EXP PSYCHOL-APPL, V19, P287, DOI 10.1037/a0034386

Schleicher R, 2008, ERGONOMICS, V51, P982, DOI 10.1080/00140130701817062

Shinohara M, 2006, EXERC SPORT SCI REV, V34, P59, DOI 10.1249/00003677-200604000-00004

Siegle GJ, 2008, PSYCHOPHYSIOLOGY, V45, P679, DOI 10.1111/j.1469-8986.2008.00681.x

SIMONSON E, 1952, PHYSIOL REV, V32, P349, DOI 10.1152/physrev.1952.32.3.349

Sogaard K, 2003, J ELECTROMYOGR KINES, V13, P441, DOI 10.1016/S1050-6411(03)00075-0

Srinivasan D, 2015, J ELECTROMYOGR KINES, V25, P121, DOI 10.1016/j.jelekin.2014.10.011

STERN JA, 1984, PSYCHOPHYSIOLOGY, V21, P22, DOI 10.1111/j.1469-8986.1984.tb02312.x

Styles E., 2014, INTRO COGNITIVE PSYC, P70

VANHILTEN JJ, 1991, J NEUROL NEUROSUR PS, V54, P516, DOI 10.1136/jnnp.54.6.516

Vollestad NK, 1997, J NEUROSCI METH, V74, P219, DOI 10.1016/S0165-0270(97)02251-6

Weir JP, 2006, BRIT J SPORT MED, V40, P573, DOI 10.1136/bjsm.2005.023028

Westerblad H, 2010, EXP CELL RES, V316, P3093, DOI 10.1016/j.yexcr.2010.05.019

Westgaard RH, 1996, APPL ERGON, V27, P79, DOI 10.1016/0003-6870(95)00062-3

WIKER SF, 1989, ERGONOMICS, V32, P211, DOI 10.1080/00140138908966080

Yung M, 2013, J ELECTROMYOGR KINES, V23, P664, DOI 10.1016/j.jelekin.2013.01.001

Yung M., 2017, DOCUMENTING TE UNPUB

Yung M., 2016, THESIS

Yung M, 2017, ERGONOMICS, V60, P923, DOI 10.1080/00140139.2016.1243734

Yung M, 2017, ERGONOMICS, V60, P940, DOI 10.1080/00140139.2016.1242782

Yung M, 2014, ERGONOMICS, V57, P1562, DOI 10.1080/00140139.2014.934299

NR 81

TC 11

Z9 11

U1 1

U2 33

PU SAGE PUBLICATIONS INC

PI THOUSAND OAKS

PA 2455 TELLER RD, THOUSAND OAKS, CA 91320 USA

SN 0018-7208

EI 1547-8181

J9 HUM FACTORS

JI Hum. Factors

PD NOV

PY 2017

VL 59

IS 7

BP 1029

EP 1047

DI 10.1177/0018720817717026

PG 19

WC Behavioral Sciences; Engineering, Industrial; Ergonomics; Psychology,

Applied; Psychology

WE Science Citation Index Expanded (SCI-EXPANDED); Social Science Citation Index (SSCI)

SC Behavioral Sciences; Engineering; Psychology

GA FJ1PU

UT WOS:000412490700001

PM 28658591

DA 2022-05-04

ER

PT J

AU Daniels, GL

Newell, KM

AF Daniels, GL

Newell, KM

TI Attentional focus influences the walk-run transition in human locomotion

SO BIOLOGICAL PSYCHOLOGY

LA English

DT Article

DE walking; running; gait transitions; perceived exertion; attentional

demand; dual-task paradigm

ID PERCEIVED EXERTION; OXYGEN-CONSUMPTION; ENERGY-EXPENDITURE; TREADMILL

WALKING; HUMAN OPERATOR; HEART-RATE; EXERCISE; SPEED; HABITUATION;

DETERMINANTS

AB This study tested the hypothesis that cognitive perceptual processes are involved in determining the walk-run transition in human locomotion. In a dual-task paradigm, 12 healthy male participants (aged 21.8 +/- 2.4 years) walked and ran on a treadmill while solving mental arithmetic (MA) organized in two levels of difficulty (easy and hard). Speed was increased over seven increments of 0.1 ms(-1) while the walk-run transition speed (TS) and central and peripheral ratings of perceived exertion (RPE) were recorded. MA performance was maintained between control (no locomotion) and treadmill conditions and participants rated hard MA as more difficult and more mentally engaging than easy MA. The TS increased during both levels of MA, although RPE values did not reflect psychological attenuation across the transition. Together, these results support the hypothesis that cognitive load distracts attentional focus from physiological cues that contribute to triggering human gait transitions. (C) 2003 Elsevier Science B.V. All rights reserved.

C1 Penn State Univ, Dept Kinesiol, Coll Hlth & Human Dev, University Pk, PA 16802 USA.

US Mil Acad, Dept Educ Phys, West Point, NY 10996 USA.

RP Daniels, GL (corresponding author), Penn State Univ, Dept Kinesiol, Coll Hlth & Human Dev, 201 Henderson Bldg, University Pk, PA 16802 USA.

EM pg0107@exmail.usma.army.mil; kmn1@psu.edu

CR ABERNETHY B, 1988, J HUM MOVEMENT STUD, V14, P101

Abernethy B, 1995, ADV PSYCHOL, V111, P171

BEUTER A, 1988, NEUROSCI RES COMMUN, V3, P127

Borg GA, 1962, PHYSICAL PERFORMANCE

BORG GAV, 1973, MED SCI SPORT EXER, V5, P90

BOUTCHER SH, 1990, J SPORT EXERCISE PSY, V12, P167, DOI 10.1123/jsep.12.2.167

Brown LA, 1999, J GERONTOL A-BIOL, V54, pM165, DOI 10.1093/gerona/54.4.M165

CARROLL D, 1986, PSYCHOPHYSIOLOGY, V23, P174, DOI 10.1111/j.1469-8986.1986.tb00613.x

CASPERSEN CJ, 1986, UNPUB TECHNICAL MONO

CHARTERIS J, 1978, PERCEPT MOTOR SKILL, V47, P659, DOI 10.2466/pms.1978.47.2.659

Craik KJW, 1948, B J PSYCHOL-GEN SECT, V38, P142, DOI 10.1111/j.2044-8295.1948.tb01149.x

Craik KJW, 1947, B J PSYCHOL-GEN SECT, V38, P56, DOI 10.1111/j.2044-8295.1947.tb01141.x

DANIELS GL, 2002, UNPUB PERCEIVED TASK

DIEDRICH FJ, 1995, J EXP PSYCHOL HUMAN, V21, P183, DOI 10.1037/0096-1523.21.1.183

GEURTS ACH, 1994, J MOTOR BEHAV, V26, P162, DOI 10.1080/00222895.1994.9941670

HARDY CJ, 1986, J SPORT EXERCISE PSY, V8, P88, DOI 10.1123/jsp.8.2.88

Hassmen P, 1995, PERCEPT PSYCHOPHYS, P53

HOLT KG, 1995, J MOTOR BEHAV, V27, P164, DOI 10.1080/00222895.1995.9941708

HRELJAC A, 1993, MED SCI SPORT EXER, V25, P1158

HRELJAC A, 1995, HUM MOVEMENT SCI, V14, P205, DOI 10.1016/0167-9457(95)00017-M

HRELJAC A, 1993, GAIT POSTURE, V1, P217, DOI 10.1016/0966-6362(93)90049-7

HRELJAC A, 1995, J BIOMECH, V28, P669, DOI 10.1016/0021-9290(94)00120-S

JOHNSON J, 1987, CAN J SPORT SCI, V12, P41

Kahneman D., 1973, ATTENTION EFFORT

Kram R, 1997, J EXP BIOL, V200, P821

Lindenberger U, 2000, PSYCHOL AGING, V15, P417, DOI 10.1037//0882-7974.15.3.417

MCCAUL KD, 1984, PSYCHOL BULL, V95, P516, DOI 10.1037/0033-2909.95.3.516

MERCIER J, 1994, EUR J APPL PHYSIOL O, V69, P525, DOI 10.1007/BF00239870

MINETTI AE, 1994, ACTA PHYSIOL SCAND, V150, P315, DOI 10.1111/j.1748-1716.1994.tb09692.x

MORGAN WP, 1983, COGNITIVE THER RES, V7, P251, DOI 10.1007/BF01205139

MORGAN WP, 1981, RES Q EXERCISE SPORT, V52, P385, DOI 10.1080/02701367.1981.10607886

*NAT CTR HLTH STAT, 1991, VITAL HLTH STAT, V10, P185

NETHERY VM, 1991, J HUM MOVEMENT STUD, V20, P201

Noble B. J., 1996, PERCEIVED EXERTION

NOBLE BJ, 1973, MED SCI SPORT EXER, V5, P116

Patla A.E., 2000, ENERGETICS HUMAN ACT, P43

PENNEBAKER JW, 1982, PSYCHOL PHYSICAL SYM

Prilutsky BI, 2001, J EXP BIOL, V204, P2277

REJESKI WJ, 1985, J SPORT EXERCISE PSY, V7, P371, DOI 10.1123/jsp.7.4.371

Robergs RA, 1998, PERCEPT MOTOR SKILL, V86, P915, DOI 10.2466/pms.1998.86.3.915

SCHIEB DA, 1986, RES Q EXERCISE SPORT, V57, P1

SCHOENBORN CA, 1986, PUBLIC HEALTH REP, V101, P571

SCHONER G, 1990, J THEOR BIOL, V142, P359, DOI 10.1016/S0022-5193(05)80558-2

SHEPHARD RJ, 1988, SPORTS MED, V5, P185, DOI 10.2165/00007256-198805030-00005

Sparrow WA, 1998, PSYCHON B REV, V5, P173, DOI 10.3758/BF03212943

Temprado JJ, 1999, J EXP PSYCHOL HUMAN, V25, P1579, DOI 10.1037/0096-1523.25.6.1579

THORSTENSSON A, 1987, ACTA PHYSIOL SCAND, V131, P211, DOI 10.1111/j.1748-1716.1987.tb08228.x

TURNER JR, 1985, PSYCHOPHYSIOLOGY, V22, P261, DOI 10.1111/j.1469-8986.1985.tb01597.x

Turvey MT, 1999, J MOTOR BEHAV, V31, P265, DOI 10.1080/00222899909600993

vanEmmerik REA, 1996, J BIOMECH, V29, P1175, DOI 10.1016/0021-9290(95)00128-X

WALL JC, 1980, ERGONOMICS, V23, P425, DOI 10.1080/00140138008924758

WALL JC, 1981, ERGONOMICS, V24, P531, DOI 10.1080/00140138108924874

Wickens C.D., 1980, ATTENT PERFORM 8, V8, P239, DOI 10.4324/9781315802961

[No title captured]

[No title captured]

NR 55

TC 28

Z9 28

U1 2

U2 17

PU ELSEVIER SCIENCE BV

PI AMSTERDAM

PA PO BOX 211, 1000 AE AMSTERDAM, NETHERLANDS

SN 0301-0511

EI 1873-6246

J9 BIOL PSYCHOL

JI Biol. Psychol.

PD MAY

PY 2003

VL 63

IS 2

BP 163

EP 178

DI 10.1016/S0301-0511(03)00024-3

PG 16

WC Psychology, Biological; Behavioral Sciences; Psychology; Psychology,

Experimental

WE Science Citation Index Expanded (SCI-EXPANDED); Social Science Citation Index (SSCI)

SC Psychology; Behavioral Sciences

GA 693TL

UT WOS:000183734400005

PM 12738406

DA 2022-05-04

ER

PT J

AU Renfree, A

do Carmo, EC

Martin, L

Peters, DM

AF Renfree, Andrew

do Carmo, Everton Crivoi

Martin, Louise

Peters, Derek M.

TI The Influence of Collective Behavior on Pacing in Endurance Competitions

SO FRONTIERS IN PHYSIOLOGY

LA English

DT Article

DE decision-making; endurance performance; complex systems; sport

AB A number of theoretical models have been proposed in recent years to explain pacing strategies observed in individual competitive endurance events. These have typically related to the internal regulatory processes that inform the making of decisions relating to muscular work rate. Despite a substantial body of research which has investigated the influence of collective group dynamics on individual behaviors in various animal species, this issue has not been comprehensively studied in individual athletic events. This is somewhat surprising given that athletes often directly compete in close proximity to one another, and that collective behavior has also been observed in other human environments including pedestrian interactions and financial market trading. Whilst the reasons for adopting collective behavior are not fully understood, collective behavior is thought to result from individual agents following simple local rules that result in seemingly complex large systems that act to confer some biological advantage to the collective as a whole. Although such collective behaviors may generally be beneficial, competitive endurance events are complicated by the fact that increasing levels of physiological disruption as activity progresses may compromise the ability of some individuals to continue to interact with other group members. This could result in early fatigue and relative underperformance due to suboptimal utilization of physiological resources by some athletes. Alternatively, engagement with a collective behavior may benefit all due to a reduction in the complexity of decisions to be made and a subsequent reduction in cognitive loading and mental fatigue. This paper seeks evidence for collective behavior in previously published analyses of pacing behavior and proposes mechanisms through which it could potentially be either beneficial, or detrimental to individual performance. It concludes with suggestions for future research to enhance understanding of this phenomenon.

C1 [Renfree, Andrew; Martin, Louise; Peters, Derek M.] Univ Worcester, Inst Sport & Exercise Sci, Worcester, England.

[do Carmo, Everton Crivoi] Senac Univ Ctr, Dept Phys Educ, Sao Paulo, Brazil.

[Peters, Derek M.] Univ Agder, Fac Hlth & Sport Sci, Kristiansand, Norway.

RP Renfree, A (corresponding author), Univ Worcester, Inst Sport & Exercise Sci, Worcester, England.

EM a.renfree@worc.ac.uk

RI Carmo, Everton C/F-7129-2012

OI Carmo, Everton C/0000-0003-1353-0206; Renfree,

Andrew/0000-0001-9039-8574; Peters, Derek/0000-0002-7873-7737

CR Abbiss CR, 2008, SPORTS MED, V38, P239, DOI 10.2165/00007256-200838030-00004

BANERJEE AV, 1992, Q J ECON, V107, P797, DOI 10.2307/2118364

Baron B, 2011, BRIT J SPORT MED, V45, P511, DOI 10.1136/bjsm.2009.059964

Cont R, 2000, MACROECON DYN, V4, P170

Couzin ID, 2002, J THEOR BIOL, V218, P1, DOI 10.1006/jtbi.2002.3065

de Koning JJ, 2011, PLOS ONE, V6, DOI 10.1371/journal.pone.0015863

Duarte R, 2012, SPORTS MED, V42, P633, DOI 10.2165/11632450-000000000-00000

Esteve-Lanao J, 2014, INT J SPORT PHYSIOL, V9, P1000, DOI 10.1123/ijspp.2013-0457

FOSTER C, 1994, SPORTS MED, V17, P77, DOI 10.2165/00007256-199417020-00001

Giardina I, 2008, HFSP J, V2, P205, DOI 10.2976/1.2961038

Giomi L, 2013, P ROY SOC A-MATH PHY, V469, DOI 10.1098/rspa.2012.0637

Hanley B, 2015, J SPORT SCI, V33, P1189, DOI 10.1080/02640414.2014.988742

Hanley B, 2014, J SPORT SCI, V32, P1060, DOI 10.1080/02640414.2013.878807

Helbing D, 2005, TRANSPORT SCI, V39, P1, DOI 10.1287/trsc.1040.0108

King AJ, 2012, CURR BIOL, V22, pR112, DOI 10.1016/j.cub.2011.11.033

Konings MJ, 2016, INT J SPORT PHYSIOL, V11, P122, DOI 10.1123/ijspp.2015-0137

KYLE CR, 1979, ERGONOMICS, V22, P387, DOI 10.1080/00140137908924623

Marcora SM, 2009, J APPL PHYSIOL, V106, P857, DOI 10.1152/japplphysiol.91324.2008

Marcora SM, 2010, EUR J APPL PHYSIOL, V109, P763, DOI 10.1007/s00421-010-1418-6

Mytton GJ, 2015, INT J SPORT PHYSIOL, V10, P369, DOI 10.1123/ijspp.2014-0207

Renfree A, 2014, SPORTS MED, V44, P147, DOI 10.1007/s40279-013-0107-0

Renfree A, 2013, INT J SPORT PHYSIOL, V8, P279, DOI 10.1123/ijspp.8.3.279

Renfree A, 2012, INT J SPORT PHYSIOL, V7, P121, DOI 10.1123/ijspp.7.2.121

Skorski S, 2013, INT J SPORTS MED, V34, P152, DOI 10.1055/s-0032-1316357

Skorski S, 2014, INT J SPORT PHYSIOL, V9, P217, DOI [10.1123/ijspp.2012-0258, 10.1123/IJSPP.2012-0258]

Smits BLM, 2014, SPORTS MED, V44, P763, DOI 10.1007/s40279-014-0163-0

Swart J, 2009, BRIT J SPORT MED, V43, P775, DOI 10.1136/bjsm.2008.056036

Trenchard H., 2010, AAAI FALL S COMPL AD

Trenchard H, 2015, APPL MATH COMPUT, V270, P179, DOI 10.1016/j.amc.2015.08.006

Trenchard H, 2014, PHYSICA A, V405, P92, DOI 10.1016/j.physa.2014.03.002

Tucker R, 2009, BRIT J SPORT MED, V43, P392, DOI 10.1136/bjsm.2008.050799

VOHS KD, 2014, MOTIVATION SCI, V1, P19, DOI DOI 10.1037/2333-8113.1.S.19

Wolfram S., 1988, EMERGING SYNTHESES S, P183

Zouhal H, 2015, INT J SPORT PHYSIOL, V10, P147, DOI 10.1123/ijspp.2013-0498

NR 34

TC 22

Z9 23

U1 1

U2 2

PU FRONTIERS MEDIA SA

PI LAUSANNE

PA AVENUE DU TRIBUNAL FEDERAL 34, LAUSANNE, CH-1015, SWITZERLAND

SN 1664-042X

J9 FRONT PHYSIOL

JI Front. Physiol.

PD DEC 9

PY 2015

VL 6

AR 373

DI 10.3389/fphys.2015.00373

PG 5

WC Physiology

WE Science Citation Index Expanded (SCI-EXPANDED)

SC Physiology

GA VF7RK

UT WOS:000443541800001

PM 26696903

OA Green Published, gold, Green Accepted

DA 2022-05-04

ER

PT J

AU Lorist, MM

Kernell, D

Meijman, TF

Zijdewind, I

AF Lorist, MM

Kernell, D

Meijman, TF

Zijdewind, I

TI Motor fatigue and cognitive task performance in humans

SO JOURNAL OF PHYSIOLOGY-LONDON

LA English

DT Article

ID MENTAL FATIGUE; INFORMATION; FORCE; ACTIVATION

AB During fatiguing submaximal contractions a constant force production can be obtained at the cost of an increasing central command intensity. Little is known about the interaction between the underlying central mechanisms driving motor behaviour and cognitive functions. To address this issue, subjects performed four tasks: an auditory choice reaction task (CRT), a CRT simultaneously with a fatiguing or a non-fatiguing submaximal muscle contraction task, and a fatiguing submaximal contraction task alone. Results showed that performance in the single-CRT condition was relatively stable. However, in the fatiguing dual-task condition, performance levels in the cognitive CRT deteriorated drastically with time-on-task. Moreover, in the fatiguing dual-task condition the rise in force variability was significantly larger than during the fatiguing submaximal contraction alone. Thus, our results indicate a mutual interaction between cognitive functions and the central mechanisms driving motor behaviour during fatigue. The precise nature of this interference, and at what level this interaction takes place is still unknown.

C1 Univ Groningen, Dept Med Physiol, NL-9712 TS Groningen, Netherlands.

Univ Groningen, Dept Expt & Work Psychol, NL-9712 TS Groningen, Netherlands.

RP Lorist, MM (corresponding author), Univ Groningen, Dept Med Physiol, Grote Kruisstr 2-1, NL-9712 TS Groningen, Netherlands.

EM m.m.lorist@ppsw.rug.nlk

RI Zijdewind, inge/A-7506-2008

OI Zijdewind, inge/0000-0003-2146-1212; Lorist, Monicque

M./0000-0002-7361-093X

CR BiglandRitchie B, 1995, ADV EXP MED BIOL, V384, P361

DETTMERS C, 1995, J NEUROPHYSIOL, V74, P802, DOI 10.1152/jn.1995.74.2.802

Dettmers C, 1996, NEUROREPORT, V7, P2103, DOI 10.1097/00001756-199609020-00008

DIMITRIJEVIC MR, 1992, J NEUROL SCI, V109, P49, DOI 10.1016/0022-510X(92)90092-Y

Freeman GL, 1933, AM J PSYCHOL, V45, P17, DOI 10.2307/1414185

Gandevia SC, 2001, PHYSIOL REV, V81, P1725, DOI 10.1152/physrev.2001.81.4.1725

GRATTON G, 1988, J EXP PSYCHOL HUMAN, V14, P331, DOI 10.1037/0096-1523.14.3.331

Hockey G.R.J., 1993, ATTENTION SELECTION, P328

LIPPOLD O, 1981, HUMAN MUSCLE FATIGUE, P234

Lorist MM, 2000, PSYCHOPHYSIOLOGY, V37, P614, DOI 10.1111/1469-8986.3750614

MAYSTON M, 1994, J PHYSIOL-LONDON, V480P, pP44

Meijman TF, 1997, INT J IND ERGONOM, V20, P31, DOI 10.1016/S0169-8141(96)00029-7

Miller J, 1998, J EXP PSYCHOL HUMAN, V24, P1521, DOI 10.1037/0096-1523.24.5.1521

MULDER G, 1983, J CHILD PSYCHOL PSYC, V24, P19, DOI 10.1111/j.1469-7610.1983.tb00100.x

Pachella R.G., 1974, HUMAN INFORM PROCESS, P41

QUINTANA SM, 1994, J EDUC STAT, V19, P57, DOI 10.3102/10769986019001057

SANDERS AF, 1990, ACTA PSYCHOL, V74, P123, DOI 10.1016/0001-6918(90)90004-Y

SCHNEIDER W, 1988, BEHAV RES METH INSTR, V20, P206, DOI 10.3758/BF03203833

Schubert M, 1998, NEUROSCI RES, V30, P125, DOI 10.1016/S0168-0102(97)00115-6

Wickens C.D., 1984, VARIETIES ATTENTION, P63

Wickens C.D., 2000, ENG PSYCHOL HUMAN PE, V5th ed.

ZIJDEWIND I, 1994, J APPL PHYSIOL, V77, P987, DOI 10.1152/jappl.1994.77.2.987

Zijdewind I, 2001, J NEUROPHYSIOL, V85, P1907, DOI 10.1152/jn.2001.85.5.1907

[No title captured]

NR 24

TC 116

Z9 117

U1 1

U2 39

PU WILEY-BLACKWELL

PI MALDEN

PA COMMERCE PLACE, 350 MAIN ST, MALDEN 02148, MA USA

SN 0022-3751

J9 J PHYSIOL-LONDON

JI J. Physiol.-London

PD NOV 15

PY 2002

VL 545

IS 1

BP 313

EP 319

DI 10.1113/jphysiol.2002.027938

PG 7

WC Neurosciences; Physiology

WE Science Citation Index Expanded (SCI-EXPANDED); Social Science Citation Index (SSCI)

SC Neurosciences & Neurology; Physiology

GA 620VN

UT WOS:000179553500030

PM 12433971

OA Green Submitted, Green Published

DA 2022-05-04

ER

PT J

AU Atashfeshan, N

Razavi, H

AF Atashfeshan, Nooshin

Razavi, Hamideh

TI Determination of the Proper Rest Time for a Cyclic Mental Task Using

ACT-R Architecture

SO HUMAN FACTORS

LA English

DT Article

DE rest breaks; mental fatigue; cognitive task; ACT-R architecture

ID COGNITIVE ARCHITECTURE; SLEEP-DEPRIVATION; FATIGUE; PERFORMANCE; WORK;

INTERFERENCE; SIMULATOR

AB Objective: Analysis of the effect of mental fatigue on a cognitive task and determination of the right start time for rest breaks in work environments. Background: Mental fatigue has been recognized as one of the most important factors influencing individual performance. Subjective and physiological measures are popular methods for analyzing fatigue, but they are restricted to physical experiments. Computational cognitive models are useful for predicting operator performance and can be used for analyzing fatigue in the design phase, particularly in industrial operations and inspections where cognitive tasks are frequent and the effects of mental fatigue are crucial.

Method: A cyclic mental task is modeled by the ACT-R architecture, and the effect of mental fatigue on response time and error rate is studied. The task includes visual inspections in a production line or control workstation where an operator has to check products' conformity to specifications. Initially, simulated and experimental results are compared using correlation coefficients and paired t test statistics. After validation of the model, the effects are studied by human and simulated results, which are obtained by running 50-minute tests.

Results: It is revealed that during the last 20 minutes of the tests, the response time increased by 20%, and during the last 12.5 minutes, the error rate increased by 7% on average.

Conclusion: The proper start time for the rest period can be identified by setting a limit on the error rate or response time.

Application: The proposed model can be applied early in production planning to decrease the negative effects of mental fatigue by predicting the operator performance. It can also be used for determining the rest breaks in the design phase without an operator in the loop.

C1 [Atashfeshan, Nooshin; Razavi, Hamideh] Ferdowsi Univ Mashhad, Mashhad, Iran.

RP Razavi, H (corresponding author), Ferdowsi Univ Mashhad, Fac Engn, POB 9177948974, Mashhad, Iran.

EM h-razavi@um.ac.ir

RI Razavi, Hamideh/AAD-1063-2020

OI Razavi, Hamideh/0000-0003-1837-7933

CR Anderson J., 1998, ATOMIC COMPONENTS TH

Anderson J. R., 2007, CAN HUMAN MIND OCCUR

Anderson J. R., 1993, RULES MIND

ANDERSON JR, 1981, J EXP PSYCHOL-HUM L, V7, P326

Anderson JR, 2004, PSYCHOL REV, V111, P1036, DOI 10.1037/0033-295x.111.4.1036

ANDERSON JR, 1974, COGNITIVE PSYCHOL, V6, P451, DOI 10.1016/0010-0285(74)90021-8

Anderson JR, 1996, COGNITIVE PSYCHOL, V30, P221, DOI 10.1006/cogp.1996.0007

Barker LM, 2011, ERGONOMICS, V54, P815, DOI 10.1080/00140139.2011.597878

BERTELSON P, 1963, ERGONOMICS, V6, P109, DOI 10.1080/00140136308930682

Boksem MAS, 2008, BRAIN RES REV, V59, P125, DOI 10.1016/j.brainresrev.2008.07.001

Boksem MAS, 2005, COGNITIVE BRAIN RES, V25, P107, DOI 10.1016/j.cogbrainres.2005.04.011

BUNCE DJ, 1993, PSYCHOL AGING, V8, P26, DOI 10.1037/0882-7974.8.1.26

DeMarco T., 1988, PEOPLEWARE PRODUCTIV

FISK JD, 1994, CLIN INFECT DIS, V18, pS79, DOI 10.1093/clinids/18.Supplement_1.S79

Folkard S, 2003, OCCUP MED-OXFORD, V53, P95, DOI 10.1093/occmed/kqg047

Fu W. T., 2006, P HUM FACT ERG SOC A, P1122

Gartenberg D., 2014, P HUM FACT ERG SOC 5, P909

Gonzalez Cleotilde, 2011, Cognitive Systems Research, V12, P19, DOI 10.1016/j.cogsys.2010.06.004

Gonzalez C., 2006, P C BEH REPR MOD SIM

Gunzelmann G., 2009, COGNITIVE TECHNOLOGY, V14, P14

Gunzelmann G., 2008, P 17 C BEH REPR MOD, P136

Gunzelmann G., 2015, TRANSLATIONAL ISSUES, V1, P106

Gunzelmann G, 2012, COGN SYST RES, V13, P1, DOI 10.1016/j.cogsys.2010.09.001

Gunzelmann G, 2009, HUM FACTORS, V51, P251, DOI 10.1177/0018720809334592

Howell D. C., 2012, STAT METHODS PSYCHOL

Jagannath M, 2014, APPL ERGON, V45, P1140, DOI 10.1016/j.apergo.2014.02.001

Jo S, 2012, INT J IND ERGONOM, V42, P359, DOI 10.1016/j.ergon.2012.03.004

Jongman L., 1998, P 2 EUR C COGN MOD N, P52

Kato Y, 2009, INT J PSYCHOPHYSIOL, V72, P204, DOI 10.1016/j.ijpsycho.2008.12.008

Kim KH, 2004, MED BIOL ENG COMPUT, V42, P419, DOI 10.1007/BF02344719

Klerman EB, 2007, J BIOL RHYTHM, V22, P91, DOI 10.1177/0748730407299200

Langner R, 2010, ACTA PSYCHOL, V133, P64, DOI 10.1016/j.actpsy.2009.10.001

Lew FL, 2014, ERGONOMICS, V57, P1927, DOI 10.1080/00140139.2014.937771

LISPER H, 1972, J EXP PSYCHOL, V96, P287, DOI 10.1037/h0033615

Mallis MM, 2004, AVIAT SPACE ENVIR MD, V75, pA4

Marcora SM, 2009, J APPL PHYSIOL, V106, P857, DOI 10.1152/japplphysiol.91324.2008

Meijman TF, 1997, INT J IND ERGONOM, V20, P31, DOI 10.1016/S0169-8141(96)00029-7

Meshkati N., 1988, ADV PSYCHOL, pv

Mital A, 1991, INT J IND ERGON, V8, P165, DOI DOI 10.1016/0169-8141(91)90017-G

Montgomery D.C., 2007, INTRO STAT QUALITY C

Murata A, 2005, INT J IND ERGONOM, V35, P761, DOI 10.1016/j.ergon.2004.12.003

Oh H, 2014, INT J IND ERGONOM, V44, P857, DOI 10.1016/j.ergon.2014.09.004

OKOGBAA OG, 1994, APPL ERGON, V25, P355, DOI 10.1016/0003-6870(94)90054-X

Petzold P., 1982, PSYCHOPHYSICAL JUDGE, P25, DOI DOI 10.1016/J.ERGON.2009.11.006

Picard RW, 2001, IEEE T PATTERN ANAL, V23, P1175, DOI 10.1109/34.954607

Reder L. M., 1997, P 19 ANN C COGN SCI, V19, P460

Ritter F.E., 2011, HUMAN LOOP SIMULATIO, P97

SANDERS AF, 1970, ACTA PSYCHOL, V33, P414, DOI 10.1016/0001-6918(70)90151-4

Shen KQ, 2007, IEEE T BIO-MED ENG, V54, P1231, DOI 10.1109/TBME.2007.890733

Steinborn Michael B, 2010, Adv Cogn Psychol, V5, P105, DOI 10.2478/v10053-008-0070-8

STERNBERG S, 1969, AM SCI, V57, P421

Wagner J., 2005, MULT EXP ICME

Wickens CD., 2015, ENG PSYCHOL HUMAN PE

Yang B, 2013, NEUROSCI LETT, V532, P12, DOI 10.1016/j.neulet.2012.08.080

ZBRODOFF NJ, 1995, MEM COGNITION, V23, P689, DOI 10.3758/BF03200922

Zhao CL, 2012, ACCIDENT ANAL PREV, V45, P83, DOI 10.1016/j.aap.2011.11.019

NR 56

TC 1

Z9 1

U1 1

U2 17

PU SAGE PUBLICATIONS INC

PI THOUSAND OAKS

PA 2455 TELLER RD, THOUSAND OAKS, CA 91320 USA

SN 0018-7208

EI 1547-8181

J9 HUM FACTORS

JI Hum. Factors

PD MAR

PY 2017

VL 59

IS 2

BP 299

EP 313

DI 10.1177/0018720816670767

PG 15

WC Behavioral Sciences; Engineering, Industrial; Ergonomics; Psychology,

Applied; Psychology

WE Science Citation Index Expanded (SCI-EXPANDED); Social Science Citation Index (SSCI)

SC Behavioral Sciences; Engineering; Psychology

GA EQ3KO

UT WOS:000397971200010

PM 27738278

DA 2022-05-04

ER

PT J

AU Linnhoff, S

Wolter-Weging, J

Zaehle, T

AF Linnhoff, Stefanie

Wolter-Weging, Janika

Zaehle, Tino

TI Objective electrophysiological fatigability markers and their modulation

through tDCS

SO CLINICAL NEUROPHYSIOLOGY

LA English

DT Article

DE Cognitive fatigue; Fatigability; tDCS; Sensory gating; Prepulse

inhibition; Objective measurement

ID DIRECT-CURRENT STIMULATION; HUMAN MOTOR CORTEX; MENTAL FATIGUE; MEMORY

PERFORMANCE; COGNITIVE FATIGUE; WORKING-MEMORY; THETA ACTIVITY;

SENSORIMOTOR; EEG; OSCILLATIONS

AB Objective: Cognitive fatigability is a frequent symptom after sustained performance. Fatigability is evident in healthy subjects but is also often comorbid in several neuropsychiatric diseases. However, to date, clinical diagnostic almost solely relies on the self-reported subjective experience of fatigue. The goals of this present study were i) to complement the purely subjective fatigue diagnostic with objective electrophysiological fatigability parameters and ii) to prove the potential therapeutic application of transcranial direct current stimulation (tDCS) as a fatigability intervention.

Methods: We performed a pseudo-randomized, sham-controlled, parallel-group trial. Forty healthy participants received either anodal or sham tDCS over the left dorsolateral prefrontal cortex (DLPFC) while they performed an exhaustive cognitive task to induce cognitive fatigability. To assess fatigability changes, we analyzed variations of prepulse inhibition (PPI) and P50 suppression as well as frontomedial theta and occipital alpha power with time-on-task.

Results: The task reliably induced subjective exhaustion in all participants. Furthermore, we confirmed fatigability-related increases in frontomedial theta and occipital alpha power throughout the task. Additionally, fatigability significantly reduced PPI as well as P50 sensory gating. Anodal tDCS over the left DLPFC successfully counteracted fatigability and reduced the fatigability-related increase in alpha power as well as the decline in both gating parameters.

Conclusion: Occipital alpha and sensorimotor/sensory gating are suitable parameters to assess the severity of fatigability objectively. Anodal tDCS can counteract fatigability and has therapeutic potential for the treatment of fatigability in neuropsychiatric diseases.

Significance: Fatigability can be objectively assessed by electrophysiological measures and attenuated by tDCS. (c) 2021 International Federation of Clinical Neurophysiology. Published by Elsevier B.V. All rights reserved.

C1 [Linnhoff, Stefanie; Wolter-Weging, Janika; Zaehle, Tino] Otto von Guericke Univ, Dept Neurol, Leipziger St 44, D-39120 Magdeburg, Germany.

[Zaehle, Tino] Ctr Behav Brain Sci CBBS, D-39106 Magdeburg, Germany.

RP Zaehle, T (corresponding author), Otto von Guericke Univ, Dept Neurol, Leipziger St 44, D-39120 Magdeburg, Germany.; Zaehle, T (corresponding author), Ctr Behav Brain Sci CBBS, D-39106 Magdeburg, Germany.

EM stefanie.linnhoff@med.ovgu.de; janika.wolter-weging@posteo.de;

tino.zaehle@ovgu.de

FU Hertie Foundation [P1180018]

FX This work was supported by the Hertie Foundation (P1180018).

CR Aleksandrov AA, 2016, FRONT SYST NEUROSCI, V10, DOI [10.3389/hsys.2015.00044, 10.3389/fnsys.2016.00044]

Ambrus GG, 2012, BRAIN STIMUL, V5, P499, DOI 10.1016/j.brs.2011.12.001

Antal A, 2017, CLIN NEUROPHYSIOL, V128, P1774, DOI 10.1016/j.clinph.2017.06.001

Ayache SS, 2017, BRAIN STIMUL, V10, P1001, DOI 10.1016/j.brs.2017.05.004

Bak N, 2014, HUM BRAIN MAPP, V35, P3578, DOI 10.1002/hbm.22422

Barwick F, 2012, CLIN NEUROPHYSIOL, V123, P278, DOI 10.1016/j.clinph.2011.06.027

Batouli SAH, 2020, CLIN NEUROL NEUROSUR, V189, DOI 10.1016/j.clineuro.2019.105626

Beck AT, 1993, MANUAL BECK DEPRESSI

Blumenthal TD, 2005, PSYCHOPHYSIOLOGY, V42, P1, DOI 10.1111/j.1469-8986.2005.00271.x

Boksem MAS, 2008, BRAIN RES REV, V59, P125, DOI 10.1016/j.brainresrev.2008.07.001

Boksem MAS, 2005, COGNITIVE BRAIN RES, V25, P107, DOI 10.1016/j.cogbrainres.2005.04.011

Borragan G, 2018, FRONT PSYCHOL, V9, DOI 10.3389/fpsyg.2018.02351

Caldwell JA, 2019, NEUROSCI BIOBEHAV R, V96, P272, DOI 10.1016/j.neubiorev.2018.10.024

Chalah MA, 2020, J NEURAL TRANSM, V127, P953, DOI 10.1007/s00702-020-02166-2

Chalah MA, 2017, J NEUROL SCI, V372, P131, DOI 10.1016/j.jns.2016.11.015

Chalah MA, 2015, FRONT CELL NEUROSCI, V9, DOI 10.3389/fncel.2015.00460

Charvet LE, 2018, MULT SCLER J, V24, P1760, DOI 10.1177/1352458517732842

Chaudhuri A, 2000, J NEUROL SCI, V179, P34, DOI 10.1016/S0022-510X(00)00411-1

Claros-Salinas D, 2010, J NEUROL SCI, V295, P75, DOI 10.1016/j.jns.2010.04.018

Clayton MS, 2015, TRENDS COGN SCI, V19, P188, DOI 10.1016/j.tics.2015.02.004

Craig A, 2012, PSYCHOPHYSIOLOGY, V49, P574, DOI 10.1111/j.1469-8986.2011.01329.x

Crivelli L, 2012, J INT NEUROPSYCH SOC, V18, P757, DOI 10.1017/S1355617712000410

Field A., 2018, DISCOVERING STAT USI, V5th

Fiene M, 2018, J NEUROL, V265, P607, DOI 10.1007/s00415-018-8754-6

FISK JD, 1994, CAN J NEUROL SCI, V21, P9, DOI 10.1017/S0317167100048691

Gandiga PC, 2006, CLIN NEUROPHYSIOL, V117, P845, DOI 10.1016/j.clinph.2005.12.003

GRATTON G, 1983, ELECTROEN CLIN NEURO, V55, P468, DOI 10.1016/0013-4694(83)90135-9

Grundey J, 2017, ADDICT BEHAV, V74, P90, DOI 10.1016/j.addbeh.2017.05.017

Grundey J, 2015, PSYCHOPHARMACOLOGY, V232, P2491, DOI 10.1007/s00213-015-3880-7

Hanken K, 2016, FRONT NEUROL, V7, DOI 10.3389/fneur.2016.00154

Holstein DH, 2013, PSYCHIAT RES, V205, P117, DOI 10.1016/j.psychres.2012.08.013

Ji B, 2013, EXP BRAIN RES, V229, P139, DOI 10.1007/s00221-013-3600-7

JOHNS MW, 1991, SLEEP, V14, P540, DOI 10.1093/sleep/14.6.540

Klem G H, 1999, Electroencephalogr Clin Neurophysiol Suppl, V52, P3

Klimesch W, 1999, BRAIN RES REV, V29, P169, DOI 10.1016/S0165-0173(98)00056-3

Kluger BM, 2013, NEUROLOGY, V80, P409, DOI 10.1212/WNL.0b013e31827f07be

Kumari M, 2009, PSYCHONEUROENDOCRINO, V34, P1476, DOI 10.1016/j.psyneuen.2009.05.001

Lal SKL, 2002, PSYCHOPHYSIOLOGY, V39, P313, DOI 10.1017/S0048577201393095

Langner R, 2010, ACTA PSYCHOL, V133, P64, DOI 10.1016/j.actpsy.2009.10.001

Le Mansec Y, 2019, EUR J APPL PHYSIOL, V119, P1323, DOI 10.1007/s00421-019-04124-7

Liebetanz D, 2002, BRAIN, V125, P2238, DOI 10.1093/brain/awf238

Light Gregory A, 2010, Curr Protoc Neurosci, VChapter 6, DOI 10.1002/0471142301.ns0625s52

Linnhoff S, 2019, BRAIN SCI, V9, DOI 10.3390/brainsci9050100

Mangia AL, 2014, FRONT HUM NEUROSCI, V8, DOI 10.3389/fnhum.2014.00601

Mann C, 2008, NEUROPSYCHOPHARMACOL, V33, P1653, DOI 10.1038/sj.npp.1301556

Marcora SM, 2009, J APPL PHYSIOL, V106, P857, DOI 10.1152/japplphysiol.91324.2008

Mathewson KE, 2014, J COGNITIVE NEUROSCI, V26, P2400, DOI 10.1162/jocn_a_00637

Mattioli F, 2016, MULT SCLER J, V22, P222, DOI 10.1177/1352458515587597

Mayer AR, 2009, NEUROIMAGE, V44, P182, DOI 10.1016/j.neuroimage.2008.08.025

Mazaheri A, 2010, BIOL PSYCHIAT, V67, P617, DOI 10.1016/j.biopsych.2009.11.022

Mazaheri A, 2009, HUM BRAIN MAPP, V30, P1791, DOI 10.1002/hbm.20763

McIntire LK, 2017, BRAIN STIMUL, V10, P1070, DOI 10.1016/j.brs.2017.08.005

McIntire LK, 2014, BRAIN STIMUL, V7, P499, DOI 10.1016/j.brs.2014.04.008

Micoulaud-Franchi JA, 2015, BIOL PSYCHOL, V107, P16, DOI 10.1016/j.biopsycho.2015.03.002

Miller J, 2015, NEUROSCI LETT, V588, P114, DOI 10.1016/j.neulet.2015.01.014

Monte-Silva K, 2013, BRAIN STIMUL, V6, P424, DOI 10.1016/j.brs.2012.04.011

Morris ME, 2002, J NEUROL NEUROSUR PS, V72, P361, DOI 10.1136/jnnp.72.3.361

Nasseri P, 2015, FRONT HUM NEUROSCI, V9, DOI 10.3389/fnhum.2015.00054

Nelson JT, 2014, NEUROIMAGE, V85, P909, DOI 10.1016/j.neuroimage.2012.11.061

Nitsche MA, 2000, J PHYSIOL-LONDON, V527, P633, DOI 10.1111/j.1469-7793.2000.t01-1-00633.x

Pageaux B, 2013, MED SCI SPORT EXER, V45, P2254, DOI 10.1249/MSS.0b013e31829b504a

Patterson JV, 2008, PSYCHIAT RES, V158, P226, DOI 10.1016/j.psychres.2007.02.009

Philip P, 2005, IND HEALTH, V43, P30, DOI 10.2486/indhealth.43.30

Reato D, 2013, FRONT HUM NEUROSCI, V7, DOI 10.3389/fnhum.2013.00687

Saiote C, 2014, RESTOR NEUROL NEUROS, V32, P423, DOI 10.3233/RNN-130372

Simmons RD, 2010, J NEUROL, V257, P926, DOI 10.1007/s00415-009-5441-7

Swerdlow NR, 2016, J PSYCHOPHARMACOL, V30, P1072, DOI 10.1177/0269881116661075

SWERDLOW NR, 1993, BIOL PSYCHIAT, V34, P253, DOI 10.1016/0006-3223(93)90079-S

van der Linden D, 2006, INT J PSYCHOPHYSIOL, V62, P168, DOI 10.1016/j.ijpsycho.2006.04.001

Wascher E, 2014, BIOL PSYCHOL, V96, P57, DOI 10.1016/j.biopsycho.2013.11.010

Zaehle T, 2011, BMC NEUROSCI, V12, DOI 10.1186/1471-2202-12-2

NR 71

TC 1

Z9 1

U1 3

U2 5

PU ELSEVIER IRELAND LTD

PI CLARE

PA ELSEVIER HOUSE, BROOKVALE PLAZA, EAST PARK SHANNON, CO, CLARE, 00000,

IRELAND

SN 1388-2457

EI 1872-8952

J9 CLIN NEUROPHYSIOL

JI Clin. Neurophysiol.

PD JUL

PY 2021

VL 132

IS 7

BP 1721

EP 1732

DI 10.1016/j.clinph.2021.02.391

EA JUN 2021

PG 12

WC Clinical Neurology; Neurosciences

WE Science Citation Index Expanded (SCI-EXPANDED); Social Science Citation Index (SSCI)

SC Neurosciences & Neurology

GA SP2QH

UT WOS:000659518700014

PM 33867262

DA 2022-05-04

ER

PT J

AU Chen, ZL

Xu, XF

Zhang, JP

Liu, YS

Xu, XG

Li, LL

Wang, W

Xu, HS

Jiang, W

Wang, Y

AF Chen, Zhenling

Xu, Xianfa

Zhang, Jianping

Liu, Yongsuo

Xu, Xianggang

Li, Lili

Wang, Wei

Xu, Haishan

Jiang, Wei

Wang, Yan

TI Application of LC-MS-Based Global Metabolomic Profiling Methods to Human

Mental Fatigue

SO ANALYTICAL CHEMISTRY

LA English

DT Article

ID IONIZATION MASS-SPECTROMETRY; IN-VITRO MODEL; DRIVER FATIGUE; RAT-BRAIN;

NANOPARTICLES; CELLS; METABONOMICS; ASCORBATE; ISCHEMIA; SURFACES

AB Mental fatigue is characterized by a reduced capacity for work and a loss of capacity to respond to stimulation and is usually accompanied by a feeling of tiredness and drowsiness. Mental fatigue at work is a serious problem and can raise safety concerns especially in the transportation system. It is believed that mental fatigue is a direct or contributing cause of road and air related accidents and incidents. Psychological studies indicate that fatigue results in reduced work efficiency, alertness, and impaired mental performance. However, its underlying biochemical mechanisms are poorly understood. We hypothesized that the human body is an integrated system, and mental fatigue results in changes not only in psychology but also in biochemistry of the human body. These biochemical changes are detectable in metabolites. We employed global metabolomic profiling methods to screen biochemical changes that occur with mental fatigue in air traffic controllers (ATCs) in civil aviation. A total of 45, all male, ATCs (two batches) were recruited as two mental fatigue groups and 23 executive staff acted as a control group for this study. The volunteers' urine samples were collected before and after their work. The samples were analyzed with liquid chromatography/mass spectrometry equipped with a polar, a weak polar, and a nonpolar column, respectively. Three candidate biomarkers were selected on the basis of statistical significance, coefficient of variance, and compared with data of the three groups. The results suggest that urine metabolites may provide a complete new clue from biochemistry to understand, monitor, and manage human mental fatigue.

C1 [Chen, Zhenling; Xu, Xianfa; Liu, Yongsuo; Li, Lili; Wang, Wei; Xu, Haishan; Jiang, Wei; Wang, Yan] Civil Aviat Adm China, Civil Aviat Hosp, Civil Aviat Med Ctr, Gaojing A1, Beijing 100123, Peoples R China.

[Zhang, Jianping] Civil Aviat Adm China, Res Inst 2, 2nd Ring Rd,South Sect 2,17, Chengdu 610041, Sichuan Provinc, Peoples R China.

[Xu, Xianggang] Civil Aviat Management Inst China, Huajiadi East Rd 3, Beijing 100102, Peoples R China.

RP Chen, ZL; Xu, XF (corresponding author), Civil Aviat Adm China, Civil Aviat Hosp, Civil Aviat Med Ctr, Gaojing A1, Beijing 100123, Peoples R China.

EM chenzhenling@camc-caac.cn; xuxianfa@hotmail.com

FU National Natural Science Foundation of ChinaNational Natural Science

Foundation of China (NSFC) [U1333132]; Civil Aviation Administration of

China [U1333132]; Major Science and Technology Programs of Civil

Aviation Administration of China [MHRD20140101]; Safety Foundation of

Civil Aviation Administration of China [TMSA 1608]

FX We acknowledge the financial support of the Joint Funds of the National

Natural Science Foundation of China and the Civil Aviation

Administration of China (No. U1333132), Major Science and Technology

Programs of Civil Aviation Administration of China (MHRD20140101), and

Safety Foundation of Civil Aviation Administration of China (TMSA 1608).

CR [Anonymous], 1946, 61 CAB

Caldwell J A, 2001, Air Med J, V20, P25

CAMERON C, 1973, ERGONOMICS, V16, P633, DOI 10.1080/00140137308924554

Capuron L, 2011, BIOL PSYCHIAT, V70, P175, DOI 10.1016/j.biopsych.2010.12.006

CHAMBERS EG, 1961, OCCUP PSYCHOL, V35, P44

Chen R, 2014, ACS NANO, V8, P2562, DOI 10.1021/nn406184r

Chen ZL, 2012, INTEGR BIOL-UK, V4, P1090, DOI 10.1039/c2ib00172a

Chen ZL, 2010, LANGMUIR, V26, P17790, DOI 10.1021/la103132m

Chen ZL, 2009, ANGEW CHEM INT EDIT, V48, P8303, DOI 10.1002/anie.200902708

Feng BS, 2014, ANAL CHEM, V86, P4164, DOI 10.1021/ac403310k

Frost DC, 2015, ANAL CHEM, V87, P1646, DOI 10.1021/ac503276z

Gaba DM, 2002, NEW ENGL J MED, V347, P1249, DOI 10.1056/NEJMsa020846

Gemperline E, 2016, ANAL CHEM, V88, P3422, DOI 10.1021/acs.analchem.5b02938

Gemperline E, 2014, ANAL CHEM, V86, P10030, DOI 10.1021/ac5028534

Grandjean E., 1979, OCCUP ENVIRON MED, V36, P175

Hancock PA, 1997, ACCIDENT ANAL PREV, V29, P495, DOI 10.1016/S0001-4575(97)00029-8

Huang PC, 2015, ANAL CHEM, V87, P6834, DOI 10.1021/acs.analchem.5b01155

Ji Q, 2004, IEEE T VEH TECHNOL, V53, P1052, DOI 10.1109/TVT.2004.830974

Lal SKL, 2002, PSYCHOPHYSIOLOGY, V39, P313, DOI 10.1017/S0048577201393095

Li LL, 2016, ANAL CHEM, V88, P6734, DOI 10.1021/acs.analchem.6b01008

Li XC, 2012, ANAL CHEM, V84, P5285, DOI 10.1021/ac300354z

Lin YQ, 2014, ANAL CHEM, V86, P3895, DOI 10.1021/ac4042087

Lindon JC, 2003, ANAL CHEM, V75, p384A, DOI 10.1021/ac031386+

Liu K, 2013, ANAL CHEM, V85, P9947, DOI 10.1021/ac402620c

Liu YQ, 2014, ANAL CHEM, V86, P7096, DOI 10.1021/ac501596v

Lockridge D, 1999, NEWPORTS ROADSTAR, V1, P1

Mapstone M, 2014, NAT MED, V20, P415, DOI 10.1038/nm.3466

Mishur RJ, 2012, MASS SPECTROM REV, V31, P70, DOI 10.1002/mas.20338

Nealley MA, 2015, INT J AVIAT PSYCHOL, V25, P14, DOI 10.1080/10508414.2015.981488

Phan NTN, 2016, ANAL CHEM, V88, P1734, DOI 10.1021/acs.analchem.5b03942

Salazar G. J, 2007, OK07193 FAA CIV AER

Silber BY, 2010, NEUROSCI BIOBEHAV R, V34, P387, DOI 10.1016/j.neubiorev.2009.08.005

Thakkar MM, 2011, SLEEP MED REV, V15, P65, DOI 10.1016/j.smrv.2010.06.004

Wang GY, 2004, CHRONOBIOL INT, V21, P229, DOI 10.1081/CBI-120037822

Wang J, 2014, ANAL CHEM, V86, P4515, DOI 10.1021/ac500443q

Wang XY, 2014, J MATER SCI, V49, P4394, DOI 10.1007/s10853-014-8148-9

Wang X, 2014, ANAL CHEM, V86, P4739, DOI 10.1021/ac500382x

Zhang YD, 2015, ANAL CHEM, V87, P6505, DOI 10.1021/acs.analchem.5b01272

Zheng WP, 2002, CURR MED CHEM, V9, P1187, DOI 10.2174/0929867023370013

NR 39

TC 8

Z9 11

U1 3

U2 63

PU AMER CHEMICAL SOC

PI WASHINGTON

PA 1155 16TH ST, NW, WASHINGTON, DC 20036 USA

SN 0003-2700

EI 1520-6882

J9 ANAL CHEM

JI Anal. Chem.

PD DEC 6

PY 2016

VL 88

IS 23

BP 11293

EP 11296

DI 10.1021/acs.analchem.6b03421

PG 4

WC Chemistry, Analytical

WE Science Citation Index Expanded (SCI-EXPANDED); Social Science Citation Index (SSCI)

SC Chemistry

GA EE4FE

UT WOS:000389556900006

PM 27934122

OA Bronze

DA 2022-05-04

ER

PT J

AU Hogan, PS

Chen, SX

Teh, WW

Chib, VS

AF Hogan, Patrick S.

Chen, Steven X.

Teh, Wen Wen

Chib, Vikram S.

TI Neural mechanisms underlying the effects of physical fatigue on

effort-based choice

SO NATURE COMMUNICATIONS

LA English

DT Article

ID MOTOR EVOKED-POTENTIALS; BRAIN; VALUATION; NEUROSCIENCE; DEPRESSION;

ACTIVATION; CORTEX

AB Physical fatigue crucially influences our decisions to partake in effortful action. However, there is a limited understanding of how fatigue impacts effort-based decision-making at the level of brain and behavior. We use functional magnetic resonance imaging to record markers of brain activity while human participants engage in uncertain choices for prospective physical effort, before and after bouts of exertion. Using computational modeling of choice behavior we find that fatiguing exertions cause participants to increase their subjective cost of effort, compared to a baseline/rested state. We describe a mechanism by which signals related to motor cortical state in premotor cortex influence effort value computations, instantiated by insula, thereby increasing an individual's subjective valuation of prospective physical effort while fatigued. Our findings provide a neurobiological account of how information about bodily state modulates decisions to engage in physical activity. Fatigue influences our choices to engage in physical activity. Here, the authors investigate the underlying cognitive and neuronal mechanisms by which fatigue influences decisions to exert, and show that information about motor cortical state modulates decisions to engage in physical activity.

C1 [Hogan, Patrick S.; Chen, Steven X.; Teh, Wen Wen; Chib, Vikram S.] Johns Hopkins Sch Med, Dept Biomed Engn, Baltimore, MD 21205 USA.

[Chib, Vikram S.] Johns Hopkins Univ, Kavli Neurosci Discovery Inst, Baltimore, MD 21205 USA.

[Chib, Vikram S.] Kennedy Krieger Inst, 707 North Broadway, Baltimore, MD 21205 USA.

RP Chib, VS (corresponding author), Johns Hopkins Sch Med, Dept Biomed Engn, Baltimore, MD 21205 USA.; Chib, VS (corresponding author), Johns Hopkins Univ, Kavli Neurosci Discovery Inst, Baltimore, MD 21205 USA.; Chib, VS (corresponding author), Kennedy Krieger Inst, 707 North Broadway, Baltimore, MD 21205 USA.

EM vchib@jhu.edu

FU Eunice Kennedy Shriver National Institute of Child Health & Human

Development of the National Institutes of Health [R01HD097619]; National

Institutes of Mental HealthUnited States Department of Health & Human

ServicesNational Institutes of Health (NIH) - USANIH National Institute

of Mental Health (NIMH) [R56MH113627, R01MH119086]

FX This work was supported by the Eunice Kennedy Shriver National Institute

of Child Health & Human Development of the National Institutes of Health

under Award Number R01HD097619 and the National Institutes of Mental

Health under Award Numbers R56MH113627 and R01MH119086 to V.S.C.

CR Ahn Woo-Young, 2017, Comput Psychiatr, V1, P24, DOI 10.1162/CPSY_a_00002

Ainley V, 2016, PHILOS T R SOC B, V371, DOI 10.1098/rstb.2016.0003

Aridan N, 2019, NEUROIMAGE, V185, P446, DOI 10.1016/j.neuroimage.2018.10.051

Arulpragasam AR, 2018, P NATL ACAD SCI USA, V115, pE5233, DOI 10.1073/pnas.1800444115

BARON RM, 1986, J PERS SOC PSYCHOL, V51, P1173, DOI 10.1037/0022-3514.51.6.1173

Benwell NM, 2006, EXP BRAIN RES, V175, P575, DOI 10.1007/s00221-006-0573-9

Benwell NM, 2005, EXP BRAIN RES, V167, P160, DOI 10.1007/s00221-005-0013-2

Betancourt M., 2015, CURRENT TRENDS BAYES, DOI [10.1201/b18502, DOI 10.1201/B.18502-5]

Blain B, 2019, CURR BIOL, V29, P3289, DOI 10.1016/j.cub.2019.08.054

Blain B, 2016, P NATL ACAD SCI USA, V113, P6967, DOI 10.1073/pnas.1520527113

Bonnelle V, 2016, CEREB CORTEX, V26, P807, DOI 10.1093/cercor/bhv247

Brainard DH, 1997, SPATIAL VISION, V10, P433, DOI 10.1163/156856897X00357

BRASILNETO J, 1993, EXP BRAIN RES, V93, P181

Camerer C, 2005, J ECON LIT, V43, P9, DOI 10.1257/0022051053737843

Carpenter B, 2017, J STAT SOFTW, V76, P1, DOI 10.18637/jss.v076.i01

Chaudhuri A, 2004, LANCET, V363, P978, DOI 10.1016/S0140-6736(04)15794-2

Chong TTJ, 2017, PLOS BIOL, V15, DOI 10.1371/journal.pbio.1002598

Craig AD, 2003, CURR OPIN NEUROBIOL, V13, P500, DOI 10.1016/S0959-4388(03)00090-4

Critchley HD, 2004, NAT NEUROSCI, V7, P189, DOI 10.1038/nn1176

Croxson PL, 2009, J NEUROSCI, V29, P4531, DOI 10.1523/JNEUROSCI.4515-08.2009

Deichmann R, 2003, NEUROIMAGE, V19, P430, DOI 10.1016/S1053-8119(03)00073-9

Gorgolewski KJ, 2015, FRONT NEUROINFORM, V9, DOI 10.3389/fninf.2015.00008

Hogan PS, 2019, CEREB CORTEX, V29, P4277, DOI 10.1093/cercor/bhy310

JUDD CM, 1981, EVALUATION REV, V5, P602, DOI 10.1177/0193841X8100500502

Klein-Flugge MC, 2016, J NEUROSCI, V36, P10002, DOI 10.1523/JNEUROSCI.0292-16.2016

Kriegeskorte N, 2009, NAT NEUROSCI, V12, P535, DOI 10.1038/nn.2303

Kuppuswamy A, 2017, BRAIN, V140, P2240, DOI 10.1093/brain/awx153

Kurniawan IT, 2013, J NEUROSCI, V33, P6160, DOI 10.1523/JNEUROSCI.4777-12.2013

Le Bouc R, 2016, J NEUROSCI, V36, P6623, DOI 10.1523/JNEUROSCI.3078-15.2016

Lieberman MD, 2009, SOC COGN AFFECT NEUR, V4, P423, DOI 10.1093/scan/nsp052

Liu JZ, 2002, BRAIN RES, V957, P320, DOI 10.1016/S0006-8993(02)03665-X

LOSCHER WN, 1993, EUR J APPL PHYSIOL O, V67, P99, DOI 10.1007/BF00376651

MERLETTI R, 1990, J APPL PHYSIOL, V69, P1810, DOI 10.1152/jappl.1990.69.5.1810

Merletti R., 2016, SURFACE ELECTROMYOGR, P273, DOI [10.1002/9781119082934.ch10, DOI 10.1002/9781119082934.CH10]

Meyniel F, 2014, PLOS COMPUT BIOL, V10, DOI 10.1371/journal.pcbi.1003584

Meyniel F, 2013, P NATL ACAD SCI USA, V110, P2641, DOI 10.1073/pnas.1211925110

Morel P, 2017, PLOS BIOL, V15, DOI 10.1371/journal.pbio.2001323

Pessiglione M, 2018, BRAIN, V141, P629, DOI 10.1093/brain/awx278

Prevost C, 2010, J NEUROSCI, V30, P14080, DOI 10.1523/JNEUROSCI.2752-10.2010

Rangel A, 2008, NAT REV NEUROSCI, V9, P545, DOI 10.1038/nrn2357

Samii A, 1996, NEUROLOGY, V46, P1376, DOI 10.1212/WNL.46.5.1376

Shadmehr R, 2016, CURR BIOL, V26, P1929, DOI 10.1016/j.cub.2016.05.065

Skvortsova V, 2014, J NEUROSCI, V34, P15621, DOI 10.1523/JNEUROSCI.1350-14.2014

Stephan KE, 2016, FRONT HUM NEUROSCI, V10, DOI 10.3389/fnhum.2016.00550

van der Schaaf ME, 2018, BIOL PSYCHIAT-COGN N, V3, P392, DOI 10.1016/j.bpsc.2018.01.015

van Duinen H, 2007, NEUROIMAGE, V35, P1438, DOI 10.1016/j.neuroimage.2007.02.008

Yoon T, 2018, P NATL ACAD SCI USA, V115, pE10476, DOI 10.1073/pnas.1812979115

ZIPP P, 1982, EUR J APPL PHYSIOL O, V50, P41, DOI 10.1007/BF00952243

NR 48

TC 4

Z9 4

U1 3

U2 6

PU NATURE PUBLISHING GROUP

PI LONDON

PA MACMILLAN BUILDING, 4 CRINAN ST, LONDON N1 9XW, ENGLAND

SN 2041-1723

J9 NAT COMMUN

JI Nat. Commun.

PD AUG 12

PY 2020

VL 11

IS 1

AR 4026

DI 10.1038/s41467-020-17855-5

PG 15

WC Multidisciplinary Sciences

WE Science Citation Index Expanded (SCI-EXPANDED); Social Science Citation Index (SSCI)

SC Science & Technology - Other Topics

GA NF8SV

UT WOS:000563563800003

PM 32788604

OA gold, Green Published

DA 2022-05-04

ER

PT J

AU Krigolson, OE

Hammerstrom, MR

Abimbola, W

Trska, R

Wright, BW

Hecker, KG

Binsted, G

AF Krigolson, Olave E.

Hammerstrom, Mathew R.

Abimbola, Wande

Trska, Robert

Wright, Bruce W.

Hecker, Kent G.

Binsted, Gordon

TI Using Muse: Rapid Mobile Assessment of Brain Performance

SO FRONTIERS IN NEUROSCIENCE

LA English

DT Article

DE EEG; ERP; fatigue; cognitive fatigue; performance; health; mobile EEG

ID EEG ALPHA POWER; MENTAL FATIGUE; DRIVER FATIGUE; COGNITIVE CONTROL;

SLEEP; DROWSINESS; ELECTROENCEPHALOGRAPHY; OSCILLATIONS; ATTENTION;

ALERTNESS

AB The advent of mobile electroencephalography (mEEG) has created a means for large scale collection of neural data thus affording a deeper insight into cognitive phenomena such as cognitive fatigue. Cognitive fatigue - a neural state that is associated with an increased incidence of errorful performance - is responsible for accidents on a daily basis which at times can cost human lives. To gain better insight into the neural signature of cognitive fatigue in the present study we used mEEG to examine the relationship between perceived cognitive fatigue and human-event related brain potentials (ERPs) and electroencephalographic (EEG) oscillations in a sample of 1,000 people. As a secondary goal, we wanted to further demonstrate the capability of mEEG to accurately measure ERP and EEG data. To accomplish these goals, participants performed a standard visual oddball task on an Apple iPad while EEG data were recorded from a Muse EEG headband. Counter to traditional EEG studies, experimental setup and data collection was completed in less than seven minutes on average. An analysis of our EEG data revealed robust N200 and P300 ERP components and neural oscillations in the delta, theta, alpha, and beta bands. In line with previous findings we observed correlations between ERP components and EEG power and perceived cognitive fatigue. Further, we demonstrate here that a linear combination of ERP and EEG features is a significantly better predictor of perceived cognitive fatigue than any ERP or EEG feature on its own. In sum, our results provide validation of mEEG as a viable tool for research and provide further insight into the impact of cognitive fatigue on the human brain.

C1 [Krigolson, Olave E.; Hammerstrom, Mathew R.; Abimbola, Wande; Trska, Robert] Univ Victoria, Ctr Biomed Res, Victoria, BC, Canada.

[Wright, Bruce W.] Univ Victoria, Div Med Sci, Victoria, BC, Canada.

[Hecker, Kent G.] Univ Calgary, Fac Vet Med, Calgary, AB, Canada.

[Binsted, Gordon] Univ British Columbia Okanagan, Fac Hlth & Social Dev, Kelowna, BC, Canada.

RP Krigolson, OE (corresponding author), Univ Victoria, Ctr Biomed Res, Victoria, BC, Canada.

EM krigolson@uvic.ca

FU NSERCNatural Sciences and Engineering Research Council of Canada (NSERC)

[RGPIN 2016-0943]; NSERC CRD Grant [CRDPJ 530889-18]

FX This research was funded by NSERC Discovery Grant RGPIN 2016-0943 and

NSERC CRD Grant CRDPJ 530889-18 awarded to OK.

CR Abiri R, 2019, J NEURAL ENG, V16, DOI 10.1088/1741-2552/aaf12e

Aeschbach D, 1997, NEUROSCI LETT, V239, P121, DOI 10.1016/S0304-3940(97)00904-X

Ahsberg E, 1997, INT J IND ERGONOM, V20, P121, DOI 10.1016/S0169-8141(96)00044-3

Aidman E, 2015, ACCIDENT ANAL PREV, V81, P8, DOI 10.1016/j.aap.2015.03.041

AKERSTEDT T, 1991, SCAND J WORK ENV HEA, V17, P330, DOI 10.5271/sjweh.1694

Arnau S, 2017, INT J PSYCHOPHYSIOL, V117, P17, DOI 10.1016/j.ijpsycho.2017.04.003

Baranski JV, 2007, J EXP PSYCHOL-APPL, V13, P182, DOI 10.1037/1076-898X.13.4.182

Bartlett PL, 2020, P NATL ACAD SCI USA, V117, P30063, DOI 10.1073/pnas.1907378117

Belz SM, 2004, HUM FACTORS, V46, P154, DOI 10.1518/hfes.46.1.154.30393

Boksem MAS, 2005, COGNITIVE BRAIN RES, V25, P107, DOI 10.1016/j.cogbrainres.2005.04.011

Borghini G, 2012, IEEE ENG MED BIO, P6442, DOI 10.1109/EMBC.2012.6347469

Borghini G, 2014, NEUROSCI BIOBEHAV R, V44, P58, DOI 10.1016/j.neubiorev.2012.10.003

Broadway JM, 2015, BIOL PSYCHOL, V107, P31, DOI 10.1016/j.biopsycho.2015.02.009

Buysse Daniel J, 2003, Sleep, V26, P218

Cajochen C, 1996, NEUROSCI LETT, V207, P209, DOI 10.1016/0304-3940(96)12517-9

Cajochen C, 1995, SLEEP, V18, P890, DOI 10.1093/sleep/18.10.890

Caldwell JA, 2002, INT J AVIAT PSYCHOL, V12, P19, DOI 10.1207/S15327108IJAP1201_3

Cammu H, 2012, EUR J CONTRACEP REPR, V17, P314, DOI 10.3109/13625187.2012.672664

Campagne A, 2004, PHYSIOL BEHAV, V80, P515, DOI 10.1016/j.physbeh.2003.10.004

Cao R, 2014, BIO-MED MATER ENG, V24, P2927, DOI 10.3233/BME-141112

Clayton MS, 2015, TRENDS COGN SCI, V19, P188, DOI 10.1016/j.tics.2015.02.004

Cohen J., 1988, STAT POWER ANAL BEHA, V2nd ed., P1, DOI DOI 10.4324/9780203771587

Cohen MX, 2008, BRAIN RES, V1238, P127, DOI 10.1016/j.brainres.2008.07.114

Cohen MX, 2014, ISS CLIN COGN NEUROP, P1

Craig A, 2012, PSYCHOPHYSIOLOGY, V49, P574, DOI 10.1111/j.1469-8986.2011.01329.x

Debener S, 2012, PSYCHOPHYSIOLOGY, V49, P1617, DOI 10.1111/j.1469-8986.2012.01471.x

Delorme A, 2004, J NEUROSCI METH, V134, P9, DOI 10.1016/j.jneumeth.2003.10.009

Dinges DF, 1997, SLEEP, V20, P267

Dorrian J, 2011, APPL ERGON, V42, P202, DOI 10.1016/j.apergo.2010.06.009

Dumont M., 1997, SLEEP RES, V26, P712

Eoh HJ, 2005, INT J IND ERGONOM, V35, P307, DOI 10.1016/j.ergon.2004.09.006

Fickling SD, 2020, IEEE T BIO-MED ENG, V67, P2916, DOI 10.1109/TBME.2020.2973617

Fickling SD, 2019, BRAIN, V142, P255, DOI 10.1093/brain/awy317

Fletcher A, 2005, AUST NZ J PUBL HEAL, V29, P471, DOI 10.1111/j.1467-842X.2005.tb00229.x

Fonseca A, 2018, FRONT HUM NEUROSCI, V12, DOI 10.3389/fnhum.2018.00418

Foxe JJ, 2011, FRONT PSYCHOL, V2, DOI 10.3389/fpsyg.2011.00154

Gaba DM, 2002, NEW ENGL J MED, V347, P1249, DOI 10.1056/NEJMsa020846

Gharagozlou F, 2015, IRAN J PUBLIC HEALTH, V44, P1693

Goode JH, 2003, J SAFETY RES, V34, P309, DOI 10.1016/S0022-4375(03)00033-1

Guntekin B, 2010, COGN NEURODYNAMICS, V4, P107, DOI 10.1007/s11571-010-9106-0

Hajra SG, 2016, FRONT NEUROSCI-SWITZ, V10, DOI 10.3389/fnins.2016.00211

Hopstaken JF, 2015, PSYCHOPHYSIOLOGY, V52, P305, DOI 10.1111/psyp.12339

Jackson CE, 2008, ALZHEIMERS DEMENT, V4, pS137, DOI 10.1016/j.jalz.2007.10.008

Jin CY, 2019, COGN AFFECT BEHAV NE, V19, P1059, DOI 10.3758/s13415-019-00707-1

Kathner I, 2014, BIOL PSYCHOL, V102, P118, DOI 10.1016/j.biopsycho.2014.07.014

Kaseda Y, 1998, J NEUROL SCI, V158, P96, DOI 10.1016/S0022-510X(98)00100-2

Kato Y, 2009, INT J PSYCHOPHYSIOL, V72, P204, DOI 10.1016/j.ijpsycho.2008.12.008

KECKLUND G, 1993, ERGONOMICS, V36, P1007, DOI 10.1080/00140139308967973

Klimesch W, 2012, TRENDS COGN SCI, V16, P606, DOI 10.1016/j.tics.2012.10.007

Kotowski K, 2019, BIOCYBERN BIOMED ENG, V39, P1036, DOI 10.1016/j.bbe.2019.09.002

Krigolson OE, 2017, FRONT NEUROSCI-SWITZ, V11, DOI 10.3389/fnins.2017.00109

Lal SKL, 2002, PSYCHOPHYSIOLOGY, V39, P313, DOI 10.1017/S0048577201393095

Lamti HA, 2016, COMPUT METHOD BIOMEC, V19, P1749, DOI 10.1080/10255842.2016.1183198

Lin CT, 2008, IEEE T BIO-MED ENG, V55, P1582, DOI 10.1109/TBME.2008.918566

Lin CT, 2005, IEEE T CIRCUITS-I, V52, P2726, DOI 10.1109/TCSI.2005.857555

Lorist MM, 2005, COGNITIVE BRAIN RES, V24, P199, DOI 10.1016/j.cogbrainres.2005.01.018

Luck SJ, 2014, INTRODUCTION TO THE EVENT-RELATED POTENTIAL TECHNIQUE, 2ND EDITION, P1

Macchi MM, 2002, ACCIDENT ANAL PREV, V34, P825, DOI 10.1016/S0001-4575(01)00089-6

Mathewson KE, 2012, PSYCHOPHYSIOLOGY, V49, P1558, DOI 10.1111/j.1469-8986.2012.01474.x

Mercado-Aguirre IM, 2019, SYMP IMAG SIG PROC A

Mizuno K, 2011, BEHAV BRAIN FUNCT, V7, DOI 10.1186/1744-9081-7-17

OKEN BS, 1986, ANN NEUROL, V19, P493, DOI 10.1002/ana.410190511

Pal NR, 2008, EURASIP J ADV SIG PR, DOI 10.1155/2008/519480

Papadelis Christos, 2006, Conf Proc IEEE Eng Med Biol Soc, V2006, P6201

Phipps-Nelson J, 2011, J SLEEP RES, V20, P404, DOI 10.1111/j.1365-2869.2010.00900.x

Picot A, 2012, MACH VISION APPL, V23, P1195, DOI 10.1007/s00138-011-0374-4

Qin Y, 2016, COGN NEURODYNAMICS, V10, P275, DOI 10.1007/s11571-016-9378-0

Qiu JM, 2019, FRONT HUM NEUROSCI, V13, DOI 10.3389/fnhum.2019.00258

Raduntz T, 2018, FRONT PHYSIOL, V9, DOI 10.3389/fphys.2018.00098

SANTAMARIA J, 1987, J CLIN NEUROPHYSIOL, V4, P327, DOI 10.1097/00004691-198710000-00002

Sauseng P, 2007, NEUROSCIENCE, V146, P1435, DOI 10.1016/j.neuroscience.2007.03.014

Sauseng P, 2005, EUR J NEUROSCI, V22, P2917, DOI 10.1111/j.1460-9568.2005.04482.x

Scammell TE, 2017, NEURON, V93, P747, DOI 10.1016/j.neuron.2017.01.014

Scanlon JEM, 2020, INT J PSYCHOPHYSIOL, V151, P59, DOI 10.1016/j.ijpsycho.2020.02.016

Schier MA, 2000, INT J PSYCHOPHYSIOL, V37, P155, DOI 10.1016/S0167-8760(00)00079-9

Schmidt EA, 2009, ACCIDENT ANAL PREV, V41, P1087, DOI 10.1016/j.aap.2009.06.007

Strijkstra AM, 2003, NEUROSCI LETT, V340, P17, DOI 10.1016/S0304-3940(03)00033-8

Stuster J., 2010, REV ANAL ASTRONAUT J

Tabachnick B.G., 2018, USING MULTIVARIATE S, V7th

Tanaka H, 1997, SLEEP, V20, P523, DOI 10.1093/sleep/20.7.523

TORSVALL L, 1988, INT J NEUROSCI, V38, P435, DOI 10.3109/00207458808990704

Tran Y, 2020, PSYCHOPHYSIOLOGY, V57, DOI 10.1111/psyp.13554

Trejo L. J., 2015, PSYCHOLOGY, V6, P572, DOI [10.4236/psych.2015.65055, DOI 10.4236/PSYCH.2015.65055]

Trejo LJ, 2005, PROC SPIE, V5797, P105, DOI 10.1117/12.604286

Tshiluna NB, 2016, 2016 THIRD INTERNATIONAL CONFERENCE ON ADVANCES IN COMPUTING, COMMUNICATION AND ENGINEERING (ICACCE 2016), P13, DOI 10.1109/ICACCE.2016.8073716

Uetake A, 2000, IEEE RO-MAN 2000: 9TH IEEE INTERNATIONAL WORKSHOP ON ROBOT AND HUMAN INTERACTIVE COMMUNICATION, PROCEEDINGS, P235, DOI 10.1109/ROMAN.2000.892501

Vejvoda M, 2014, J SLEEP RES, V23, P564, DOI 10.1111/jsr.12186

Wang CM, 2012, J NEURAL ENG, V9, DOI 10.1088/1741-2560/9/5/056013

Wijesuriya N, 2007, INT J PSYCHOPHYSIOL, V63, P77, DOI 10.1016/j.ijpsycho.2006.08.005

Zhao CL, 2012, ACCIDENT ANAL PREV, V45, P83, DOI 10.1016/j.aap.2011.11.019

NR 90

TC 1

Z9 1

U1 4

U2 7

PU FRONTIERS MEDIA SA

PI LAUSANNE

PA AVENUE DU TRIBUNAL FEDERAL 34, LAUSANNE, CH-1015, SWITZERLAND

EI 1662-453X

J9 FRONT NEUROSCI-SWITZ

JI Front. Neurosci.

PD JAN 28

PY 2021

VL 15

AR 634147

DI 10.3389/fnins.2021.634147

PG 11

WC Neurosciences

WE Science Citation Index Expanded (SCI-EXPANDED)

SC Neurosciences & Neurology

GA QF4WI

UT WOS:000616895800001

PM 33584194

OA gold, Green Published

DA 2022-05-04

ER

PT J

AU Sasahara, I

Fujimura, N

Nozawa, Y

Furuhata, Y

Sato, H

AF Sasahara, Ikuko

Fujimura, Naoko

Nozawa, Yoshizu

Furuhata, Yasufumi

Sato, Hitoshi

TI The effect of histidine on mental fatigue and cognitive performance in

subjects with high fatigue and sleep disruption scores

SO PHYSIOLOGY & BEHAVIOR

LA English

DT Article

DE Histidine; Mental fatigue; POMS; Cognitive performance; CogState

ID HISTAMINERGIC NEURON SYSTEM; AMINO-ACID-TRANSPORT; DRIED-BONITO BROTH;

BRAIN HISTAMINE; MOOD STATES; BLOOD; ROLES; MICE; H1; DECARBOXYLASE

AB Our previous study reported that a dried bonito broth known in japan as 'dashi' improved or ameliorated mood states, including fatigue, during the daily lives of human subjects. Histidine is an amino acid that is present in dried bonito broth, and we sought to evaluate whether histidine would affect feelings of fatigue in humans. We investigated the effects of histidine intake on the feeling of fatigue, mood states and mental task performance by performing a placebo-controlled, double-blind crossover trial. Twenty subjects with high fatigue and sleep disruption scores were asked to ingest histidine or a placebo every day for two weeks. The subjects' mood states were evaluated using the Profile of Mood States (POMS) scale and a visual analog scale (VAS) for eight feelings (fatigue, depression, carelessness, drowsiness, clear thinking, motivation, attentiveness and concentration). We also measured subjects' cognitive performance using the CogHealth test battery. The fatigue T-scores on the POMS test decreased significantly following histidine ingestion compared to placebo ingestion (p < 0.05). After two weeks of histidine ingestion, the reaction time for the working memory task in the CogHealth test battery was significantly shorten compared to placebo ingestion. The VAS scores for clear thinking and for attentiveness were increased significantly following histidine ingestion compared to placebo ingestion (p < 0.05). These results suggest that daily ingestion of histidine may ameliorate feelings of fatigue, increase performance during working memory tasks, and improve the clear thinking and attentiveness. (C) 2015 The Authors. Published by Elsevier Inc.

C1 [Sasahara, Ikuko; Fujimura, Naoko; Nozawa, Yoshizu; Furuhata, Yasufumi] Ajinomoto Co Inc, Inst Food Sci & Technol, Food Prod Div, Kawasaki, Kanagawa 2108681, Japan.

[Sato, Hitoshi] Ajinomoto Co Inc, Hlth & Wellness Business Dept, Amino Sci Div, Kawasaki, Kanagawa 2108681, Japan.

RP Sasahara, I (corresponding author), Ajinomoto Co Inc, Inst Food Sci & Technol, Food Prod Div, 1-1 Suzuki Cho, Kawasaki, Kanagawa 2108681, Japan.

EM ikuko_sasahara@ajinomoto.com

CR Arnold LM, 2008, PSYCHOSOMATICS, V49, P185, DOI 10.1176/appi.psy.49.3.185

Baddeley A, 2010, CURR BIOL, V20, pR136, DOI 10.1016/j.cub.2009.12.014

Belenky G, 2003, J SLEEP RES, V12, P1, DOI 10.1046/j.1365-2869.2003.00337.x

Boksem MAS, 2008, BRAIN RES REV, V59, P125, DOI 10.1016/j.brainresrev.2008.07.001

Boksem MAS, 2005, COGNITIVE BRAIN RES, V25, P107, DOI 10.1016/j.cogbrainres.2005.04.011

Brown RE, 2001, PROG NEUROBIOL, V63, P637, DOI 10.1016/S0301-0082(00)00039-3

Brown RF, 2006, J PSYCHOSOM RES, V60, P585, DOI 10.1016/j.jpsychores.2006.05.001

BUYSSE DJ, 1989, PSYCHIAT RES, V28, P193, DOI 10.1016/0165-1781(89)90047-4

Dai HM, 2005, PSYCHOPHARMACOLOGY, V183, P285, DOI 10.1007/s00213-005-0203-4

Dai HM, 2007, NEUROSCI RES, V57, P306, DOI 10.1016/j.neures.2006.10.020

Darsow U, 2000, J INVEST DERMATOL, V115, P1029, DOI 10.1046/j.1523-1747.2000.00193.x

DESPOSITO M, 1995, NATURE, V378, P279, DOI 10.1038/378279a0

Evengard B, 1998, PAIN, V78, P153, DOI 10.1016/S0304-3959(98)00134-1

Falleti MG, 2003, J SLEEP RES, V12, P265, DOI 10.1111/j.1365-2869.2003.00363.x

FUKE S, 1991, PHYSIOL BEHAV, V49, P863, DOI 10.1016/0031-9384(91)90195-T

Furuta T, 1996, DRUG METAB DISPOS, V24, P49

Haas H, 2003, NAT REV NEUROSCI, V4, P121, DOI 10.1038/nrn1034

Haas HL, 2008, PHYSIOL REV, V88, P1183, DOI 10.1152/physrev.00043.2007

Halbach MM, 2003, AM J OBSTET GYNECOL, V188, P1198, DOI 10.1067/mob.2003.306

HARGREAVES KM, 1988, J BIOL CHEM, V263, P19392

Hirano Y, 2008, NEUROSCI LETT, V436, P189, DOI 10.1016/j.neulet.2008.03.033

Ikegami K, 2009, J OCCUP HEALTH, V51, P412, DOI 10.1539/joh.L8127

Ishizaki T., 2005, J STUDY DIETARY HABI, V16, P39, DOI [10.2740/jisdh.16.39, DOI 10.2740/JISDH.16.39]

Ishizaki T, 2006, J JPN SOC FOOD SCI, V53, P225, DOI 10.3136/nskkk.53.225

Ito C, 2000, BIOMED PHARMACOTHER, V54, P263, DOI 10.1016/S0753-3322(00)80069-4

Ito C, 1999, NEUROSCI LETT, V262, P143, DOI 10.1016/S0304-3940(99)00052-X

Japanese Society of Fatigue Science, 2013, ANT CLIN EV GUID

Kano M, 2004, EUR J NEUROSCI, V20, P803, DOI 10.1111/j.1460-9568.2004.03540.x

Keeler JF, 2011, BIOCHEM PHARMACOL, V81, P1356, DOI 10.1016/j.bcp.2010.12.028

Kuroda M, 2008, BIOMED RES-TOKYO, V29, P175, DOI 10.2220/biomedres.29.175

Kuroda M, 2007, PHYSIOL BEHAV, V92, P957, DOI 10.1016/j.physbeh.2007.07.002

Markus R, 2000, PHYSIOL BEHAV, V70, P333, DOI 10.1016/S0031-9384(00)00265-1

Maruff P, 2005, J SLEEP RES, V14, P21, DOI 10.1111/j.1365-2869.2004.00438.x

Miczek KA, 2001, BEHAV BRAIN RES, V125, P167, DOI 10.1016/S0166-4328(01)00298-4

Midoh N, 2009, J HEALTH SCI, V55, P56, DOI 10.1248/jhs.55.56

Miller EK, 2001, ANNU REV NEUROSCI, V24, P167, DOI 10.1146/annurev.neuro.24.1.167

Nozawa Y, 2008, PHYSIOL BEHAV, V93, P267, DOI 10.1016/j.physbeh.2007.08.021

Ono Y, 2009, NEUROSCI RES, V64, P385, DOI 10.1016/j.neures.2009.04.011

Panula P, 1998, NEUROSCIENCE, V82, P993

PARDRIDGE WM, 1986, NUTR REV, V44, P15

Parmentier R, 2002, J NEUROSCI, V22, P7695

SMITH QR, 1987, J NEUROCHEM, V49, P1651, DOI 10.1111/j.1471-4159.1987.tb01039.x

Surani S., 2014, J PATIENT SAF

Tashiro M, 2005, HUM PSYCHOPHARM CLIN, V20, P501, DOI 10.1002/hup.713

Tashiro M, 2002, LIFE SCI, V72, P409, DOI 10.1016/S0024-3205(02)02276-2

TEWS JK, 1983, AM J PHYSIOL, V245, pR556, DOI 10.1152/ajpregu.1983.245.4.R556

Thomsen DK, 2005, PERS INDIV DIFFER, V38, P1935, DOI 10.1016/j.paid.2004.12.001

van der Linden D, 2003, ACTA PSYCHOL, V113, P45, DOI 10.1016/S0001-6918(02)00150-6

Watanabe T, 2001, TOHOKU J EXP MED, V195, P197, DOI 10.1620/tjem.195.197

WATANABE T, 1984, BRAIN RES, V295, P13, DOI 10.1016/0006-8993(84)90811-4

Yanai K, 2007, PHARMACOL THERAPEUT, V113, P1, DOI 10.1016/j.pharmthera.2006.06.008

Yokoyama K., 1994, JAPANESE EDITION POM

NR 52

TC 34

Z9 34

U1 2

U2 17

PU PERGAMON-ELSEVIER SCIENCE LTD

PI OXFORD

PA THE BOULEVARD, LANGFORD LANE, KIDLINGTON, OXFORD OX5 1GB, ENGLAND

SN 0031-9384

J9 PHYSIOL BEHAV

JI Physiol. Behav.

PD AUG 1

PY 2015

VL 147

BP 238

EP 244

DI 10.1016/j.physbeh.2015.04.042

PG 7

WC Psychology, Biological; Behavioral Sciences

WE Science Citation Index Expanded (SCI-EXPANDED); Social Science Citation Index (SSCI)

SC Psychology; Behavioral Sciences

GA CL1XB

UT WOS:000356737100033

PM 25921948

OA hybrid

DA 2022-05-04

ER

PT J

AU Munoz-de-Escalona, E

Canas, JJ

Noriega, P

AF Munoz-de-Escalona, Enrique

Canas, Jose J.

Noriega, Paulo

TI Inconsistencies between mental fatigue measures under compensatory

control theories

SO PSICOLOGICA

LA English

DT Article

ID COGNITIVE FATIGUE; PUPIL-SIZE; PERFORMANCE; WORKLOAD; LOAD

AB Mental fatigue has traditionally been defined as a condition of reduced cognitive efficiency and performance, accompanied by a subjective feeling of fatigue. Even though we could expect to find associations between the three defining characteristic of mental fatigue (performance impairment, physiological deactivation and subjective fatigue), research has shown that the emergence of inconsistencies between measures is more frequent than one might expect: people proved capable of maintaining adequate performance levels even after having declared themselves fatigued. This could be explained under the compensatory control mechanism models, which state that humans are able to provide additional resources under demanding conditions, but only at the expense of psychophysiological cost and subjective fatigue. We tested this explanation by manipulating task complexity and time performing a simulated air-traffic control task. We collected psychophysiological, performance and subjective data. A decrease in pupil size was seen in the low-aircraft-density condition, while pupil size remained constant in the high-aircraft-density condition. Participants' task performance was optimal in both conditions, though they showed an increase in subjective feelings of fatigue, especially in the high-complexity task condition. Thus, complexity seemed to trigger compensatory mechanisms, which reallocated extra resources that physiologically activated participants in order to deal with a higher complexity task, whereas subjective fatigue could be acting as a signal to the organism of impending resource depletion. Our findings support compensatory control theories and offer an explanation of inconsistencies between fatigue measures. Further research on compensatory mechanisms is needed to enable better management of fatigue effects to prevent work-related accidents.

C1 [Munoz-de-Escalona, Enrique; Canas, Jose J.] Univ Granada, Dept Expt Psychol, Cognit Ergon Grp, Mind Brain & Behav Res Ctr CIMCYC, Granada, Spain.

[Noriega, Paulo] Univ Lisbon, Fac Arquitetura, CIAUD, ErgoUX Lab, Lisbon, Portugal.

RP Munoz-de-Escalona, E (corresponding author), Univ Granada, Dept Expt Psychol, Campus Cartuja S-N, Granada 18071, Spain.

EM enriquemef@ugr.es

RI ; Noriega, Paulo/D-2470-2017

OI Canas Delgado, Jose Juan/0000-0002-0666-2123; Munoz-de-Escalona

Fernandez, Enrique/0000-0002-1775-3477; Noriega,

Paulo/0000-0002-0433-6201

FU Spanish Ministry of IndustrySpanish Government [PI-1461/2015]

FX This research has been funded by Project AIRPORTS from the Spanish

Ministry of Industry, Grant number: PI-1461/2015.

CR Ackerman PL., 2011, COGNITIVE FATIGUE MU, P189, DOI DOI 10.1037/12343-009

Ahlstrom U, 2006, INT J IND ERGONOM, V36, P623, DOI 10.1016/j.ergon.2006.04.002

Bailey A, 2007, MULT SCLER J, V13, P73, DOI 10.1177/1352458506071162

Bailey BP, 2008, ACM T COMPUT-HUM INT, V14, DOI 10.1145/1314683.1314689

Barker LM, 2011, ERGONOMICS, V54, P815, DOI 10.1080/00140139.2011.597878

BEATTY J, 1982, PSYCHOL BULL, V91, P276, DOI 10.1037/0033-2909.91.2.276

Benedetto S, 2011, TRANSPORT RES F-TRAF, V14, P199, DOI 10.1016/j.trf.2010.12.001

Bruce JM, 2010, NEUROPSYCHOLOGY, V24, P77, DOI 10.1037/a0015046

Canas JJ, 2017, ADV INTELL SYST COMP, V488, P269, DOI 10.1007/978-3-319-41691-5_23

CHALDER T, 1993, J PSYCHOSOM RES, V37, P147, DOI 10.1016/0022-3999(93)90081-P

Chiara Leva M., 2017, HUMAN MENTAL WORKLOA, P3, DOI DOI 10.1007/978-3-319-61061-0_1

Christodoulou C, 2005, ISS CLIN COGN NEUROP, P19

Dawson D, 2011, ACCIDENT ANAL PREV, V43, P549, DOI 10.1016/j.aap.2009.12.030

De Alwis Edirisinghe V., 2017, THESIS

DeLuca J, 2005, ISS CLIN COGN NEUROP, P319

Di Stasi LL, 2010, J AIR TRANSP MANAG, V16, P330, DOI 10.1016/j.jairtraman.2010.02.004

Fothergill S, 2009, BEHAV RES METHODS, V41, P118, DOI 10.3758/BRM.41.1.118

Gilbert D, 2009, STUMBLING HAPPINESS

Gopher D., 1986, HDB PERCEPTION HUMAN, V2, P41

Grandjean E., 1989, FITTING TASK MAN TXB, V4th

HAINE TWN, 2008, SPACE MED MED ENG, V21, P35, DOI DOI 10.1016/J.OCEMOD.2007.11.004

HESS EH, 1964, SCIENCE, V143, P1190, DOI 10.1126/science.143.3611.1190

Hockey G.R.J., 2011, COGNITIVE FATIGUE MU, P167, DOI [10.1037/12343-008, DOI 10.1037/12343-008]

Hockey GRJ, 1997, BIOL PSYCHOL, V45, P73

Hockey R, 2013, PSYCHOL FATIGUE WORK

Hopstaken JF, 2016, J EXP PSYCHOL HUMAN, V42, P878, DOI 10.1037/xhp0000189

Hopstaken JF, 2015, BIOL PSYCHOL, V110, P100, DOI 10.1016/j.biopsycho.2015.06.013

Horiuchi R, 2017, MICRO NANO LETT, V12, P554, DOI 10.1049/mnl.2017.0136

Ishii A, 2013, BRAIN RES, V1529, P105, DOI 10.1016/j.brainres.2013.07.022

Jainta S, 2010, INT J PSYCHOPHYSIOL, V77, P1, DOI 10.1016/j.ijpsycho.2010.03.008

Jarosz AF, 2014, J PROBL SOLVING, V7, P2, DOI 10.7771/1932-6246.1167

Johnson S K, 1997, Appl Neuropsychol, V4, P145, DOI 10.1207/s15324826an0403_1

Johnson SK, 1998, COGNITIVE NEUROPSYCH, V3, P269, DOI DOI 10.1080/135468098396099

KAHNEMAN D, 1966, SCIENCE, V154, P1583, DOI 10.1126/science.154.3756.1583

Kahneman D., 1973, ATTENTION EFFORT

Kohl A. D., 2016, THESIS

Krupp LB, 2000, NEUROLOGY, V55, P934, DOI 10.1212/WNL.55.7.934

LeDuc PA, 2005, AVIAT SPACE ENVIR MD, V76, pC86

Lemonnier S, 2014, J EYE MOVEMENT RES, V7

Lorist MM, 2005, COGNITIVE BRAIN RES, V24, P199, DOI 10.1016/j.cogbrainres.2005.01.018

Lucero-Wagoner, 2000, HDB PSYCHOPHYSIOLOGY, P142

Mathot S, 2018, BEHAV RES METHODS, V50, P94, DOI 10.3758/s13428-017-1007-2

Nakayama M., 2002, Proceedings ETRA 2002. Eye Tracking Research and Applications Symposium, P37

Nie Nie Xin Xin, 2005, THESIS

Paul RH, 1998, APPL NEUROPSYCHOL, V5, P143, DOI 10.1207/s15324826an0503_5

Roy RN, 2014, BIOMED SIGNAL PROCES, V14, P256, DOI 10.1016/j.bspc.2014.08.007

Sakamoto K., 2009, P ERG HLTH ASP WORK, P177

Sauer J, 2003, HUM FACTORS, V45, P657, DOI 10.1518/hfes.45.4.657.27090

Shen KQ, 2008, CLIN NEUROPHYSIOL, V119, P1524, DOI 10.1016/j.clinph.2008.03.012

SMETS EMA, 1995, J PSYCHOSOM RES, V39, P315, DOI 10.1016/0022-3999(94)00125-O

Stern J., 1997, HUMAN ENG QUALITY LI, V8, P1

van der Linden D, 2003, ACTA PSYCHOL, V113, P45, DOI 10.1016/S0001-6918(02)00150-6

Van Orden KF, 2001, HUM FACTORS, V43, P111, DOI 10.1518/001872001775992570

Venables L, 2009, MOTIV EMOTION, V33, P63, DOI 10.1007/s11031-008-9116-y

Vrijkotte S, 2018, INT J SPORT PHYSIOL, V13, P510, DOI 10.1123/ijspp.2016-0797

Walker LAS, 2012, J NEUROL SCI, V316, P86, DOI 10.1016/j.jns.2012.01.021

Yokoyama H, 2018, IEEE INT C INTELL TR, P1775, DOI 10.1109/ITSC.2018.8569279

[张崇 Zhang Chong], 2006, [航天医学与医学工程, Space Medicine & Medical Engineering], V19, P459

Zhang C, 2010, POL J MED PHYS ENG, V16, P67, DOI 10.2478/v10013-010-0007-7

NR 59

TC 0

Z9 0

U1 3

U2 8

PU SCIENDO

PI WARSAW

PA BOGUMILA ZUGA 32A, WARSAW, MAZOVIA, POLAND

SN 0211-2159

EI 1576-8597

J9 PSICOLOGICA

JI Psicologica

PD JUL

PY 2020

VL 41

IS 2

BP 103

EP 126

DI 10.2478/psicolj-2020-0006

PG 24

WC Psychology, Experimental

WE Social Science Citation Index (SSCI)

SC Psychology

GA MU7AK

UT WOS:000555822000002

OA gold

DA 2022-05-04

ER

PT J

AU Zeng, ZK

Huang, Z

Leng, KM

Han, WX

Niu, H

Yu, Y

Ling, Q

Liu, JH

Wu, ZG

Zang, JF

AF Zeng, Zhikang

Huang, Zhao

Leng, Kangmin

Han, Wuxiao

Niu, Hao

Yu, Yan

Ling, Qing

Liu, Jihong

Wu, Zhigang

Zang, Jianfeng

TI Nonintrusive Monitoring of Mental Fatigue Status Using Epidermal

Electronic Systems and Machine-Learning Algorithms

SO ACS SENSORS

LA English

DT Article

DE mental fatigue; nonintrusive monitoring; physiological signals;

epidermal electronics; machine learning

ID DRIVER FATIGUE; STRESS

AB Mental fatigue, characterized by subjective feelings of "tiredness" and "lack of energy", can degrade individual performance in a variety of situations, for example, in motor vehicle driving or while performing surgery. Thus, a method for nonintrusive monitoring of mental fatigue status is urgently needed. Recent research shows that physiological signal-based fatigue-classification methods using wearable electronics can be sufficiently accurate; by contrast, rigid, bulky devices constrain the behavior of those wearing them, potentially interfering with test signals. Recently, wearable electronics, such as epidermal electronics systems (EES) and electronic tattoos (E-tattoos), have been developed to meet the requirements for the comfortable measurement of various physiological signals. However, comfortable, effective, and nonintrusive monitoring of mental fatigue levels remains to be fulfilled. In this work, an EES is established to simultaneously detect multiple physiological signals in a comfortable and nonintrusive way. Machine-learning algorithms are employed to determine the mental fatigue levels and a predictive accuracy of up to 89% is achieved based on six different kinds of physiological features using decision tree algorithms. Furthermore, EES with the trained predictive model are applied to monitor in situ human mental fatigue levels when doing several routine research jobs, as well as the effect of relaxation methods in relieving fatigue.

C1 [Zeng, Zhikang; Huang, Zhao; Han, Wuxiao; Niu, Hao; Yu, Yan; Zang, Jianfeng] Huazhong Univ Sci & Technol, Sch Opt & Elect Informat, Wuhan 430074, Peoples R China.

[Zeng, Zhikang; Huang, Zhao; Han, Wuxiao; Niu, Hao; Yu, Yan; Zang, Jianfeng] Huazhong Univ Sci & Technol, Wuhan Natl Lab Optoelect, Wuhan 430074, Peoples R China.

[Zeng, Zhikang; Huang, Zhao; Zang, Jianfeng] Huazhong Univ Sci & Technol, Innovat Inst, Wuhan 430074, Peoples R China.

[Leng, Kangmin; Wu, Zhigang] Huazhong Univ Sci & Technol, State Key Lab Digital Mfg Equipment & Technol, Wuhan 430074, Peoples R China.

[Ling, Qing; Liu, Jihong] Huazhong Univ Sci & Technol, Tongji Med Coll, Tongji Hosp, Dept Urol, Wuhan 430074, Peoples R China.

RP Zang, JF (corresponding author), Huazhong Univ Sci & Technol, Sch Opt & Elect Informat, Wuhan 430074, Peoples R China.; Zang, JF (corresponding author), Huazhong Univ Sci & Technol, Wuhan Natl Lab Optoelect, Wuhan 430074, Peoples R China.; Zang, JF (corresponding author), Huazhong Univ Sci & Technol, Innovat Inst, Wuhan 430074, Peoples R China.; Wu, ZG (corresponding author), Huazhong Univ Sci & Technol, State Key Lab Digital Mfg Equipment & Technol, Wuhan 430074, Peoples R China.

EM zgwu@hust.edu.cn; jfzang@hust.edu.cn

RI Wu, Zhigang/F-6064-2012; Zang, Jianfeng/A-9363-2011

OI Wu, Zhigang/0000-0002-3719-406X; Zang, Jianfeng/0000-0002-1775-4605;

Niu, Hao/0000-0002-3323-0705

FU National Key Research and Development Program of China [2018YFB1105100];

National Natural Science Foundation of ChinaNational Natural Science

Foundation of China (NSFC) [51572096, 51820105008]

FX This work was supported by the National Key Research and Development

Program of China (2018YFB1105100) and the National Natural Science

Foundation of China (51572096 and 51820105008).

CR Ameri SK, 2017, ACS NANO, V11, P7634, DOI 10.1021/acsnano.7b02182

Awais M, 2017, SENSORS-BASEL, V17, DOI 10.3390/s17091991

Azim T, 2014, APPL SOFT COMPUT, V18, P25, DOI 10.1016/j.asoc.2014.01.020

Bills AG, 1931, AM J PSYCHOL, V43, P230, DOI 10.2307/1414771

Borghini G, 2014, NEUROSCI BIOBEHAV R, V44, P58, DOI 10.1016/j.neubiorev.2012.10.003

CHALDER T, 1993, J PSYCHOSOM RES, V37, P147, DOI 10.1016/0022-3999(93)90081-P

Chen LL, 2017, EXPERT SYST APPL, V85, P279, DOI 10.1016/j.eswa.2017.01.040

Chiang HS, 2015, J MED BIOL ENG, V35, P833, DOI 10.1007/s40846-015-0095-7

Chung HU, 2019, SCIENCE, V363, P947, DOI 10.1126/science.aau0780

Eoh HJ, 2005, INT J IND ERGONOM, V35, P307, DOI 10.1016/j.ergon.2004.09.006

FOWLES DC, 1981, PSYCHOPHYSIOLOGY, V18, P232, DOI 10.1111/j.1469-8986.1981.tb03024.x

FREWIN DB, 1976, AUSTRALAS J DERMATOL, V17, P82, DOI 10.1111/j.1440-0960.1976.tb00794.x

Huang ST, 2018, INT J MED INFORM, V119, P39, DOI 10.1016/j.ijmedinf.2018.08.010

Jeong H, 2019, ADV MATER TECHNOL-US, V4, DOI 10.1002/admt.201900117

Jeong JW, 2013, ADV MATER, V25, P6839, DOI 10.1002/adma.201301921

Kim DH, 2011, SCIENCE, V333, P838, DOI 10.1126/science.1206157

Lal SKL, 2003, J SAFETY RES, V34, P321, DOI 10.1016/S0022-4375(03)00027-6

Lal SKL, 2002, PSYCHOPHYSIOLOGY, V39, P313, DOI 10.1017/S0048577201393095

Lee BG, 2014, SENSORS-BASEL, V14, P17915, DOI 10.3390/s141017915

Miyamoto A, 2017, NAT NANOTECHNOL, V12, P907, DOI [10.1038/NNANO.2017.125, 10.1038/nnano.2017.125]

Nawrocki RA, 2018, ADV FUNCT MATER, V28, DOI 10.1002/adfm.201803279

Nishiyama K, 1997, INT J HEALTH SERV, V27, P625, DOI 10.2190/1JPC-679V-DYNT-HJ6G

Park SH, 2018, SENSOR ACTUAT B-CHEM, V273, P804, DOI 10.1016/j.snb.2018.06.125

Salahuddin L, 2007, P ANN INT IEEE EMBS, P4656, DOI 10.1109/IEMBS.2007.4353378

Scheffers MK, 1999, PSYCHOPHYSIOLOGY, V36, P149, DOI 10.1017/S0048577299980307

Seoane F, 2014, SENSORS-BASEL, V14, P7120, DOI 10.3390/s140407120

Seong H. M., 2004, 26 ANN INT C IEEE EM, P283

Singh RR, 2013, BIOMED SIGNAL PROCES, V8, P740, DOI 10.1016/j.bspc.2013.06.014

Urrila AS, 2007, BEHAV BRAIN RES, V180, P42, DOI 10.1016/j.bbr.2007.02.019

van Dooren M, 2012, PHYSIOL BEHAV, V106, P298, DOI 10.1016/j.physbeh.2012.01.020

Vicente J, 2016, MED BIOL ENG COMPUT, V54, P927, DOI 10.1007/s11517-015-1448-7

Widlund T, 2014, INT J SOLIDS STRUCT, V51, P4026, DOI 10.1016/j.ijsolstr.2014.07.025

Windmiller JR, 2012, CHEM COMMUN, V48, P6794, DOI 10.1039/c2cc32839a

Yamada Y, 2018, ARTIF INTELL MED, V91, P39, DOI 10.1016/j.artmed.2018.06.005

Yang SX, 2015, ADV MATER, V27, P6423, DOI 10.1002/adma.201502386

Yeo WH, 2013, ADV MATER, V25, P2773, DOI 10.1002/adma.201204426

Zeng H, 2018, COGN NEURODYNAMICS, V12, P597, DOI 10.1007/s11571-018-9496-y

Zeng ZK, 2017, ACS APPL MATER INTER, V9, P41078, DOI 10.1021/acsami.7b14501

Zhou C., 2018, TRANSL MAT RES, V5

NR 39

TC 10

Z9 10

U1 10

U2 45

PU AMER CHEMICAL SOC

PI WASHINGTON

PA 1155 16TH ST, NW, WASHINGTON, DC 20036 USA

SN 2379-3694

J9 ACS SENSORS

JI ACS Sens.

PD MAY 22

PY 2020

VL 5

IS 5

BP 1305

EP 1313

DI 10.1021/acssensors.9b02451

PG 9

WC Chemistry, Multidisciplinary; Chemistry, Analytical; Nanoscience &

Nanotechnology

WE Science Citation Index Expanded (SCI-EXPANDED); Social Science Citation Index (SSCI)

SC Chemistry; Science & Technology - Other Topics

GA LU1SB

UT WOS:000537540700010

PM 31939287

DA 2022-05-04

ER

PT J

AU Wang, C

Trongnetrpunya, A

Samuel, IBH

Ding, MZ

Kluger, BM

AF Wang, Chao

Trongnetrpunya, Amy

Samuel, Immanuel Babu Henry

Ding, Mingzhou

Kluger, Benzi M.

TI Compensatory Neural Activity in Response to Cognitive Fatigue

SO JOURNAL OF NEUROSCIENCE

LA English

DT Article

DE cognitive; event related potential; fatigue; Stroop

ID MENTAL FATIGUE; PERFORMANCE; ATTENTION; MODULATION; NETWORK; LOAD; FMRI

AB Prolonged continuous performance of a cognitively demanding task induces cognitive fatigue and is associated with a time-related deterioration of objective performance, the degree of which is referred to cognitive fatigability. Although the neural underpinnings of cognitive fatigue are poorly understood, prior studies report changes in neural activity consistent with deterioration of task-related networks over time. While compensatory brain activity is reported to maintain motor task performance in the face of motor fatigue and cognitive performance in the face of other stressors (e.g., aging) and structural changes, there are no studies to date demonstrating compensatory activity for cognitive fatigue. High-density electroencephalography was recorded from human subjects during a 160 min continuous performance of a cognitive control task. While most time-varying neural activity showed a linear decline over time, we identified an evoked potential over the anterior frontal region which demonstrated an inverted U-shaped time-on-task profile. This evoked brain activity peaked between 60 and 100 min into the task and was positively associated with better behavioral performance only during this interval. Following the peak and during subsequent decline of this anterior frontal activity, the rate of performance decline also accelerated. These findings demonstrate that this anterior frontal brain activity, which is not part of the primary task-related activity at baseline, is recruited to compensate for fatigue-induced impairments in the primary task-related network, and that this compensation terminates as cognitive fatigue further progresses. These findings may be relevant to understanding individual differences in cognitive fatigability and developing interventions for clinical conditions afflicted by fatigue.

C1 [Wang, Chao; Trongnetrpunya, Amy; Samuel, Immanuel Babu Henry; Ding, Mingzhou] Univ Florida, J Crayton Pruitt Family Dept Biomed Engn, Gainesville, FL 32611 USA.

[Kluger, Benzi M.] Univ Colorado Denver, Dept Neurol, Mail Stop B-185,12631 East 17th Ave, Aurora, CO 80045 USA.

[Kluger, Benzi M.] Univ Colorado Denver, Dept Psychiat, Aurora, CO 80045 USA.

RP Kluger, BM (corresponding author), Univ Colorado Denver, Dept Neurol, Mail Stop B-185,12631 East 17th Ave, Aurora, CO 80045 USA.

EM benzi.kluger@ucdenver.edu

RI Wang, Chao/M-3165-2016

OI Wang, Chao/0000-0002-1313-2569; Babu Henry Samuel,

Immanuel/0000-0001-7658-9454

FU U.S. Army Research LaboratoryUnited States Department of DefenseUS Army

Research Laboratory (ARL); U.S. Army Research Office [W911NF-10-1-0192];

Colorado Clinical and Translational Sciences Institute KL2 Program

(NIH/National Center for Advancing Translational Sciences) [8 KL2

TR000156-05]; National Institutes of HealthUnited States Department of

Health & Human ServicesNational Institutes of Health (NIH) - USA [R21

AG044862, 5 K02 NS080885-02]; National Science FoundationNational

Science Foundation (NSF) [BCS-1439188]; NATIONAL CENTER FOR ADVANCING

TRANSLATIONAL SCIENCESUnited States Department of Health & Human

ServicesNational Institutes of Health (NIH) - USANIH National Center for

Advancing Translational Sciences (NCATS) [KL2TR001080, UL1TR001082,

KL2TR000156] Funding Source: NIH RePORTER; NATIONAL INSTITUTE OF

NEUROLOGICAL DISORDERS AND STROKEUnited States Department of Health &

Human ServicesNational Institutes of Health (NIH) - USANIH National

Institute of Neurological Disorders & Stroke (NINDS) [K02NS080885]

Funding Source: NIH RePORTER; NATIONAL INSTITUTE ON AGINGUnited States

Department of Health & Human ServicesNational Institutes of Health (NIH)

- USANIH National Institute on Aging (NIA) [R21AG044862] Funding Source:

NIH RePORTER

FX This work was supported by the U.S. Army Research Laboratory and the

U.S. Army Research Office (Contract/Grant W911NF-10-1-0192), the

Colorado Clinical and Translational Sciences Institute KL2 Program

(NIH/National Center for Advancing Translational Sciences Grant 8 KL2

TR000156-05), the National Institutes of Health (NIA Grant R21 AG044862;

NINDS Grant 5 K02 NS080885-02), and the National Science Foundation

(Grant BCS-1439188).

CR Anderson KL, 2011, NEUROSCIENCE, V180, P165, DOI 10.1016/j.neuroscience.2011.02.004

Ansado J, 2012, BRAIN RES, V1454, P14, DOI 10.1016/j.brainres.2012.02.061

Barulli D, 2013, TRENDS COGN SCI, V17, P502, DOI 10.1016/j.tics.2013.08.012

Boksem MAS, 2008, BRAIN RES REV, V59, P125, DOI 10.1016/j.brainresrev.2008.07.001

Burianova H, 2013, NEUROBIOL AGING, V34, P2759, DOI 10.1016/j.neurobiolaging.2013.06.016

Chein JM, 2005, COGNITIVE BRAIN RES, V25, P607, DOI 10.1016/j.cogbrainres.2005.08.013

Cohen JD, 1999, J ABNORM PSYCHOL, V108, P120, DOI 10.1037/0021-843X.108.1.120

Delorme A, 2004, J NEUROSCI METH, V134, P9, DOI 10.1016/j.jneumeth.2003.10.009

Drummond SPA, 2000, NATURE, V403, P655, DOI 10.1038/35001068

Esposito F, 2014, PLOS ONE, V9, DOI 10.1371/journal.pone.0094222

Eyler LT, 2011, BIOL PSYCHIAT, V70, P115, DOI 10.1016/j.biopsych.2010.12.032

Filtness AJ, 2014, J SLEEP RES, V23, P568, DOI 10.1111/jsr.12163

GEVINS AS, 1987, SCIENCE, V235, P580, DOI 10.1126/science.3810158

Habeck C, 2012, BRAIN IMAGING BEHAV, V6, P568, DOI 10.1007/s11682-012-9208-x

HOLM S, 1979, SCAND J STAT, V6, P65

HUGHES JR, 1988, CLIN ELECTROENCEPHAL, V19, P210, DOI 10.1177/155005948801900407

Ishii A, 2013, BRAIN RES, V1529, P105, DOI 10.1016/j.brainres.2013.07.022

Jung TP, 2000, PSYCHOPHYSIOLOGY, V37, P163, DOI 10.1017/S0048577200980259

Kassubek J, 2001, COGNITIVE BRAIN RES, V10, P207, DOI 10.1016/S0926-6410(00)00037-9

Kluger BM, 2013, NEUROLOGY, V80, P409, DOI 10.1212/WNL.0b013e31827f07be

LEHMANN D, 1980, ELECTROEN CLIN NEURO, V48, P609, DOI 10.1016/0013-4694(80)90419-8

Liu JZ, 2002, BRAIN RES, V957, P320, DOI 10.1016/S0006-8993(02)03665-X

Lorist MM, 2005, COGNITIVE BRAIN RES, V24, P199, DOI 10.1016/j.cogbrainres.2005.01.018

Murray MM, 2008, BRAIN TOPOGR, V20, P249, DOI 10.1007/s10548-008-0054-5

PAGE EB, 1963, J AM STAT ASSOC, V58, P216, DOI 10.2307/2282965

Pardini M, 2013, SCI REP-UK, V3, DOI 10.1038/srep02001

Smith A, 2005, J PSYCHOPHARMACOL, V19, P620, DOI 10.1177/0269881105056534

Smith A, 1996, NATURE, V380, P291, DOI 10.1038/380291a0

Sohn JW, 2007, J NEUROSCI, V27, P13655, DOI 10.1523/JNEUROSCI.2982-07.2007

Stormer VS, 2013, J COGNITIVE NEUROSCI, V25, P188, DOI 10.1162/jocn_a_00303

Tajima S, 2010, NEUROL RES INT, V2010, DOI 10.1155/2010/671421

Tanaka M, 2014, BRAIN RES, V1561, P60, DOI 10.1016/j.brainres.2014.03.009

Vassena E, 2014, PLOS ONE, V9, DOI 10.1371/journal.pone.0091008

Wang C, 2015, PLOS ONE, V10, DOI 10.1371/journal.pone.0134686

Wang C, 2014, BRAIN COGNITION, V85, P251, DOI 10.1016/j.bandc.2014.01.004

Wong PCM, 2009, NEUROPSYCHOLOGIA, V47, P693, DOI 10.1016/j.neuropsychologia.2008.11.032

NR 36

TC 51

Z9 52

U1 2

U2 37

PU SOC NEUROSCIENCE

PI WASHINGTON

PA 11 DUPONT CIRCLE, NW, STE 500, WASHINGTON, DC 20036 USA

SN 0270-6474

EI 1529-2401

J9 J NEUROSCI

JI J. Neurosci.

PD APR 6

PY 2016

VL 36

IS 14

BP 3919

EP 3924

DI 10.1523/JNEUROSCI.3652-15.2016

PG 6

WC Neurosciences

WE Science Citation Index Expanded (SCI-EXPANDED)

SC Neurosciences & Neurology

GA DI4GC

UT WOS:000373457400004

PM 27053200

OA Bronze, Green Published

DA 2022-05-04

ER

PT J

AU Guastello, SJ

Correro, AN

Marra, DE

AF Guastello, Stephen J.

Correro, Anthony N., II

Marra, David E.

TI Cusp catastrophe models for cognitive workload and fatigue in teams

SO APPLIED ERGONOMICS

LA English

DT Article

DE Cognitive workload; Fatigue; Teams; Resilience; Cusp catastrophe;

Emergency response

ID WORKING-MEMORY LOAD; NONLINEAR DYNAMICS; PROCEDURAL FLEXIBILITY;

INDIVIDUAL-DIFFERENCES; CONCEPTUAL KNOWLEDGE; HUMAN-PERFORMANCE;

CAPACITY LIMITS; MENTAL WORK; SYSTEMS; STRESS

AB The use of two cusp catastrophe models has been effective for untangling the effects of cognitive workload, fatigue, and other complications on the performance of individuals. This study is the first to use the two models to separate workload and fatigue effects on team performance. In an experiment involving an emergency response simulation, 360 undergraduates were organized into 44 teams. Workload was varied by team size, number of opponents, and time pressure. The cusp models for workload and fatigue were more accurate for describing trends in team performance criteria compared to linear alternatives. Individual differences in elasticity-rigidity were less important than subjective workload and experimental conditions as control variables. Fluid intelligence within the team was an important compensatory ability in the fatigue model. Results further supported the nonlinear paradigm for the assessment of cognitive workload and fatigue and demonstrated its effectiveness for understanding team phenomena.

C1 [Guastello, Stephen J.; Correro, Anthony N., II; Marra, David E.] Marquette Univ, POB 1881, Milwaukee, WI 53201 USA.

RP Guastello, SJ (corresponding author), Marquette Univ, POB 1881, Milwaukee, WI 53201 USA.

EM Stephen.guastello@marquette.edu

RI Marra, David/AAK-4613-2021

FU Way-Klingler Fellowship grant from Marquette University

FX This research was funded by a Way-Klingler Fellowship grant from

Marquette University. The authors wish to thank Kaitlyn Bishop, Nicholas

Callard, Julian Castro, Maribeth Gomez, Michael Equi, Michael Esson,

Jacqueline Hackmon, Harli Hancock, Liana Harriri, and Claire Perna for

their assistance with data collection and data management. A report

based on this research was presented at the 27th annual conference of

the Society for Chaos Theory in Psychology & Life Sciences, Cincinnati,

Ohio in August 2017.

CR Ackerman P. L., 2011, COGNITIVE FATIGUE

Alessandri G, 2018, NONLIN DYNAM PSYCHOL, V22, P53

Ash J. E., 2014, ARCH PSYCHOL, V31, P1

Baddeley A, 2003, NAT REV NEUROSCI, V4, P829, DOI 10.1038/nrn1201

BARRON FRANK, 1955, JOUR ABNORMAL AND SOCIAL PSYCHOL, V51-31, P478, DOI 10.1037/h0048073

Barrouillet P, 2011, PSYCHOL REV, V118, P175, DOI 10.1037/a0022324

Boehm-Davis D.A., 2015, APA HDB HUMAN SYSTEM

Bonito Joseph A., 1997, COMMUNICATION YB, V20, P227, DOI DOI 10.1080/23808985.1997.11678943

Butner JE, 2014, NONLIN DYNAM PSYCHOL, V18, P397

Cantwell RH, 1996, CONTEMP EDUC PSYCHOL, V21, P500, DOI 10.1006/ceps.1996.0034

Cattell H. E. P., 1994, 16PF TECHNICAL MANUA, P1

COHEN S, 1980, PSYCHOL BULL, V88, P82, DOI 10.1037/0033-2909.88.1.82

COMER DR, 1995, HUM RELAT, V48, P647, DOI 10.1177/001872679504800603

CONRAD R, 1951, BRIT J IND MED, V8, P1

Cooke NJ, 2015, CURR DIR PSYCHOL SCI, V24, P415, DOI 10.1177/0963721415602474

Costa P. T., 1992, PERSONALITY INDIVIDU, V13, P653, DOI [DOI 10.1016/0191-8869(92)90236-I, 10.1561/0300000080, DOI 10.1561/0300000080]

Cox-Fuenzalida LE, 2006, J RES PERS, V40, P432, DOI 10.1016/j.jrp.2005.02.003

Darlington R. B., 1990, REGRESSION LINEAR MO

DENNIS AR, 1993, J APPL PSYCHOL, V78, P531, DOI 10.1037/0021-9010.78.4.531

Desmond, 2001, STRESS WORKLOAD FATI

Dodge R, 1917, PSYCHOL REV, V24, P89, DOI 10.1037/h0075549

Ein-Dor T, 2010, PERSPECT PSYCHOL SCI, V5, P123, DOI 10.1177/1745691610362349

Escartin J, 2013, NONLIN DYNAM PSYCHOL, V17, P493

Funke GJ, 2012, HUM FACTORS, V54, P36, DOI 10.1177/0018720811427901

Gerdes KE, 2011, SOC WORK RES, V35, P83, DOI 10.1093/swr/35.2.83

Gilmore R., 1981, CATASTROPHE THEORY S

Gmszka A., 2010, HDB INDIVIDUAL DIFFE

Goldstein J., 2011, SAGE HDB COMPLEXITY, P65

Gorman J. C., 2014, CURR DIR PSYCHOL SCI, V37, P255, DOI [10.1177/0963731414545215, DOI 10.1177/0963731414545215]

Gorman JC, 2017, FRONT PSYCHOL, V8, DOI 10.3389/fpsyg.2017.01053

Guastello S. J., 1995, CHAOS CATASTROPHE HU

Guastello S. J., 2014, HUMAN FACTORS ENG ER, V2nd

Guastello S. J., 2009, CHAOS COMPLEXITY PSY

Guastello S. J., 2011, NONLINEAR DYNAMICAL

Guastello S. J., 2011, NONLINEAR DYNAMICAL, P305

Guastello S. J., 2009, CHAOS COMPLEXITY PSY, P402

Guastello SJ, 2003, HUM FACTOR ERGON MAN, V13, P293, DOI 10.1002/hfm.10045

GUASTELLO SJ, 1987, J MATH PSYCHOL, V31, P248, DOI 10.1016/0022-2496(87)90029-0

GUASTELLO SJ, 1985, BEHAV SCI, V30, P204, DOI 10.1002/bs.3830300405

Guastello SJ., 2002, MANAGING EMERGENT PH

GUASTELLO SJ, 2013, MODERN RES METHODS S, P00029

GUASTELLO SJ, 2014, P HUMAN FACTORS ERGO, V0058, P00908

Guastello SJ, 2016, COGNITIVE WORKLOAD F

GUASTELLO SJ, 2017, HUMAN MENTAL WORKLOA, P00051

Guastello Stephen J, 2004, Nonlinear Dynamics Psychol Life Sci, V8, P345

Guastello Stephen J, 2005, Nonlinear Dynamics Psychol Life Sci, V9, P463

Guastello SJ, 2018, NONLIN DYNAM PSYCHOL, V22, P359

Guastello SJ, 2018, THEOR ISS ERGON SCI, V19, P229, DOI 10.1080/1463922X.2017.1356395

Guastello SJ, 2017, NONLIN DYNAM PSYCHOL, V21, P319

Guastello SJ, 2017, ERGONOMICS, V60, P167, DOI 10.1080/00140139.2016.1162851

Guastello SJ, 2016, NONLIN DYNAM PSYCHOL, V20, P509

Guastello SJ, 2016, NONLIN DYNAM PSYCHOL, V20, P49

Guastello SJ, 2015, THEOR ISS ERGON SCI, V16, P20, DOI 10.1080/1463922X.2013.869371

Guastello SJ, 2015, NONLIN DYNAM PSYCHOL, V19, P173

Guastello SJ, 2014, HUM FACTORS, V56, P737, DOI 10.1177/0018720813508777

Guastello SJ, 2014, NONLIN DYNAM PSYCHOL, V18, P177

Guastello SJ, 2013, NONLIN DYNAM PSYCHOL, V17, P405

Guastello SJ, 2013, NONLIN DYNAM PSYCHOL, V17, P23

Guastello SJ, 2012, HUM FACTORS, V54, P811, DOI 10.1177/0018720812442537

Guastello SJ, 2012, THEOR ISS ERGON SCI, V13, P586, DOI 10.1080/1463922X.2011.552131

Guastello SJ, 2012, NONLIN DYNAM PSYCHOL, V16, P471

Guastello SJ, 2010, HUM FACTORS, V52, P162, DOI 10.1177/0018720809359003

HAKSTIAN AR, 1978, J EDUC PSYCHOL, V70, P657, DOI 10.1037/0022-0663.70.5.657

HANCOCK PA, 1989, HUM FACTORS, V31, P519

Hancock PA, 1995, INT J AVIAT PSYCHOL, V5, P63, DOI 10.1207/s15327108ijap0501_5

Hancock PA, 2007, IEEE T SYST MAN CY A, V37, P586, DOI 10.1109/TSMCA.2007.897610

HART S G, 1988, P139

Hart S.G, 2006, P HUM FACT ERG SOC A, V50, P904

Hazy JK, 2015, FRONT PSYCHOL, V6, DOI 10.3389/fpsyg.2015.00806

Helton WS, 2015, COGNITION, V134, P165, DOI 10.1016/j.cognition.2014.10.001

Helton WS, 2014, HUM FACTORS, V56, P322, DOI 10.1177/0018720813490727

Helton WS, 2013, EXP BRAIN RES, V224, P429, DOI 10.1007/s00221-012-3322-2

Helton WS, 2011, EXP BRAIN RES, V212, P429, DOI 10.1007/s00221-011-2749-1

Hockey G. R. J., 2012, HDB OPERATOR FATIGUE, P45

Hockey G.R.J., 2011, COGNITIVE FATIGUE MU, P167, DOI [10.1037/12343-008, DOI 10.1037/12343-008]

Hockey GRJ, 1997, BIOL PSYCHOL, V45, P73

Hollnagel E., 2012, FRAM FUNCTION RESONA

Hollnagel E., 2006, RESILIENCE ENG

Hong SL, 2010, NONLIN DYNAM PSYCHOL, V14, P291

Jacobsen JJ, 2011, NONLIN DYNAM PSYCHOL, V15, P307

Jacobsen Joseph J, 2007, Nonlinear Dynamics Psychol Life Sci, V11, P499

Jansen RJ, 2016, HUM FACTORS, V58, P1143, DOI 10.1177/0018720816669271

Kane MJ, 2005, PSYCHOL BULL, V131, P66, DOI 10.1037/0033-2909.131.1.66

Kane MJ, 2002, PSYCHON B REV, V9, P637, DOI 10.3758/BF03196323

Kantowitz B., 1983, HUMAN FACTORS UNDERS, DOI [10.1037/a0027770, DOI 10.1037/A0027770]

KANTOWITZ BH, 1985, J MATH PSYCHOL, V29, P135, DOI 10.1016/0022-2496(85)90014-8

Karwowski W, 2012, HUM FACTORS, V54, P983, DOI 10.1177/0018720812467459

Katerelos I, 2017, NONLIN DYNAM PSYCHOL, V21, P89

Katerndahl D, 2011, NONLIN DYNAM PSYCHOL, V15, P253

Katidioti I, 2014, HUM FACTORS, V56, P728, DOI 10.1177/0018720813504216

Kato T, 2012, J COUNS PSYCHOL, V59, P262, DOI 10.1037/a0027770

Kiefer AW, 2015, NONLIN DYNAM PSYCHOL, V19, P489

KROLL W, 1981, RES Q EXERCISE SPORT, V52, P523, DOI 10.1080/02701367.1981.10607899

LATANE B, 1979, J PERS SOC PSYCHOL, V37, P822, DOI 10.1037//0022-3514.37.6.822

Leva, 2017, HUMAN MENTAL WORKLOA, VVol 726, P90, DOI DOI 10.1007/978-3-319-61061-0_6

Lietz C.A., 2011, J SOC SOC WORK RES, V2, P104, DOI [10.5243/jsswr.2011.6, DOI 10.5243/JSSWR.2011.6]

Liu Y, 2014, PSYCHOL SCI, V25, P1116, DOI 10.1177/0956797614525213

Logie RH, 2011, CURR DIR PSYCHOL SCI, V20, P240, DOI 10.1177/0963721411415340

MacLean M. H., 2012, PSYCHONOMIC B REV, V17, P556, DOI DOI 10.3758/PBR.17.4.556

Matthews G., 2012, HDB OPERATOR FATIGUE

Morgan JF, 2011, HUM FACTORS, V53, P75, DOI 10.1177/0018720810393505

MOSSO A, 1894, FATIGUE INTELLECTUEL

Naber AM, 2015, HUM FACTORS, V57, P163, DOI 10.1177/0018720814538814

NEWHOUSE S, 1978, COMMUN MATH PHYS, V64, P35, DOI 10.1007/BF01940759

Oberauer K, 2006, J MEM LANG, V55, P601, DOI 10.1016/j.jml.2006.08.009

Oliva T. A., 2008, PSYCHOL LIFE SCI, V12, P261

PASCUALLJ, 1970, ACTA PSYCHOL, V32, P301, DOI DOI 10.1016/0001-6918(70)90108-3

Pincus D, 2010, NONLIN DYNAM PSYCHOL, V14, P353

RALPH J, 2010, P HUMAN FACTORS ERGO, V0054, P00299

Rebelo T, 2016, NONLIN DYNAM PSYCHOL, V20, P537

Reuter-Lorenz PA, 2008, CURR DIR PSYCHOL SCI, V17, P177, DOI 10.1111/j.1467-8721.2008.00570.x

Rittle-Johnson B, 2009, J EDUC PSYCHOL, V101, P836, DOI 10.1037/a0016026

Rittle-Johnson B, 2009, J EDUC PSYCHOL, V101, P529, DOI 10.1037/a0014224

Rose CL, 2002, EUR J PERSONALITY, V16, P185, DOI 10.1002/per.451

Rubinstein JS, 2001, J EXP PSYCHOL HUMAN, V27, P763, DOI 10.1037//0096-1523.27.4.763

Sawyer R.K., 2005, SOCIAL EMERGENCE SOC, DOI [10.1017/CBO9780511734892, DOI 10.1017/CBO9780511734892]

Schneider M, 2011, DEV PSYCHOL, V47, P1525, DOI 10.1037/a0024997

Schuldberg D, 2015, NONLIN DYNAM PSYCHOL, V19, P553

Schutte NS, 1998, PERS INDIV DIFFER, V25, P167, DOI 10.1016/S0191-8869(98)00001-4

Sellers J., 2014, P HUM FACT ERG SOC A, V58, P989, DOI DOI 10.1177/154193121

Sheridan TB, 2008, HUM FACTORS, V50, P418, DOI 10.1518/001872008X250773

Shockley K, 2009, TOP COGN SCI, V1, P305, DOI 10.1111/j.1756-8765.2009.01021.x

Sprott J. C., 2003, CHAOS TIME SERIES AN

Stamovlasis D, 2017, NONLIN DYNAM PSYCHOL, V21, P267

Stamovlasis D, 2014, NONLIN DYNAM PSYCHOL, V18, P67

Stamovlasis D, 2012, SCI EDUC, V96, P392, DOI 10.1002/sce.21002

Stamovlasis D, 2011, NONLIN DYNAM PSYCHOL, V15, P145

Stamovlasis Dimitrios, 2006, Nonlinear Dynamics Psychol Life Sci, V10, P37

Starch D, 1917, PSYCHOL REV, V24, P391, DOI 10.1037/h0074010

Sulis W., 2009, CHAOS COMPLEXITY PSY, P41, DOI 10.1017/CBO9781139058544.003

Szalma J. L., 2012, HDB OPERATOR FATIGUE, P75

Szalma JL, 2012, ACTA PSYCHOL, V139, P471, DOI 10.1016/j.actpsy.2011.12.009

Taleb N.N., 2012, ANTIFRAGILE THINGS G

TAYLOR JA, 1953, J ABNORM SOC PSYCH, V48, P285, DOI 10.1037/h0056264

Thom R., 1975, STRUCTURAL STABILITY

Thompson HL., 2010, STRESS EFFECT WHY SM

Thomson DR, 2015, PERSPECT PSYCHOL SCI, V10, P82, DOI 10.1177/1745691614556681

Vytal K, 2012, PSYCHOPHYSIOLOGY, V49, P842, DOI 10.1111/j.1469-8986.2012.01358.x

Warm JS, 2008, HUM FACTORS, V50, P433, DOI 10.1518/001872008X312152

Wheelan SA, 2009, SMALL GR RES, V40, P247, DOI 10.1177/1046496408328703

Wickens, 2002, THEORETICAL ISSUES E, V3, P159, DOI [DOI 10.1080/14639220210123806, 10.1080/14639220210123806]

Wickens CD, 2008, HUM FACTORS, V50, P397, DOI 10.1518/001872008X288420

Witkin H. A., 2002, MANUAL EMBEDDED FIGU

Zander A., 1994, MAKING GROUPS EFFECT

Zeeman E. C., 1977, CATASTROPHE THEORY S

[No title captured]

[No title captured]

NR 147

TC 10

Z9 10

U1 7

U2 25

PU ELSEVIER SCI LTD

PI OXFORD

PA THE BOULEVARD, LANGFORD LANE, KIDLINGTON, OXFORD OX5 1GB, OXON, ENGLAND

SN 0003-6870

EI 1872-9126

J9 APPL ERGON

JI Appl. Ergon.

PD SEP

PY 2019

VL 79

BP 152

EP 168

DI 10.1016/j.apergo.2018.08.019

PG 17

WC Engineering, Industrial; Ergonomics; Psychology, Applied

WE Science Citation Index Expanded (SCI-EXPANDED); Social Science Citation Index (SSCI)

SC Engineering; Psychology

GA IC4RF

UT WOS:000470952300016

PM 30195844

OA Green Published

DA 2022-05-04

ER

PT J

AU Borragan, G

Guerrero-Mosquera, C

Guillaume, C

Slama, H

Peigneux, P

AF Borragan, Guillermo

Guerrero-Mosquera, Carlos

Guillaume, Celine

Slama, Hichem

Peigneux, Philippe

TI Decreased prefrontal connectivity parallels cognitive fatigue-related

performance decline after sleep deprivation. An optical imaging study

SO BIOLOGICAL PSYCHOLOGY

LA English

DT Article

DE Cognitive fatigue; Sleep deprivation; Connectivity; fNIRS;

Haemodynamics; Human performance

ID NEAR-INFRARED SPECTROSCOPY; WORKING-MEMORY; MENTAL FATIGUE; CEREBRAL

HEMODYNAMICS; TASK; VIGILANCE; CORTEX; METAANALYSIS; INSTRUMENT;

NETWORKS

AB Fatigue induced by sustained cognitive demands often entails decreased behavioural performance and the unavailability of brain resources, either due to reduced levels or impaired access. In the present study, we investigated the neural dynamics underlying preserved behavioural performance after inducing cognitive fatigue (CF) in a sleep deprivation (SD) condition in which resources are naturally compromised. Using functional near infrared spectroscopy (fNIRS), we recorded cortical brain activity during task-related CF induction in the evening, in the middle of the night and early in the morning. Although cortical oxygenation similarly increased over the 3 sessions, decreased intra-hemispheric connectivity between left anterior frontal and frontal areas paralleled a sudden drop in task performance in the early morning. Our data indicate that decreased sustained attention after the induction of cognitive fatigue in a situation of high sleep pressure results from impaired connectivity between left prefrontal cortical areas rather than from a mere modulation in brain resources.

C1 [Borragan, Guillermo; Guillaume, Celine; Slama, Hichem; Peigneux, Philippe] Univ Libre Bruxelles, CRCN, UR2NF Neuropsychol & Funct Neuroimaging, Brussels, Belgium.

[Borragan, Guillermo; Guillaume, Celine; Slama, Hichem; Peigneux, Philippe] Univ Libre Bruxelles, UNI ULB Neurosci Inst, Brussels, Belgium.

[Slama, Hichem] Univ Libre Bruxelles, CRCN, UNESCOG Cognit Neurosci Res Unit, Brussels, Belgium.

[Guerrero-Mosquera, Carlos] Univ Pompeu Fabra, Ctr Brain & Cognit, Barcelona, Spain.

[Slama, Hichem] Erasme Univ Hosp, Dept Clin & Cognit Neuropsychol, Anderlecht, Belgium.

RP Borragan, G; Peigneux, P (corresponding author), Univ Libre Bruxelles, CRCN, UR2NF Neuropsychol & Funct Neuroimaging, Brussels, Belgium.; Borragan, G; Peigneux, P (corresponding author), Univ Libre Bruxelles, UNI ULB Neurosci Inst, Brussels, Belgium.

EM gborraganpedraz@gmail.com; Philippe.Peigneux@ulb.ac.be

RI Guerrero-Mosquera, Carlos/AAN-6241-2020

OI Guerrero-Mosquera, Carlos/0000-0001-8265-3651; Borragan,

Guillermo/0000-0003-1370-4384; Slama, Hichem/0000-0003-3856-4488

FU Programme IPA/PAI COOL [P7/33]; FRS-FNRSFonds de la Recherche

Scientifique - FNRS [T.0109.13]; FRS-FNRS "Grands Equipements"

grantFonds de la Recherche Scientifique - FNRS [2.5020.12]

FX GB was supported by the Programme P7/33 IPA/PAI COOL. CGM was supported

by FRS-FNRS grant T.0109.13. PP was Francqui Research Professor

2013-2016. The NIRS equipment was supported by FRS-FNRS "Grands

Equipements" grant 2.5020.12.

CR Aasted CM, 2015, NEUROPHOTONICS, V2, DOI 10.1117/1.NPh.2.2.020801

AKERSTEDT T, 1990, INT J NEUROSCI, V52, P29, DOI 10.3109/00207459008994241

Akerstedt T, 2004, J PSYCHOSOM RES, V57, P427, DOI 10.1016/j.jpsychores.2003.12.001

Ayaz H, 2012, NEUROIMAGE, V59, P36, DOI 10.1016/j.neuroimage.2011.06.023

Basner M, 2011, SLEEP, V34, P581, DOI 10.1093/sleep/34.5.581

Blumenfeld RS, 2006, J NEUROSCI, V26, P916, DOI 10.1523/JNEUROSCI.2353-05.2006

Borghini G, 2014, NEUROSCI BIOBEHAV R, V44, P58, DOI 10.1016/j.neubiorev.2012.10.003

Borragan G, 2017, CORTEX, V89, P71, DOI 10.1016/j.cortex.2017.01.023

Borragan G, 2016, FRONT HUM NEUROSCI, V10, DOI 10.3389/fnhum.2016.00086

Buysse D J, 1989, Psychiatry Res, V28, P193

Chee M. W. L, 2004, J NEUROSCIENCE OFFIC

Cook DB, 2007, NEUROIMAGE, V36, P108, DOI 10.1016/j.neuroimage.2007.02.033

Curtis CE, 2003, TRENDS COGN SCI, V7, P415, DOI 10.1016/S1364-6613(03)00197-9

DELPY DT, 1988, PHYS MED BIOL, V33, P1433, DOI 10.1088/0031-9155/33/12/008

Desmond PA, 1997, ACCIDENT ANAL PREV, V29, P515, DOI 10.1016/S0001-4575(97)00031-6

DINGES DF, 1985, BEHAV RES METH INSTR, V17, P652, DOI 10.3758/BF03200977

Dosenbach NUF, 2007, P NATL ACAD SCI USA, V104, P11073, DOI 10.1073/pnas.0704320104

Drummond S. P, 2001, J SLEEP RES

Drummond SPA, 2004, SLEEP, V27, P445

Drummond SPA, 2001, NEUROPSYCHOPHARMACOL, V25, pS68, DOI 10.1016/S0893-133X(01)00325-6

ELLIS BW, 1981, SLEEP, V4, P93, DOI 10.1093/sleep/4.1.93

Fishburn FA, 2014, FRONT HUM NEUROSCI, V8, DOI 10.3389/fnhum.2014.00076

Fritz CO, 2012, J EXP PSYCHOL GEN, V141, P2, DOI 10.1037/a0024338

Guerrero-Mosquera C, 2016, J NEUROSCI METH, V271, P128, DOI 10.1016/j.jneumeth.2016.07.010

Gui DY, 2015, NEUROIMAGE, V120, P323, DOI 10.1016/j.neuroimage.2015.07.030

HANCOCK PA, 1989, HUM FACTORS, V31, P519

Harrivel AR, 2013, FRONT HUM NEUROSCI, V7, DOI 10.3389/fnhum.2013.00861

Helton WS, 2010, NEUROPSYCHOLOGIA, V48, P1683, DOI 10.1016/j.neuropsychologia.2010.02.014

Herff C, 2014, FRONT HUM NEUROSCI, V7, DOI 10.3389/fnhum.2013.00935

Hockey GRJ, 1997, BIOL PSYCHOL, V45, P73

Hockey R., 2013, PSYCHOL FATIGUE

Hopstaken JF, 2015, PSYCHOPHYSIOLOGY, V52, P305, DOI 10.1111/psyp.12339

HORNE J A, 1976, International Journal of Chronobiology, V4, P97

Huppert TJ, 2009, APPL OPTICS, V48, pD280, DOI 10.1364/AO.48.00D280

Kahnt T, 2011, NEUROIMAGE, V56, P709, DOI 10.1016/j.neuroimage.2010.05.058

Kajimoto O., 2008, FATIGUE SCI HUMAN HL, P33

KRUEGER GP, 1989, WORK STRESS, V3, P129, DOI 10.1080/02678378908256939

Kurzban R, 2013, BEHAV BRAIN SCI, V36, P661, DOI 10.1017/S0140525X12003196

Lal SKL, 2001, BIOL PSYCHOL, V55, P173, DOI 10.1016/S0301-0511(00)00085-5

LEE KA, 1991, PSYCHIAT RES, V36, P291, DOI 10.1016/0165-1781(91)90027-M

Li ZY, 2009, EUR J APPL PHYSIOL, V107, P281, DOI 10.1007/s00421-009-1122-6

Lim J, 2010, PSYCHOL BULL, V136, P375, DOI 10.1037/a0018883

Lorist MM, 2005, COGNITIVE BRAIN RES, V24, P199, DOI 10.1016/j.cogbrainres.2005.01.018

Ma N, 2015, SLEEP, V38, P233, DOI 10.5665/sleep.4404

Monk Timothy H, 2005, Clin Sports Med, V24, pe15, DOI 10.1016/j.csm.2004.12.002

Neu D, 2011, PSYCHIAT RES, V189, P128, DOI 10.1016/j.psychres.2010.12.005

Neu D, 2010, NEUROEPIDEMIOLOGY, V35, P1, DOI 10.1159/000301714

Owen AM, 2005, HUM BRAIN MAPP, V25, P46, DOI 10.1002/hbm.20131

Passingham D, 2004, CURR OPIN NEUROBIOL, V14, P163, DOI 10.1016/j.conb.2004.03.003

Penner IK, 2009, MULT SCLER J, V15, P1509, DOI 10.1177/1352458509348519

Petrides M., 1994, HDB NEUROPSYCHOLOGY, V9, P17

Pinti P, 2020, ANN NY ACAD SCI, V1464, P5, DOI 10.1111/nyas.13948

Piper SK, 2014, NEUROIMAGE, V85, P64, DOI 10.1016/j.neuroimage.2013.06.062

Pittion-Vouyovitch S, 2006, J NEUROL SCI, V243, P39, DOI 10.1016/j.jns.2005.11.025

Rankin CH, 2009, NEUROBIOL LEARN MEM, V92, P135, DOI 10.1016/j.nlm.2008.09.012

Resnick HE, 2006, J CLIN SLEEP MED, V2, P163

Rousselet GA, 2012, FRONT HUM NEUROSCI, V6, DOI 10.3389/fnhum.2012.00119

Saxby D. J., 2007, P DRIV SIM C N AM

Schulz P, 1982, ACHIEVEMENT STRESS A, P51

Setyawati Lientje, 1995, Journal of Human Ergology, V24, P129

Shen JH, 2006, SLEEP MED REV, V10, P63, DOI 10.1016/j.smrv.2005.05.004

Sievertsen HH, 2016, P NATL ACAD SCI USA, V113, P2621, DOI 10.1073/pnas.1516947113

Tachtsidis I, 2004, PHYSIOL MEAS, V25, P437, DOI 10.1088/0967-3334/25/2/003

Thomas Maria L., 2003, Thalamus & Related Systems, V2, P199, DOI 10.1016/S1472-9288(03)00020-7

Tomasi D, 2009, CEREB CORTEX, V19, P233, DOI 10.1093/cercor/bhn073

Tsujimoto S, 2005, J NEUROPHYSIOL, V93, P3687, DOI 10.1152/jn.01149.2004

Wang C, 2016, J NEUROSCI, V36, P3919, DOI 10.1523/JNEUROSCI.3652-15.2016

Warm JS, 2009, MIL PSYCHOL, V21, P75, DOI 10.1080/08995600802554706

Yoshino K, 2013, FRONT HUM NEUROSCI, V7, DOI 10.3389/fnhum.2013.00895

Young MS, 2002, HUM FACTORS, V44, P365, DOI 10.1518/0018720024497709

Zang YF, 2007, BRAIN DEV-JPN, V29, P83, DOI 10.1016/j.braindev.2006.07.002

Zhang H, 2010, NEUROIMAGE, V51, P1150, DOI 10.1016/j.neuroimage.2010.02.080

ZIGMOND AS, 1983, ACTA PSYCHIAT SCAND, V67, P361, DOI 10.1111/j.1600-0447.1983.tb09716.x

2008, NEUROIMAGE, V39, P527, DOI DOI 10.1016/J.NEUROIMAGE.2007.08.008

NR 74

TC 17

Z9 18

U1 9

U2 33

PU ELSEVIER

PI AMSTERDAM

PA RADARWEG 29, 1043 NX AMSTERDAM, NETHERLANDS

SN 0301-0511

EI 1873-6246

J9 BIOL PSYCHOL

JI Biol. Psychol.

PD MAY

PY 2019

VL 144

BP 115

EP 124

DI 10.1016/j.biopsycho.2019.03.004

PG 10

WC Psychology, Biological; Behavioral Sciences; Psychology; Psychology,

Experimental

WE Science Citation Index Expanded (SCI-EXPANDED); Social Science Citation Index (SSCI)

SC Psychology; Behavioral Sciences

GA HU6KX

UT WOS:000465390100013

PM 30930071

DA 2022-05-04

ER

PT J

AU Pattyn, N

Van Cutsem, J

Dessy, E

Mairesse, O

AF Pattyn, Nathalie

Van Cutsem, Jeroen

Dessy, Emilie

Mairesse, Olivier

TI Bridging Exercise Science, Cognitive Psychology, and Medical Practice:

Is "Cognitive Fatigue" a Remake of "The Emperor's New Clothes"?

SO FRONTIERS IN PSYCHOLOGY

LA English

DT Article

DE cognitive fatigue; exercise tolerance; fatigue; sleep disorders; chronic

fatigue; effortful control; performance

ID MULTIPLE-SCLEROSIS; MENTAL FATIGUE; PHYSICAL PERFORMANCE; SUSTAINED

ATTENTION; VIGILANCE DECREMENT; DAYTIME SLEEPINESS; CINGULATE CORTEX;

SELF; INTENSITY; GLUCOSE

AB Fatigue is such a multifaceted construct it has sprouted specific research fields and experts in domains as different as exercise physiology, cognitive psychology, human factors and engineering, and medical practice. It lacks a consensus definition: it is an experimental concept, a symptom, a risk, a cause (e.g., of performance decrement) and a consequence (e.g., of sleep deprivation). This fragmentation of knowledge leads to slower dissemination of novel insights, and thus to a poorer research. Indeed, what may seem as a novel result in one field, may very well be old news in another, hence leading to this "innovation" being a scientific equivalent to the emperor's new clothes. The current paper aims to describe the common denominator in the different areas of expertise where fatigue is investigated. Indeed, rather than focusing on the differences in semantics and conceptualization, we hope that identifying common concepts may be inductive of easier multidisciplinary research. Considering the vastness of fatigue research in all areas identified as relevant-cognitive science, exercise physiology, and medical practice, this analysis has not the ambition to be an exhaustive review in all domains. We have reviewed the fatigue concepts and research in these areas and report the ones that are used to describe the proposed common model to be further investigated. The most promising common feature to cognitive science, exercise physiology and clinical practice is the notion of "perceived effort." This allows to account for interindividual differences, as well as for the situational variations in fatigue. It is applicable to both mental and physical constructs. It integrates motivational and emotional dimensions. It overcomes current polemics in various research fields, and it does not draw on any semantic ambiguity. We thus suggest a new model of fatigue and performance, whether this performance is mental or physical; and whether it is in a clinical range or relates to optimal functioning.

C1 [Pattyn, Nathalie; Dessy, Emilie; Mairesse, Olivier] Royal Mil Acad, VIPER Res Unit, Brussels, Belgium.

[Pattyn, Nathalie; Dessy, Emilie; Mairesse, Olivier] Vrije Univ Brussel, Dept Expt & Appl Psychol, Brussels, Belgium.

[Pattyn, Nathalie; Van Cutsem, Jeroen] Vrije Univ Brussel, Human Physiol Res Grp, Brussels, Belgium.

[Van Cutsem, Jeroen] Univ Kent, Endurance Res Grp, Chatham, Kent, England.

[Mairesse, Olivier] CHU Brugmann, Sleep Unit, Brussels, Belgium.

RP Pattyn, N (corresponding author), Royal Mil Acad, VIPER Res Unit, Brussels, Belgium.; Pattyn, N (corresponding author), Vrije Univ Brussel, Dept Expt & Appl Psychol, Brussels, Belgium.; Pattyn, N (corresponding author), Vrije Univ Brussel, Human Physiol Res Grp, Brussels, Belgium.

EM npattyn@vub.ac.be

RI Van Cutsem, Jeroen/AAZ-6281-2021; Mairesse, Olivier/AAM-6427-2020; Van

Cutsem, Jeroen/AAZ-7325-2021

OI Van Cutsem, Jeroen/0000-0001-6122-7629; Pattyn,

Nathalie/0000-0002-2690-5479

FU Belgian DoD; VUB; DoDUnited States Department of Defense [HFM1705];

Brugman University Hospital

FX NP was funded by the Belgian DoD and the VUB. ED was funded by a DoD

grant HFM1705. OM was funded by Brugman University Hospital and the VUB.

CR Abbiss CR, 2005, SPORTS MED, V35, P865, DOI 10.2165/00007256-200535100-00004

Ackerman PL, 2009, J EXP PSYCHOL-APPL, V15, P163, DOI 10.1037/a0015719

Allen DG, 2008, J APPL PHYSIOL, V104, P296, DOI 10.1152/japplphysiol.00908.2007

Ament W, 2009, SPORTS MED, V39, P389, DOI 10.2165/00007256-200939050-00005

[Anonymous], 2015, NAME

Barrouillet P, 2004, J EXP PSYCHOL GEN, V133, P83, DOI 10.1037/0096-3445.133.1.83

Baumeister RF, 1998, J PERS SOC PSYCHOL, V74, P1252, DOI 10.1037/0022-3514.74.5.1252

Bazelmans E, 2005, J PSYCHOSOM RES, V59, P201, DOI 10.1016/j.jpsychores.2005.04.003

Bazelmans E, 2001, PSYCHOL MED, V31, P107, DOI 10.1017/S0033291799003189

BERNTSON GG, 1993, PSYCHOL BULL, V114, P296, DOI 10.1037/0033-2909.114.2.296

Bianchi R, 2015, FRONT PUBLIC HEALTH, V3, DOI 10.3389/fpubh.2015.00158

Bogdanis GC, 2012, FRONT PHYSIOL, V3, DOI 10.3389/fphys.2012.00142

Boksem MAS, 2008, BRAIN RES REV, V59, P125, DOI 10.1016/j.brainresrev.2008.07.001

Borragan G, 2017, CORTEX, V89, P71, DOI 10.1016/j.cortex.2017.01.023

Borragan G, 2016, FRONT HUM NEUROSCI, V10, DOI 10.3389/fnhum.2016.00086

Bray SR, 2008, PSYCHOPHYSIOLOGY, V45, P337, DOI 10.1111/j.1469-8986.2007.00625.x

Bray SR, 2012, BIOL PSYCHOL, V89, P195, DOI 10.1016/j.biopsycho.2011.10.008

BREHM JW, 1989, ANNU REV PSYCHOL, V40, P109, DOI 10.1146/annurev.ps.40.020189.000545

Brownsberger J, 2013, INT J SPORTS MED, V34, P1029, DOI 10.1055/s-0033-1343402

CALDWELL J, 1995, BIOL PSYCHOL, V40, P197, DOI 10.1016/0301-0511(95)05115-5

Clauw DJ, 2014, JAMA-J AM MED ASSOC, V311, P1547, DOI 10.1001/jama.2014.3266

Craig AD, 2002, NAT REV NEUROSCI, V3, P655, DOI 10.1038/nrn894

Critchley HD, 2003, BRAIN, V126, P2139, DOI 10.1093/brain/awg216

Dahm T, 2011, PLOS ONE, V6, DOI 10.1371/journal.pone.0026351

DAVID A, 1990, BMJ-BRIT MED J, V301, P1199, DOI 10.1136/bmj.301.6762.1199

de Morree HM, 2014, J APPL PHYSIOL, V117, P1514, DOI 10.1152/japplphysiol.00898.2013

de Morree HM, 2012, PSYCHOPHYSIOLOGY, V49, P1242, DOI 10.1111/j.1469-8986.2012.01399.x

Desmond, 2001, STRESS WORKLOAD FATI

Di Giulio C, 2006, ADV PHYSIOL EDUC, V30, P51, DOI 10.1152/advan.00041.2005

DIGIULIO C, 2011, ARCH ITAL BIOL, P149

Dinges DF, 1997, SLEEP, V20, P267

Dittner AJ, 2004, J PSYCHOSOM RES, V56, P157, DOI 10.1016/S0022-3999(03)00371-4

Dockree PM, 2007, EUR J NEUROSCI, V25, P900, DOI 10.1111/j.1460-9568.2007.05324.x

Evans D. R., 2015, PERS SOC PSYCHOL REV, DOI 10.1177/1088868315597841

Eysenck M., 1982, BERLIN, DOI 10.1007/978-3-642-68390-9

Fairclough SH, 2004, BIOL PSYCHOL, V66, P177, DOI 10.1016/j.biopsycho.2003.10.001

FOLKMAN S, 1985, J PERS SOC PSYCHOL, V48, P150, DOI 10.1037/0022-3514.48.1.150

Frank GKW, 2008, NEUROIMAGE, V39, P1559, DOI 10.1016/j.neuroimage.2007.10.061

FREAL JE, 1984, ARCH PHYS MED REHAB, V65, P135

Freeman FG, 2004, BIOL PSYCHOL, V67, P283, DOI 10.1016/j.biopsycho.2004.01.002

Gailliot MT, 2007, J PERS SOC PSYCHOL, V92, P325, DOI 10.1037/0022-3514.92.2.325

Gergelyfi M, 2015, FRONT BEHAV NEUROSCI, V9, DOI [10.3389/fnbeh.2015.00176, 10.3389/fnbeh.2015,00176]

Gibson AS, 2018, EUR J SPORT SCI, V18, P25, DOI 10.1080/17461391.2017.1321688

Gibson AS, 2004, BRIT J SPORT MED, V38, P797, DOI 10.1136/bjsm.2003.009852

Glaus A, 1996, SUPPORT CARE CANCER, V4, P82, DOI 10.1007/BF01845757

Grier RA, 2003, HUM FACTORS, V45, P349, DOI 10.1518/hfes.45.3.349.27253

Guilleminault C, 2001, BRAIN, V124, P1482, DOI 10.1093/brain/124.8.1482

Hagger MS, 2013, PERS SOC PSYCHOL B, V39, P28, DOI 10.1177/0146167212459912

Hagglund L, 2008, EUR J CARDIOVASC NUR, V7, P290, DOI 10.1016/j.ejcnurse.2007.12.004

Helton WS, 2005, BRIT J PSYCHOL, V96, P249, DOI 10.1348/000712605X38369

Hockey G.R.J., 1983, STRESS FATIGUE HUMAN

Hockey R., 2013, THE PSYCHOLOGY OF FA, DOI 10.1017/CBO9781139015394

Holroyd CB, 2002, PSYCHOL REV, V109, P679, DOI [10.1037//0033-295X.109.4.679, 10.1037/0033-295X.109.4.679]

Hossain JL, 2003, J SLEEP RES, V12, P223, DOI 10.1046/j.1365-2869.2003.00354.x

Inzlicht M, 2007, PSYCHOL SCI, V18, P933, DOI 10.1111/j.1467-9280.2007.02004.x

Inzlicht M, 2016, FRONT PSYCHOL, V7, DOI 10.3389/fpsyg.2016.00656

Kahneman D., 1982, PRESS, DOI 10.1017/CBO9780511809477

Kayser B, 2003, EUR J APPL PHYSIOL, V90, P411, DOI 10.1007/s00421-003-0902-7

Klass M, 2016, MED SCI SPORT EXER, V48, P1014, DOI 10.1249/MSS.0000000000000879

KRUPP LB, 1989, ARCH NEUROL-CHICAGO, V46, P1121, DOI 10.1001/archneur.1989.00520460115022

Krupp LB, 2003, CNS DRUGS, V17, P225, DOI 10.2165/00023210-200317040-00002

Kurzban R, 2010, EVOL PSYCHOL-US, V8, P244, DOI 10.1177/147470491000800208

Lim JL, 2008, ANN NY ACAD SCI, V1129, P305, DOI 10.1196/annals.1417.002

Mackworth NH, 1948, Q J EXP PSYCHOL, V1, P6, DOI 10.1080/17470214808416738

MacMahon C, 2014, J SPORT EXERCISE PSY, V36, P375, DOI 10.1123/jsep.2013-0249

Mairesse O., 2017, BEHAV SLEEP MED, P1, DOI 10.1080/15402002.2017.1395336

Mairesse O., 2014, J SLEEP RES, V23, P94, DOI 10.1016/j.clinph.2014.12.016

Mairesse O, 2016, PSYCHIAT RES, V238, P100, DOI 10.1016/j.psychres.2016.02.005

Manly T, 1999, NEUROPSYCHOLOGIA, V37, P661, DOI 10.1016/S0028-3932(98)00127-4

Marcora S, 2016, SPORTS MED, V46, P1, DOI 10.1007/s40279-015-0412-x

Marcora SM, 2008, EUR J APPL PHYSIOL, V104, P929, DOI 10.1007/s00421-008-0818-3

Marcora SM, 2009, J APPL PHYSIOL, V106, P857, DOI 10.1152/japplphysiol.91324.2008

Marcora SM, 2010, EUR J APPL PHYSIOL, V109, P763, DOI 10.1007/s00421-010-1418-6

Mariman AN, 2013, SLEEP MED REV, V17, P193, DOI 10.1016/j.smrv.2012.06.003

Matthews G., 2011, COGNITIVE FATIGUE MU, P209, DOI 10.1037/12343-010

Matthews SC, 2004, NEUROIMAGE, V22, P1151, DOI 10.1016/j.neuroimage.2004.03.005

McKenna MJ, 2008, J APPL PHYSIOL, V104, P286, DOI 10.1152/japplphysiol.01139.2007

Meeusen R, 2018, EUR J SPORT SCI, V18, P37, DOI 10.1080/17461391.2017.1296890

Molden DC, 2012, PSYCHOL SCI, V23, P1137, DOI 10.1177/0956797612439069

Mosso A., 1915, LONDON

Neu D, 2007, NEUROPSYCHOBIOLOGY, V56, P40, DOI 10.1159/000110727

Neu D, 2010, NEUROEPIDEMIOLOGY, V35, P1, DOI 10.1159/000301714

Neu D, 2010, ACTA NEUROL BELG, V110, P15

Neu D, 2008, J SLEEP RES, V17, P427, DOI 10.1111/j.1365-2869.2008.00679.x

NEYROUD D, 2016, FRONT PHYSIOL, V7

Nijs J, 2018, PHYS THER, V98, P325, DOI 10.1093/ptj/pzy020

Nybo L, 2004, PROG NEUROBIOL, V72, P223, DOI 10.1016/j.pneurobio.2004.03.005

Orff HJ, 2007, SLEEP, V30, P1205, DOI 10.1093/sleep/30.9.1205

Pageaux B, 2015, FRONT HUM NEUROSCI, V9, DOI 10.3389/fnhum.2015.00067

Pageaux B, 2014, EUR J APPL PHYSIOL, V114, P1095, DOI 10.1007/s00421-014-2838-5

Pattyn N, 2008, PHYSIOL BEHAV, V93, P369, DOI 10.1016/j.physbeh.2007.09.016

Pattyn N, 2014, INT J PSYCHOPHYSIOL, V93, P30, DOI 10.1016/j.ijpsycho.2013.03.008

Pattyn N, 2009, PSYCHOL BELG, V49, P101, DOI 10.5334/pb-49-2-3-101

Penner Ik, 2010, Int MS J, V17, P28

Petajan JH, 1996, ANN NEUROL, V39, P432, DOI 10.1002/ana.410390405

Rabinbach A., 1992, PRESS

Ralph B. C., 2017, PSYCHOL RES, V81, P432, DOI 10.1007/s00426-016-0752-7

Reeves William C, 2007, Popul Health Metr, V5, P5

Richter M, 2016, ADV MOTIV SCI, V3, P149, DOI 10.1016/bs.adms.2016.02.001

Robertson IH, 1997, NEUROPSYCHOLOGIA, V35, P747, DOI 10.1016/S0028-3932(97)00015-8

Sanders MA, 2012, PSYCHOL SCI, V23, P1470, DOI 10.1177/0956797612450034

Secher NH, 2008, J APPL PHYSIOL, V104, P306, DOI 10.1152/japplphysiol.00853.2007

Sen MS, 2016, ASIAN J PSYCHIATR, V22, P157, DOI 10.1016/j.ajp.2016.06.003

Severeijns R, 2001, CLIN J PAIN, V17, P165, DOI 10.1097/00002508-200106000-00009

Sharples SA, 2016, PLOS ONE, V11, DOI 10.1371/journal.pone.0149026

Shekleton JA, 2010, SLEEP MED REV, V14, P47, DOI 10.1016/j.smrv.2009.06.001

Shen JH, 2006, SLEEP MED REV, V10, P63, DOI 10.1016/j.smrv.2005.05.004

Singer S, 2011, BRIT J CANCER, V105, P445, DOI 10.1038/bjc.2011.251

Smallwood J, 2004, CONSCIOUS COGN, V13, P657, DOI 10.1016/j.concog.2004.06.003

Smirmaul Bruno Paula Caraça, 2013, Rev. bras. educ. fís. esporte, V27, P333

Stuss DT, 1995, ANN NY ACAD SCI, V769, P191, DOI 10.1111/j.1749-6632.1995.tb38140.x

Sullivan MJL, 2001, CLIN J PAIN, V17, P52, DOI 10.1097/00002508-200103000-00008

Temple JG, 2000, HUM FACTORS, V42, P183, DOI 10.1518/001872000779656480

Trojan DA, 2007, MULT SCLER J, V13, P985, DOI 10.1177/1352458507077175

Van Cutsem J, 2018, PSYCHOPHARMACOLOGY, V235, P947, DOI 10.1007/s00213-017-4809-0

Van Cutsem J, 2017, SPORTS MED, V47, P1569, DOI 10.1007/s40279-016-0672-0

Venhorst A., 2017, BR J SPORTS MED, V52, P957, DOI 10.1136/bjsports-2016-096907

Wolfe F, 1996, J RHEUMATOL, V23, P1407

Wright R.A., 1989, GOAL CONCEPTS PERSON

Wright R. A., 1996, PSYCHOL ACTION LINKI, P424

Yerkes RM, 1908, J COMP NEUROL PSYCHO, V18, P459, DOI 10.1002/cne.920180503

Yeung N., 2013, OXFORD HDB COGNITIVE, P275, DOI 10.1093/oxfordhb/9780199988709.013.0018

Zenon A, 2015, J NEUROSCI, V35, P8737, DOI 10.1523/JNEUROSCI.3789-14.2015

NR 123

TC 21

Z9 21

U1 1

U2 11

PU FRONTIERS MEDIA SA

PI LAUSANNE

PA AVENUE DU TRIBUNAL FEDERAL 34, LAUSANNE, CH-1015, SWITZERLAND

SN 1664-1078

J9 FRONT PSYCHOL

JI Front. Psychol.

PD SEP 10

PY 2018

VL 9

AR 1246

DI 10.3389/fpsyg.2018.01246

PG 13

WC Psychology, Multidisciplinary

WE Social Science Citation Index (SSCI)

SC Psychology

GA GT0VM

UT WOS:000444167900001

PM 30250436

OA Green Published, gold

DA 2022-05-04

ER

PT J

AU Souchet, AD

Philippe, S

Lourdeaux, D

Leroy, L

AF Souchet, Alexis D.

Philippe, Stephanie

Lourdeaux, Domitile

Leroy, Laure

TI Measuring Visual Fatigue and Cognitive Load via Eye Tracking while

Learning with Virtual Reality Head-Mounted Displays: A Review

SO INTERNATIONAL JOURNAL OF HUMAN-COMPUTER INTERACTION

LA English

DT Article

ID MENTAL WORKLOAD; TECHNOLOGY; CONVERGENCE; EXPERIENCE; ATTENTION;

QUALITY; IMAGES; 2D

AB Virtual Reality Head-Mounted Displays (HMDs) reached the consumer market and are used for learning purposes. Risks regarding visual fatigue and high cognitive load arise while using HMDs. These risks could impact learning efficiency. Visual fatigue and cognitive load can be measured with eye tracking, a technique that is progressively implemented in HMDs. Thus, we investigate how to assess visual fatigue and cognitive load via eye tracking. We conducted this review based on five research questions. We first described visual fatigue and possible cognitive overload while learning with HMDs. The review indicates that visual fatigue can be measured with blinks and cognitive load with pupil diameter based on thirty-seven included papers. Yet, distinguishing visual fatigue from cognitive load with such measures is challenging due to possible links between them. Despite measure interpretation issues, eye tracking is promising for live assessment. More researches are needed to make data interpretation more robust and document human factor risks when learning with HMDs.

C1 [Souchet, Alexis D.; Leroy, Laure] Paris 8 Univ, Paragraphe Lab, St Denis, France.

[Souchet, Alexis D.; Philippe, Stephanie] Manzalab, R&D Dept, Paris, France.

[Souchet, Alexis D.; Lourdeaux, Domitile] UTC Compiegne, UMR CNRS 7253, Heudiasyc, Compiegne, France.

[Souchet, Alexis D.; Leroy, Laure] Armed Forces Biomed Res Inst IRBA, Neurosci Dept, Bretigny Sur Orge, France.

RP Souchet, AD (corresponding author), Univ Technol Compiegne, UMR CNRS 7253, Heudiasyc, CS 60319,57 Ave Landshut, F-60203 Compiegne, France.

EM alexis.souchet@hds.utc.fr

OI Souchet, Alexis/0000-0003-4885-1392

FU French National Association of Research and Technology [CIFRE 2016-1571]

FX This work was supported by the French National Association of Research

and Technology [CIFRE 2016-1571].

CR Abdulin E., 2016, P 2016 CHI C HUM FAC, P1503, DOI DOI 10.1145/2851581.2892306

Abromavicius V, 2018, BIOCYBERN BIOMED ENG, V38, P810, DOI 10.1016/j.bbe.2018.08.001

Abromavicius V, 2017, 2017 5TH IEEE WORKSHOP ON ADVANCES IN INFORMATION, ELECTRONIC AND ELECTRICAL ENGINEERING (AIEEE'2017)

Ackerman P.L., 2011, COGNITIVE FATIGUE MU, P149164, DOI [10.1037/12343-007, DOI 10.1037/12343-007]

Adams EJ, 2018, LANG SPEECH HEAR SER, V49, P340, DOI 10.1044/2018_LSHSS-17-0114

Alca?iz, 2018, EURASIA J MATH SCI T, V14, P20452057, DOI [10.29333/ejmste/85874, DOI 10.29333/EJMSTE/85874]

Alhusuny A, 2021, SURG ENDOSC, V35, P6660, DOI 10.1007/s00464-020-08167-2

Almustanyir, 2021, INT J OPHTHALMOLOGY, V6, P10, DOI [10.11648/j.ijovs.20210601.12, DOI 10.11648/J.IJOVS.20210601.12]

Anmarkrud O, 2019, EDUC PSYCHOL-US, V54, P61, DOI 10.1080/00461520.2018.1554484

Appel T, 2018, 2018 ACM SYMPOSIUM ON EYE TRACKING RESEARCH & APPLICATIONS (ETRA 2018), DOI 10.1145/3204493.3204531

Bacher LF, 2017, INFANCY, V22, P150, DOI 10.1111/infa.12164

Baddeley A, 2010, CURR BIOL, V20, pR136, DOI 10.1016/j.cub.2009.12.014

Baekgaard P, 2019, ETRA 2019: 2019 ACM SYMPOSIUM ON EYE TRACKING RESEARCH & APPLICATIONS, DOI 10.1145/3314111.3319831

Bailey S.K.T., 2017, HCI INT 2017, P54, DOI [10.1007/978-3-319-58753-0_9, DOI 10.1007/978-3-319-58753-0_9]

Bando T, 2012, DISPLAYS, V33, P76, DOI 10.1016/j.displa.2011.09.001

Bang JW, 2014, SENSORS-BASEL, V14, P16467, DOI 10.3390/s140916467

Banks MS, 2012, SMPTE MOTION IMAG J, V121, P24, DOI 10.5594/j18173

Bednarik R, 2018, 2018 ACM SYMPOSIUM ON EYE TRACKING RESEARCH & APPLICATIONS (ETRA 2018), DOI 10.1145/3204493.3204577

Benedetto S, 2015, COMPUT HUM BEHAV, V45, P352, DOI 10.1016/j.chb.2014.12.043

Bernhardt KA, 2021, APPL ERGON, V90, DOI 10.1016/j.apergo.2020.103152

Bhavsar, 2018, COMPUTER AIDED CHEM, V44, P2347, DOI [10.1016/B978-0-444-64241-7.50386-4, DOI 10.1016/B978-0-444-64241-7.50386-4]

Bian YL, 2018, PROCEEDINGS OF THE 2018 CHI CONFERENCE ON HUMAN FACTORS IN COMPUTING SYSTEMS (CHI 2018)

Bischof H., 2009, MVA, P350

Bottenheft C, 2021, COGN TECHNOL WORK, V23, P805, DOI 10.1007/s10111-020-00653-w

Bracq MS, 2019, NURS EDUC TODAY, V79, P153, DOI 10.1016/j.nedt.2019.05.026

Bradley, 2018, ADAPTING VIRTUAL REA

Bulling A., 2014, ADV PHYSL COMPUTING, P39

Cai TT, 2017, PLOS ONE, V12, DOI 10.1371/journal.pone.0172426

Caldas OI, 2020, IEEE T NEUR SYS REH, V28, P1109, DOI 10.1109/TNSRE.2020.2985308

Camina E, 2017, FRONT PHARMACOL, V8, DOI 10.3389/fphar.2017.00438

Caplan B., 2011, ENCY CLIN NEUROPSYCH, P247, DOI [10.1007/978-0-387-79948-3_1266, DOI 10.1007/978-0-387-79948-3_1266]

Carenini, 2015, P 20 INT C INT US IN, P357, DOI [10.1145/2678025.2701376, DOI 10.1145/2678025.2701376]

Carl E, 2019, J ANXIETY DISORD, V61, P27, DOI 10.1016/j.janxdis.2018.08.003

Carter BT, 2020, INT J PSYCHOPHYSIOL, V155, P49, DOI 10.1016/j.ijpsycho.2020.05.010

Chai WJ, 2018, FRONT PSYCHOL, V9, DOI 10.3389/fpsyg.2018.00401

Chan E, 2018, PLOS ONE, V13, DOI 10.1371/journal.pone.0200987

Chang E, 2020, INT J HUM-COMPUT INT, V36, P1658, DOI 10.1080/10447318.2020.1778351

Charles RL, 2019, APPL ERGON, V74, P221, DOI 10.1016/j.apergo.2018.08.028

Chen CX, 2017, DISPLAYS, V50, P14, DOI 10.1016/j.displa.2017.09.003

Chen SY, 2014, HUM-COMPUT INTERACT, V29, P390, DOI 10.1080/07370024.2014.892428

Cho, 2012, 2012 IEEE COMP VIS P, P23, DOI [10.1109/CVPRW.2012.6238904, DOI 10.1109/CVPRW.2012.6238904]

Cho TH, 2017, DISPLAYS, V49, P59, DOI 10.1016/j.displa.2017.07.002

Ciuffreda, 2002, MODELS VISUAL SYSTEM, P341, DOI [10.1007/978-1-4757-5865-8_9, DOI 10.1007/978-1-4757-5865-8_9]

Clay V, 2019, J EYE MOVEMENT RES, V12, DOI 10.16910/jemr.12.1.3

Conti J, 2017, COMPUT GRAPH-UK, V69, P24, DOI 10.1016/j.cag.2017.08.017

Costa N., 2015, OCCUPATIONAL SAFETY, P231, DOI [10.1201/b18042-48, DOI 10.1201/B18042-48]

Critchley HD, 2018, CURR OPIN BEHAV SCI, V19, P13, DOI 10.1016/j.cobeha.2017.08.014

Csikzentmihalyi M., 1990, FLOW PSYCHOL OPTIMAL, DOI [10.5860/choice.28-0597, DOI 10.5860/CHOICE.28-0597]

Dalrymple KA, 2018, FRONT PSYCHOL, V9, DOI 10.3389/fpsyg.2018.00803

Daniel F, 2019, SCI REP-UK, V9, DOI 10.1038/s41598-018-37778-y

Das S, 2020, INT J IND ERGONOM, V80, DOI 10.1016/j.ergon.2020.103017

David S, 2014, PROCEEDINGS OF INTERNATIONAL CONFERENCE INFORMATION SYSTEMS AND DESIGN OF COMMUNICATION (ISDOC2014), P1, DOI 10.1145/2618168.2618169

de Jong T, 2010, INSTR SCI, V38, P105, DOI 10.1007/s11251-009-9110-0

Dennison MS, 2016, DISPLAYS, V44, P42, DOI 10.1016/j.displa.2016.07.002

Descheneaux Charles R., 2020, Virtual, Augmented and Mixed Reality. Design and Interaction. 12th International Conference, VAMR 2020 Held as Part of the 22nd HCI International Conference, HCII 2020. Proceedings. Lecture Notes in Computer Science (LNCS 12190), P410, DOI 10.1007/978-3-030-49695-1_27

Dobryakova E, 2013, J INT NEUROPSYCH SOC, V19, P849, DOI 10.1017/S1355617713000684

Duchowski, 2017, EYE TRACKING METHODO, DOI DOI 10.1007/978-3-319-57883-5

Duchowski AT, 2018, PROCEEDINGS OF THE 2018 CHI CONFERENCE ON HUMAN FACTORS IN COMPUTING SYSTEMS (CHI 2018), DOI 10.1145/3173574.3173856

Eckstein MK, 2017, DEV COGN NEUROS-NETH, V25, P69, DOI 10.1016/j.dcn.2016.11.001

Elias ZM, 2019, APPL ERGON, V81, DOI 10.1016/j.apergo.2019.102879

Epps J., 2018, WILEY HDB HUMAN COMP, P207, DOI 10.1002/9781118976005.ch11

Evans, 2007, PICKWELLS BINOCULAR, P12, DOI 10.1016/B978-0-7506-8897-0.50005-6

Evinger C, 2002, MOVEMENT DISORD, V17, pS75, DOI 10.1002/mds.10065

Fink G., 2016, STRESS CONCEPTS COGN, P3, DOI [10.1016/B978-0-12-800951-2.00001-7, DOI 10.1016/B978-0-12-800951-2.00001-7]

Fodor LA, 2018, SCI REP-UK, V8, DOI 10.1038/s41598-018-28113-6

Fuchs, 2017, VIRTUAL REALITY HEAD

Gabana D, 2017, INT CONF AFFECT, P36, DOI 10.1109/ACII.2017.8273576

Gilboa Y, 2021, J ATTEN DISORD, V25, P300, DOI 10.1177/1087054718808590

Goldberg JH, 2003, MIND'S EYE: COGNITIVE AND APPLIED ASPECTS OF EYE MOVEMENT RESEARCH, P493, DOI 10.1016/B978-044451020-4/50027-X

Guedes HG, 2019, INT J SURG, V61, P60, DOI 10.1016/j.ijsu.2018.12.001

Guo J, 2019, J SOC INF DISPLAY, V27, P108, DOI 10.1002/jsid.750

Guo J, 2017, P IEEE VIRT REAL ANN, P249, DOI 10.1109/VR.2017.7892270

Gyoung Kim, 2018, Augmented Cognition. Intelligent Technologies. 12th International Conference, AC 2018 Held as Part of HCI International 2018. Proceedings: LNAI 10915, P120, DOI 10.1007/978-3-319-91470-1_11

Hamari J, 2014, COMPUT HUM BEHAV, V40, P133, DOI 10.1016/j.chb.2014.07.048

Hansard, 2017, ABS170304574 CORR

HART S G, 1988, P139

Hirota M, 2019, ERGONOMICS, V62, P759, DOI 10.1080/00140139.2019.1582805

Hofler C, 2018, INT J PSYCHOPHYSIOL, V125, P29, DOI 10.1016/j.ijpsycho.2018.01.014

Hoffman DM, 2008, J VISION, V8, DOI 10.1167/8.3.33

Holleman GA, 2020, FRONT PSYCHOL, V11, DOI 10.3389/fpsyg.2020.00721

Holtzer R, 2010, AGING NEUROPSYCHOL C, V18, P108, DOI 10.1080/13825585.2010.517826

Hopstaken JF, 2016, J EXP PSYCHOL HUMAN, V42, P878, DOI 10.1037/xhp0000189

Howard MC, 2020, COMPUT EDUC, V144, DOI 10.1016/j.compedu.2019.103707

Huffing RC, 2016, 2016 ACM SYMPOSIUM ON EYE TRACKING RESEARCH & APPLICATIONS (ETRA 2016), P341, DOI 10.1145/2857491.2888586

Hupont I, 2015, 2015 7 INT WORKSHOP, P1, DOI [10.1109/qomex.2015.7148110, 10.1109/QoMEX.2015.7148110, DOI 10.1109/QOMEX.2015.7148110]

Hynes Niall J, 2018, Vision (Basel), V2, DOI 10.3390/vision2030036

Iatsun I, 2015, DISPLAYS, V39, P11, DOI 10.1016/j.displa.2015.07.001

Iatsun I, 2013, PROC SPIE, V8648, DOI 10.1117/12.2008206

Iskander J, 2019, APPL ERGON, V81, DOI 10.1016/j.apergo.2019.102883

Iskander J, 2018, IEEE ACCESS, V6, P19345, DOI 10.1109/ACCESS.2018.2815663

Jacob S, 2018, PERVASIVE EYE TRACKING AND MOBILE EYE-BASED INTERACTION (PETMEI 2018), DOI 10.1145/3208031.3208034

Jacobs J, 2019, ACM T APPL PERCEPT, V16, DOI 10.1145/3353902

John B, 2018, 24TH ACM SYMPOSIUM ON VIRTUAL REALITY SOFTWARE AND TECHNOLOGY (VRST 2018), DOI 10.1145/3281505.3281538

Johnson B., 2015, INT ENCY SOCIAL BEHA, P618, DOI [10.1016/B978-0-08-097086-8.10550-1, DOI 10.1016/B978-0-08-097086-8.10550-1]

Johnson R.B., 2015, OXFORD HDB MULTIMETH, P129, DOI [10.1093/oxfordhb/9780199933624.013.51, DOI 10.1093/OXFORDHB/9780199933624.013.51]

Jongkees BJ, 2016, NEUROSCI BIOBEHAV R, V71, P58, DOI 10.1016/j.neubiorev.2016.08.020

Kiili K, 2014, ENTERTAIN COMPUT, V5, P367, DOI 10.1016/j.entcom.2014.08.002

Kim D, 2019, COMPUT HUM BEHAV, V93, P346, DOI 10.1016/j.chb.2018.12.040

Kim D, 2011, PROC SPIE, V7863, DOI 10.1117/12.873354

Kim HK, 2018, APPL ERGON, V69, P66, DOI 10.1016/j.apergo.2017.12.016

Kim J, 2014, VISION RES, V105, P159, DOI 10.1016/j.visres.2014.10.021

Kim J, 2018, SYMMETRY-BASEL, V10, DOI 10.3390/sym10090400

Kim T, 2020, SENSORS-BASEL, V20, DOI 10.3390/s20174814

Kohnen T., 2018, ENCY OPHTHALMOLOGY, P1236, DOI [10.1007/978-3-540-69000-9_536, DOI 10.1007/978-3-540-69000-9_536]

Komogortsev, 2015, P 33 ANN ACM C HUM F, P1265, DOI [10.1145/2702613.2732812, DOI 10.1145/2702613.2732812]

Kong XJ, 2019, ADV INTELL SYST, V876, P314, DOI 10.1007/978-3-030-02053-8_48

Kosch T, 2018, PROCEEDINGS OF THE 2018 CHI CONFERENCE ON HUMAN FACTORS IN COMPUTING SYSTEMS (CHI 2018), DOI 10.1145/3173574.3174010

Krejtz K, 2018, PLOS ONE, V13, DOI 10.1371/journal.pone.0203629

Kweon SH, 2018, ADV INTELL SYST, V592, P194, DOI 10.1007/978-3-319-60366-7_19

Lackey SJ, 2016, ERGONOMICS, V59, P1060, DOI 10.1080/00140139.2015.1122234

Lambooij M, 2009, J IMAGING SCI TECHN, V53, DOI 10.2352/J.ImagingSci.Technol.2009.53.3.030201

Lanier M, 2019, COMPUT HUM BEHAV, V100, P70, DOI 10.1016/j.chb.2019.06.015

Le Callet P., 2013, 7 INT WORKSH VID P Q, P1

Lee EC, 2010, IEEE T CONSUM ELECTR, V56, P1677, DOI 10.1109/TCE.2010.5606312

Lee N., 2018, ENCY COMPUTER GRAPHI, P1, DOI [10.1007/978-3-319-08234-9_252-1, DOI 10.1007/978-3-319-08234-9_252-1]

Leigh R.J., 2015, NEUROLOGY EYE MOVEME, DOI DOI 10.1093/MED/9780199969289.001.0001

Lenskiy Artem, 2016, 2016 IEEE Conference on Norbert Wiener in the 21st Century (21CW). Proceedings, DOI 10.1109/NORBERT.2016.7547466

Lensky T, 2015, 2 ADV INF TECHN INT

Leppink J, 2017, J TAIBAH UNIV MED SC, V12, P385, DOI 10.1016/j.jtumed.2017.05.003

Leroy, 2016, EYESTRAIN REDUCTION, DOI [10.1002/9781119318330, DOI 10.1002/9781119318330]

Leva M.C., 2019, HUMAN MENTAL WORKLOA, P2348, DOI [10.1007/978-3-030-14273-5_3, DOI 10.1007/978-3-030-14273-5_3, 10.1007/978-3-030- 14273-5_3]

Lin CJ, 2018, APPL ERGON, V69, P10, DOI 10.1016/j.apergo.2017.12.020

Lindner P, 2019, FRONT PSYCHOL, V10, DOI 10.3389/fpsyg.2019.00132

Liu P, 2018, ATTEN PERCEPT PSYCHO, V80, P929, DOI 10.3758/s13414-017-1464-9

Lohr DJ, 2016, 2016 ACM SYMPOSIUM ON EYE TRACKING RESEARCH & APPLICATIONS (ETRA 2016), P315, DOI 10.1145/2857491.2884058

Long Y, 2020, SEMIN OPHTHALMOL, V35, P170, DOI 10.1080/08820538.2020.1776342

Loup-Escande E, 2017, INT J HUM-COMPUT INT, V33, P115, DOI 10.1080/10447318.2016.1220105

Lourdeaux, 2018, VIRTUAL REALITY AUGM, P171, DOI [10.1002/9781119341031.ch1, DOI 10.1002/9781119341031.CH1]

Soler JL, 2017, INTED PROC, P8684

Luo, 2016, SID S, V47, P903, DOI [10.1002/sdtp.10839, DOI 10.1002/SDTP.10839]

Luro FL, 2019, ETRA 2019: 2019 ACM SYMPOSIUM ON EYE TRACKING RESEARCH & APPLICATIONS, DOI 10.1145/3317956.3318153

Matsuura Y, 2019, CURR TOP ENV HEAL PR, P89, DOI 10.1007/978-981-13-1601-2_8

Matthews G, 2020, THEOR ISS ERGON SCI, V21, P369, DOI 10.1080/1463922X.2018.1547459

Midha S, 2021, INT J HUM-COMPUT ST, V147, DOI 10.1016/j.ijhcs.2020.102580

Milivojevich, 2016, QUALITY EXPT DESIGN

Mittelstaedt JM, 2019, VIRTUAL REAL-LONDON, V23, P143, DOI 10.1007/s10055-018-0370-3

Mon-Williams M, 1998, HUM FACTORS, V40, P42, DOI 10.1518/001872098779480622

MONWILLIAMS M, 1993, OPHTHAL PHYSL OPT, V13, P387, DOI 10.1111/j.1475-1313.1993.tb00496.x

Moreno R, 2010, INSTR SCI, V38, P135, DOI 10.1007/s11251-009-9122-9

Mun S, 2012, NEUROSCI LETT, V525, P89, DOI 10.1016/j.neulet.2012.07.049

Munsamy AJ, 2020, J OPTOM, V13, P163, DOI 10.1016/j.optom.2020.02.004

Murray, 2016, 2016 8 INT C QUAL MU, P1, DOI [10.1109/QoMEX.2016.7498964, DOI 10.1109/QOMEX.2016.7498964]

Nesbitt K, 2017, DISPLAYS, V48, P1, DOI 10.1016/j.displa.2017.01.002

Neveu P, 2016, INVEST OPHTH VIS SCI, V57, P4321, DOI 10.1167/iovs.15-18854

Paprocki R, 2017, FRONT HUM NEUROSCI, V11, DOI 10.3389/fnhum.2017.00620

Parent M, 2019, INT J PSYCHOPHYSIOL, V146, P139, DOI 10.1016/j.ijpsycho.2019.09.005

Parikh Kunjal, 2018, SID Symposium Digest of Technical Papers, V49, P502, DOI 10.1002/sdtp.12611

Park, 2011, 17 DSP 2011 INT C DI, P1, DOI [10.1109/ICDSP.2011.6004997, DOI 10.1109/ICDSP.2011.6004997]

Park MC, 2015, J DISP TECHNOL, V11, P877, DOI 10.1109/JDT.2015.2389212

Park S, 2015, INT J PSYCHOPHYSIOL, V97, P120, DOI 10.1016/j.ijpsycho.2015.04.006

Park S, 2019, APPL ERGON, V78, P26, DOI 10.1016/j.apergo.2019.01.014

Parong J, 2018, J EDUC PSYCHOL, V110, P785, DOI 10.1037/edu0000241

Patney A, 2016, ACM T GRAPHIC, V35, DOI 10.1145/2980179.2980246

Pautasso M, 2013, PLOS COMPUT BIOL, V9, DOI 10.1371/journal.pcbi.1003149

Peinkhofer C, 2019, PEERJ, V7, DOI 10.7717/peerj.6882

Peissl S, 2018, INT J AEROSP PSYCHOL, V28, P98, DOI 10.1080/24721840.2018.1514978

Peitek N, 2018, EYE MOVEMENTS IN PROGRAMMING (EMIP 2018), DOI 10.1145/3216723.3216726

Perttula A, 2017, INT J SERIOUS GAMES, V4, P57, DOI 10.17083/ijsg.v4i1.151

Phillips F., 2016, P ACM S APPL PERC, P15, DOI [10.1145/2931002.2931020, DOI 10.1145/2931002.2931020]

Porcino T, 2020, IEEE INT CONF SERIOU

Portelli M, 2020, ANN ROY COLL SURG, V102, P672, DOI 10.1308/rcsann.2020.0178

Puma S, 2018, INT J PSYCHOPHYSIOL, V123, P111, DOI 10.1016/j.ijpsycho.2017.10.004

Rac-Lubashevsky R, 2017, SCI REP-UK, V7, DOI 10.1038/s41598-017-02942-3

Rebenitsch L, 2021, VIRTUAL REAL-LONDON, V25, P165, DOI 10.1007/s10055-020-00446-6

Reichelt S, 2010, PROC SPIE, V7690, DOI 10.1117/12.850094

Ro Y.M., 2011, P INT C DIG SIGN PRO, P1, DOI 10.1109/ICDSP.2011.6004985

Rodriguez JD, 2018, CURR EYE RES, V43, P52, DOI 10.1080/02713683.2017.1381270

Rotter P, 2017, IEEE TECHNOL SOC MAG, V36, P81, DOI 10.1109/MTS.2017.2654294

RUSHTON S, 1994, DISPLAYS, V15, P255, DOI 10.1016/0141-9382(94)90073-6

SCHOR CM, 1992, OPTOMETRY VISION SCI, V69, P258, DOI 10.1097/00006324-199204000-00002

Schut MJ, 2017, J VISION, V17, DOI 10.1167/17.6.15

Selzer MN, 2019, DISPLAYS, V59, P9, DOI 10.1016/j.displa.2019.04.002

Sharek D, 2014, SIMULAT GAMING, V45, P569, DOI 10.1177/1046878114554176

Sharma D., 2018, BASIC SCI ANESTHESIA, P355, DOI [10.1007/978-3-319-62067-1_19, DOI 10.1007/978-3-319-62067-1_19]

Shen, 2019, IMAGE GRAPHICS TECHN, P310, DOI [10.1007/978-981-13-9917-6_30, DOI 10.1007/978-981-13-9917-6_30]

Sheppard AL, 2018, BMJ OPEN OPHTHALMOL, V3, DOI 10.1136/bmjophth-2018-000146

Shin D, 2018, COMPUT HUM BEHAV, V78, P64, DOI 10.1016/j.chb.2017.09.012

Sidman, 2005, GAMING TRAINING REV, DOI [10.1037/e500852012-001, DOI 10.1037/E500852012-001]

Sirois S, 2014, WIRES COGN SCI, V5, P679, DOI 10.1002/wcs.1323

Skaramagkas Vasileios, 2021, IEEE Rev Biomed Eng, VPP, DOI 10.1109/RBME.2021.3066072

Slater M, 2004, PRESENCE-VIRTUAL AUG, V13, P484, DOI 10.1162/1054746041944849

Slater M, 2009, PHILOS T R SOC B, V364, P3549, DOI 10.1098/rstb.2009.0138

Soler-Dominguez JL, 2017, LECT NOTES COMPUT SC, V10280, P369, DOI 10.1007/978-3-319-57987-0_30

Souchet AD, 2019, INT SYM MIX AUGMENT, P328, DOI 10.1109/ISMAR.2019.00031

Souchet AD, 2018, 24TH ACM SYMPOSIUM ON VIRTUAL REALITY SOFTWARE AND TECHNOLOGY (VRST 2018), DOI 10.1145/3281505.3281509

Spitzer M, 2014, TRENDS NEUROSCI EDUC, V3, P81, DOI 10.1016/j.tine.2014.09.002

Stanney K, 2020, INT J HUM-COMPUT INT, V36, P1783, DOI 10.1080/10447318.2020.1828535

Steed A, 2016, P IEEE VIRT REAL ANN, P67, DOI 10.1109/VR.2016.7504689

Stein N, 2021, I-PERCEPTION, V12, DOI 10.1177/2041669520983338

STERN JA, 1994, HUM FACTORS, V36, P285, DOI 10.1177/001872089403600209

Stone RJ, 2016, PRESENCE-TELEOP VIRT, V25, P151, DOI 10.1162/PRES_a_00253

Stratton SJ, 2016, PREHOSP DISASTER MED, V31, P347, DOI 10.1017/S1049023X16000649

Suh A, 2018, COMPUT HUM BEHAV, V86, P77, DOI 10.1016/j.chb.2018.04.019

Suk H.-J., 2017, ELECT IMAGING, V2017, P212, DOI [10.2352/.2470-1173.2017.14.hvei-146, DOI 10.2352/ISSN.2470-1173.2017.14.HVEI-146]

Sweller J, 2016, P 47 ACM TECHN S COM, DOI [10.1145/2839509.2844549, DOI 10.1145/2839509.2844549]

Sweller J, 2011, PSYCHOL LEARN MOTIV, V55, P37

Szpak A, 2020, J MED INTERNET RES, V22, DOI 10.2196/19840

Terzis, 2016, HDB CAMERA MONITOR S, P279, DOI [10.1007/978-3-319-29611-1_9, DOI 10.1007/978-3-319-29611-1_9]

Thai KTP, 2020, 2020 IEEE CONFERENCE ON VIRTUAL REALITY AND 3D USER INTERFACES WORKSHOPS (VRW 2020), P468, DOI 10.1109/VRW50115.2020.0-177

Toosi, 2015, P 4 INT S PERV DISPL, P269, DOI 10.1145/2757710.2776816

Tozman T, 2015, COMPUT HUM BEHAV, V52, P408, DOI 10.1016/j.chb.2015.06.023

Turner J.R., 2013, ENCY BEHAV MED, P1436, DOI [10.1007/978-1-4419-1005-9_822, DOI 10.1007/978-1-4419-1005-9_822]

Turner J.R., 2013, ENCY BEHAV MED, P1943, DOI 10.1007/978-1-4419-1005-9_853

Turner J.R., 2013, ENCY BEHAV MED, P165, DOI [10.1007/978-1-4419-1005-9_790, DOI 10.1007/978-1-4419-1005-9_790]

Ukai K, 2008, DISPLAYS, V29, P106, DOI 10.1016/j.displa.2007.09.004

Urvoy M, 2013, ANN TELECOMMUN, V68, P641, DOI 10.1007/s12243-013-0394-3

Van Acker BB, 2018, COGN TECHNOL WORK, V20, P351, DOI 10.1007/s10111-018-0481-3

Vera J, 2017, OPTOMETRY VISION SCI, V94, P797, DOI 10.1097/OPX.0000000000001105

Vienne C, 2012, PROC SPIE, V8288, DOI 10.1117/12.906994

Wang Y, 2019, BIOMED ENG ONLINE, V18, DOI 10.1186/s12938-019-0731-5

Wang Y, 2018, IEEE ACCESS, V6, P55948, DOI 10.1109/ACCESS.2018.2869624

Windhorst, 2009, ENCY NEUROSCIENCE, P10, DOI [10.1007/978-3-540-29678-2_31, DOI 10.1007/978-3-540-29678-2_31]

Wise RA, 2004, NAT REV NEUROSCI, V5, P483, DOI 10.1038/nrn1406

WONG MMH, 1991, J PERS, V59, P539, DOI 10.1111/j.1467-6494.1991.tb00259.x

Yamada Y, 2017, 2017 IEEE INTERNATIONAL CONFERENCE ON HEALTHCARE INFORMATICS (ICHI), P275, DOI 10.1109/ICHI.2017.74

Yang XZ, 2019, J EDUC COMPUT RES, V57, P846, DOI 10.1177/0735633118770800

Yoon HJ, 2020, BMC OPHTHALMOL, V20, DOI 10.1186/s12886-020-01471-4

Yu XY, 2018, ADJUNCT PROCEEDINGS OF THE 2018 IEEE INTERNATIONAL SYMPOSIUM ON MIXED AND AUGMENTED REALITY (ISMAR), P93, DOI 10.1109/ISMAR-Adjunct.2018.00042

Yuan J., 2018, INT J OPHTHALMOLOGY, V5, DOI [10.23937/2378-346X/1410085, DOI 10.23937/2378-346X/1410085]

Yue K, 2018, J SOC INF DISPLAY, V26, P427, DOI 10.1002/jsid.667

Yunhong Zhang, 2020, Advances in Physical, Social & Occupational Ergonomics. Proceedings of the AHFE 2020 Virtual Conferences on Physical Ergonomics and Human Factors, Social & Occupational Ergonomics and Cross-Cultural Decision Making. Advances in Intelligent Systems and Computing (1215), P213, DOI 10.1007/978-3-030-51549-2_28

Zagermann J, 2018, CHI 2018: EXTENDED ABSTRACTS OF THE 2018 CHI CONFERENCE ON HUMAN FACTORS IN COMPUTING SYSTEMS, DOI 10.1145/3170427.3188628

Zagermann J, 2016, BEYOND TIME AND ERRORS: NOVEL EVALUATION METHODS FOR VISUALIZATION, BELIV 2016, P78, DOI 10.1145/2993901.2993908

ZEMBLYS R, 2018, P 2018 ACM S EYE TRA, V83, P1, DOI DOI 10.1145/3204493.3208341

Zeri F, 2015, OPHTHAL PHYSL OPT, V35, P271, DOI 10.1111/opo.12194

Zhang YH, 2015, LECT NOTES ARTIF INT, V9174, P84, DOI 10.1007/978-3-319-20373-7_9

Zhao GJ, 2021, ANN TRANSL MED, V9, DOI 10.21037/atm-20-2785

Zhou J, 2019, DIGIT SIGNAL PROCESS, V91, P41, DOI 10.1016/j.dsp.2018.12.008

Zu TL, 2017, PHYS EDUC RES CONF, P472, DOI 10.1119/perc.2017.pr.113

NR 229

TC 1

Z9 1

U1 33

U2 40

PU TAYLOR & FRANCIS INC

PI PHILADELPHIA

PA 530 WALNUT STREET, STE 850, PHILADELPHIA, PA 19106 USA

SN 1044-7318

EI 1532-7590

J9 INT J HUM-COMPUT INT

JI Int. J. Hum.-Comput. Interact.

PD MAY 28

PY 2022

VL 38

IS 9

BP 801

EP 824

DI 10.1080/10447318.2021.1976509

EA OCT 2021

PG 24

WC Computer Science, Cybernetics; Ergonomics

WE Science Citation Index Expanded (SCI-EXPANDED); Social Science Citation Index (SSCI)

SC Computer Science; Engineering

GA 0U3KO

UT WOS:000702253900001

DA 2022-05-04

ER

PT J

AU Torres, Y

Nadeau, S

Landau, K

AF Torres, Yaniel

Nadeau, Sylvie

Landau, Kurt

TI Evaluation of Fatigue and Workload among Workers Conducting Complex

Manual Assembly in Manufacturing

SO IISE TRANSACTIONS ON OCCUPATIONAL ERGONOMICS & HUMAN FACTORS

LA English

DT Article

DE Production demand; shift work; sleep; mental demand; subjective metrics

ID NASA-TLX; HUMAN-PERFORMANCE; MENTAL WORKLOAD; SLEEP; QUALITY;

ERGONOMICS; TASK; INSTRUCTIONS; PRINCIPLES; HEALTH

AB OCCUPATIONAL APPLICATIONS

We conducted a study to evaluate fatigue and workload among workers performing complex assembly tasks. We investigate several predictors of fatigue, including subjective workload estimates, sleep duration, the shift being worked, and production levels. High levels of fatigue were reported in one-third of the shifts evaluated. The main predictors of high fatigue were workload estimates, working evening shifts, and baseline fatigue. Among the six dimensions of workload, only mental demand and frustration were predictors of high fatigue. Mental demand was also rated highest. Participants reported less than seven hours of sleep in 60% of the nights evaluated. These results suggest that managers and supervisors should consider cognitive workload as a key contributing factor to fatigue in complex manual assembly. Similarly, work schedule planning should consider shift duration, start times, and end times, because of the negative influence on fatigue and the potential disruptions on sleep among workers.

TECHNICAL ABSTRACT

Background: General fatigue and mental workload have been studied extensively in safety-critical contexts; wherein human performance degradation can lead to catastrophic outcomes. In the manufacturing sector, the physical demands of a job have received most of the attention because of the presence of biomechanical loads and the incidence of musculoskeletal disorders. However, in complex manual assembly, cognitive and chronobiology aspects of work can contribute to fatigue and degrade worker performance.

Purpose: We aimed to evaluate self-reported levels of fatigue and workload among a group of workers performing complex assembly tasks. We also sought to investigate several predictors of fatigue, including workload estimates, sleep duration, the shift being worked, and production levels.

Methods: Fourteen assembly line workers participated in a two-week study. They evaluated their levels of fatigue at the beginning and end of each shift using the Samn-Perelli Fatigue Scale. They also evaluated their workload according to the NASA-TLX scale at the end of each shift.

Results: High levels of fatigue (fatigue score >= 5) were reported in approximately one-third of 114 work shifts evaluated. Binary logistic regression indicated that fatigue scores at the beginning of the shift, NASA-TLX scores, and working evening shifts were significant predictors of high levels of fatigue. Among the six dimensions measured by NASA-TLX, only mental demand and frustration were predictors of high fatigue. Mental demand was also rated highest by the workers. Participants reported less than seven hours of sleep in 60% of the nights evaluated.

Conclusions: These results suggest that cognitive load can contribute to fatigue in complex manual assembly work. Circadian and homeostatic processes related to shift duration, start times, and end times are also potential contributing factors. Similarly, existing work schedules may be contributing to sleep disruptions among workers.

C1 [Torres, Yaniel; Nadeau, Sylvie; Landau, Kurt] Ecole Technol Super, Dept Mech Engn, Montreal, PQ, Canada.

[Landau, Kurt] Tech Univ Darmstadt, Inst Ergon & Human Factors, Darmstadt, Germany.

RP Torres, Y (corresponding author), Ecole Technol Super, Dept Mech Engn, Montreal, PQ, Canada.

EM yaniel.torres-medina.1@ens.etsmtl.ca

FU MITACS [IT12360]; Natural Sciences and Engineering Research Council of

Canada (NSERC)Natural Sciences and Engineering Research Council of

Canada (NSERC)

FX This research was funded by MITACS, grant number IT12360 and the Natural

Sciences and Engineering Research Council of Canada (NSERC).

CR Ahsberg E, 1997, INT J IND ERGONOM, V20, P121, DOI 10.1016/S0169-8141(96)00044-3

AKERSTEDT T, 1990, INT J NEUROSCI, V52, P29, DOI 10.3109/00207459008994241

Andlauer P., 1981, NIGHT SHIFT WORK BIO, P1

Annett J, 2002, ERGONOMICS, V45, P966, DOI 10.1080/00140130210166951

Arellano J.L.H., 2015, INT J PHYS MED REHAB, V3, P315, DOI [10.4172/2329-9096.1000315, DOI 10.4172/2329-9096.1000315]

Bao Stephen, 2015, Handb Clin Neurol, V131, P367, DOI 10.1016/B978-0-444-62627-1.00019-6

Barker LM, 2011, ERGONOMICS, V54, P815, DOI 10.1080/00140139.2011.597878

Binoosh SA, 2017, S AFR J IND ENG, V28, P164, DOI 10.7166/28-1-1697

Blais G., 2011, METHODOLOGIE SELECTI

Boivin DB, 2014, PATHOL BIOL, V62, P292, DOI 10.1016/j.patbio.2014.08.001

Boothroyd G, 1996, DESIGN X CONCURRENT, P19, DOI DOI 10.1007/978-94-011-3985-4

Bosch T, 2011, ERGONOMICS, V54, P154, DOI 10.1080/00140139.2010.538723

Botti L, 2017, COMPUT IND ENG, V111, P481, DOI 10.1016/j.cie.2017.05.011

Boudreau P, 2018, CHRONOBIOL INT, V35, P773, DOI 10.1080/07420528.2018.1466796

Brolin A, 2017, PROD MANUF RES, V5, P141, DOI 10.1080/21693277.2017.1374893

Bruder R, 2009, INDUSTRIAL ENGINEERING AND ERGONOMICS: VISIONS, CONCEPTS, METHODS AND TOOLS - FESTSCHRIFT IN HONOR OF PROFESSOR HOLGER LUCZAK, P383, DOI 10.1007/978-3-642-01293-8_29

Bubb H, 2005, HUM FACTOR ERGON MAN, V15, P353, DOI 10.1002/hfm.20032

Correia D, 2018, PROCEDIA MANUF, V17, P663, DOI 10.1016/j.promfg.2018.10.115

Dorrian J, 2011, APPL ERGON, V42, P202, DOI 10.1016/j.apergo.2010.06.009

Eklund J, 1997, ERGONOMICS, V40, P982, DOI 10.1080/001401397187559

Fagerland MW, 2012, BMC MED RES METHODOL, V12, DOI 10.1186/1471-2288-12-78

Falck AC, 2016, PROC CIRP, V44, P424, DOI 10.1016/j.procir.2016.02.152

Falck AC, 2014, INT J IND ERGONOM, V44, P455, DOI 10.1016/j.ergon.2014.02.001

Ferguson SA, 2011, APPL ERGON, V42, P210, DOI 10.1016/j.apergo.2010.06.010

FOLKARD S, 1993, ERGONOMICS, V36, P85, DOI 10.1080/00140139308967858

Folkard S., 2006, DEV FATIGUE RISK IND

Folkard S., 2007, SOMNOL SCHLAFFORSCHU, V11, P177, DOI [10.1007/s11818-007-0308-6, DOI 10.1007/S11818-007-0308-6]

FRIZELLE G, 1995, INT J OPER PROD MAN, V15, P26, DOI 10.1108/01443579510083640

Geng JH, 2015, J COMPUT INF SCI ENG, V15, DOI 10.1115/1.4029753

Glemser S., 2017, IND 4 0 STARTING NEX

Glock CH, 2019, INT J PROD ECON, V207, P107, DOI 10.1016/j.ijpe.2018.09.022

Gomes de Carvalho Larissa Maria, 2021, Proceedings of the 21st Congress of the International Ergonomics Association (IEA 2021). Sector Based Ergonomics. Lecture Notes in Networks and Systems (LNNS 221), P136, DOI 10.1007/978-3-030-74608-7_18

HART S G, 1988, P139

Hart S.G, 2006, P HUM FACT ERG SOC A, V50, P904

Harvey AG, 2008, SLEEP, V31, P383, DOI 10.1093/sleep/31.3.383

Honn KA, 2019, ACCIDENT ANAL PREV, V126, P191, DOI 10.1016/j.aap.2018.02.013

Honn KA, 2019, IND HEALTH, V57, P264, DOI 10.2486/indhealth.SW-8

Honn KA, 2016, ACCIDENT ANAL PREV, V86, P199, DOI 10.1016/j.aap.2015.10.005

ICAO, 2019, MAN OV FAT MAN APPR

Ingre M, 2008, CHRONOBIOL INT, V25, P349, DOI 10.1080/07420520802110704

Judt D, 2020, P I MECH ENG B-J ENG, V234, P840, DOI 10.1177/0954405419883047

Karran AJ, 2019, FRONT HUM NEUROSCI, V13, DOI 10.3389/fnhum.2019.00393

Karwowski W., 2006, INT ENCY ERGONOMICS, DOI [https://doi.org/10.1201/9780849375477.ch166, DOI 10.1201/9780849375477.CH166]

KNAUTH P, 1980, INT ARCH OCC ENV HEA, V46, P167, DOI 10.1007/BF00378195

Kolus A, 2018, APPL ERGON, V73, P55, DOI 10.1016/j.apergo.2018.05.010

Kong FS, 2019, INT J PROD RES, V57, P2429, DOI 10.1080/00207543.2018.1519266

Kuzgunkaya O, 2006, INT J FLEX MANUF SYS, V18, P145, DOI 10.1007/s10696-006-9012-2

Lamond N, 1999, J SLEEP RES, V8, P255, DOI 10.1046/j.1365-2869.1999.00167.x

Laurig, 1975, 5221E EU EUR COMM EU

LEPLAT J, 1967, ANN PSYCHOL, V67, P255

Leva, 2017, HUMAN MENTAL WORKLOA, VVol 726, P90, DOI DOI 10.1007/978-3-319-61061-0_6

Li D, 2018, PROCEDIA MANUF, V25, P628, DOI 10.1016/j.promfg.2018.06.092

Mansikka H, 2019, ERGONOMICS, V62, P246, DOI 10.1080/00140139.2018.1471159

Marras W.S., 2003, OCCUPATIONAL ERGONOM, P20

Mattsson S, 2020, COMPUT IND ENG, V139, DOI 10.1016/j.cie.2018.08.011

Mattsson S, 2018, PROC CIRP, V76, P42, DOI 10.1016/j.procir.2018.02.011

MAY J, 1988, PERS INDIV DIFFER, V9, P831, DOI 10.1016/0191-8869(88)90075-X

Medbo L, 2003, INT J IND ERGONOM, V31, P263, DOI 10.1016/S0169-8141(02)00220-2

Moreno CRC, 2019, IND HEALTH, V57, P139, DOI 10.2486/indhealth.SW-1

Narvaez A., 1986, P 10 PSYCH DOD S, P514

NEVILLE KJ, 1994, HUM FACTORS, V36, P339, DOI 10.1177/001872089403600213

Norman G, 2010, ADV HEALTH SCI EDUC, V15, P625, DOI 10.1007/s10459-010-9222-y

Noyes JM, 2007, ERGONOMICS, V50, P514, DOI 10.1080/00140130701235232

Petzold P., 1982, PSYCHOPHYSICAL JUDGE, P25, DOI DOI 10.1016/J.ERGON.2009.11.006

Powell DMC, 2011, AVIAT SPACE ENVIR MD, V82, P1037, DOI 10.3357/ASEM.3115.2011

Puspawardhani EH, 2016, ADV INTELL SYST, V491, P311, DOI 10.1007/978-3-319-41929-9_29

RASMUSSEN J, 1983, IEEE T SYST MAN CYB, V13, P257, DOI 10.1109/TSMC.1983.6313160

Reason J, 2000, BMJ-BRIT MED J, V320, P768, DOI 10.1136/bmj.320.7237.768

Redeker NS, 2019, J CLIN SLEEP MED, V15, P649, DOI 10.5664/jcsm.7734

Richards Brandon D., 2018, Proceedings of the Human Factors and Ergonomics Society Annual Meeting, V62, P1599, DOI 10.1177/1541931218621361

Richardson M, 2006, HUM FACTORS, V48, P511, DOI 10.1518/001872006778606868

Riffenburgh, 2012, STAT MED, P325, DOI [10.1016/B978-0-12-384864-2.00015-9.Statistique, DOI 10.1016/B978-0-12-384864-2.00015-9, https://doi.org/10.1016/B978-0-12-384864-2.00015-9]

Rosenqvist M, 2014, PROC CIRP, V23, P98, DOI 10.1016/j.procir.2014.10.072

Rubio S, 2004, APPL PSYCHOL-INT REV, V53, P61, DOI 10.1111/j.1464-0597.2004.00161.x

Said S, 2020, J MED INTERNET RES, V22, DOI 10.2196/19472

Samn S. W., 1982, SAMTR8221 USAF

Skinner N, 2015, IND HEALTH, V53, P417, DOI 10.2486/indhealth.2015-0009

Statistics D.a, 2017, SHORT SLEEP DUR US A

Stiger TR, 1998, COMMUN STAT-SIMUL C, V27, P357, DOI 10.1080/03610919808813485

Swift K.G., 2013, MANUFACTURING PROCES, P281

Thorvald P, 2019, ROBOT CIM-INT MANUF, V59, P252, DOI 10.1016/j.rcim.2019.04.012

Torres Y., 2016, Occupational Ergonomics, V13, P79, DOI 10.3233/OER-160240

UK-CAA, 2007, AIRCR FAT REV RES UN

Watson NF, 2015, SLEEP, V38, P843, DOI 10.5665/sleep.4716

Williamson A, 2011, ACCIDENT ANAL PREV, V43, P498, DOI 10.1016/j.aap.2009.11.011

Wong IS, 2019, IND HEALTH, V57, P228, DOI 10.2486/indhealth.SW-6

Yang ST, 2020, INT J IND ERGONOM, V77, DOI 10.1016/j.ergon.2020.102954

Yilmaz D, 2017, Curr Health Sci J, V43, P20, DOI 10.12865/CHSJ.43.01.03

Young MS, 2015, ERGONOMICS, V58, P1, DOI 10.1080/00140139.2014.956151

Yung M, 2020, APPL ERGON, V82, DOI 10.1016/j.apergo.2019.102919

Zadeh RS, 2018, HERD-HEALTH ENV RES, V11, P72, DOI 10.1177/1937586717729349

Zhu XW, 2008, J MANUF SCI E-T ASME, V130, DOI 10.1115/1.2953076

NR 92

TC 0

Z9 0

U1 1

U2 1

PU TAYLOR & FRANCIS INC

PI PHILADELPHIA

PA 530 WALNUT STREET, STE 850, PHILADELPHIA, PA 19106 USA

SN 2472-5838

EI 2472-5846

J9 IISE T OCCUP ERG HUM

JI IISE Trans. Occup. Ergon. Hum. Factors

PD JAN 2

PY 2021

VL 9

IS 1

BP 49

EP 63

DI 10.1080/24725838.2021.1997835

EA OCT 2021

PG 15

WC Ergonomics

WE Emerging Sources Citation Index (ESCI)

SC Engineering

GA XC4XK

UT WOS:000718229200001

PM 34706621

DA 2022-05-04

ER

PT J

AU Osaki, T

Morikawa, T

Kajita, H

Kobayashi, N

Kondo, K

Maeda, K

AF Osaki, Tohmi

Morikawa, Takako

Kajita, Hiroyuki

Kobayashi, Nobuyuki

Kondo, Kazuhiro

Maeda, Kiyoshi

TI Caregiver burden and fatigue in caregivers of people with dementia:

Measuring human herpesvirus (HHV)-6 and-7 DNA levels in saliva

SO ARCHIVES OF GERONTOLOGY AND GERIATRICS

LA English

DT Article

DE Dementia; Caregivers; Fatigue; Human herpesvirus 6; Chalder Fatigue

Scale; Psychological stress

ID PHYSICAL-ACTIVITY; ALZHEIMERS-DISEASE; FAMILY CAREGIVERS; SCALE; RISK;

VALIDITY; CANCER

AB Purpose: We examined chronic fatigue, which has not been investigated in detail, in family caregivers for people with dementia.

Methods and materials: Forty-four community-dwelling family caregivers (the caregiver group: CG) and 50 elderly control participants (the non-caregiver group: NCG) participated in this study. We measured salivary human herpesvirus (HHV)-6 and -7 DNA levels and the Chalder fatigue scale (CFS) to assess levels of fatigue; we also measured the Center for Epidemiologic Studies-Depression Scale, Physical Activity Scale for the Elderly, Zarit Caregiver Burden Interview, Mini-Mental State Examination, Assessment of Motor and Process Skills, and Dementia Behavior Disturbance Scale.

Results: For CG, the salivary HHV-6 DNA levels and CFS scores were significantly higher than those in NCG. The salivary HHV-6 DNA levels in CG were significantly correlated with depressive symptoms, the cognitive function of the patients, and the activities of daily living/instrumental activities of daily living (ADL/IADL) abilities of the patients. The CFS scores in CG significantly correlated with caregiver burden, depression symptoms, leisure physical activity, the number of other family caregivers, and the hours spent for caregiving per week, as well as with behavior disturbances and ADL/IADL abilities.

Conclusions: The salivary HHV-6 DNA levels may be added as a new biomarker for caregiver exhaustion. We concluded that fatigue assessments should be performed by not only a questionnaire, such as the CFS, but also by a biomarker search, such as HHV-6, when estimating the caregiver burden for family caregivers of people with dementia. (C) 2016 Elsevier Ireland Ltd. All rights reserved.

C1 [Osaki, Tohmi] Kobe Univ Hosp, Med Ctr Dementia, Chuo Ku, 7-5-2 Kusunoki Cho, Kobe, Hyogo 6500017, Japan.

[Osaki, Tohmi; Morikawa, Takako; Kajita, Hiroyuki; Maeda, Kiyoshi] Kobe Gakuin Univ, Fac Rehabil, Nishi Ku, 518 Arise,Ikawadani Cho, Kobe, Hyogo 6512180, Japan.

[Kobayashi, Nobuyuki; Kondo, Kazuhiro] Jikei Univ, Dept Virol, Sch Med, Minato Ku, 3-25-8 Nishi Shimbashi, Tokyo 1058461, Japan.

RP Osaki, T (corresponding author), Kobe Univ Hosp, Med Ctr Dementia, Chuo Ku, 7-5-2 Kusunoki Cho, Kobe, Hyogo 6500017, Japan.

EM tohmiiiii@gmail.com; maedak@reha.kobegakuin.ac.jp

RI Kobayashi, Nobuyuki/AAS-9795-2021

OI Kobayashi, Nobuyuki/0000-0001-8627-515X; Osaki,

Tohmi/0000-0001-8162-6811

FU Ministry of Education, Culture, Sports, Science and Technology (MEXT)

KAKENHIMinistry of Education, Culture, Sports, Science and Technology,

Japan (MEXT)Japan Society for the Promotion of ScienceGrants-in-Aid for

Scientific Research (KAKENHI) [26461758]; MEXTMinistry of Education,

Culture, Sports, Science and Technology, Japan (MEXT) [S1201032]

FX We thank all participants for their cooperation. This study was

supported by the Ministry of Education, Culture, Sports, Science and

Technology (MEXT) KAKENHI Grant Number 26461758 and MEXT-Supported

Program for the Strategic Research Foundation at Private Universities

[grant number S1201032].

CR Arai Y, 2014, AGING MENT HEALTH, V18, P81, DOI 10.1080/13607863.2013.787045

Brodaty Henry, 2009, Dialogues Clin Neurosci, V11, P217

CHALDER T, 1993, J PSYCHOSOM RES, V37, P147, DOI 10.1016/0022-3999(93)90081-P

Chattillion EA, 2013, HEALTH PSYCHOL, V32, P793, DOI 10.1037/a0029412

Dauphinot V, 2015, J ALZHEIMERS DIS, V44, P907, DOI 10.3233/JAD-142337

Fisher AG, 2012, ASSESSMENT MOTOR PRO

FOLSTEIN MF, 1975, J PSYCHIAT RES, V12, P189, DOI 10.1016/0022-3956(75)90026-6

Fukuda S., 2015, PLOS ONE, V10

Gautheret-Dejean A, 2002, J VIROL METHODS, V100, P27, DOI 10.1016/S0166-0934(01)00390-1

Hagiwara A, 2008, GERIATR GERONTOL INT, V8, P143, DOI 10.1111/j.1447-0594.2008.00463.x

Hara S, 2002, MICROBIOL IMMUNOL, V46, P177, DOI 10.1111/j.1348-0421.2002.tb02683.x

Hirano A, 2011, ARCH GERONTOL GERIAT, V52, P295, DOI 10.1016/j.archger.2010.04.011

Ito T, 2014, NUTR CANCER, V66, P377, DOI 10.1080/01635581.2014.884232

Kamiya M, 2014, GERIATR GERONTOL INT, V14, P45, DOI 10.1111/ggi.12260

Kondo K., 2009, IGAKUNO AYUMI, V228, P664

Kondo Kazuhiro, 2007, Nihon Rinsho, V65, P1043

Kondo K, 2007, HUMAN HERPESVIRUSES: BIOLOGY, THERAPY, AND IMMUNOPROPHYLAXIS, P843

Lee S, 2003, AM J PREV MED, V24, P113, DOI 10.1016/S0749-3797(02)00582-2

Lengert N, 2015, BIOPHYS CHEM, V202, P21, DOI 10.1016/j.bpc.2015.03.009

Machida Ayako, 2012, Nihon Ronen Igakkai Zasshi, V49, P463

Morris G, 2016, MOL NEUROBIOL, V53, P2550, DOI 10.1007/s12035-015-9262-7

Morriss RK, 1998, J PSYCHOSOM RES, V45, P411, DOI 10.1016/S0022-3999(98)00022-1

RADLOFF L S, 1977, Applied Psychological Measurement, V1, P385, DOI 10.1177/014662167700100306

Rullier L, 2014, INT PSYCHOGERIATR, V26, P105, DOI 10.1017/S1041610213001579

Schulz R, 1999, JAMA-J AM MED ASSOC, V282, P2215, DOI 10.1001/jama.282.23.2215

Stalder T, 2014, PSYCHONEUROENDOCRINO, V47, P26, DOI 10.1016/j.psyneuen.2014.04.021

Tanaka M, 2012, PLOS ONE, V7, DOI 10.1371/journal.pone.0034774

Timmerman JG, 2015, EUR J ONCOL NURS, V19, P162, DOI 10.1016/j.ejon.2014.09.005

Whitley R, 2007, HUMAN HERPESVIRUSES: BIOLOGY, THERAPY, AND IMMUNOPROPHYLAXIS, P589

ZARIT SH, 1980, GERONTOLOGIST, V20, P649, DOI 10.1093/geront/20.6.649

NR 30

TC 16

Z9 16

U1 0

U2 14

PU ELSEVIER IRELAND LTD

PI CLARE

PA ELSEVIER HOUSE, BROOKVALE PLAZA, EAST PARK SHANNON, CO, CLARE, 00000,

IRELAND

SN 0167-4943

EI 1872-6976

J9 ARCH GERONTOL GERIAT

JI Arch. Gerontol. Geriatr.

PD SEP-OCT

PY 2016

VL 66

BP 42

EP 48

DI 10.1016/j.archger.2016.04.015

PG 7

WC Geriatrics & Gerontology

WE Science Citation Index Expanded (SCI-EXPANDED); Social Science Citation Index (SSCI)

SC Geriatrics & Gerontology

GA DT7CZ

UT WOS:000381646000006

PM 27214797

DA 2022-05-04

ER

PT J

AU Murcia, N

Cardin, O

Mohafid, A

Senkel, MP

AF Murcia, Nicolas

Cardin, Olivier

Mohafid, Abdelmoula

Senkel, Marie-Pascale

TI Health-Related Parameters for Evaluation Methodologies of Human

Operators in Industry: A Systematic Literature Review

SO SUSTAINABILITY

LA English

DT Article

DE ergonomics; industry; human factor; fatigue; long-term physical strain;

psychosocial risk; occupational disease

ID LOW-BACK-PAIN; MUSCULOSKELETAL SYMPTOMS; VIRTUAL-REALITY; MENTAL STRAIN;

HUMAN WORK; DESIGN; PERFORMANCE; ERGONOMICS; FRAMEWORK; ARCHITECTURE

AB Human factors have always been an important part of research in industry, but more recently the idea of sustainable development has attracted considerable interest for manufacturing companies and management practitioners. Incorporating human factors into a decision system is a difficult challenge for manufacturing companies because the data related to human factors are difficult to sense and integrate into the decision-making processes. Our objectives with this review are to propose an overview of the different methods to measure human factors, of the solutions to reduce the occupational strain for workers and of the technical solutions to integrate these measures and solutions into a complex industrial decision system. The Scopus database was systematically searched for works from 2014 to 2021 that describe some aspects of human factors in industry. We categorized these works into three different classes, representing the specificity of the studied human factor. This review aims to show the main differences between the approaches of short-term fatigue, long-term physical strain and psychosocial risks. Long-term physical strain is the subject that concentrates the most research efforts, mainly with physical and simulation techniques to highlight physical constraints at work. Short-term fatigue and psychosocial constraints have become a growing concern in industry due to new technologies that increase the requirements of cognitive activities of workers. Human factors are taking an important place in the sustainable development of industry, in order to ameliorate working conditions. However, vigilance is required because health-related data creation and exploitation are sensible for the integrity and privacy of workers.

C1 [Murcia, Nicolas] Airbus Grp, Airbus Atlantic, F-44550 Montoir De Bretagne, France.

[Murcia, Nicolas; Cardin, Olivier; Mohafid, Abdelmoula] Nantes Univ, LS2N UMR CNRS 6004 IUT Nantes, F-44470 Carquefou, France.

[Senkel, Marie-Pascale] Nantes Univ, LEMNA EA 4272 IUT St Nazaire, F-44600 St Nazaire, France.

RP Cardin, O (corresponding author), Nantes Univ, LS2N UMR CNRS 6004 IUT Nantes, F-44470 Carquefou, France.

EM nicolas.murcia@ls2n.fr; olivier.cardin@ls2n.fr;

abdelmoula.mohafid@ls2n.fr; marie-pascale.senkel@univ-nantes.fr

CR Ali Shan E., 2020, 2020 IEEE International Conference on Industry 4.0, Artificial Intelligence, and Communications Technology (IAICT). Proceedings, P69, DOI 10.1109/IAICT50021.2020.9172037

Angelopoulou Anastasia, 2020, Procedia Manufacturing, V42, P296, DOI 10.1016/j.promfg.2020.02.094

Ansari F., 2018, 2018 IEEE TECHN ENG, P1

Antao L, 2018, 2018 13TH APCA INTERNATIONAL CONFERENCE ON CONTROL AND SOFT COMPUTING (CONTROLO), P430, DOI 10.1109/CONTROLO.2018.8514549

Aslan M, 2021, BIOL METHODS PROTOC, V6, DOI 10.1093/biomethods/bpab014

Battini D, 2015, IFAC PAPERSONLINE, V48, P586, DOI 10.1016/j.ifacol.2015.06.145

Becker T, 2016, PROC CIRP, V57, P404, DOI 10.1016/j.procir.2016.11.070

Bernard B.P., 1997, CRITICAL REV EPIDEMI

Bertram P., 2019, PROCEDIA MANUF, V38, P983, DOI [10.1016/j.promfg.2020.01.182, DOI 10.1016/J.PROMFG.2020.01.182]

Bortolini M, 2020, COMPUT IND ENG, V139, DOI 10.1016/j.cie.2018.10.046

Brauner P, 2019, IEEE INTL CONF IND I, P861, DOI 10.1109/INDIN41052.2019.8972142

Brocal F, 2019, COMPLEXITY, V2019, DOI 10.1155/2019/2089763

Bruno F, 2020, INT J INTERACT DES M, V14, P805, DOI 10.1007/s12008-020-00664-x

Caporaso T., 2019, P 2019 2 WORKSH METR

Caputo F, 2019, PROD MANUF RES, V7, P195, DOI 10.1080/21693277.2019.1616631

Caputo F, 2018, PROCEDIA STRUCT INTE, V8, P297, DOI 10.1016/j.prostr.2017.12.031

Caputo F., 2018, INT C INT HUM SYST I, P450, DOI DOI 10.1007/978-3-319-73888-8_70

Caputo F, 2019, ADV INTELL SYST, V822, P170, DOI 10.1007/978-3-319-96077-7_18

Chiasson ME, 2012, INT J IND ERGONOM, V42, P478, DOI 10.1016/j.ergon.2012.07.003

Cierniak-Emerych A., 2020, IBIMA BUS REV, V2020, DOI 10.5171/2020.141027

Cimini C, 2020, J MANUF SYST, V54, P258, DOI 10.1016/j.jmsy.2020.01.002

Cohen Y, 2018, IFAC PAPERSONLINE, V51, P399, DOI 10.1016/j.ifacol.2018.08.327

Conforti I, 2020, 2020 IEEE INTERNATIONAL WORKSHOP ON METROLOGY FOR INDUSTRY 4.0 & IOT (METROIND4.0&IOT), P501, DOI 10.1109/MetroInd4.0IoT48571.2020.9138259

Conforti I, 2019, 2019 IEEE INTERNATIONAL WORKSHOP ON METROLOGY FOR INDUSTRY 4.0 AND INTERNET OF THINGS (METROIND4.0&IOT), P388, DOI 10.1109/METROI4.2019.8792843

Cordella F, 2019, 2019 IEEE INTERNATIONAL WORKSHOP ON METROLOGY FOR INDUSTRY 4.0 AND INTERNET OF THINGS (METROIND4.0&IOT), P405, DOI 10.1109/METROI4.2019.8792920

Costa D, 2019, 2019 IEEE INTERNATIONAL CONFERENCE ON INDUSTRIAL CYBER PHYSICAL SYSTEMS (ICPS 2019), P139, DOI 10.1109/ICPHYS.2019.8780138

Di Nardo M, 2020, PROD MANUF RES, V8, P20, DOI 10.1080/21693277.2020.1737592

DOBROWOLSKA M, 2020, SUSTAINABILITY-BASEL, V12, DOI DOI 10.3390/su12135302

Dombrowski U, 2014, PROC CIRP, V17, P100, DOI 10.1016/j.procir.2014.01.077

Edwards DJ, 2020, ENG CONSTR ARCHIT MA, V27, P2179, DOI 10.1108/ECAM-09-2019-0518

Fan ZJ, 2009, AM J IND MED, V52, P479, DOI 10.1002/ajim.20700

Fantini P, 2016, IEEE IND ELEC, P5711, DOI 10.1109/IECON.2016.7793579

Fantini P, 2020, COMPUT IND ENG, V139, DOI 10.1016/j.cie.2018.01.025

Gaham M, 2015, STUD COMPUT INTELL, V594, P315, DOI 10.1007/978-3-319-15159-5_29

Gasova M, 2017, PROCEDIA ENGINEER, V192, P219, DOI 10.1016/j.proeng.2017.06.038

Ghislieri C, 2018, FRONT PSYCHOL, V9, DOI 10.3389/fpsyg.2018.02365

Grazi L., 2018, P 2019 2 WORKSH METR, P400

Grazi L, 2020, IEEE T NEUR SYS REH, V28, P2276, DOI 10.1109/TNSRE.2020.3014408

Greco A, 2020, APPL SCI-BASEL, V10, DOI 10.3390/app10217758

Gualtieri L, 2021, ROBOT CIM-INT MANUF, V67, DOI 10.1016/j.rcim.2020.101998

Gualtieri L, 2020, SUSTAINABILITY-BASEL, V12, DOI 10.3390/su12093606

Hartvigsen J, 2004, OCCUP ENVIRON MED, V61

Havard V, 2019, PROD MANUF RES, V7, P472, DOI 10.1080/21693277.2019.1660283

Horvathova B, 2019, TRANSP RES PROC, V40, P1067, DOI 10.1016/j.trpro.2019.07.149

Jenderny S, 2018, 11TH ACM INTERNATIONAL CONFERENCE ON PERVASIVE TECHNOLOGIES RELATED TO ASSISTIVE ENVIRONMENTS (PETRA 2018), P319, DOI 10.1145/3197768.3201566

Kaasinen E, 2019, IFIP ADV INF COMM TE, P615, DOI 10.1007/978-3-030-30000-5_75

Kadir BA, 2020, INT J IND ERGONOM, V76, DOI 10.1016/j.ergon.2020.102936

Kadir BA, 2019, COMPUT IND ENG, V137, DOI 10.1016/j.cie.2019.106004

KARASEK RA, 1979, ADMIN SCI QUART, V24, P285, DOI 10.2307/2392498

Klippert Jurgen, 2020, PROCEDIA MANUFACTURI, V45, P55, DOI [10.1016/j.promfg.2020.04.062, DOI 10.1016/J.PROMFG.2020.04.062]

Koukoulaki T, 2014, APPL ERGON, V45, P198, DOI 10.1016/j.apergo.2013.07.018

Lamon E., 2018, P IEEE RAS 18 INT C, P1

Lanzotti A, 2018, INT J SAFETY SECURIT, V8, P132, DOI [10.2495/SAFE-V8-N1-132-138, DOI 10.2495/SAFE-V8-N1-132-138]

Laudante E., 2016, P LIBR ACT SYST DES

Laudante E, 2017, DES J, V20, pS2724, DOI 10.1080/14606925.2017.1352784

Longo F, 2020, APPL SCI-BASEL, V10, DOI 10.3390/app10124182

Longo F, 2019, INT J IND ERGONOM, V69, P29, DOI 10.1016/j.ergon.2018.09.002

Gonzalez-Munoz EL, 2015, PROCEDIA MANUF, V3, P4964, DOI 10.1016/j.promfg.2015.07.642

Mach S, 2019, ADV INTELL SYST, V825, P978, DOI 10.1007/978-3-319-96068-5_106

Maczewska A., 2020, ADV HUMAN FACTORS BU, V961, P378

Madonna M., 2019, WIT T BUILT ENV, DOI [10.2495/safe190021, DOI 10.2495/SAFE190021]

Mahmoudabadi M.Z., 2015, THESIS

Manghisi Vito M., 2020, Procedia Manufacturing, V42, P97, DOI 10.1016/j.promfg.2020.02.091

Mannhardt F, 2019, J AMB INTEL SMART EN, V11, P201, DOI 10.3233/AIS-190521

Mark Benedikt G., 2020, Procedia CIRP, V88, P98, DOI 10.1016/j.procir.2020.05.018

Mattsson S, 2020, COMPUT IND ENG, V139, DOI 10.1016/j.cie.2018.08.011

Meissner J, 2018, PROCEDIA MANUF, V24, P264, DOI 10.1016/j.promfg.2018.06.029

Mengoni M, 2018, PROCEDIA MANUF, V17, P476, DOI 10.1016/j.promfg.2018.10.072

Menolotto M, 2020, SENSORS-BASEL, V20, DOI 10.3390/s20195687

Merkel L, 2017, IN C IND ENG ENG MAN, P1189, DOI 10.1109/IEEM.2017.8290080

Muller SL, 2017, LECT NOTES COMPUT SC, V10286, P447, DOI 10.1007/978-3-319-58463-8_37

Murcia N., 2021, INT WORKSH SERV OR H, P274

Nayyar A., 2020, ADV SCI TECHNOLOGY

Nicoletti L., 2019, INT J SIMUL PROCESS, V14, P178, DOI [10.1504/IJSPM.2019.099912, DOI 10.1504/IJSPM.2019.099912]

Ojstersek R, 2020, APPL SCI-BASEL, V10, DOI 10.3390/app10207037

Otto A, 2017, COMPUT IND ENG, V111, P467, DOI 10.1016/j.cie.2017.04.011

Pacaux-Lemoine Marie-Pierre, 2018, 2018 IEEE Industrial Cyber-Physical Systems (ICPS). Proceedings, P615, DOI 10.1109/ICPHYS.2018.8390776

Pacaux-Lemoine MP, 2017, COMPUT IND ENG, V111, P581, DOI 10.1016/j.cie.2017.05.014

Page MJ, 2021, BMJ-BRIT MED J, V372, DOI 10.1136/bmj.n71

Panariello D, 2019, 2019 IEEE INTERNATIONAL WORKSHOP ON METROLOGY FOR INDUSTRY 4.0 AND INTERNET OF THINGS (METROIND4.0&IOT), P78, DOI 10.1109/METROI4.2019.8792847

Panariello D, 2021, INT J INTERACT DES M, V15, P121, DOI 10.1007/s12008-020-00737-x

Papetti Alessandra, 2020, Procedia CIRP, V91, P295, DOI 10.1016/j.procir.2020.02.179

Paredes-Astudillo Y.A., 2020, P 2020 IEEE INT C HU, P1

Paviglianiti A, 2020, 2020 IEEE INTERNATIONAL WORKSHOP ON METROLOGY FOR INDUSTRY 4.0 & IOT (METROIND4.0&IOT), P314, DOI 10.1109/MetroInd4.0IoT48571.2020.9138291

Peruzzini M, 2020, COMPUT IND ENG, V139, DOI 10.1016/j.cie.2018.12.047

Peruzzini M, 2019, DYNA-BILBAO, V94, P182, DOI 10.6036/8889

Peruzzini M, 2017, ADV ENG INFORM, V33, P330, DOI 10.1016/j.aei.2017.02.003

Phillips RO, 2015, TRANSPORT RES F-TRAF, V29, P48, DOI 10.1016/j.trf.2015.01.003

Pistolesi F, 2020, IEEE T IND INFORM, V16, P7199, DOI 10.1109/TII.2020.2992984

Pradani W. R., 2019, IOP Conference Series: Materials Science and Engineering, V528, DOI 10.1088/1757-899X/528/1/012011

Putnik GD, 2019, FME TRANS, V47, P663, DOI 10.5937/fmet1904663P

Ranavolo A, 2020, SENSORS-BASEL, V20, DOI 10.3390/s20205750

Reis J, 2017, IEEE IND ELEC, P8634, DOI 10.1109/IECON.2017.8217517

Richert A, 2016, IEEE WORK ADV ROBOT, P49, DOI 10.1109/ARSO.2016.7736255

Scafa M, 2019, PROC CIRP, V81, P162, DOI 10.1016/j.procir.2019.03.029

Schulte PA, 2020, ANN WORK EXPOS HEAL, V64, P786, DOI 10.1093/annweh/wxaa051

Sgarbossa, 2020, INT SERIES OPERATION, V289, P141

Sgarbossa F, 2020, ANNU REV CONTROL, V49, P295, DOI 10.1016/j.arcontrol.2020.04.007

Siafara LC, 2018, ELEKTROTECH INFORMAT, V135, P270, DOI 10.1007/s00502-018-0614-7

Simoes B, 2019, INT J ADV MANUF TECH, V105, P3965, DOI 10.1007/s00170-019-03939-0

Stern H, 2019, SUSTAINABILITY-BASEL, V11, DOI 10.3390/su11164508

Stern H, 2018, PROC CIRP, V72, P1233, DOI 10.1016/j.procir.2018.03.077

Stern H, 2017, PROCEDIA MANUF, V9, P151, DOI 10.1016/j.promfg.2017.04.030

de Miranda SSF, 2020, APPL SCI-BASEL, V10, DOI 10.3390/app10134442

Takala EP, 2010, SCAND J WORK ENV HEA, V36, P3, DOI 10.5271/sjweh.2876

TUTAK M, 2020, SUSTAINABILITY BASEL, DOI DOI 10.3390/SU12135390

Vernim S, 2017, IN C IND ENG ENG MAN, P1576, DOI 10.1109/IEEM.2017.8290158

Weckenborg C, 2019, IFAC PAPERSONLINE, V52, P1860, DOI 10.1016/j.ifacol.2019.11.473

Widanarko B, 2011, INT J IND ERGONOM, V41, P561, DOI 10.1016/j.ergon.2011.06.002

Widodo L., 2019, IOP C SERIES MAT SCI, V528

NR 110

TC 0

Z9 0

U1 2

U2 2

PU MDPI

PI BASEL

PA ST ALBAN-ANLAGE 66, CH-4052 BASEL, SWITZERLAND

EI 2071-1050

J9 SUSTAINABILITY-BASEL

JI Sustainability

PD DEC

PY 2021

VL 13

IS 23

AR 13387

DI 10.3390/su132313387

PG 20

WC Green & Sustainable Science & Technology; Environmental Sciences;

Environmental Studies

WE Science Citation Index Expanded (SCI-EXPANDED); Social Science Citation Index (SSCI)

SC Science & Technology - Other Topics; Environmental Sciences & Ecology

GA XV0JF

UT WOS:000734638800001

OA gold, Green Published

DA 2022-05-04

ER

PT J

AU Micklewright, D

Angus, C

Suddaby, J

Gibson, ASC

Sandercock, G

Chinnasamy, C

AF Micklewright, Dominic

Angus, Caroline

Suddaby, Jane

Gibson, Alan St Clair

Sandercock, Gavin

Chinnasamy, Camilla

TI Pacing Strategy in Schoolchildren Differs with Age and Cognitive

Development

SO MEDICINE AND SCIENCE IN SPORTS AND EXERCISE

LA English

DT Article

DE RUNNING; PERFORMANCE; EXERCISE; FATIGUE; TELEOANTICIPATION

ID PERIPHERAL PHYSIOLOGICAL SYSTEMS; CENTRAL NEURAL REGULATION; CYCLE TIME

TRIAL; PERCEIVED EXERTION; ENERGY-EXPENDITURE; SIMULATED COMPETITION;

ATHLETIC COMPETITION; EXERCISE; HUMANS; FATIGUE

AB MICKLEWRIGHT, D., C. ANGUS, J. SUDDABY, A. ST CLAIR GIBSON, G. SANDERCOCK, and C. CHINNASAMY. Pacing Strategy in Schoolchildren Differs with Age and Cognitive Development. Med. Sci. Sports Exerc., Vol. 44, No. 2, pp. 362-369, 2012. Purpose: The study's purpose was to examine differences in pacing strategy between schoolchildren of different age, gender, and stage of cognitive development. Methods: Schoolchildren (n = 106) from four age groups (5-6, 8-9, 11-12, and 14 yr) participated in this study. Each schoolchild completed four conservation tasks to evaluate his or her Piagetian stage of cognitive development. Each schoolchild then performed a best-effort running task on a 150-m running track that was video recorded to analyze pace at 5% increments. The length of the run was varied for each age group to ensure that all schoolchildren were running for approximately 4 min (5-6 yr = 450 m, 8-9 yr = 600 m, 11-12 yr = 750 m, and 14 yr = 900 m). Results: Differences in pacing strategy were found between schoolchildren of different age (P < 0.0001), gender (P < 0.0001), and cognitive development (P < 0.0001). Pacing differences were also found between age groups after controlling for cognitive development (P < 0.001), between cognitive abilities after controlling for age (P < 0.01), and between genders after controlling for both age (P < 0.0001) and cognitive ability (P < 0.0001). Conclusions: Younger schoolchildren with less advanced cognitive development exhibited a negative pacing strategy indicating an inability to anticipate exercise demand. Older schoolchildren at a more advanced stage of cognitive development exhibited a more conservative U-shaped pacing strategy characterized by faster running speeds during the first 15% and last 20% of the run. Anticipatory pacing strategy seems to be related to both the age and cognitive development of schoolchildren.

C1 [Micklewright, Dominic; Angus, Caroline; Suddaby, Jane; Sandercock, Gavin; Chinnasamy, Camilla] Univ Essex, Dept Biol Sci, Colchester CO4 3SQ, Essex, England.

[Gibson, Alan St Clair] Northumbria Univ, Sch Psychol & Sport Sci, Newcastle Upon Tyne NE1 8ST, Tyne & Wear, England.

RP Micklewright, D (corresponding author), Univ Essex, Dept Biol Sci, Wivenhoe Pk, Colchester CO4 3SQ, Essex, England.

EM dpmick@essex.ac.uk

RI Micklewright, Dominic/C-3307-2016

OI Micklewright, Dominic/0000-0002-7519-3252

FU University of Essex

FX This study was funded entirely by the University of Essex, and no other

sources of external funding were used.

CR Abbiss CR, 2008, SPORTS MED, V38, P239, DOI 10.2165/00007256-200838030-00004

ALBERTS JR, 1978, J COMP PHYSIOL PSYCH, V92, P231, DOI 10.1037/h0077459

Albertus Y, 2005, MED SCI SPORT EXER, V37, P461, DOI 10.1249/01.MSS.0000155700.72702.76

ANSHEL MH, 1995, AUST PSYCHOL, V30, P78, DOI 10.1080/00050069508258907

Ansley L, 2004, MED SCI SPORT EXER, V36, P1819, DOI 10.1249/01.MSS.0000142409.70181.9D

Ansley L, 2004, MED SCI SPORT EXER, V36, P309, DOI 10.1249/01.MSS.0000113474.31529.C6

Atkinson G, 2007, SPORTS MED, V37, P647, DOI 10.2165/00007256-200737080-00001

de Koning JJ, 2011, PLOS ONE, V6, DOI 10.1371/journal.pone.0015863

Elferink-Gemser M. T., 2008, J PHYS ED SPORT SCI, V4, P95

Faulkner J, 2008, PSYCHOPHYSIOLOGY, V45, P977, DOI 10.1111/j.1469-8986.2008.00712.x

Foster C, 2005, MED SCI SPORT EXER, V37, P670, DOI 10.1249/01.MSS.0000158183.64465.BF

Foster C, 2004, INT J SPORTS MED, V25, P198, DOI 10.1055/s-2003-45260

Foster C, 2003, MED SCI SPORT EXER, V35, P826, DOI 10.1249/01.MSS.0000065001.17658.68

Foster C, 2009, BRIT J SPORT MED, V43, P765, DOI 10.1136/bjsm.2008.054841

FOSTER C, 1994, SPORTS MED, V17, P77, DOI 10.2165/00007256-199417020-00001

Gass CL, 1999, FUNCT ECOL, V13, P483, DOI 10.1046/j.1365-2435.1999.00335.x

Gibson ASC, 2006, SPORTS MED, V36, P705

Gibson AS, 2004, BRIT J SPORT MED, V38, P797, DOI 10.1136/bjsm.2003.009852

Gibson AS, 2003, SPORTS MED, V33, P167

Gibson AS, 2001, SPORTS MED, V31, P637, DOI 10.2165/00007256-200131090-00001

Groslambert A, 2006, SPORTS MED, V36, P911, DOI 10.2165/00007256-200636110-00001

Houston AI, 1998, J AVIAN BIOL, V29, P395, DOI 10.2307/3677158

Hulleman M, 2007, MED SCI SPORT EXER, V39, P709, DOI 10.1249/mss.0b013e31802eff36

Jonker L, 2010, J SPORT SCI, V28, P901, DOI 10.1080/02640411003797157

Lambert EV, 2005, BRIT J SPORT MED, V39, P52, DOI 10.1136/bjsm.2003.011247

Mauger AR, 2009, MED SCI SPORT EXER, V41, P451, DOI 10.1249/MSS.0b013e3181854957

Micklewright D, 2010, BRIT J SPORT MED, V44, P952, DOI 10.1136/bjsm.2009.057315

Noakes TD, 2005, BRIT J SPORT MED, V39, P120, DOI 10.1136/bjsm.2003.010330

Noakes TD, 2004, BRIT J SPORT MED, V38, P511, DOI 10.1136/bjsm.2003.009860

PIAGET J, 1972, HUM DEV, V15, P1, DOI 10.1159/000271225

Piaget J., 1966, PSYCHOL INTELL, P131

Sims DW, 2006, J ANIM ECOL, V75, P176, DOI 10.1111/j.1365-2656.2005.01033.x

Swart J, 2009, BRIT J SPORT MED, V43, P775, DOI 10.1136/bjsm.2008.056036

Tatterson A J, 2000, J Sci Med Sport, V3, P186

Tucker R, 2009, BRIT J SPORT MED, V43, P392, DOI 10.1136/bjsm.2008.050799

Tucker R, 2006, J PHYSIOL-LONDON, V574, P905, DOI 10.1113/jphysiol.2005.101733

Ulmer H. V., 1986, PERCEPTION EXERTION, P317

Ulmer HV, 1996, EXPERIENTIA, V52, P416, DOI 10.1007/BF01919309

WELSH MC, 1988, DEV NEUROPSYCHOL, V4, P199, DOI 10.1080/87565648809540405

NR 39

TC 31

Z9 31

U1 1

U2 12

PU LIPPINCOTT WILLIAMS & WILKINS

PI PHILADELPHIA

PA TWO COMMERCE SQ, 2001 MARKET ST, PHILADELPHIA, PA 19103 USA

SN 0195-9131

EI 1530-0315

J9 MED SCI SPORT EXER

JI Med. Sci. Sports Exerc.

PD FEB

PY 2012

VL 44

IS 2

BP 362

EP 369

DI 10.1249/MSS.0b013e31822cc9ec

PG 8

WC Sport Sciences

WE Science Citation Index Expanded (SCI-EXPANDED); Social Science Citation Index (SSCI)

SC Sport Sciences

GA 879FY

UT WOS:000299316300024

PM 21796049

DA 2022-05-04

ER

PT J

AU Yin, Z

Zhang, JH

AF Yin, Zhong

Zhang, Jianhua

TI Task-generic mental fatigue recognition based on neurophysiological

signals and dynamical deep extreme learning machine

SO NEUROCOMPUTING

LA English

DT Article

DE Mental fatigue; Human-machine system; Electroencephalography; Extreme

learning machine; Deep learning

ID OPERATOR FUNCTIONAL-STATE; ARTIFICIAL NEURAL-NETWORK; COGNITIVE

PERFORMANCE; EEG; WORKLOAD; CLASSIFICATION; MODEL; ALERTNESS; FEATURES;

SYSTEM

AB The electroencephalography (EEG) based machine-learning model for mental fatigue recognition can evaluate the reliability of the human operator performance. The task-generic model is particularly important since the time cost for preparing the task-specific training EEG dataset is avoid. This study develops a novel mental fatigue classifier, dynamical deep extreme learning machine (DD-ELM), to adapt the variation of the EEG feature distributions across two mental tasks. Different from the static deep learning approaches, DD-ELM iteratively updates the shallow weights at multiple time steps during the testing stage. The proposed method incorporates the both of the merits from the deep network for EEG feature abstraction and the ELM autoencoder for fast weight recompuation. The feasibility of the DD-ELM is validated by investigating EEG datasets recorded under two paradigms of AutoCAMS human-machine tasks. The accuracy comparison indicates the new classifier significantly outperforms several state-of-the-art mental fatigue estimators. By examining the CPU time, the computational burden of the DD-ELM is also acceptable for high-dimensional EEG features. (C) 2018 Elsevier B.V. All rights reserved.

C1 [Yin, Zhong] Univ Shanghai Sci & Technol, Engn Res Ctr Opt Instrument & Syst, Minist Educ, Shanghai Key Lab Modern Opt Syst, Jungong Rd 516, Shanghai 200093, Peoples R China.

[Zhang, Jianhua] East China Univ Sci & Technol, Dept Automat, Shanghai 200237, Peoples R China.

RP Yin, Z (corresponding author), Univ Shanghai Sci & Technol, Engn Res Ctr Opt Instrument & Syst, Minist Educ, Shanghai Key Lab Modern Opt Syst, Jungong Rd 516, Shanghai 200093, Peoples R China.

EM yinzhong@usst.edu.cn

FU National Natural Science Foundation of ChinaNational Natural Science

Foundation of China (NSFC) [61703277]; Shanghai Sailing Program

[17YF1427000]

FX This work is sponsored by the National Natural Science Foundation of

China under Grant no. 61703277 and the Shanghai Sailing Program

(17YF1427000).

CR Baldwin CL, 2012, NEUROIMAGE, V59, P48, DOI 10.1016/j.neuroimage.2011.07.047

Belkin M, 2002, ADV NEUR IN, V14, P585

Charbonnier S, 2016, EXPERT SYST APPL, V52, P91, DOI 10.1016/j.eswa.2016.01.013

Christensen JC, 2012, NEUROIMAGE, V59, P57, DOI 10.1016/j.neuroimage.2011.07.091

GRANDJEAN E, 1988, FITTING TASK MAN

Grozdanovic M., 2018, INT J IND E IN PRESS

Hajinoroozi M, 2016, SIGNAL PROCESS-IMAGE, V47, P549, DOI 10.1016/j.image.2016.05.018

Heine T, 2017, APPL ERGON, V61, P31, DOI 10.1016/j.apergo.2016.12.015

Hinton GE, 2006, NEURAL COMPUT, V18, P1527, DOI 10.1162/neco.2006.18.7.1527

Hockey G. R. J., 2003, OPERATOR FUNCTIONAL

Huang GB, 2006, NEUROCOMPUTING, V70, P489, DOI 10.1016/j.neucom.2005.12.126

Huang GB, 2006, IEEE T NEURAL NETWOR, V17, P879, DOI 10.1109/TNN.2006.875977

Huang GB, 2012, IEEE T SYST MAN CY B, V42, P513, DOI 10.1109/TSMCB.2011.2168604

Huang GB, 2010, NEUROCOMPUTING, V74, P155, DOI 10.1016/j.neucom.2010.02.019

Ke YF, 2015, INT J PSYCHOPHYSIOL, V98, P157, DOI 10.1016/j.ijpsycho.2015.10.004

Kiymik MK, 2004, J NEUROSCI METH, V139, P231, DOI 10.1016/j.jneumeth.2004.04.027

Lal SKL, 2001, BIOL PSYCHOL, V55, P173, DOI 10.1016/S0301-0511(00)00085-5

LAL SKL, 2000, P 4 INT C FAT TRANSP

Liu JP, 2010, BIOMED SIGNAL PROCES, V5, P124, DOI 10.1016/j.bspc.2010.01.001

Lo JC, 2016, TRANSPORT RES F-TRAF, V43, P325, DOI 10.1016/j.trf.2016.09.006

Lu SY, 2017, CNS NEUROL DISORD-DR, V16, P23, DOI 10.2174/1871527315666161019153259

Makeig S, 1995, NEUROREPORT, V7, P213, DOI 10.1097/00001756-199512000-00051

MARKAND ON, 1990, J CLIN NEUROPHYSIOL, V7, P163, DOI 10.1097/00004691-199004000-00003

OKOGBAA OG, 1994, APPL ERGON, V25, P355, DOI 10.1016/0003-6870(94)90054-X

Papadelis C, 2007, CLIN NEUROPHYSIOL, V118, P1906, DOI 10.1016/j.clinph.2007.04.031

Parasuraman R, 1996, HUM FACTORS, V38, P665, DOI 10.1518/001872096778827279

Reinerman-Jones L, 2016, SAFETY SCI, V88, P97, DOI 10.1016/j.ssci.2016.05.002

Sasahara I, 2015, PHYSIOL BEHAV, V147, P238, DOI 10.1016/j.physbeh.2015.04.042

SHIGIHARA Y, 2013, NEUROL PSYCHIAT BRAI, V19, DOI [10.1016/j.npbr.2012.07.002, DOI 10.1016/J.NPBR.2012.07.002]

Simon M, 2011, CLIN NEUROPHYSIOL, V122, P1168, DOI 10.1016/j.clinph.2010.10.044

Smolders KCHJ, 2014, J ENVIRON PSYCHOL, V39, P77, DOI 10.1016/j.jenvp.2013.12.010

Suykens JAK, 1999, NEURAL PROCESS LETT, V9, P293, DOI 10.1023/A:1018628609742

Tang JX, 2016, IEEE T NEUR NET LEAR, V27, P809, DOI 10.1109/TNNLS.2015.2424995

Ting CH, 2010, IEEE T SYST MAN CY A, V40, P251, DOI 10.1109/TSMCA.2009.2035301

Vieira S, 2017, NEUROSCI BIOBEHAV R, V74, P58, DOI 10.1016/j.neubiorev.2017.01.002

Vuckovic A, 2002, MED ENG PHYS, V24, P349, DOI 10.1016/S1350-4533(02)00030-9

Yeo MVM, 2009, SAFETY SCI, V47, P115, DOI 10.1016/j.ssci.2008.01.007

Yildiz A, 2009, EXPERT SYST APPL, V36, P7390, DOI 10.1016/j.eswa.2008.09.003

Yin Z, 2017, NEUROCOMPUTING, V260, P349, DOI 10.1016/j.neucom.2017.05.002

Yin Z, 2017, BIOMED SIGNAL PROCES, V33, P30, DOI 10.1016/j.bspc.2016.11.013

Zhang JH, 2017, COMPUT METH PROG BIO, V144, P147, DOI 10.1016/j.cmpb.2017.03.016

Zhang JH, 2017, IEEE T HUM-MACH SYST, V47, P536, DOI 10.1109/THMS.2017.2700631

Zhang JH, 2015, IEEE T HUM-MACH SYST, V45, P200, DOI 10.1109/THMS.2014.2366914

Zhang YD, 2018, MULTIMED TOOLS APPL, V77, P22629, DOI 10.1007/s11042-017-5023-0

NR 44

TC 20

Z9 21

U1 7

U2 64

PU ELSEVIER SCIENCE BV

PI AMSTERDAM

PA PO BOX 211, 1000 AE AMSTERDAM, NETHERLANDS

SN 0925-2312

EI 1872-8286

J9 NEUROCOMPUTING

JI Neurocomputing

PD MAR 29

PY 2018

VL 283

BP 266

EP 281

DI 10.1016/j.neucom.2017.12.062

PG 16

WC Computer Science, Artificial Intelligence

WE Science Citation Index Expanded (SCI-EXPANDED)

SC Computer Science

GA FV9GT

UT WOS:000424896600024

DA 2022-05-04

ER

PT J

AU Arstein-Kerslake, A

Flynn, E

AF Arstein-Kerslake, Anna

Flynn, Eilionoir

TI The right to legal agency: domination, disability and the protections of

Article 12 of the Convention on the Rights of Persons with Disabilities

SO INTERNATIONAL JOURNAL OF LAW IN CONTEXT

LA English

DT Article

ID MENTAL-CAPACITY; AUTONOMY; SUPPORT

AB Article 12 of the Convention on the Rights of Persons with Disabilities has created a revolution in legal-capacity law reform. It protects the right to exercise legal agency for people with disabilities with more clarity than any prior human rights instrument. This paper explores what constitutes an exercise of legal agency and what exactly Article 12 protects. It proposes a definition of legal agency and applies it to the lived experience of cognitive disability. It also uses a republican theory of domination to argue that people with cognitive disabilities who are experiencing domination are forced to assert legal agency in even daily decision-making because of the high level of external regulation of their lives and the ever-present threat of others substituting their decision-making. It identifies Article 12 as a tool for protecting such exertions of legal agency and curtailing relationships of domination.

C1 [Arstein-Kerslake, Anna] Hallmark Disabil Res Initiat, Melbourne, Vic, Australia.

[Arstein-Kerslake, Anna] Melbourne Law Sch, Melbourne, Vic, Australia.

[Flynn, Eilionoir] NUI Galway, Ctr Disabil Law & Policy, Galway, Ireland.

RP Arstein-Kerslake, A (corresponding author), Hallmark Disabil Res Initiat, Melbourne, Vic, Australia.; Arstein-Kerslake, A (corresponding author), Melbourne Law Sch, Melbourne, Vic, Australia.

EM anna.arstein@unimelb.edu.au; eilionoir.flynn@nuigalway.ie

OI Flynn, Eilionoir/0000-0002-2157-3655; Arstein-Kerslake,

Anna/0000-0002-8411-9724

CR Anna Arstein-Kerslake, 2014, SCANDINAVIAN J DISAB, V18, P77

Anscombe G.E.M., 1957, INTENTION

Arstein-Kerslake A, 2016, INT J HUM RIGHTS, V20, P471, DOI 10.1080/13642987.2015.1107052

Bach M., 2010, NEW PARADIGM PROTECT

Brammer A, 2012, J ADULT PROT, V14, P297, DOI 10.1108/14668201211286822

Bratman M.E., 1999, FACES INTENTION SELE

BRATMAN ME, 1990, SYS DEV FDN, P15

Brosnan L, 2017, INT J LAW CONTEXT, V13, P58, DOI 10.1017/S1744552316000471

Chappell A.L., 2001, BRIT J LEARN DISABIL, V29, P45, DOI [10.1046/j.1468-3156.2001.00084.x, DOI 10.1046/J.1468-3156.2001.00084.X]

Charlton James., 1998, NOTHING US US DISABI

COHEN PR, 1990, ARTIF INTELL, V42, P213, DOI 10.1016/0004-3702(90)90055-5

De Bhailis C, 2017, INT J LAW CONTEXT, V13, P6, DOI 10.1017/S174455231600046X

De Wispelaere J, 2014, DISABIL SOC, V29, P402, DOI 10.1080/09687599.2013.823076

Dhanda A, 2007, SYRACUSE J INT LAW C, V34, P429

Edwards J. L. J., 1958, MOD LAW REV, V21, P375, DOI [10.1111/j.1468-2230.1958.tb00482.x, DOI 10.1111/J.1468-2230.1958.TB00482.X]

Flynn E, 2014, INT J LAW CONTEXT, V10, P81, DOI 10.1017/S1744552313000384

Gooding P, 2015, HUM RIGHTS LAW REV, V15, P45, DOI 10.1093/hrlr/ngu045

Griffith GM, 2013, CLIN PSYCHOL-SCI PR, V20, P469, DOI 10.1111/cpsp.12053

Kanter AS, 2012, ISR LAW REV, V45, P181, DOI 10.1017/S0021223712000015

Kayess R, 2008, HUM RIGHTS LAW REV, V8, P1, DOI 10.1093/hrlr/ngm044

Kittay EF, 2010, COGNITIVE DISABILITY

Kittay EvaFeder., 1999, LOVES LABOR ESSAYS W

Lehman Jeffrey, 2005, W ENCY AM LAW

Lo B, 1990, Law Med Health Care, V18, P193, DOI 10.1111/j.1748-720X.1990.tb00022.x

Lovett F, 2010, GEN THEORY DOMINANCE

Lucy Series, 2013, THESIS

McSherry B, 2012, J LAW MED, V20, P22

Minkowitz T., 2007, SYRACUSE J INT LAW C, V34, P405

Nicholson TRJ, 2008, BRIT MED J, V336, P322, DOI 10.1136/bmj.39457.485347.80

Nussbaum M.C., 2006, FRONTIERS JUSTICE DI

O'Shea Tom, 2015, J APPL PHILOS, V33

Office of the High Commissioner of Human Rights, 2005, AD HOC COMM DEL FOLL

Oliver M., 2004, IMPLEMENTING SOCIAL

Owen GS, 2009, INQUIRY, V52, P79, DOI 10.1080/00201740802661502

Pettit Philip., 1997, REPUBLICANISM THEORY

Quinn G., 2009, EUROPEAN YB DISABILI, V1

Rawls J., 2009, THEORY JUSTICE

Rehbinder M., 1970, STANFORD LAW REV, V23, P941

Series L, 2015, INT J LAW PSYCHIAT, V40, P80, DOI 10.1016/j.ijlp.2015.04.010

Shakespeare T, 1997, DISABIL SOC, V12, P293, DOI 10.1080/09687599727380

SHIELDS SA, 1982, SIGNS, V7, P769, DOI 10.1086/493921

Tajfel H., 2010, SOCIAL IDENTITY INTE

Taylor JamesStacey., 2005, PERSONAL AUTONOMY NE

Teubner G, 2000, SOC LEGAL STUD, V9, P399, DOI 10.1177/096466390000900305

Topor A, 2016, COMMUNITY MENT HLT J, V52, P731, DOI 10.1007/s10597-015-9928-7

NR 45

TC 26

Z9 26

U1 0

U2 9

PU CAMBRIDGE UNIV PRESS

PI CAMBRIDGE

PA EDINBURGH BLDG, SHAFTESBURY RD, CB2 8RU CAMBRIDGE, ENGLAND

SN 1744-5523

EI 1744-5531

J9 INT J LAW CONTEXT

JI Int. J. Law Context

PD MAR

PY 2017

VL 13

IS 1

SI SI

BP 22

EP 38

DI 10.1017/S1744552316000458

PG 17

WC Law

WE Emerging Sources Citation Index (ESCI)

SC Government & Law

GA EM7CF

UT WOS:000395468400003

DA 2022-05-04

ER

PT J

AU Schmit, C

Brisswalter, J

AF Schmit, Cyril

Brisswalter, Jeanick

TI Executive functioning during prolonged exercise: a fatigue-based

neurocognitive perspective

SO INTERNATIONAL REVIEW OF SPORT AND EXERCISE PSYCHOLOGY

LA English

DT Article

DE Exhaustion; self-regulation; self-control; cognitive performance;

lucidity

ID ANTERIOR CINGULATE CORTEX; COGNITIVE TASK-PERFORMANCE; SELF-CONTROL;

HUMAN BRAIN; INCREMENTAL EXERCISE; PREFRONTAL CORTEX; REACTION-TIME;

INDIVIDUAL-DIFFERENCES; PERCEIVED EXERTION; BLOOD LACTATE

AB Despite emotional, technical and endurance implications for athletes' performance, a consensus has yet to be reached to explain the impairment of executive functioning during exercise. In particular, recent research challenges the original assumption of a linear dose-response effect of exercise intensity on cerebral physiology and executive functioning. We propose a fatigue-based neurocognitive perspective of executive functioning during prolonged exercise, suggesting that top-down (cognitive and physical efforts) and bottom-up processes (body sensations) act in parallel of arousing mechanisms to determine cognitive outcomes. In this perspective, executive functioning during prolonged exercise would be dynamical rather than steady (i.e. positively then negatively impacted by exercise) and would be to analyse in regards of exercise termination rather than of exercise intensity.

C1 [Schmit, Cyril] Innov Training, Endurance Performance Lab, Aubagne, France.

[Schmit, Cyril] French Natl Inst Sport Expertise & Performance IN, Lab Sport Expertise & Performance, EA7370, Res Dept, Paris, France.

[Brisswalter, Jeanick] Univ Cote Azur, LAMHESS, Lab LAMHESS, EA6312, Nice, France.

RP Schmit, C (corresponding author), Innov Training, Endurance Performance Lab, Aubagne, France.; Schmit, C (corresponding author), French Natl Inst Sport Expertise & Performance IN, Lab Sport Expertise & Performance, EA7370, Res Dept, Paris, France.

EM cyril.schmit@gmail.com

CR Abbiss CR, 2015, SPORTS MED, V45, P1235, DOI 10.1007/s40279-015-0344-5

ABERNETHY B, 1988, J HUM MOVEMENT STUD, V14, P101

Aitchison C, 2013, PERCEPT MOTOR SKILL, V117, P11, DOI 10.2466/06.30.PMS.117x11z3

Amann M, 2010, J APPL PHYSIOL, V109, P966, DOI 10.1152/japplphysiol.00462.2010

Ando S, 2005, EUR J APPL PHYSIOL, V94, P461, DOI 10.1007/s00421-005-1330-7

Ando S, 2011, EUR J APPL PHYSIOL, V111, P1973, DOI 10.1007/s00421-011-1827-1

Badin OO, 2016, INT J SPORT PHYSIOL, V11, P1100, DOI 10.1123/ijspp.2015-0710

Balague N, 2012, PSYCHOL SPORT EXERC, V13, P591, DOI 10.1016/j.psychsport.2012.02.013

Bandelow S, 2010, SCAND J MED SCI SPOR, V20, P148, DOI 10.1111/j.1600-0838.2010.01220.x

Bartholomew CJ, 1999, INT J AVIAT PSYCHOL, V9, P351, DOI 10.1207/s15327108ijap0904_3

Boksem MAS, 2008, BRAIN RES REV, V59, P125, DOI 10.1016/j.brainresrev.2008.07.001

Bonaz B, 2003, J Physiol Pharmacol, V54 Suppl 4, P27

Borg G., 1998, BORGS PERCEIVED EXER

Botvinick MM, 2004, TRENDS COGN SCI, V8, P539, DOI 10.1016/j.tics.2004.10.003

Bray SR, 2015, J SPORT SCI, V33, P534, DOI 10.1080/02640414.2014.949830

Bray SR, 2012, BIOL PSYCHOL, V89, P195, DOI 10.1016/j.biopsycho.2011.10.008

Brisswalter J, 2002, SPORTS MED, V32, P555, DOI 10.2165/00007256-200232090-00002

Buhle JT, 2014, CEREB CORTEX, V24, P2981, DOI 10.1093/cercor/bht154

Bullock T, 2014, FRONT PSYCHOL, V5, DOI 10.3389/fpsyg.2014.01290

Carter CS, 1998, SCIENCE, V280, P747, DOI 10.1126/science.280.5364.747

Chang YK, 2012, BRAIN RES, V1453, P87, DOI 10.1016/j.brainres.2012.02.068

CHMURA J, 1994, INT J SPORTS MED, V15, P172, DOI 10.1055/s-2007-1021042

Chmura J, 2010, INT J PSYCHOPHYSIOL, V75, P287, DOI 10.1016/j.ijpsycho.2009.12.011

Cohen N, 2016, NEUROIMAGE, V125, P1022, DOI 10.1016/j.neuroimage.2015.10.069

Davranche K, 2009, BRAIN COGNITION, V69, P565, DOI 10.1016/j.bandc.2008.12.001

de Morree HM, 2012, PSYCHOPHYSIOLOGY, V49, P1242, DOI 10.1111/j.1469-8986.2012.01399.x

De Pauw K, 2015, J APPL PHYSIOL, V118, P776, DOI 10.1152/japplphysiol.01050.2014

de Wit S, 2009, J NEUROSCI, V29, P11330, DOI 10.1523/JNEUROSCI.1639-09.2009

Dehaene S, 2001, COGNITION, V79, P1, DOI 10.1016/S0010-0277(00)00123-2

Del Giorno JM, 2010, J SPORT EXERCISE PSY, V32, P312, DOI 10.1123/jsep.32.3.312

DETTMERS C, 1995, J NEUROPHYSIOL, V74, P802, DOI 10.1152/jn.1995.74.2.802

Dettmers C, 1996, J APPL PHYSIOL, V81, P596, DOI 10.1152/jappl.1996.81.2.596

Dietrich A, 2004, BRAIN COGNITION, V55, P516, DOI 10.1016/j.bandc.2004.03.002

Dietrich A, 2003, CONSCIOUS COGN, V12, P231, DOI 10.1016/S1053-8100(02)00046-6

Dietrich A, 2011, NEUROSCI BIOBEHAV R, V35, P1305, DOI 10.1016/j.neubiorev.2011.02.001

Dishman RK, 2006, OBESITY, V14, P345, DOI 10.1038/oby.2006.46

Edwards AM, 2013, SPORTS MED, V43, P1057, DOI 10.1007/s40279-013-0091-4

Englert C, 2012, J SPORT EXERCISE PSY, V34, P580, DOI 10.1123/jsep.34.5.580

ERIKSEN BA, 1974, PERCEPT PSYCHOPHYS, V16, P143, DOI 10.3758/BF03203267

Evans DR, 2016, PERS SOC PSYCHOL REV, V20, P291, DOI 10.1177/1088868315597841

Evans JSBT, 2008, ANNU REV PSYCHOL, V59, P255, DOI 10.1146/annurev.psych.59.103006.093629

Everitt BJ, 1997, ANNU REV PSYCHOL, V48, P649, DOI 10.1146/annurev.psych.48.1.649

Forstmann BU, 2008, J COGNITIVE NEUROSCI, V20, P1854, DOI 10.1162/jocn.2008.20122

Fox MD, 2005, P NATL ACAD SCI USA, V102, P9673, DOI 10.1073/pnas.0504136102

Fuster JM, 1997, TRENDS NEUROSCI, V20, P451, DOI 10.1016/S0166-2236(97)01128-4

Gandevia SC, 2001, PHYSIOL REV, V81, P1725, DOI 10.1152/physrev.2001.81.4.1725

Gaoua N, 2011, INT J HYPERTHER, V27, P1, DOI [10.3109/02656736.2010.516305, 10.3109/02656736.2010.519371]

Grego F, 2004, NEUROSCI LETT, V364, P76, DOI 10.1016/j.neulet.2004.03.085

Grego F, 2005, INT J SPORTS MED, V26, P27, DOI 10.1055/s-2004-817915

Hilty L, 2011, HUM BRAIN MAPP, V32, P2151, DOI 10.1002/hbm.21177

Hocking C, 2001, COMP BIOCHEM PHYS A, V128, P719, DOI 10.1016/S1095-6433(01)00278-1

Hofmann W, 2012, TRENDS COGN SCI, V16, P174, DOI 10.1016/j.tics.2012.01.006

Houben K, 2011, APPETITE, V56, P345, DOI 10.1016/j.appet.2010.12.017

Huang H J, 2001, Pediatr Phys Ther, V13, P133, DOI 10.1097/00001577-200110000-00005

HUMPHREYS MS, 1984, PSYCHOL REV, V91, P153, DOI 10.1037/0033-295X.91.2.153

JENKINS IH, 1994, J NEUROSCI, V14, P3775

Jouanin JC, 2009, HUM BRAIN MAPP, V30, P675, DOI 10.1002/hbm.20534

Joyner MJ, 2008, J PHYSIOL-LONDON, V586, P35, DOI 10.1113/jphysiol.2007.143834

Kahneman D., 1973, ATTENTION EFFORT

Kelso J.S., 1995, DYNAMIC PATTERNS SEL

Kerns JG, 2004, SCIENCE, V303, P1023, DOI 10.1126/science.1089910

Kim S, 2011, BIOL PSYCHIAT, V69, P1140, DOI 10.1016/j.biopsych.2010.07.005

Labbe D, 2011, FOOD QUAL PREFER, V22, P92, DOI 10.1016/j.foodqual.2010.08.002

Labelle V, 2013, BRAIN COGNITION, V81, P10, DOI 10.1016/j.bandc.2012.10.001

Lambourne K, 2010, MED SCI SPORT EXER, V42, P1396, DOI 10.1249/MSS.0b013e3181cbee11

Lambourne K, 2010, BRAIN RES, V1341, P12, DOI 10.1016/j.brainres.2010.03.091

Leh SE, 2010, NEUROPSYCHOPHARMACOL, V35, P70, DOI 10.1038/npp.2009.88

Lieberman S., 2007, J AM COLL NUTR, V26, P5495

Lorist MM, 2005, COGNITIVE BRAIN RES, V24, P199, DOI 10.1016/j.cogbrainres.2005.01.018

Lorist MM, 2002, J PHYSIOL-LONDON, V545, P313, DOI 10.1113/jphysiol.2002.027938

Lucas SJE, 2012, EXP GERONTOL, V47, P541, DOI 10.1016/j.exger.2011.12.002

Marcora SM, 2009, J APPL PHYSIOL, V106, P857, DOI 10.1152/japplphysiol.91324.2008

McCarron J., 2013, BRIT J SPORT MED, V47, pe4

McEwan D, 2013, J SPORT EXERCISE PSY, V35, P239, DOI 10.1123/jsep.35.3.239

McMorris T, 2008, PHARMACOL BIOCHEM BE, V89, P106, DOI 10.1016/j.pbb.2007.11.007

McMorris T, 2000, INT J SPORT PSYCHOL, V31, P66

McMorris T., 2016, EXERCISE COGNITION I, P65, DOI [10.1016/B978-0-12-800778-500004-9, DOI 10.1016/B978-0-12-800778-5.00004-9]

McMorris T E, 2009, EXERCISE COGNITIVE F

McMorris T, 2012, BRAIN COGNITION, V80, P338, DOI 10.1016/j.bandc.2012.09.001

McMorris T, 2011, PHYSIOL BEHAV, V102, P421, DOI 10.1016/j.physbeh.2010.12.007

McMorris T, 2009, INT J PSYCHOPHYSIOL, V73, P334, DOI 10.1016/j.ijpsycho.2009.05.004

Meeusen R, 2006, SPORTS MED, V36, P881, DOI 10.2165/00007256-200636100-00006

Meeusen R, 2016, J APPL PHYSIOL, V120, P467, DOI 10.1152/japplphysiol.00967.2015

Meyniel F, 2014, PLOS COMPUT BIOL, V10, DOI 10.1371/journal.pcbi.1003584

Meyniel F, 2013, P NATL ACAD SCI USA, V110, P2641, DOI 10.1073/pnas.1211925110

Millet GY, 2011, SPORTS MED, V41, P489, DOI 10.2165/11588760-000000000-00000

Miyake A, 2000, COGNITIVE PSYCHOL, V41, P49, DOI 10.1006/cogp.1999.0734

Morrison AB, 2011, PSYCHON B REV, V18, P46, DOI 10.3758/s13423-010-0034-0

Muraven M, 2000, PSYCHOL BULL, V126, P247, DOI 10.1037/0033-2909.126.2.247

Muzik O, 2016, HUM BRAIN MAPP, V37, P3188, DOI 10.1002/hbm.23233

Noble B. J., 1996, PERCEIVED EXERTION

Nowak A., 1998, DYNAMICAL SOCIAL PSY

Ochsner KN, 2008, CURR DIR PSYCHOL SCI, V17, P153, DOI 10.1111/j.1467-8721.2008.00566.x

Ochsner KN, 2005, TRENDS COGN SCI, V9, P242, DOI 10.1016/j.tics.2005.03.010

Ogoh S, 2014, PHYSIOL REP, V2, DOI 10.14814/phy2.12163

Olson RL, 2016, NEUROIMAGE, V131, P171, DOI 10.1016/j.neuroimage.2015.10.011

Pageaux B, 2016, EUR J SPORT SCI, V16, P885, DOI 10.1080/17461391.2016.1188992

Paus T, 2001, NAT REV NEUROSCI, V2, P417, DOI 10.1038/35077500

Pesce C, 2012, J SPORT EXERCISE PSY, V34, P766, DOI 10.1123/jsep.34.6.766

Peyron R, 2000, NEUROPHYSIOL CLIN, V30, P263, DOI 10.1016/S0987-7053(00)00227-6

Piepmeier AT, 2015, J SPORT HEALTH SCI, V4, P14, DOI 10.1016/j.jshs.2014.11.001

Poldrack RA, 2003, NEUROPSYCHOLOGIA, V41, P245, DOI 10.1016/S0028-3932(02)00157-4

Pontifex MB, 2007, CLIN NEUROPHYSIOL, V118, P570, DOI 10.1016/j.clinph.2006.09.029

Ramos BP, 2007, PHARMACOL THERAPEUT, V113, P523, DOI 10.1016/j.pharmthera.2006.11.006

Rattray B, 2015, FRONT PHYSIOL, V6, DOI 10.3389/fphys.2015.00079

Robbins TW, 2007, PSYCHOPHARMACOLOGY, V191, P433, DOI 10.1007/s00213-006-0528-7

Romeas T, 2016, PSYCHOL SPORT EXERC, V22, P1, DOI 10.1016/j.psychsport.2015.06.002

Rooks CR, 2010, PROG NEUROBIOL, V92, P134, DOI 10.1016/j.pneurobio.2010.06.002

ROTH RH, 1988, ANN NY ACAD SCI, V537, P138, DOI 10.1111/j.1749-6632.1988.tb42102.x

SANDERS AF, 1983, ACTA PSYCHOL, V53, P61, DOI 10.1016/0001-6918(83)90016-1

Schmidt L, 2012, PLOS BIOL, V10, DOI 10.1371/journal.pbio.1001266

Schmidt L, 2009, J NEUROSCI, V29, P9450, DOI 10.1523/JNEUROSCI.1951-09.2009

Schmit C, 2015, NEUROPSYCHOLOGIA, V68, P71, DOI 10.1016/j.neuropsychologia.2015.01.006

Shenhav A, 2013, NEURON, V79, P217, DOI 10.1016/j.neuron.2013.07.007

Singh AM, 2015, J MOTOR BEHAV, V47, P328, DOI 10.1080/00222895.2014.983450

Smith MR, 2015, MED SCI SPORT EXER, V47, P1682, DOI 10.1249/MSS.0000000000000592

Sridharan D, 2008, P NATL ACAD SCI USA, V105, P12569, DOI 10.1073/pnas.0800005105

Sun G, 2013, PLOS ONE, V8, DOI 10.1371/journal.pone.0061157

Swart J, 2012, BRIT J SPORT MED, V46, P42, DOI 10.1136/bjsports-2011-090337

Tanaka M, 2012, NEUROSCI BIOBEHAV R, V36, P727, DOI 10.1016/j.neubiorev.2011.10.004

Teh MM, 2010, NEUROIMAGE, V53, P584, DOI 10.1016/j.neuroimage.2010.06.033

Tenenbaum G, 2008, PSYCHOL SPORT EXERC, V9, P704, DOI 10.1016/j.psychsport.2007.09.002

Tomporowski PD, 2003, ACTA PSYCHOL, V112, P297, DOI 10.1016/S0001-6918(02)00134-8

van Duinen H, 2007, NEUROIMAGE, V35, P1438, DOI 10.1016/j.neuroimage.2007.02.008

Van Orden GC, 2003, J EXP PSYCHOL GEN, V132, P331, DOI 10.1037/0096-3445.132.3.331

Vealey R.S., 2007, HDB SPORT PSYCHOL, P285

Vestberg T, 2012, PLOS ONE, V7, DOI 10.1371/journal.pone.0034731

von Leupoldt A, 2008, AM J RESP CRIT CARE, V177, P1026, DOI 10.1164/rccm.200712-1821OC

Walton ME, 2006, NEURAL NETWORKS, V19, P1302, DOI 10.1016/j.neunet.2006.03.005

Walton ME, 2003, J NEUROSCI, V23, P6475

Williamson JW, 2006, EXP PHYSIOL, V91, P51, DOI 10.1113/expphysiol.2005.032037

Wills TA, 2002, J CONSULT CLIN PSYCH, V70, P986, DOI 10.1037//0022-006X.70.4.986

Yerkes RM, 1908, J COMP NEUROL PSYCHO, V18, P459, DOI 10.1002/cne.920180503

Zwierko T., 2014, CENT EUR J SPORT SCI, V7, P99

NR 134

TC 22

Z9 23

U1 1

U2 8

PU ROUTLEDGE JOURNALS, TAYLOR & FRANCIS LTD

PI ABINGDON

PA 2-4 PARK SQUARE, MILTON PARK, ABINGDON OX14 4RN, OXON, ENGLAND

SN 1750-984X

EI 1750-9858

J9 INT REV SPORT EXER P

JI Int. Rev. Sport Exerc. Psychol.

PD JAN 1

PY 2020

VL 13

IS 1

BP 21

EP 39

DI 10.1080/1750984X.2018.1483527

PG 19

WC Hospitality, Leisure, Sport & Tourism; Psychology, Applied

WE Social Science Citation Index (SSCI)

SC Social Sciences - Other Topics; Psychology

GA ND3AD

UT WOS:000561775200002

DA 2022-05-04

ER

PT J

AU Goold, I

Maslen, H

AF Goold, Imogen

Maslen, Hannah

TI Must the Surgeon Take the Pill? Negligence Duty in the Context of

Cognitive Enhancement

SO MODERN LAW REVIEW

LA English

DT Article

DE Negligence; omissions; cognitive enhancement; medical; modafinil;

surgeon

ID HEALTHY-VOLUNTEERS; PRESCRIPTION STIMULANTS; MODAFINIL; PERFORMANCE;

SLEEP; FATIGUE; COUNTERMEASURES; WAKEFULNESS; STUDENTS; MIDDLE

AB Recently, attention has turned to the possibility of enhancing human cognitive abilities via pharmacological interventions. Known as cognitive enhancers', these drugs can alter human mental capacities, and in some cases can effect significant improvements. One prime example is modafinil, a drug used to treat narcolepsy, which can help combat decreases in wakefulness and cognitive capacity that arise due to fatigue in otherwise healthy individuals. In this paper, we respond to calls in the philosophical and ethical literature that surgeons and other medical professionals should be morally obliged to take cognitively enhancing drugs. We examine whether surgeons who make fatigue-related errors during patient care might be considered legally obliged to enhance themselves. We focus on liability for a failure to medicate, and conclude that it is highly unlikely that surgeons will be legally obliged to address their fatigue through the use of cognitive enhancing drugs.

C1 [Goold, Imogen] Univ Oxford, Fac Law, Oxford OX1 2JD, England.

[Goold, Imogen] Univ Oxford St Annes Coll, Oxford OX2 6HS, England.

[Maslen, Hannah] Univ Oxford, Uehiro Ctr Pract Eth, Oxford OX1 2JD, England.

[Maslen, Hannah] New Coll, Oxford, England.

RP Goold, I (corresponding author), Univ Oxford, Fac Law, Oxford OX1 2JD, England.

OI Goold, Imogen/0000-0001-7622-0999

CR Agency for Healthcare Research and Quality, 2001, AHRQ PUBLICATION 01

Baranski JV, 2002, MIL PSYCHOL, V14, P23, DOI 10.1207/S15327876MP1401_02

Bostrom N, 2009, SCI ENG ETHICS, V15, P311, DOI 10.1007/s11948-009-9142-5

Caldwell J. A., 2005, DEXTROAMPHETAMINE AN

Caldwell JA, 2005, AVIAT SPACE ENVIR MD, V76, pC39

Chandler JA, 2013, NEUROETHICS-NETH, V6, P249, DOI 10.1007/s12152-011-9109-5

Finke K, 2010, PSYCHOPHARMACOLOGY, V210, P317, DOI 10.1007/s00213-010-1823-x

Gaba DM, 2002, NEW ENGL J MED, V347, P1249, DOI 10.1056/NEJMsa020846

Goodman R, 2010, KENNEDY INST ETHIC J, V20, P145

Grady S, 2010, NEUROPSYCHOPHARMACOL, V35, P1910, DOI 10.1038/npp.2010.63

Great Ormond Street Hospital, CLINICAL GUIDELINES

Hart CL, 2006, NEUROPSYCHOPHARMACOL, V31, P1526, DOI 10.1038/sj.npp.1300991

Jackson E., 2010, MEDICAL LAW TEXT CAS, P103

Kramer Milton, 2010, Front Neurol, V1, P128, DOI 10.3389/fneur.2010.00128

Landrigan CP, 2004, NEW ENGL J MED, V351, P1838, DOI 10.1056/NEJMoa041406

Lynch G, 2011, PHARMACOL BIOCHEM BE, V99, P116, DOI 10.1016/j.pbb.2010.12.024

Maher B, 2008, NATURE, V452, P674, DOI 10.1038/452674a

Maslen H., OBLIGING SURGEONS TO

McCabe SE, 2005, ADDICTION, V100, P96, DOI 10.1111/j.1360-0443.2005.00944.x

McCabe SE, 2004, SUBST USE MISUSE, V39, P1095, DOI 10.1081/JA-120038031

Muller U, 2013, NEUROPHARMACOLOGY, V64, P490, DOI 10.1016/j.neuropharm.2012.07.009

Mulheron R., 2010, CAMB LAW J, V69, P609

Mulheron R., 2010, CAMBRIDGE LAW JOURNA, V69, P620

National Cancer Institute, 2011, NATIONAL CANCER INST

Randall DC, 2004, PHARMACOL BIOCHEM BE, V77, P547, DOI 10.1016/j.pbb.2003.12.016

Rose SH, 2009, MAYO CLIN PROC, V84, P955, DOI 10.1016/S0025-6196(11)60665-4

Savulescu J, 2006, ANN NY ACAD SCI, V1093, P321, DOI 10.1196/annals.1382.021

Singh I, 2005, AM J BIOETHICS, V5, P34, DOI 10.1080/15265160590945129

Sugden C, 2012, ANN SURG, V255, P222, DOI 10.1097/SLA.0b013e3182306c99

The Academy of Medical Sciences, 2012, REPORT FROM A JOINT, P38

The Royal College of Surgeons of England, 2008, GOOD SURGICAL PRACTI, P39

Thomas RJ, 2006, SLEEP, V29, P1471, DOI 10.1093/sleep/29.11.1471

Turner DC, 2003, PSYCHOPHARMACOLOGY, V165, P260, DOI 10.1007/s00213-002-1250-8

Vastag B, 2004, JAMA-J AM MED ASSOC, V291, P167, DOI 10.1001/jama.291.2.167

Warren O. J., 2009, JOURNAL OF SURGICAL, V152, P171

Warren OJ, 2009, J SURG RES, V152, P167, DOI 10.1016/j.jss.2007.12.761

Winder-Rhodes SE, 2010, J PSYCHOPHARMACOL, V24, P1649, DOI 10.1177/0269881109105899

Wong YN, 1998, J CLIN PHARMACOL, V38, P276, DOI 10.1002/j.1552-4604.1998.tb04425.x

NR 38

TC 14

Z9 14

U1 0

U2 15

PU WILEY-BLACKWELL

PI HOBOKEN

PA 111 RIVER ST, HOBOKEN 07030-5774, NJ USA

SN 0026-7961

EI 1468-2230

J9 MOD LAW REV

JI Mod. Law Rev.

PD JAN

PY 2014

VL 77

IS 1

BP 60

EP 86

DI 10.1111/1468-2230.12056

PG 27

WC Law

WE Social Science Citation Index (SSCI)

SC Government & Law

GA 282KH

UT WOS:000329169600003

OA Green Submitted

DA 2022-05-04

ER

PT J

AU Cheuvront, SN

Carter, R

Kolka, MA

Lieberman, HR

Kellogg, MD

Sawka, MN

AF Cheuvront, SN

Carter, R

Kolka, MA

Lieberman, HR

Kellogg, MD

Sawka, MN

TI Branched-chain amino acid supplementation and human performance when

hypohydrated in the heat

SO JOURNAL OF APPLIED PHYSIOLOGY

LA English

DT Article

DE dehydration; central fatigue; serotonin; prolactin; mood

ID EXERCISE-INDUCED CHANGE; PLASMA-FREE TRYPTOPHAN; PROLONGED EXERCISE;

CARBOHYDRATE SUPPLEMENTATION; COGNITIVE PERFORMANCE; PERCEIVED EXERTION;

SUSTAINED EXERCISE; VISUAL VIGILANCE; FATIGUE; STRESS

AB The serotonin system may contribute to reduced human performance when hypohydrated in the heat. This study determined whether branched-chain amino acid (BCAA) supplementation could sustain exercise and cognitive performance in the heat (40degreesC dry bulb, 20% relative humidity) when hypohydrated by 4% of body mass. Seven heat-accliniated men completed two experimental trials, each consisting of one preparation and one test day. On day 1, a low-carbohydrate diet was eaten and subjects performed exhaustive cycling (morning) and treadmill exercise in the heat (afternoon) to lower muscle glycogen and achieve the desired hypohydration level. On day 2, subjects consumed an isocaloric BCAA and carbohydrate (BC) or carbohydrate-only drink during exercise. Experimental trials included 60 min of cycle ergometry (50% peak oxygen uptake) followed by a 30-min time trial in the heat. A cognitive test battery was completed before and after exercise, and blood samples were taken. BC produced a 2.5-fold increase (P<0.05) in plasma BCAA and lowered (P<0.05) the ratios of total tryptophan to BCAA and large neutral amino acid. Blood prolactin, glucose, lactate, and osmolality were not different between trials but increased over time. Cardiovascular and thermoregulatory data were also similar between trials. BC did not alter time-trial performance, cognitive performance, mood, perceived exertion, or perceived thermal comfort. We conclude that BCAA does not alter exercise or cognitive performance in the heat when subjects are hypohydrated.

C1 USA, Environm Med Res Inst, Thermal & Mt Med Div, Natick, MA 01760 USA.

Childrens Hosp, Dept Lab Med, Boston, MA 02115 USA.

RP Cheuvront, SN (corresponding author), USA, Environm Med Res Inst, Thermal & Mt Med Div, Kansas St, Natick, MA 01760 USA.

EM samuel.cheuvront@na.amedd.army.mil

RI Sawka, Michael/R-7222-2019

OI Kellogg, Mark/0000-0003-1868-2153

CR BADDELEY AD, 1968, PSYCHON SCI, V10, P341

Blomstrand E, 1997, ACTA PHYSIOL SCAND, V159, P41, DOI 10.1046/j.1365-201X.1997.547327000.x

BLOMSTRAND E, 1992, ACTA PHYSIOL SCAND, V146, P293, DOI 10.1111/j.1748-1716.1992.tb09422.x

BLOMSTRAND E, 1991, EUR J APPL PHYSIOL, V63, P83, DOI 10.1007/BF00235174

BLOMSTRAND E, 1995, ACTA PHYSIOL SCAND, V153, P87, DOI 10.1111/j.1748-1716.1995.tb09839.x

BRISSON GR, 1986, HORM RES, V23, P200, DOI 10.1159/000180324

CASTALANO JF, 1973, PERCEPT MOTOR SKILL, V36, P363

Cian C, 2001, INT J PSYCHOPHYSIOL, V42, P243, DOI 10.1016/S0167-8760(01)00142-8

CRONIN MJ, 1976, BRAIN RES, V110, P175, DOI 10.1016/0006-8993(76)90219-5

Davis JM, 1997, MED SCI SPORT EXER, V29, P45, DOI 10.1097/00005768-199701000-00008

DAVIS JM, 1992, EUR J APPL PHYSIOL, V65, P513, DOI 10.1007/BF00602357

Davis JM, 1999, INT J SPORTS MED, V20, P309, DOI 10.1055/s-2007-971136

FINE BJ, 1994, PSYCHOPHARMACOLOGY, V114, P233, DOI 10.1007/BF02244842

HASSMEN P, 1994, NUTRITION, V10, P405

KAUFMAN S, 1983, J PHYSIOL-LONDON, V336, P73, DOI 10.1113/jphysiol.1983.sp014567

Kennedy MM, 1997, J SPORT MED PHYS FIT, V37, P200

Lieberman HR, 1998, BEHAV RES METH INS C, V30, P416, DOI 10.3758/BF03200674

Lieberman HR, 2002, PSYCHOPHARMACOLOGY, V164, P250, DOI 10.1007/s00213-002-1217-9

Lieberman HR, 2002, AM J CLIN NUTR, V76, P120, DOI 10.1093/ajcn/76.1.120

Madsen K, 1996, J APPL PHYSIOL, V81, P2644, DOI 10.1152/jappl.1996.81.6.2644

MELIN B, 1988, EUR J APPL PHYSIOL O, V58, P146, DOI 10.1007/BF00636618

Mittleman KD, 1998, MED SCI SPORT EXER, V30, P83, DOI 10.1097/00005768-199801000-00012

Montain SJ, 1998, J APPL PHYSIOL, V84, P1889, DOI 10.1152/jappl.1998.84.6.1889

Newsholme EA, 1987, ADV MYOCHEMISTRY

Nielsen B, 2001, PFLUG ARCH EUR J PHY, V442, P41, DOI 10.1007/s004240100515

Nybo L, 2003, MED SCI SPORT EXER, V35, P589, DOI 10.1249/01.MSS.0000058433.85789.66

Nybo L, 2001, J APPL PHYSIOL, V91, P1055, DOI 10.1152/jappl.2001.91.3.1055

Nybo L, 2003, J APPL PHYSIOL, V95, P1125, DOI 10.1152/japplphysiol.00241.2003

Pardridge WM, 1998, NEUROCHEM RES, V23, P635, DOI 10.1023/A:1022482604276

Pitsiladis YP, 2002, EXP PHYSIOL, V87, P215, DOI 10.1113/eph8702342

Popova N K, 2001, Neurosci Behav Physiol, V31, P327, DOI 10.1023/A:1010346904526

Radomski MW, 1998, CAN J PHYSIOL PHARM, V76, P547, DOI 10.1139/cjpp-76-5-547

ROLE L, 1991, PRINCIPLES NEURAL SC

SAWKA MN, 1992, J APPL PHYSIOL, V73, P368, DOI 10.1152/jappl.1992.73.1.368

SAWKA MN, 1985, J APPL PHYSIOL, V59, P1394, DOI 10.1152/jappl.1985.59.5.1394

SAWKA MN, 1996, HDB PHYSIOL SECT 4, V1, P157

SHARMA HS, 1987, BRAIN RES, V424, P153, DOI 10.1016/0006-8993(87)91205-4

Struder HK, 1996, INT J SPORTS MED, V17, P73, DOI 10.1055/s-2007-972811

Struder HK, 1998, HORM METAB RES, V30, P188, DOI 10.1055/s-2007-978864

VANHALL G, 1995, J PHYSIOL-LONDON, V486, P789, DOI 10.1113/jphysiol.1995.sp020854

VARNIER M, 1994, EUR J APPL PHYSIOL O, V69, P26, DOI 10.1007/BF00867923

WAGENMAKERS AJM, 1991, AM J PHYSIOL, V260, pE883, DOI 10.1152/ajpendo.1991.260.6.E883

WURTMAN RJ, 1988, MT SINAI J MED, V55, P75

WURTMAN RJ, 1994, FOOD COMPONENTS ENHA

YATHAM LN, 1993, LIFE SCI, V53, P447, DOI 10.1016/0024-3205(93)90696-Z

Young SN, 1986, NUTR BRAIN

NR 46

TC 52

Z9 55

U1 0

U2 12

PU AMER PHYSIOLOGICAL SOC

PI BETHESDA

PA 9650 ROCKVILLE PIKE, BETHESDA, MD 20814 USA

SN 8750-7587

J9 J APPL PHYSIOL

JI J. Appl. Physiol.

PD OCT

PY 2004

VL 97

IS 4

BP 1275

EP 1282

DI 10.1152/japplphysiol.00357.2004

PG 8

WC Physiology; Sport Sciences

WE Science Citation Index Expanded (SCI-EXPANDED)

SC Physiology; Sport Sciences

GA 859FB

UT WOS:000224247700014

PM 15358751

DA 2022-05-04

ER

PT J

AU Magana, VC

Scherz, WD

Seepold, R

Madrid, NM

Paneda, XG

Garcia, R

AF Corcoba Magana, Victor

Daniel Scherz, Wilhelm

Seepold, Ralf

Martinez Madrid, Natividad

Garcia Paneda, Xabiel

Garcia, Roberto

TI The Effects of the Driver's Mental State and Passenger Compartment

Conditions on Driving Performance and Driving Stress

SO SENSORS

LA English

DT Article

DE driving safety; driving emotions; driving stress; lifestyle; sensors;

heart rate

ID CARBON-DIOXIDE; VEHICLE; WORKLOAD; MUSIC; ANGER; VENTILATION; EXPOSURES;

ACCIDENTS; PATTERNS; EMOTION

AB Globalization has increased the number of road trips and vehicles. The result has been an intensification of traffic accidents, which are becoming one of the most important causes of death worldwide. Traffic accidents are often due to human error, the probability of which increases when the cognitive ability of the driver decreases. Cognitive capacity is closely related to the driver's mental state, as well as other external factors such as the CO(2)concentration inside the vehicle. The objective of this work is to analyze how these elements affect driving. We have conducted an experiment with 50 drivers who have driven for 25 min using a driving simulator. These drivers completed a survey at the start and end of the experiment to obtain information about their mental state. In addition, during the test, their stress level was monitored using biometric sensors and the state of the environment (temperature, humidity and CO(2)level) was recorded. The results of the experiment show that the initial level of stress and tiredness of the driver can have a strong impact on stress, driving behavior and fatigue produced by the driving test. Other elements such as sadness and the conditions of the interior of the vehicle also cause impaired driving and affect compliance with traffic regulations.

C1 [Corcoba Magana, Victor; Garcia Paneda, Xabiel; Garcia, Roberto] Univ Oviedo, Dept Comp Sci, Oviedo 33003, Spain.

[Daniel Scherz, Wilhelm; Seepold, Ralf] Univ Technol Business & Design Konstanz, Dept Comp Sci, Ubiquitous Comp Lab, D-78462 Constance, Germany.

[Seepold, Ralf; Martinez Madrid, Natividad] IM Sechenov First Moscow State Med Univ, Inst Digital Med, Moscow 119435, Russia.

[Martinez Madrid, Natividad] Reutlingen Univ, Sch Informat, IoT Lab, D-72762 Reutlingen, Germany.

RP Magana, VC (corresponding author), Univ Oviedo, Dept Comp Sci, Oviedo 33003, Spain.

EM corcobavictor@uniovi.es; wscherz@htwg-konstanz.de; ralf@ieee.org;

nati@ieee.org; xabiel@uniovi.es; garciaroberto@uniovi.es

RI Seepold, Ralf/Y-2510-2018; Martínez Madrid, Natividad/Y-2636-2018;

Magaña, Víctor Corcoba/AAZ-2273-2020

OI Seepold, Ralf/0000-0002-5192-2537; Martínez Madrid,

Natividad/0000-0003-1965-9481; Magaña, Víctor

Corcoba/0000-0001-6804-7428; Garcia-Fernandez,

Roberto/0000-0002-5042-8684; Garcia-Paneda, Xicu

Xabiel/0000-0001-6381-5459

FU Spanish National Research ProgramSpanish Government [TIN2017-82928-R]

FX This work was supported in part by the Spanish National Research Program

under Project TIN2017-82928-R.

CR Alkinani MH, 2020, IEEE ACCESS, V8, P105008, DOI 10.1109/ACCESS.2020.2999829

Allen JG, 2016, ENVIRON HEALTH PERSP, V124, P805, DOI 10.1289/ehp.1510037

[Anonymous], SMART IND AIR QUAL M

[Anonymous], P 19 AUSTR C COMP HU, DOI [10.1145/1324892.1324946, DOI 10.1145/1324892.1324946]

Balasubramanian V, 2007, J BODYW MOV THER, V11, P151, DOI 10.1016/j.jbmt.2006.12.005

Bellis E.A., 2008, SECURITY, P1

Bitkina OV, 2019, SENSORS-BASEL, V19, DOI 10.3390/s19092152

Braun Michael, 2018, Multimodal Technologies and Interaction, V2, DOI 10.3390/mti2040075

Brodsky W., 2001, TRANSPORT RES F, V4, P219, DOI [10.1016/S1369-8478(01)00025-0, DOI 10.1016/S1369-8478(01)00025-0, DOI 10.1016/S1369-8478(01)00025-ANDBEHAVIOUR0]

Can YS, 2019, SENSORS-BASEL, V19, DOI 10.3390/s19081849

Castaldo R, 2015, BIOMED SIGNAL PROCES, V18, P370, DOI 10.1016/j.bspc.2015.02.012

Chan AT, 2003, ATMOS ENVIRON, V37, P3795, DOI 10.1016/S1352-2310(03)00466-7

Coughlin J., 2009, TECHNICAL REPORT

Daanen HAM, 2003, APPL ERGON, V34, P597, DOI 10.1016/S0003-6870(03)00055-3

DEFFENBACHER JL, 1994, PSYCHOL REP, V74, P83, DOI 10.2466/pr0.1994.74.1.83

DEWAR RE, 1977, CAN PSYCHOL REV, V18, P365, DOI 10.1037/h0081453

Endsley MR, 2017, J COGN ENG DECIS MAK, V11, P225, DOI 10.1177/1555343417695197

Ericsson E, 2001, TRANSPORT RES D-TR E, V6, P325, DOI 10.1016/S1361-9209(01)00003-7

Fernandez A, 2016, SENSORS-BASEL, V16, DOI 10.3390/s16111805

Fountas G, 2019, ANAL METHODS ACCID R, V22, DOI 10.1016/j.amar.2019.100091

Frijda N.H., 1986, EMOTIONS

Funke G, 2007, ERGONOMICS, V50, P1302, DOI 10.1080/00140130701318830

Gao H, 2014, IEEE IMAGE PROC, P5961, DOI 10.1109/ICIP.2014.7026203

Georgiou Konstantinos, 2018, Folia Med (Plovdiv), V60, P7, DOI 10.2478/folmed-2018-0012

Giannakakis G, 2022, IEEE T AFFECT COMPUT, V13, P440, DOI 10.1109/TAFFC.2019.2927337

Gilgen-Ammann R, 2019, EUR J APPL PHYSIOL, V119, P1525, DOI 10.1007/s00421-019-04142-5

Gjoreski M, 2016, UBICOMP'16 ADJUNCT: PROCEEDINGS OF THE 2016 ACM INTERNATIONAL JOINT CONFERENCE ON PERVASIVE AND UBIQUITOUS COMPUTING, P1185, DOI 10.1145/2968219.2968306

Goh CC, 2016, INT CONF ELECTRON D, P427, DOI 10.1109/ICED.2016.7804682

Greenblatt NA, 2016, IEEE SPECTRUM, V53, P46, DOI 10.1109/MSPEC.2016.7419800

GULIAN E, 1989, ERGONOMICS, V32, P585, DOI 10.1080/00140138908966134

Halim Z, 2020, INFORM FUSION, V53, P66, DOI 10.1016/j.inffus.2019.06.006

Hancock PA, 1997, ACCIDENT ANAL PREV, V29, P495, DOI 10.1016/S0001-4575(97)00029-8

Hanowski R.J., ANAL RISK FUNCTION D

Hansen JHL, 1996, SPEECH COMMUN, V20, P151, DOI 10.1016/S0167-6393(96)00050-7

HART S G, 1988, P139

Hartley L.R., 2019, FATIGUE DRIVING DRIV

Healey JA, 2005, IEEE T INTELL TRANSP, V6, P156, DOI 10.1109/TITS.2005.848368

Herrington J.D., 1996, J SERV MARK, V10, P26, DOI [10.1108/08876049610114249, DOI 10.1108/08876049610114249]

Hu TY, 2013, TRANSPORT RES F-TRAF, V16, P29, DOI 10.1016/j.trf.2012.08.009

Huffziger S, 2009, BEHAV RES THER, V47, P224, DOI 10.1016/j.brat.2008.12.005

Izquierdo-Reyes J, 2018, INT J INTERACT DES M, V12, P1447, DOI 10.1007/s12008-018-0473-9

Jallais C, 2014, TRANSPORT RES F-TRAF, V23, P125, DOI 10.1016/j.trf.2013.12.023

Jeon M., 2013, P HUM FACT ERG SOC A, P1849, DOI [10.1177/1541931213571413., DOI 10.1177/1541931213571413, 10.1177/1541931213571413]

Jeon M, 2016, INT J HUM-COMPUT INT, V32, P777, DOI 10.1080/10447318.2016.1198524

Joshi A., 2015, CURR J APPL SCI TECH, V7, P396, DOI [10.9734/BJAST/2015/14975, DOI 10.9734/BJAST/2015/14975]

Jovanis P.P., HOURS SERVICE DRIVER

Jung HS, 2017, ATMOS ENVIRON, V160, P77, DOI 10.1016/j.atmosenv.2017.04.014

Kajtar L., 2003, P 7 INT C HLTH BUILD, V3, P176

Kajtar L, 2012, IDOJARAS, V116, P145

Kaplan S, 2015, IEEE T INTELL TRANSP, V16, DOI 10.1109/TITS.2015.2462084

Kim J, 2020, HUM FACTOR ERGON MAN, V30, P321, DOI 10.1002/hfm.20843

Kontogiannis T, 2006, ACCIDENT ANAL PREV, V38, P913, DOI 10.1016/j.aap.2006.03.002

Kreibig SD, 2007, PSYCHOPHYSIOLOGY, V44, P787, DOI 10.1111/j.1469-8986.2007.00550.x

Kuribayashi R, 2015, PSYCHOL MUSIC, V43, P808, DOI 10.1177/0305735614543216

Lanata A, 2015, IEEE T INTELL TRANSP, V16, P1505, DOI 10.1109/TITS.2014.2365681

Lazarus R.S., 1991, EMOTION ADAPTATION

Lazarus R.S., 1984, STRESS APPRAISAL COP

Lee BG, 2017, IEEE T INTELL TRANSP, V18, P1835, DOI 10.1109/TITS.2016.2617881

Lee BG, 2015, IEEE ENG MED BIO, P6126, DOI 10.1109/EMBC.2015.7319790

Legree PJ, 2003, J APPL PSYCHOL, V88, P15, DOI 10.1037/0021-9010.88.1.15

Limyati Y., 2019, J MED HLTH, V2, DOI [10.28932/jmh.v2i4.1127, DOI 10.28932/JMH.V2I4.1127]

LOCKHART RA, 1972, PSYCHOPHYSIOLOGY, V9, P437, DOI 10.1111/j.1469-8986.1972.tb01791.x

Loft S, 2007, HUM FACTORS, V49, P376, DOI 10.1518/001872007X197017

Lu JY, 2013, J SAFETY RES, V45, P65, DOI 10.1016/j.jsr.2013.01.009

Lutin J., 2013, CELL, V215, P630

MACKAY C, 1978, BRIT J SOC CLIN PSYC, V17, P283, DOI 10.1111/j.2044-8260.1978.tb00280.x

MacNaughton P, 2016, BUILD ENVIRON, V104, P138, DOI 10.1016/j.buildenv.2016.05.011

Malik M., 1996, AQUACULTURE, V1, P151, DOI DOI 10.1111/J.1542-474X.1996.TB00275.X

Marksberry K., WHAT IS STRESS

Matthews G., 2020, PERSONAL INDIVID DIF, DOI 10.1016/j.paid.2020.110083

Matthews G., COMPREHENSIVE QUESTI

MAYFIELD C, 1989, PSYCHOL REP, V65, P1283, DOI 10.2466/pr0.1989.65.3f.1283

Mayou R, 2003, INJURY, V34, P197, DOI 10.1016/S0020-1383(02)00285-1

McCarthy C., 2020, P 2016 IEEE EMBS INT, P1

McMurray L., 1970, BEHAV RES HIGHWAY SA, V1, P100

Meiring GAM, 2015, SENSORS-BASEL, V15, P30653, DOI 10.3390/s151229822

Millet B, 2019, TRANSPORT RES F-TRAF, V60, P743, DOI 10.1016/j.trf.2018.10.007

Milstein N, 2020, FRONT BEHAV NEUROSCI, V14, DOI 10.3389/fnbeh.2020.00148

Mladenovic MN, 2014, 2014 IEEE INTERNATIONAL SYMPOSIUM ON ETHICS IN SCIENCE, TECHNOLOGY AND ENGINEERING

Munla N, 2015, I CON ADV BIOMED ENG, P61, DOI 10.1109/ICABME.2015.7323251

Naevestad TO, 2019, SAFETY, V5, DOI 10.3390/safety5020020

Nelson BW, 2019, JMIR MHEALTH UHEALTH, V7, DOI 10.2196/10828

Norris FH, 2000, ACCIDENT ANAL PREV, V32, P505, DOI 10.1016/S0001-4575(99)00068-8

North AC, 1999, ENVIRON BEHAV, V31, P136, DOI 10.1177/00139169921972038

Oakes S., 2000, J SERV MARK, V14, P539, DOI DOI 10.1108/08876040010352673

Pauzie A, 2008, IET INTELL TRANSP SY, V2, P315, DOI 10.1049/iet-its:20080023

Petersen J., 2018, P INT C BUILD EN ENV, P203

Pruetz J., 2019, ASSESSMENT AUTOMOTIV, DOI [10.4271/2019-01-1597, DOI 10.4271/2019-01-1597]

Reimer B., 2010, TECHNICAL REPORT

Rendon-Velez E, 2016, TRANSPORT RES F-TRAF, V41, P150, DOI 10.1016/j.trf.2016.06.013

Rodjegard H., 2020, DROWSY DRIVER CHILD

Rony Rahat Jahangir, 2019, 2019 11th International Conference on Communication Systems & Networks (COMSNETS), P417, DOI 10.1109/COMSNETS.2019.8711411

Satish U, 2012, ENVIRON HEALTH PERSP, V120, P1671, DOI 10.1289/ehp.1104789

Serban G., 1976, PSYCHOPATHOLOGY HUMA, P137, DOI DOI 10.1007/978-1-4684-2238-2_9

Sikander G, 2019, IEEE T INTELL TRANSP, V20, P2339, DOI 10.1109/TITS.2018.2868499

Simion M, 2016, ENRGY PROCED, V85, P472, DOI 10.1016/j.egypro.2015.12.229

Snow S., 2018, USING EEG CHARACTERI, DOI [10.1101/483750, DOI 10.1101/483750]

Stern HS, 2019, ACCIDENT ANAL PREV, V126, P37, DOI 10.1016/j.aap.2018.02.021

Sun XL, 2018, TRANSPORT RES F-TRAF, V59, P436, DOI 10.1016/j.trf.2018.09.022

Westerman SJ, 2000, PERS INDIV DIFFER, V29, P981, DOI 10.1016/S0191-8869(99)00249-4

Worner D, 2014, 2014 INTERNATIONAL CONFERENCE ON THE INTERNET OF THINGS (IOT), P25, DOI 10.1109/IOT.2014.7030110

Yamakoshi T, 2008, IEEE ENG MED BIO, P1076, DOI 10.1109/IEMBS.2008.4649346

Ying Shi, 2011, 2011 IEEE 18th International Conference on Industrial Engineering and Engineering Management (IE&EM 2011), P1424, DOI 10.1109/ICIEEM.2011.6035423

Zimasa T, 2019, TRANSPORT RES F-TRAF, V66, P87, DOI 10.1016/j.trf.2019.08.019

ZLATOPER TJ, 1991, ACCIDENT ANAL PREV, V23, P431, DOI 10.1016/0001-4575(91)90062-A

NR 105

TC 3

Z9 3

U1 5

U2 18

PU MDPI

PI BASEL

PA ST ALBAN-ANLAGE 66, CH-4052 BASEL, SWITZERLAND

EI 1424-8220

J9 SENSORS-BASEL

JI Sensors

PD SEP

PY 2020

VL 20

IS 18

AR 5274

DI 10.3390/s20185274

PG 31

WC Chemistry, Analytical; Engineering, Electrical & Electronic; Instruments

& Instrumentation

WE Science Citation Index Expanded (SCI-EXPANDED); Social Science Citation Index (SSCI)

SC Chemistry; Engineering; Instruments & Instrumentation

GA OE8NP

UT WOS:000580780800001

PM 32942684

OA Green Submitted, gold, Green Published

DA 2022-05-04

ER

PT J

AU Monteiro, TG

Skourup, C

Zhang, HX

AF Monteiro, Thiago Gabriel

Skourup, Charlotte

Zhang, Houxiang

TI Using EEG for Mental Fatigue Assessment: A Comprehensive Look Into the

Current State of the Art

SO IEEE TRANSACTIONS ON HUMAN-MACHINE SYSTEMS

LA English

DT Article

DE Electroencephalography; Feature extraction; Human factors; Sensor

fusion; Fatigue; Risk management; Electroencephalogram (EEG); human

factors; human-machine systems; mental fatigue (MF) assessment; risk

assessment; sensor fusion

ID EPILEPTIC SEIZURE DETECTION; DROWSINESS DETECTION; AUTOMATIC DETECTION;

DRIVER DROWSINESS; DETECTION SYSTEM; WEARABLE EEG; SIGNALS; ENTROPY;

MODEL; SLEEPINESS

AB This paper provides a brief survey of recent developments on the use of electroencephalogram (EEG) sensors for detecting mental fatigue (MF) in human operators during tasks involving human-machine interaction. This research topic has received much attention since there is a consensus among experts on the increasing relation between human failure and accidents in safety-critical tasks. MF is one of the most influential aspects leading to human failure and the most reliable way to assess it is using operators physiological data, especially EEG. In the past few decades, hundreds of publications have explored the use of EEG alone or together with other objective and subjective measures for assessing MF, drowsiness, and tiredness in human operators. With recent improvements in data preprocessing, feature extraction, and classification algorithms, the monitoring and mitigation of MF in real time has become a reality. This trend is mainly due to the increasing use of machine learning techniques. This paper provides a comprehensive look at the current state of the art in the field of MF detection using EEG, identifying the currently used technique, algorithms, and methods and possible trends and promising areas for further research. The paper is concluded by suggesting a kernel partial least squares discrete-output linear regression based model as an all-around good option for an MF assessment system.

C1 [Monteiro, Thiago Gabriel; Zhang, Houxiang] Norwegian Univ Sci & Technol, Dept Ocean Operat & Civil Engn, N-6009 Alesund, Norway.

[Skourup, Charlotte] ABB AS, Prod & Serv R&D Oil Gas & Chem, N-0603 Oslo, Norway.

RP Monteiro, TG (corresponding author), Norwegian Univ Sci & Technol, Dept Ocean Operat & Civil Engn, N-6009 Alesund, Norway.

EM thiago.g.monteiro@ntnu.no; charlotte.skourup@no.abb.com; hozh@ntnu.no

OI Zhang, Houxiang/0000-0003-0122-0964; Monteiro, Thiago

Gabriel/0000-0002-2496-423X

FU Norway Research Council, Norway [237929]

FX This work was supported in part by the project "SFI

OffshoreMechatronics" funded by Norway Research Council, Norway (Project

237896) and in part by the project "SFI Marine Operations" funded by

Norway Research Council, Norway (Project 237929). This article was

recommended by Associate Editor L. Contreras-Vidal.

CR Acharya UR, 2012, BIOMED SIGNAL PROCES, V7, P401, DOI 10.1016/j.bspc.2011.07.007

Ahn S, 2016, FRONT HUM NEUROSCI, V10, DOI 10.3389/fnhum.2016.00219

Ahvenjarvi S, 2016, TRANSNAV, V10, P517, DOI 10.12716/1001.10.03.18

AKERSTEDT T, 1990, INT J NEUROSCI, V52, P29, DOI 10.3109/00207459008994241

Alotaiby TN, 2014, EURASIP J ADV SIG PR, DOI 10.1186/1687-6180-2014-183

Avers K., 2011, AVIAT PSYCHOL APPL H, V1, P87, DOI [DOI 10.1027/2192-0923/A000016, 10.1027/2192-0923/a000016]

Awais M, 2017, SENSORS-BASEL, V17, DOI 10.3390/s17091991

Bi LZ, 2013, IEEE T HUM-MACH SYST, V43, P161, DOI 10.1109/TSMCC.2012.2219046

Cao L, 2016, COGN COMPUT, V8, P228, DOI 10.1007/s12559-015-9351-y

Chai RF, 2017, FRONT NEUROSCI-SWITZ, V11, DOI 10.3389/fnins.2017.00103

Chai RF, 2017, IEEE J BIOMED HEALTH, V21, P715, DOI 10.1109/JBHI.2016.2532354

CHALDER T, 1993, J PSYCHOSOM RES, V37, P147, DOI 10.1016/0022-3999(93)90081-P

Chatrian G. E., 1985, American Journal of EEG Technology, V25, P83

Chauvin C, 2013, ACCIDENT ANAL PREV, V59, P26, DOI 10.1016/j.aap.2013.05.006

Chen LL, 2015, EXPERT SYST APPL, V42, P7344, DOI 10.1016/j.eswa.2015.05.028

Chuang CH, 2015, KNOWL-BASED SYST, V80, P143, DOI 10.1016/j.knosys.2015.01.007

Chuang CH, 2014, IEEE T NEUR SYS REH, V22, P230, DOI 10.1109/TNSRE.2013.2293139

Chuang SW, 2012, NEUROIMAGE, V62, P1469, DOI 10.1016/j.neuroimage.2012.05.035

Colic A., 2014, DRIVERDROWSINESSDETE

Craig A, 2012, PSYCHOPHYSIOLOGY, V49, P574, DOI 10.1111/j.1469-8986.2011.01329.x

da Silveira TLT, 2016, EXPERT SYST APPL, V55, P559, DOI 10.1016/j.eswa.2016.02.041

Delorme A, 2004, J NEUROSCI METH, V134, P9, DOI 10.1016/j.jneumeth.2003.10.009

Forsman PM, 2013, ACCIDENT ANAL PREV, V50, P341, DOI 10.1016/j.aap.2012.05.005

Correa AG, 2014, MED ENG PHYS, V36, P244, DOI 10.1016/j.medengphy.2013.07.011

Gerla M, 2014, 2014 IEEE WORLD FORUM ON INTERNET OF THINGS (WF-IOT), P241, DOI 10.1109/WF-IoT.2014.6803166

Giraudet L, 2015, BEHAV BRAIN RES, V294, P246, DOI 10.1016/j.bbr.2015.07.041

Guo L, 2010, J NEUROSCI METH, V193, P156, DOI 10.1016/j.jneumeth.2010.08.030

Guo MZ, 2016, INT J ENV RES PUB HE, V13, DOI 10.3390/ijerph13121174

Hajinoroozi M, 2016, SIGNAL PROCESS-IMAGE, V47, P549, DOI 10.1016/j.image.2016.05.018

HART S G, 1988, P139

He B, 2013, NEURAL ENG, P87, DOI [10.1088/1741-2560/10/4/046003, DOI 10.1088/1741-2560/10/4/046003, DOI 10.1007/978-1-4614-5227-0_2]

He QC, 2015, IET INTELL TRANSP SY, V9, P547, DOI 10.1049/iet-its.2014.0103

Hefron RG, 2017, PATTERN RECOGN LETT, V94, P96, DOI 10.1016/j.patrec.2017.05.020

Hockey G. R. J., 2003, OPERATOR FUNCTIONAL

Hu JF, 2017, ENTROPY-SWITZ, V19, DOI 10.3390/e19080385

Hu JF, 2017, FRONT COMPUT NEUROSC, V11, DOI 10.3389/fncom.2017.00072

Hu JF, 2017, COMPUT MATH METHOD M, V2017, DOI 10.1155/2017/5109530

Hu SY, 2013, IET INTELL TRANSP SY, V7, P105, DOI 10.1049/iet-its.2012.0045

JOHNS MW, 1991, SLEEP, V14, P540, DOI 10.1093/sleep/14.6.540

Jung TP, 2001, P IEEE, V89, P1107, DOI 10.1109/5.939827

Kandaswamy A, 2005, IETE J RES, V51, P121, DOI 10.1080/03772063.2005.11416387

Kannathal N, 2005, COMPUT METH PROG BIO, V80, P187, DOI 10.1016/j.cmpb.2005.06.012

Kaur R., 2013, INT J SCI RES, V10, P157

Kim DS, 2013, SAFETY SCI, V60, P57, DOI 10.1016/j.ssci.2013.06.010

Klem G H, 1999, Electroencephalogr Clin Neurophysiol Suppl, V52, P3

Langkvist M, 2014, PATTERN RECOGN LETT, V42, P11, DOI 10.1016/j.patrec.2014.01.008

Laurent F, 2013, BIOMED SIGNAL PROCES, V8, P400, DOI 10.1016/j.bspc.2013.01.007

Lawhern V, 2013, BMC NEUROSCI, V14, DOI 10.1186/1471-2202-14-101

Lee BG, 2014, SENSORS-BASEL, V14, P17915, DOI 10.3390/s141017915

LI GP, 2013, SENSORS, V15, P2002, DOI DOI 10.1021/OL4006823

Liang SF, 2012, IEEE T INSTRUM MEAS, V61, P1649, DOI 10.1109/TIM.2012.2187242

Lin CT, 2014, IEEE T BIOMED CIRC S, V8, P165, DOI 10.1109/TBCAS.2014.2316224

Liu JP, 2010, BIOMED SIGNAL PROCES, V5, P124, DOI 10.1016/j.bspc.2010.01.001

Liu YT, 2016, IEEE T NEUR NET LEAR, V27, P347, DOI 10.1109/TNNLS.2015.2496330

Menon V, 2000, NEUROIMAGE, V12, P357, DOI 10.1006/nimg.2000.0613

Min JL, 2017, PLOS ONE, V12, DOI 10.1371/journal.pone.0188756

Mu ZD, 2017, INT J PATTERN RECOGN, V31, DOI 10.1142/S0218001417500112

Mu ZD, 2017, APPL SCI-BASEL, V7, DOI 10.3390/app7020150

Mu ZD, 2016, ENTROPY-SWITZ, V18, DOI 10.3390/e18120432

Nolan H, 2010, J NEUROSCI METH, V192, P152, DOI 10.1016/j.jneumeth.2010.07.015

Oostenveld R, 2001, CLIN NEUROPHYSIOL, V112, P713, DOI 10.1016/S1388-2457(00)00527-7

Pathak M, 2017, BIOMED ENG-APP BAS C, V29, DOI 10.4015/S1016237217500193

Razavipour F, 2014, ARAB J SCI ENG, V39, P7049, DOI 10.1007/s13369-014-1242-0

Reinerman-Jones L, 2016, SAFETY SCI, V88, P97, DOI 10.1016/j.ssci.2016.05.002

Resalat SN, 2015, SIGNAL IMAGE VIDEO P, V9, P1751, DOI 10.1007/s11760-015-0760-x

Rohit F, 2017, IET INTELL TRANSP SY, V11, P255, DOI 10.1049/iet-its.2016.0183

Sahayadhas A, 2012, SENSORS-BASEL, V12, P16937, DOI 10.3390/s121216937

Sauvet F, 2014, IEEE T BIO-MED ENG, V61, P2840, DOI 10.1109/TBME.2014.2331189

Shamseer L, 2015, BMJ-BRIT MED J, V349, DOI [10.1136/bmj.g7647, 10.1136/bmj.b2700, 10.1016/j.ijsu.2010.02.007, 10.1136/bmj.b2535]

Silveira Tiago da, 2015, Res. Biomed. Eng., V31, P107, DOI 10.1590/2446-4740.0693

Song YD, 2012, J NEUROSCI METH, V210, P132, DOI 10.1016/j.jneumeth.2012.07.003

Soria-Frisch A., 2012, PRACTICAL BRAIN COMP, P41

Stoica P., 2005, SPECTRAL ANAL SIGNAL, V1

Suarez E, 2000, BIOMED SCI INSTRUM, V395, P33

Subha DP, 2010, J MED SYST, V34, P195, DOI 10.1007/s10916-008-9231-z

Sun Y, 2014, ANN BIOMED ENG, V42, P2084, DOI 10.1007/s10439-014-1059-8

Suraweera P, 2013, TRANSPORT RES C-EMER, V26, P214, DOI 10.1016/j.trc.2012.08.002

Ting CH, 2010, IEEE T SYST MAN CY A, V40, P251, DOI 10.1109/TSMCA.2009.2035301

Trejo L. J., 2015, PSYCHOLOGY, V6, P572, DOI [10.4236/psych.2015.65055, DOI 10.4236/PSYCH.2015.65055]

Nguyen T, 2017, SCI REP-UK, V7, DOI 10.1038/srep39980

Walger D. J., 2014, COMP INT MULT UND IW, P1

Wang H, 2015, INT J NEURAL SYST, V25, DOI 10.1142/S0129065715500021

Williamson A, 2011, ACCIDENT ANAL PREV, V43, P498, DOI 10.1016/j.aap.2009.11.011

Wu DR, 2017, IEEE T FUZZY SYST, V25, P1522, DOI 10.1109/TFUZZ.2016.2633379

Xiong YJ, 2013, APPL MECH MATER, V373-375, P965, DOI 10.4028/www.scientific.net/AMM.373-375.965

Xu W, 2016, INT CONF UTIL CLOUD, P321, DOI [10.1145/2996890.3007881, 10.1145/2996890.30078810]

Yang ZL, 2013, MARIT POLICY MANAG, V40, P261, DOI 10.1080/03088839.2013.782952

Yin BC, 2009, INT J PATTERN RECOGN, V23, P575, DOI 10.1142/S021800140900720X

Yin Z., 2017, NEUROCOMPUTING

Yin Z, 2017, BIOMED SIGNAL PROCES, V33, P30, DOI 10.1016/j.bspc.2016.11.013

Zhang C, 2014, IEEE T INTELL TRANSP, V15, P168, DOI 10.1109/TITS.2013.2275192

Zhang JH, 2015, IEEE T HUM-MACH SYST, V45, P200, DOI 10.1109/THMS.2014.2366914

Zhang XL, 2017, SENSORS-BASEL, V17, DOI 10.3390/s17030486

Zhang ZT, 2016, SENSORS-BASEL, V16, DOI 10.3390/s16020242

Zheng WL, 2017, J NEURAL ENG, V14, DOI 10.1088/1741-2552/aa5a98

Zhu WB, 2017, MATH PROBL ENG, V2017, DOI 10.1155/2017/6191035

NR 96

TC 15

Z9 15

U1 7

U2 37

PU IEEE-INST ELECTRICAL ELECTRONICS ENGINEERS INC

PI PISCATAWAY

PA 445 HOES LANE, PISCATAWAY, NJ 08855-4141 USA

SN 2168-2291

EI 2168-2305

J9 IEEE T HUM-MACH SYST

JI IEEE T. Hum.-Mach. Syst.

PD DEC

PY 2019

VL 49

IS 6

BP 599

EP 610

DI 10.1109/THMS.2019.2938156

PG 12

WC Computer Science, Artificial Intelligence; Computer Science, Cybernetics

WE Science Citation Index Expanded (SCI-EXPANDED)

SC Computer Science

GA JV3RT

UT WOS:000502284100014

OA Green Submitted

DA 2022-05-04

ER

PT J

AU Kuroda, M

Ishizaki, T

Maruyama, T

Takatsuka, Y

Kuboki, T

AF Kuroda, Motonaka

Ishizaki, Taichi

Maruyama, Tomoaki

Takatsuka, Yoji

Kuboki, Tomifusa

TI Effect of dried-bonito broth on mental fatigue and mental task

performance in subjects with a high fatigue score

SO PHYSIOLOGY & BEHAVIOR

LA English

DT Article

DE dried-bonito; POMS; Uchida-Kraepelin test; fatigue; task performance

ID ANTIOXIDANT ACTIVITY; MOOD; PHYSIOLOGY; STATES
[truncated: 4,469,082 more chars]
